# Online dissemination of Cochrane reviews on digital health technologies: a cross-sectional study

**DOI:** 10.1186/s13643-024-02557-6

**Published:** 2024-05-15

**Authors:** Karina Karolina De Santis, Mathia Kirstein, Christina Kien, Ursula Griebler, Sam McCrabb, Tina Jahnel

**Affiliations:** 1 Department of Prevention and Evaluation, Leibniz Institute for Prevention Research and Epidemiology — BIPS, Bremen, 28359 Germany; 2 Department for Evidence-Based Medicine and Evaluation, University for Continuing Education Krems, Krems, Austria; 3 Faculty of Health and Medicine, School of Medicine and Public Health, University of Newcastle, Callaghan, NSW Australia; 4 Department of Health Services Research, Faculty 11 Human and Health Sciences, University of Bremen, Bremen, Germany

**Keywords:** Cochrane, Review, Digital technology, Healthcare, Public health, Cross-sectional, Dissemination, Knowledge translation, Altmetric score

## Abstract

**Background:**

This cross-sectional study investigated the online dissemination of Cochrane reviews on digital health technologies.

**Methods:**

We searched the Cochrane Database of Systematic Reviews from inception up to May 2023. Cochrane reviews with any population (P), intervention or concept supported by any digital technology (I), any or no comparison (C), and any health outcome (O) were included. Data on review characteristics (bibliographic information, PICO, and evidence quality) and dissemination strategies were extracted and processed. Dissemination was assessed using review information on the Cochrane website and Altmetric data that trace the mentions of academic publications in nonacademic online channels. Data were analysed using descriptive statistics and binary logistic regression analysis.

**Results:**

Out of 170 records identified in the search, 100 Cochrane reviews, published between 2005 and 2023, were included. The reviews focused on consumers (e.g. patients, *n* = 86), people of any age (*n* = 44), and clinical populations (*n* = 68). All reviews addressed interventions or concepts supported by digital technologies with any devices (*n* = 73), mobile devices (*n* = 17), or computers (*n* = 10). The outcomes focused on disease treatment (*n* = 56), health promotion and disease prevention (*n* = 27), or management of care delivery (*n* = 17). All reviews included 1–132 studies, and half included 1–10 studies. Meta-analysis was performed in 69 reviews, and certainty of evidence was rated as high or moderate for at least one outcome in 46 reviews.

In agreement with the Cochrane guidelines, all reviews had a plain language summary (PLS) that was available in 3–14 languages. The reviews were disseminated (i.e. mentioned online) predominantly via X/Twitter (*n* = 99) and Facebook (*n* = 69). Overall, 51 reviews were mentioned in up to 25% and 49 reviews in 5% of all research outputs traced by Altmetric data. Dissemination (i.e. higher Altmetric scores) was associated with bibliographic review characteristics (i.e. earlier publication year and PLS available in more languages), but not with evidence quality (i.e. certainty of evidence rating, number of studies, or meta-analysis performed in review).

**Conclusions:**

Online attention towards Cochrane reviews on digital health technologies is high. Dissemination is higher for older reviews and reviews with more PLS translations. Measures are required to improve dissemination of Cochrane reviews based on evidence quality.

**Systematic review registration:**

The study was prospectively registered at the Open Science Framework (https://osf.io/mpw8u/).

**Supplementary Information:**

The online version contains supplementary material available at 10.1186/s13643-024-02557-6.

## Background

### Digital health technologies

Digital technologies have revolutionised healthcare and public health by offering unprecedented opportunities for improved medical interventions and diagnostics as well as monitoring of health [1, 2]. Digital technologies, such as smartphones and wearables, can assist healthcare decisions, including more accurate and timely diagnoses [3], and contribute to health promotion and disease prevention by empowering their users to take an active role in managing their health via access to personalised health information and self-monitoring [4]. Despite the growing accessibility of digital technologies, a persistent digital divide exists that limits the equitable access to innovations due to socioeconomic factors, geographical disparities, and varying levels of technological literacy [5]. Furthermore, digital technologies require systematic evaluation to investigate if they contribute to any health benefits in their users [6].

Scientific evidence required to evaluate health outcomes of digital technologies can be synthesised using systematic reviews. Systematic reviews play a crucial role as a valuable source of evidence for stakeholders, including policy makers, clinicians, researchers, and the general population. Among systematic reviews, Cochrane reviews employ high methodological standards [7] and are therefore especially valuable for stakeholders. Considering the rapid development of digital health, it is important to assess how Cochrane reviews addressed this topic so far, in terms of digital technology types, target populations, and health outcomes. Furthermore, it is also of interest to investigate how these reviews are disseminated online to improve their impact by potentially reaching any relevant stakeholders [8, 9].

### Dissemination and analysis of its impact

Dissemination is defined as a proactive method of distributing scientific evidence to a specific audience through selected channels and planned strategies [10]. While peer-reviewed publications and conference presentations are effective strategies for disseminating research to academic audiences, other strategies, such as plain language summaries (PLS), policy briefs, reports, blogs, and communication via social media, may be better suited to reach policy makers or the interested public [11–13].

As there are a multitude of channels which can be used to disseminate research, different measures can be used to assess impact of academic publications. For example, the number of citations of an academic publication is one method for estimating its academic impact. Different measures are needed to objectively assess the nonacademic impact of academic publications due to multiple and heterogeneous channels that could be used for dissemination. As such, the nonacademic impact of academic publications could be assessed by examining citations in policy documents, reports, guidelines, and mentions on social media. Altmetric data provide one method of tracing mentions of academic publications in nonacademic online channels [14] and thus could be used to objectively quantify the nonacademic impact of such publications.

### Study aims

The aim of this study was to investigate the online dissemination of Cochrane reviews on digital health technologies. This study addressed the following research questions:

1. What are the characteristics of Cochrane reviews on digital technology use in healthcare and public health, including bibliographic, PICO (Population, Intervention, Comparison, Outcome), and evidence quality characteristics?
2. How are such Cochrane reviews disseminated online?

## Methods

### Study design

This study is a meta-research (i.e. research on research) study. We used Cochrane reviews as the unit of analysis and a cross-sectional design to collect and analyse data from such reviews (i.e. data on review content and review dissemination). A protocol for this study was prospectively registered at the Open Science Framework [15]. The study adheres to the Strengthening the Reporting of Observational Studies in Epidemiology (STROBE) guideline [16]. The STROBE checklist is reported in Additional file 1. There were no changes to the original protocol.

### Data source

The data source for this study was the Cochrane Database of Systematic Reviews [17]. This database listed 9033 records on 10 May 2023, including reviews with systematic methodology (e.g. systematic, scoping, or rapid reviews) and review protocols on any topics within healthcare and public health.

### Eligibility criteria

This study included Cochrane reviews with any methodology (e.g. systematic reviews) published up to 10 May 2023. The eligibility criteria for this study were defined based on the PICO (Population, Intervention, Comparison, Outcome) framework. The inclusion criteria were as follows: (1) any human population (P), (2) intervention or concept supported by any digital technology (I), (3) any or no comparison (C), and (4) any health outcome (O). The exclusion criteria were as follows: (1) nonhuman populations, (2) no focus on digital technologies, (3) no health outcome, and (4) other Cochrane publications (e.g. review protocols due to lack of data or original reviews if review update was published to reduce bias due to duplication).

### Data collection process

Data collection process involved the electronic search for Cochrane reviews, selection of eligible reviews, and data extraction from the included reviews.

#### Electronic search

The search strategy was created in consultation with a professional librarian and is reported in Additional file 2. The search for Cochrane reviews was performed by one researcher (M. K.) on 10 May 2023. All search results were imported to EndNote 20 (Clarivate) for study management, downloaded as full-text documents, and stored for further processing.

#### Review selection

Three researchers (M. K., T. J., K. K. D. S.) selected the reviews. One researcher (M. K.) performed the title/abstract and full-text screening. Two researchers (T. J. and K. K. D. S.) checked all excluded reviews to reduce the selection bias. The final consensus was reached by discussion between two researchers (M. K. and K. K. D. S.).

#### Data extraction

A data extraction sheet was developed in Excel 10 (Microsoft Inc.), pilot-tested using two reviews, and calibrated within the team. One researcher (M. K.) extracted all data from all reviews. To reduce bias in data extraction, team assistants helped with data entry and checked the extracted data in 10% of reviews. As no errors were detected, no further checks were performed.

### Variables (data items) and data processing

A list of variables (data items) for data extraction was developed by the team (Table 1).

**Table 1:** Data items

Item	Item content	Item details
**Review characteristics**		
1	Bibliographic	First author, title, aim, publication year, region of the corresponding author, and review type (e.g. systematic)
2	Population	Type: Consumers (clients or patients), healthcare professionals, informal carers; consumer age (any age, adults only, or children only); consumer health status (any health status: clinical or healthy, clinical only, or healthy only; if clinical, disease group, e.g. cardiovascular)
3	Intervention or concept	Type by modality: Digital only (i.e. interventions or concepts with single or multiple digital technologies) or mixed (i.e. interventions or concepts with digital and non-digital components); type by digital technology: mobile via mobile devices, non-mobile via non-mobile devices, or any mobile or non-mobile; digital device type (e.g. mobile phone, computer or wearable); interaction between users and digital technologies (e.g. via apps, text messages, or emails)
4	Comparison	Type by modality (all non-digital, at least one digital comparison, or no comparison)
5	Outcome	Focus, e.g. treatment, disease monitoring, or health promotion
6	Evidence quality	Number of studies included in review, meta-analysis performed in review, certainty of evidence rating based on the GRADE approach (number of quantitative outcomes rated, number of outcomes with strong, moderate, low, or very low certainty of evidence ratings)
**Dissemination strategies**		
7	Via Cochrane website	Plain language summary (PLS) languages, citation in clinical guidelines
8	Via Altmetric data	Altmetric score, Altmetric score interpretation, and number and type of channels traced by Altmetric data (e.g. number of mentions on Wikipedia)

Data items 1 to 5 (Table 1) were extracted as quantitative information (e.g. publication year) or qualitative, verbatim statements (e.g. description of digital technologies according to review authors) from the full text of the individual reviews. The qualitative data were subsequently processed into quantitative categories based on meaningful themes that inductively emerged from the data. For example, we assigned digital technologies into categories ‘mobile technologies’ or ‘nonmobile technologies’ based on the digital devices described in reviews. During data processing, we also considered the heterogeneous terminology used by review authors. For example, ‘mobile phone’ was used as a category to describe any portable telephone, including mobile phones, mobile telephones, videophones, smartphones, or cell phones. To reduce any biases in data processing, two researchers (M. K. and K. K. D. S.) discussed and agreed on all categories, one researcher (M. K.) processed all data, and another researcher (K. K. D. S.) checked all processed data. Any discrepancies were discussed, and the final agreement was reached by consensus between both researchers.

Data items 6 to 8 (Table 1) were extracted as quantitative information from the Cochrane website and the Altmetric data available open-access online. The Cochrane website lists various details of each Cochrane review, including a review abstract and a link to a full-text document, a plain language summary (PLS) in English that is a mandatory part of any Cochrane review, any translations of the PLS, and other review information (e.g. the number of clinical guidelines that cited the review and Altmetric data for each review). The Altmetric data are used to compute the Altmetric (attention) score that measures the attention towards academic publications by tracing their mentions in nonacademic online channels [14]. Therefore, it provides an objective method to quantify and explore online dissemination channels of academic publications. The Altmetric score is a weighted count of online mentions. It is computed as the sum of the quantity of online mentions (i.e. the number of mentions) weighted by the quality of online mentions (i.e. the source of mentions, such as Wikipedia) [18]. As of June 2023, the Altmetric score was computed based on the following 17 online channels with different weights [19] that were considered in this study:

- News (weight of 8)
- Blogs (weight of 5)
- Policy documents, patents, and Wikipedia (weight of 3)
- Post-publication peer review (on Publons or PubPeer), Weibo (traced until 2015), Google + (traced until 2019), F1000, and Syllabi (weight of 1)
- LinkedIn (traced until 2014, weight of 0.5)
- X/Twitter (posts and reposts), Facebook, Reddit, Pinterest (data until 2013), Q&A (Stack Exchange), and YouTube videos (weight of 0.25)

To prevent score inflation, the computation algorithm for the Altmetric score considers channel types and their weights as well as other factors, such as duplicate posts in the same channels [19]. In general, only the first mention in a specific online channel counts towards the Altmetric score (e.g. if a Cochrane review is mentioned in multiple articles in the same news channel, then only the first mention contributes to the Altmetric score for that Cochrane review) [19, 20].

The academic online channels, including Mendeley readers and Dimension and Web of Science citations, were also traced by Altmetric data in June 2023, but not included in the computation of the Altmetric score [19] and, thus, not considered in this study.

To reduce any bias due to updates in Altmetric data that occur daily at midnight West European time, Altmetric data for all reviews were extracted on a single day (29 June 2023) during business hours in Germany. One researcher (M. K.) made a screenshot of Altmetric data for each Cochrane review and collated all screenshots into one document. A team assistant manually entered all Altmetric data into the data extraction sheet in Excel. To reduce bias in data entry, one researcher (M. K.) checked the Altmetric data in 10% of reviews. Since no errors were detected, no further checks were performed.

### Data synthesis

The data were synthesised using descriptive statistics (e.g. absolute and relative frequencies or measures of central tendency, including mean and standard deviation) in Excel and IBM-SPSS24. Depending on data availability and type, we planned to investigate if dissemination (i.e. Altmetric scores) is associated with any review characteristics (e.g. publication year) or evidence quality characteristics (e.g. certainty of evidence ratings) using a univariate linear or binary logistic regression analysis in IBM-SPSS24.

## Results

### Sample size

**Fig. 1:**
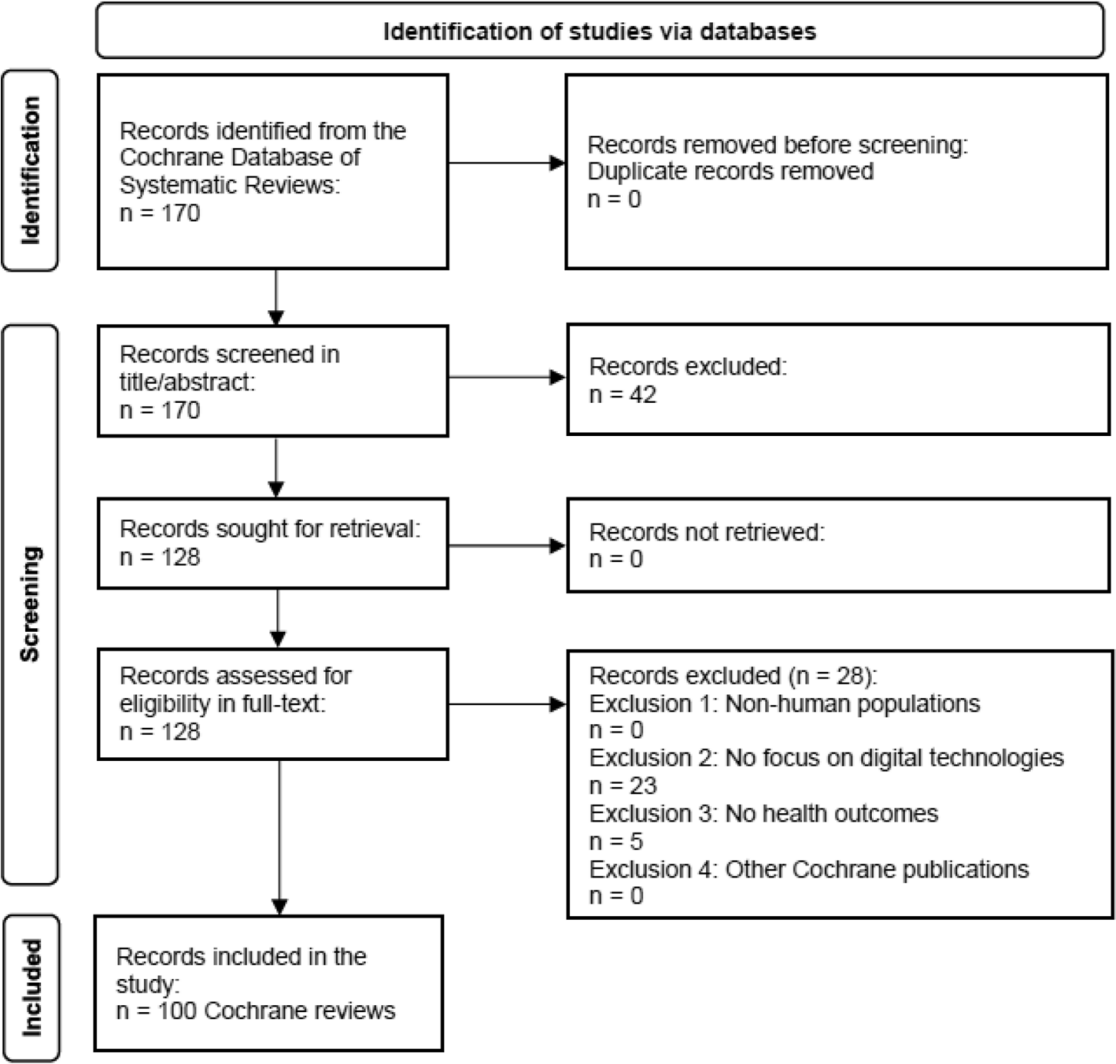
Study selection (PRISMA 2020 flowchart)

This study includes data from 100 Cochrane reviews that met the eligibility criteria out of 170 records identified in the search of Cochrane Database of Systematic Reviews (Fig. 1). A list of 100 included reviews and excluded reviews with individual reasons for exclusion is reported in Additional file 3. All data extracted from the 100 reviews are reported in Additional files 4 and 5, and data synthesis is reported in Additional file 6.

### Review characteristics

#### Bibliographic characteristics

The included 100 Cochrane reviews were published between 2005 and 2023. The reviews included systematic reviews (*n* = 97), rapid reviews (*n* = 2), and an overview of reviews (*n* = 1). The reviews originated from Europe (*n* = 61), Australia (*n* = 21), North America (*n* = 13), Asia (*n* = 3), or Africa (*n* = 2).

#### PICO characteristics

**Fig. 2:**
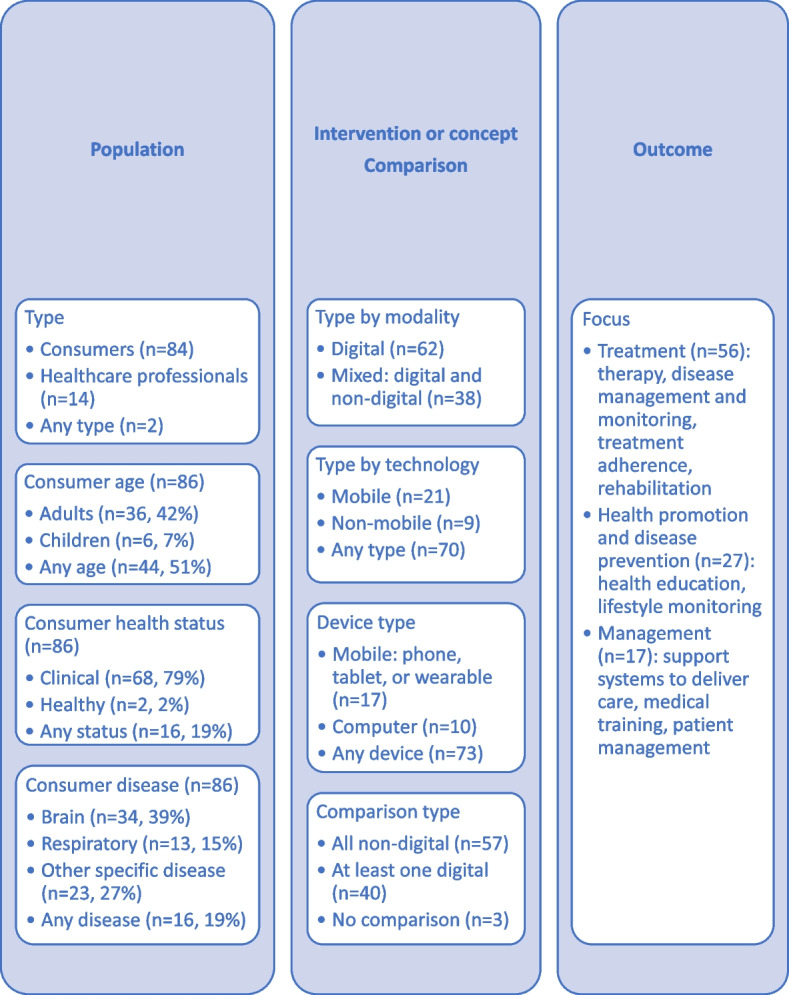
PICO characteristics of the included Cochrane reviews

The PICO characteristics of the included 100 Cochrane reviews are summarised in Fig. 2.

The reviews focused predominantly on consumers (i.e. patients, clients, or carers; *n* = 86), people of any age (i.e. adults or children; *n* = 44), and clinical populations (*n* = 68). Disease focus in most reviews (*n* = 63) was on brain, respiratory, or any diseases, while other specific diseases (e.g. cardiovascular) were addressed in less than 10 reviews each.

All reviews addressed interventions or concepts supported by digital health technologies. Depending on modality, most reviews (*n* = 62) included only digital interventions or concepts (e.g. interventions delivered via mobile phones in all primary studies). Depending on digital technology type, most reviews (*n* = 70) included any mobile or nonmobile interventions or concepts (i.e. those delivered using mobile or nonmobile devices). The digital devices used to deliver the interventions or concepts were any devices (e.g. mobile phones or computers) in most reviews (*n* = 73). The interaction between users and digital technologies occurred via emails, text messages, websites, apps, social media, interactive voice response systems, video calls, virtual reality, or electronic patient records. Most reviews (*n* = 57) compared the outcomes of digital interventions or concepts with non-digital comparison conditions.

All reviews addressed any outcomes in the healthcare context. Most reviews focused on disease treatment (i.e. therapy, adherence, disease management and monitoring, or rehabilitation; *n* = 56) and management of care delivery (i.e. support systems for care delivery, medical training, or patient management via electronic patient records; *n* = 17), while some (*n* = 27) also focused on public health (i.e. health promotion and disease prevention). Specifically, the 27 public health reviews addressed health education (e.g. in the context of reproductive and sexual health or vaccination uptake) and lifestyle monitoring (e.g. secondary and tertiary prevention for people with chronic diseases, management of weight or stress, prevention of social isolation or cognitive decline, or substance use reduction).

#### Evidence quality

All reviews included 1–132 studies (50 reviews included 1–10 studies, and further 50 reviews included 11–132 studies), and meta-analysis was performed in 69 reviews. The certainty of evidence was rated for a total of 767 quantitative outcomes assessed in 87 reviews. Among the 87 reviews, the certainty of evidence for at least one outcome was high in 10 (11%) reviews, moderate in 45 (52%) reviews, low in 71 (82%) reviews, or very low in 58 (67%) reviews. Overall, 46 (53%) reviews rated the certainty of evidence for at least one outcome as high or moderate, and 41 (47%) reviews did not rate any outcomes as high or moderate. Among the 767 quantitative outcomes, the certainty of evidence was high for 22 (3%) outcomes, moderate for 170 (22%) outcomes, low for 298 (39%) outcomes, and very low for 277 (36%) outcomes.

### Dissemination strategies

In agreement with the Cochrane guidelines, all reviews had a text-based plain language summary (PLS) that was available in 3–14 languages, including English. Most common translation languages were Spanish (*n* = 100), French (*n* = 88), and Arabic (*n* = 72). According to review information on the Cochrane website, 60 reviews were cited in 1–18 clinical guidelines.

**Fig. 3:**
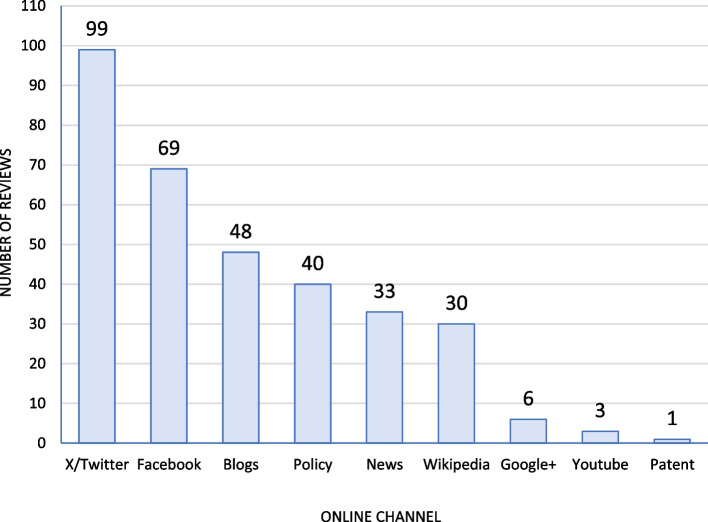
Dissemination (i.e. at least one mention) of Cochrane reviews by online channel

According to the Altmetric data, the included Cochrane reviews were disseminated (i.e. mentioned online at least once) in 9/17 online channels that are used to compute the Altmetric score (Fig. 3). These included predominantly social media (X/Twitter and Facebook), while less than 50% of reviews were disseminated via blogs, policy sources, news, Wikipedia, Google + , YouTube, or patents. The Cochrane reviews were not mentioned in 8/17 online channels, including (post-publication) peer reviews, Weibo, Syllabi, F1000, LinkedIn, Reddit, Q&A, and Pinterest.

**Fig. 4:**
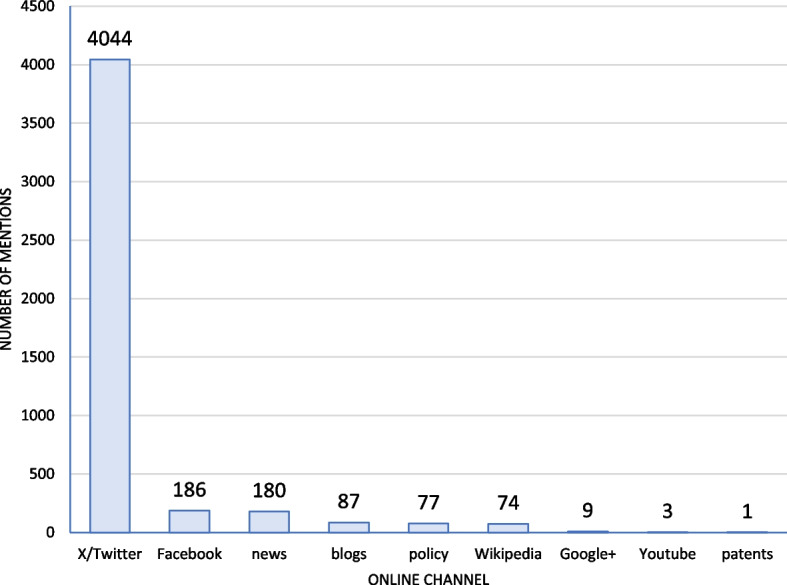
Number of mentions of Cochrane reviews by online channel

The included Cochrane reviews were mentioned 4661 times in 9/17 online channels (Fig. 4). Most traced mentions were found in the social media (X/Twitter and Facebook) and news outlets. Each Cochrane review was mentioned 1–271 times (50 reviews were mentioned 1–33 times, and further 50 were mentioned 34–271 times).

The Altmetric score for all reviews ranged between 1 and 553 (mean = 48, standard deviation = 67; 1–28 in 50 reviews and 29–553 in further 50 reviews). Most reviews (*n* = 97) had Altmetric scores between 1 and 150, and *n* = 3 reviews had Altmetric scores of more than 200 (i.e. 207 [21], 237 [22], and 553 [23]). These *n* = 3 reviews addressed topics relevant for the COVID-19 pandemic, including interventions to improve vaccination rates [23], digital technologies for contact tracing during epidemics [22], and interventions to reduce social isolation and loneliness among the elderly [21].

The online attention towards most reviews was high. Altmetric scores in 51 reviews were rated in up to the top 25% of all research outputs traced by Altmetric data (i.e. Altmetric scores of 1–6 in 10 reviews were rated below the top 25%, and Altmetric scores of 7–29 in 41 reviews were rated in the top 25%). Altmetric scores of 30–553 (in 49 reviews) were rated in the top 5% of all research outputs traced by Altmetric data.

### Association between dissemination and review characteristics

To investigate if dissemination (i.e. Altmetric scores) is associated with any review characteristics, we performed a binary logistic regression analysis with one dependent and five independent variables (Table 2).

**Table 2:** Association between dissemination (Altmetric scores) and review characteristics

Variables in binary logistic regression	Variable definition	Variable coding	Odds ratio [95% confidence interval]
Dependent	Dissemination	0 = lower Altmetric scores in < top 5% of all research outputs; 1 = higher Altmetric scores in top 5% of all research outputs	-
Independent: Bibliographic characteristics	Publication year	2005–2023	0.83 [0.70–0.99]*
	PLS languages	3–14	1.28 [1.05–1.56]*
Independent: Evidence quality characteristics	Studies in review	0 = few (1–10 studies); 1 = many (11–132 studies)	1.69 [0.59–4.79]
	Meta-analysis performed in review	0 = no; 1 = yes	1.24 [0.40–3.88]
	Certainty of evidence	0 = low (review with no outcomes rated as strong or moderate); 1 = high (review with at least one outcome rated as strong or moderate)	1.56 [0.60–4.06]

Note: Based on visual inspection, variables with a skewed distribution of scores were dichotomised using median values. Other review characteristics were not included as independent variables due to the potential overlap in categories (e.g. ‘any digital interventions’ partially overlapped with ‘mobile interventions’ or ‘non-mobile interventions’). A total of *n* = 87 reviews were included in the binary logistic regression analysis because certainty of evidence ratings was not reported in 13 reviews. Reference categories were coded as 0. Overall model details are as follows: chi-square = 12.8, *df* = 5, *p* = 0.025. *p < 0.05

Dissemination (i.e. higher Altmetric scores) was associated with bibliographic review characteristics (i.e. older review publication year and PLS available in more languages). Dissemination was not associated with evidence quality (i.e. certainty of evidence, studies in review, or meta-analysis performed in review; Table 2). The same pattern of results was obtained in a sensitivity analysis when the three reviews with the highest Altmetric scores were removed from the binary logistic regression analysis (Additional file 6).

### Dissemination of this study

In addition to this academic publication, we planned to disseminate the results of this study via a conference poster at a scientific meeting [24] and PLS in English and in German (Additional file 7).

## Discussion

### Summary of main findings

This cross-sectional study based on 100 Cochrane reviews on digital health technologies shows that such reviews (1) addressed different types of digital technologies focusing mainly on disease treatment or management in adult clinical populations and (2) were widely disseminated via nonacademic online channels. The online attention towards 90 reviews was high based on Altmetric scores in the top 25% or 5% of all research outputs traced by the Altmetric data. Dissemination was associated with bibliographic review characteristics (earlier publication year and more PLS translations), but not with evidence quality.

### Dissemination

Our study shows that Cochrane reviews on digital health technologies were predominantly disseminated via the social media (X/Twitter or Facebook). Thus, Cochrane reviews might be used as important source of health information for users of such media. Interestingly, dissemination was not associated with evidence quality but rather with relevance of review topic (i.e. the highest Altmetric scores were recorded for review related to COVID-19 topics) and accessibility of review (i.e. more years since publication and with PLS in more languages). Similar trends are also observed in citations of scientific articles that reflect the scientific impact and relevance of article topics but less so research quality [25]. Previous studies using Altmetric data suggest that higher visibility in social media is associated with other characteristics of scientific articles not considered in this study, including high journal impact factors, published open access, and having informative titles [26–28]. Various measures could be used to improve dissemination of Cochrane reviews based on their evidence quality rather than their mere presence online in various languages. For example, improvements in science communication may help academic authors to clearly communicate their scientific findings [9]. This may enhance the lay understanding of PLS for nonacademic stakeholders and thus facilitate their decision whether or not to disseminate a specific content. In particular, the evidence limitations, such as very low and low certainty of evidence, need to be adequately explained in the PLS. Furthermore, although not investigated in this study, more focus on enhancing health literacy and digital health literacy is needed to improve the understanding of scientific content for any stakeholders or population groups [29].

Poor understanding of scientific content and restrictions in access to scientific evidence may contribute to the delay in dissemination of findings from newer reviews and consequently translating research evidence into clinical practice (the so-called research to practice gap) [30]. Such time lags in the translation process are prevalent in diverse fields of healthcare, and it can take up to several years between publication and being implemented or mentioned in clinical guidelines [31, 32]. Cochrane attempted to reduce the research to practice gap for COVID-19 research by publishing relevant reviews in a timely fashion (e.g. as rapid reviews) and by establishing a register of COVID-19 publications so that they could be located online faster [33]. These measures together with the global interest in COVID-19 may have contributed to the finding that COVID-19 reviews had the highest Altmetric scores in this study indicating that they were disseminated online via various channels despite their young age (recent publication date) and potentially reaching the relevant stakeholders. Future research may examine in more detail the dissemination approaches used by Cochrane for their COVID-19 reviews to find out if similar approaches could also improve the online attention towards other Cochrane or non-Cochrane reviews.

According to Altmetric data, we show that there is high online attention to Cochrane reviews on digital health technologies. This result is in line with a high academic impact of Cochrane reviews. Specifically, Cochrane reviews in public health were cited on average 240% more than other papers in this field [8]. In contrast to academic impact, Altmetric data can be used as a proxy of online interest in academic publications. In the current study, X/Twitter was the platform where the included Cochrane reviews were mentioned most by far. However, such data have various limitations because it is unclear who does the dissemination (i.e. review authors or anyone with the internet access), what are the motivations to disseminate the scientific content, and how the receivers of online mentions interact with and use the information. Furthermore, the Altmetric score only counts the interactions on different online media platforms, but it cannot distinguish between positive and critical attention [34–36]. Altmetric data may also be more prone to manipulation than traditional bibliometrics [35]. For example, Facebook mentions can be purchased [37], and it is unclear if mentions on social media platforms only passively exist or are actually read [35]. However, the strength of the Altmetric data is that the attention metrics for an academic publication are immediately traced and available within hours of publication [35, 36]. In contrast, scientific citation metrics, such as citation counts or journal impact factors, are typically available several years after publication [35, 36].

Altmetric score relies on a selection of online channels, some of which (e.g. Google +) are no longer traced. Potentially, there is a need to capture other online channels for dissemination, such as TikTok videos or Instagram clips. Online attention could also be increased when authors of academic publications use more knowledge translation strategies that is in turn associated with higher impact of such publications on end users via health policy and practice [38]. Especially, social media dissemination channels could be used to reach out to knowledge users [11, 12]. More visibility and potentially higher Altmetric scores could result from using hashtags for social media posts that enhance searching and finding of content and including a unique identifier of the study (i.e. a link to the Cochrane review and not only its abstract) in social media posts or blogs in the main text [39].

### Future priorities for research on digital health technologies

Based on the content of the Cochrane reviews included in this study, we provide some recommendations for future research on digital health technologies.

1. *Focus on specific digital health technology types*: Digital technologies encompass a wide range of tools and systems, making it challenging to create a comprehensive and universally applicable definition [6]. Most included Cochrane reviews adopted a broad perspective by focusing on any digital health technologies and thus consequently compared ‘apples and oranges’. The evolving landscape of digital technologies shows that more targeted evaluations of specific technology types, such as smartphone apps or websites accessed via computers for specific health purposes, such as physical activity promotion, are needed [6]. Thus, a future research priority is to evaluate specific digital health technology types to establish if their use contributes to any health benefits and if such benefits have any clinical relevance.
2. *Focus on the needs of diverse populations*: Most included reviews included broad target populations, such as healthcare consumers (i.e. patients or carers) and people of any age. Healthcare consumers may have different needs and acceptance of digital health offers than healthcare professionals [40–42], who were less often studied. Furthermore, different age cohorts may have distinct needs, preferences, and responses to digital health technologies due to their digital experience. While younger age cohorts are likely to be digitised (and thus considered as ‘digital natives’), older age cohorts have varying levels of digital experience that could affect their uptake and use of digital health technologies [43, 44]. Thus, a future research priority is to assess the needs of diverse populations regarding digital health technologies.
3. *Focus expanding beyond healthcare to public health*: Most included Cochrane reviews focused on healthcare for brain or respiratory diseases, while only about a quarter addressed issues central to public health, including health promotion and disease prevention. Digital technologies have already been extensively used in the public-health response to the COVID-19 pandemic [45]. Some features of digital health technologies could be used for preventing or managing of other common diseases, such as cardiovascular diseases, which despite being the leading causes of death worldwide [46] were surprisingly addressed in very few reviews. For example, wearable technologies with feedback and nudging functions could be used to encourage physical activity and reduce sedentary behaviour [47], and smartphone apps could be used to provide recommendations on nutrition [48]. Thus, a future research priority is to focus on the role of digital technologies in health promotion and disease prevention.

### Limitations

There were several limitations in this study. First, we did not investigate who disseminated the Cochrane reviews and why. Although review authors can disseminate their own Cochrane reviews, any planned dissemination is rarely described in the text of such reviews [9]. Dissemination via channels traced by Altmetric data can be done by anyone with Internet access. Future studies could investigate the motivations for disseminating the Cochrane reviews. Second, it is unclear in what specific (online) news outlets the Cochrane reviews were disseminated. Unlike listing the names of various online media channels (e.g. Facebook or Wikipedia), Altmetric data do not specify which (online) news outlets are traced despite that news mentions contribute the highest weight to the Altmetric score. Third, the associations between dissemination (i.e. Altmetric scores) and review characteristics were only weak, possibly because most Cochrane reviews had relatively high Altmetric scores (in the top 25% or 5% of all research outputs traced by Altmetric data). Future studies could investigate such associations in non-Cochrane reviews with more variable Altmetric scores and compare Altmetric data between Cochrane and non-Cochrane reviews. Fourth, the lack of association between dissemination and certainty of evidence rating could be due to a high heterogeneity of outcomes and digital technologies in the included Cochrane reviews. Certainty of evidence ratings in reviews (Cochrane and non-Cochrane) with the same outcome and the same digital technology could be used in future analyses. Fifth, despite a large sample size (100 reviews), the results of this study might not be generalisable to non-Cochrane reviews, Cochrane reviews in other fields than digital health technologies, and other methods of assessing dissemination than Altmetric scores.

## Conclusions

Online attention towards Cochrane reviews on digital health technologies is high. In particular, social media act as nonacademic dissemination channels for such Cochrane reviews. Dissemination is higher for older reviews and reviews with more PLS translations. Measures are required to improve dissemination of Cochrane reviews based on evidence quality. Future research is needed (1) to evaluate specific digital health technology types, (2) to assess the needs of diverse populations regarding digital health technologies, and (3) to focus on the role of digital technologies in health promotion and disease prevention.

### Supplementary Information

Additional file 1. STROBE Checklist

Additional file 2. Search strategy.

Additional file 3. List of included and excluded studies.

Additional file 4. Data file.

Additional file 5. Altmetric data.

Additional file 6. Data synthesis.

Additional file 7. Plain Language Summary.

## Data Availability

The dataset supporting the conclusions of this article is included within the article and its additional file.

## Abbreviations

PICO: Population, Intervention, Comparison, Outcome
PLS: Plain language summary
PRISMA: Preferred Reporting Items for Systematic Reviews and Meta-Analyses
STROBE: Strengthening the Reporting of Observational Studies in Epidemiology
