# Supplementary material for: Online dissemination of Cochrane reviews on digital health technologies: a cross-sectional study
Source: Syst Rev. 2024 May 15;13:133. doi: 10.1186/s13643-024-02557-6 (PMC11095012; doi:10.1186/s13643-024-02557-6)
Supplement: Supplementary file 3 — Additional file 3. List of included and excluded studies. [file 13643_2024_2557_MOESM3_ESM.docx]

**Additional file 3**

## List of included and excluded studies (Cochrane reviews).

| **Study status** | **Studies, n** | **Citation** |
| --- | --- | --- |
| **Studies identified in electronic search** | **170** |  |
| **Included** | **100** | **[1-100]** |
| **Excluded studies after title and abstract screening** | **42** | **[101-142]** |
| Exclusion 1: No population | 8 |  |
| - no studies included | 6 | [101-106] |
| - withdrawal from publication | 2 | [107, 108] |
| Exclusion 2: No application of digital technologies | 32 |  |
| - no digital technology | 11 | [109-119] |
| - operative/diagnostic methods | 17 | [120-136] |
| - focus on test accuracy | 4 | [137-140] |
| Exclusion 3: No health outcome | 2 | [141, 142] |
| **Excluded studies after full-text screening** | **28** | **[143-170]** |
| Exclusion 2: No application of digital technologies | 23 |  |
| - operative/diagnostic methods | 1 | [143] |
| - telephone | 15 | [144-158] |
| - analog games | 2 | [159, 160] |
| - video | 3 | [161-163] |
| - other reason | 2 | [164, 165] |
| Exclusion 3: No health outcome | 5 |  |
| - no health outcome | 2 | [166, 167] |
| - no Altmetric score | 3 | [168-170] |

1. Aali G, Kariotis T, Shokraneh F. Avatar Therapy for people with schizophrenia or related disorders. Cochrane Database of Systematic Reviews. 2020;(5). doi: 10.1002/14651858.CD011898.pub2.

2. Adler AJ, Martin N, Mariani J, Tajer CD, Owolabi OO, Free C, Serrano NC, Casas JP, Perel P. Mobile phone text messaging to improve medication adherence in secondary prevention of cardiovascular disease. Cochrane Database of Systematic Reviews. 2017;(4). doi: 10.1002/14651858.CD011851.pub2.

3. Agarwal S, Glenton C, Henschke N, Tamrat T, Bergman H, Fønhus MS, Mehl GL, Lewin S. Tracking health commodity inventory and notifying stock levels via mobile devices: a mixed methods systematic review. Cochrane Database of Systematic Reviews. 2020;(10). doi: 10.1002/14651858.CD012907.pub2.

4. Agarwal S, Glenton C, Tamrat T, Henschke N, Maayan N, Fønhus MS, Mehl GL, Lewin S. Decision‐support tools via mobile devices to improve quality of care in primary healthcare settings. Cochrane Database of Systematic Reviews. 2021;(7). doi: 10.1002/14651858.CD012944.pub2.

5. Allida S, Du H, Xu X, Prichard R, Chang S, Hickman LD, Davidson PM, Inglis SC. mHealth education interventions in heart failure. Cochrane Database of Systematic Reviews. 2020;(7). doi: 10.1002/14651858.CD011845.pub2.

6. Ames HMR, Glenton C, Lewin S, Tamrat T, Akama E, Leon N. Clients’ perceptions and experiences of targeted digital communication accessible via mobile devices for reproductive, maternal, newborn, child, and adolescent health: a qualitative evidence synthesis. Cochrane Database of Systematic Reviews. 2019;(10). doi: 10.1002/14651858.CD013447.

7. Ammenwerth E, Neyer S, Hörbst A, Mueller G, Siebert U, Schnell-Inderst P. Adult patient access to electronic health records. Cochrane Database of Systematic Reviews. 2021;(2). doi: 10.1002/14651858.CD012707.pub2.

8. Anglemyer A, Moore THM, Parker L, Chambers T, Grady A, Chiu K, Parry M, Wilczynska M, Flemyng E, Bero L. Digital contact tracing technologies in epidemics: a rapid review. Cochrane Database of Systematic Reviews. 2020;(8). doi: 10.1002/14651858.CD013699.

9. Atherton H, Sawmynaden P, Sheikh A, Majeed A, Car J. Email for clinical communication between patients/caregivers and healthcare professionals. Cochrane Database of Systematic Reviews. 2012;(11). doi: 10.1002/14651858.CD007978.pub2.

10. Bahar‐Fuchs A, Martyr A, Goh AMY, Sabates J, Clare L. Cognitive training for people with mild to moderate dementia. Cochrane Database of Systematic Reviews. 2019;(3). doi: 10.1002/14651858.CD013069.pub2.

11. Bailey JV, Murray E, Rait G, Mercer CH, Morris RW, Peacock R, Cassell J, Nazareth I. Interactive computer‐based interventions for sexual health promotion. Cochrane Database of Systematic Reviews. 2010;(9). doi: 10.1002/14651858.CD006483.pub2.

12. Boyle R, Solberg L, Fiore M. Use of electronic health records to support smoking cessation. Cochrane Database of Systematic Reviews. 2014;(12). doi: 10.1002/14651858.CD008743.pub3.

13. Burge AT, Cox NS, Abramson MJ, Holland AE. Interventions for promoting physical activity in people with chronic obstructive pulmonary disease (COPD). Cochrane Database of Systematic Reviews. 2020;(4). doi: 10.1002/14651858.CD012626.pub2.

14. Car J, Lang B, Colledge A, Ung C, Majeed A. Interventions for enhancing consumers' online health literacy. Cochrane Database of Systematic Reviews. 2011;(6). doi: 10.1002/14651858.CD007092.pub2.

15. Chan A, De Simoni A, Wileman V, Holliday L, Newby CJ, Chisari C, Ali S, Zhu N, Padakanti P, Pinprachanan V *et al*. Digital interventions to improve adherence to maintenance medication in asthma. Cochrane Database of Systematic Reviews. 2022;(6). doi: 10.1002/14651858.CD013030.pub2.

16. Cox NS, Dal Corso S, Hansen H, McDonald CF, Hill CJ, Zanaboni P, Alison JA, O'Halloran P, Macdonald H, Holland AE. Telerehabilitation for chronic respiratory disease. Cochrane Database of Systematic Reviews. 2021;(1). doi: 10.1002/14651858.CD013040.pub2.

17. Datta SS, Daruvala R, Kumar A. Psychological interventions for psychosis in adolescents. Cochrane Database of Systematic Reviews. 2020;(7). doi: 10.1002/14651858.CD009533.pub2.

18. de Jongh T, Gurol‐Urganci I, Vodopivec‐Jamsek V, Car J, Atun R. Mobile phone messaging for facilitating self‐management of long‐term illnesses. Cochrane Database of Systematic Reviews. 2012;(12). doi: 10.1002/14651858.CD007459.pub2.

19. Dennett EJ, Janjua S, Stovold E, Harrison SL, McDonnell MJ, Holland AE. Tailored or adapted interventions for adults with chronic obstructive pulmonary disease and at least one other long‐term condition: a mixed methods review. Cochrane Database of Systematic Reviews. 2021;(7). doi: 10.1002/14651858.CD013384.pub2.

20. Devi R, Singh SJ, Powell J, Fulton EA, Igbinedion E, Rees K. Internet‐based interventions for the secondary prevention of coronary heart disease. Cochrane Database of Systematic Reviews. 2015;(12). doi: 10.1002/14651858.CD009386.pub2.

21. Eccleston C, Fisher E, Craig L, Duggan GB, Rosser BA, Keogh E. Psychological therapies (Internet‐delivered) for the management of chronic pain in adults. Cochrane Database of Systematic Reviews. 2014;(2). doi: 10.1002/14651858.CD010152.pub2.

22. Eccleston C, Fisher E, Thomas KH, Hearn L, Derry S, Stannard C, Knaggs R, Moore RA. Interventions for the reduction of prescribed opioid use in chronic non‐cancer pain. Cochrane Database of Systematic Reviews. 2017;(11). doi: 10.1002/14651858.CD010323.pub3.

23. Fiander M, McGowan J, Grad R, Pluye P, Hannes K, Labrecque M, Roberts NW, Salzwedel DM, Welch V, Tugwell P. Interventions to increase the use of electronic health information by healthcare practitioners to improve clinical practice and patient outcomes. Cochrane Database of Systematic Reviews. 2015;(3). doi: 10.1002/14651858.CD004749.pub3.

24. Fisher E, Law E, Dudeney J, Eccleston C, Palermo TM. Psychological therapies (remotely delivered) for the management of chronic and recurrent pain in children and adolescents. Cochrane Database of Systematic Reviews. 2019;(4). doi: 10.1002/14651858.CD011118.pub3.

25. Fleming PS, Strydom H, Katsaros C, MacDonald LCI, Curatolo M, Fudalej P, Pandis N. Non‐pharmacological interventions for alleviating pain during orthodontic treatment. Cochrane Database of Systematic Reviews. 2016;(12). doi: 10.1002/14651858.CD010263.pub2.

26. Flodgren G, Rachas A, Farmer AJ, Inzitari M, Shepperd S. Interactive telemedicine: effects on professional practice and health care outcomes. Cochrane Database of Systematic Reviews. 2015;(9). doi: 10.1002/14651858.CD002098.pub2.

27. Foster C, Richards J, Thorogood M, Hillsdon M. Remote and web 2.0 interventions for promoting physical activity. Cochrane Database of Systematic Reviews. 2013;(9). doi: 10.1002/14651858.CD010395.pub2.

28. Gates NJ, Rutjes AWS, Di Nisio M, Karim S, Chong LY, March E, Martínez G, Vernooij RWM. Computerised cognitive training for maintaining cognitive function in cognitively healthy people in midlife. Cochrane Database of Systematic Reviews. 2019;(3). doi: 10.1002/14651858.CD012278.pub2.

29. Gates NJ, Rutjes AWS, Di Nisio M, Karim S, Chong LY, March E, Martínez G, Vernooij RWM. Computerised cognitive training for 12 or more weeks for maintaining cognitive function in cognitively healthy people in late life. Cochrane Database of Systematic Reviews. 2020;(2). doi: 10.1002/14651858.CD012277.pub3.

30. Gates NJ, Vernooij RWM, Di Nisio M, Karim S, March E, Martínez G, Rutjes AWS. Computerised cognitive training for preventing dementia in people with mild cognitive impairment. Cochrane Database of Systematic Reviews. 2019;(3). doi: 10.1002/14651858.CD012279.pub2.

31. Gavine A, Shinwell SC, Buchanan P, Farre A, Wade A, Lynn F, Marshall J, Cumming SE, Dare S, McFadden A. Support for healthy breastfeeding mothers with healthy term babies. Cochrane Database of Systematic Reviews. 2022;(10). doi: 10.1002/14651858.CD001141.pub6.

32. Gillaizeau F, Chan E, Trinquart L, Colombet I, Walton RT, Rège‐Walther M, Burnand B, Durieux P. Computerized advice on drug dosage to improve prescribing practice. Cochrane Database of Systematic Reviews. 2013;(11). doi: 10.1002/14651858.CD002894.pub3.

33. Gonçalves-Bradley DC, J Maria AR, Ricci-Cabello I, Villanueva G, Fønhus MS, Glenton C, Lewin S, Henschke N, Buckley BS, Mehl GL *et al*. Mobile technologies to support healthcare provider to healthcare provider communication and management of care. Cochrane Database of Systematic Reviews. 2020;(8). doi: 10.1002/14651858.CD012927.pub2.

34. González-Fraile E, Ballesteros J, Rueda JR, Santos-Zorrozúa B, Solà I, McCleery J. Remotely delivered information, training and support for informal caregivers of people with dementia. Cochrane Database of Systematic Reviews. 2021;(1). doi: 10.1002/14651858.CD006440.pub3.

35. Gordon M, Sinopoulou V, Lakunina S, Gjuladin-Hellon T, Bracewell K, Akobeng AK. Remote care through telehealth for people with inflammatory bowel disease. Cochrane Database of Systematic Reviews. 2023;(5). doi: 10.1002/14651858.CD014821.pub2.

36. Goyder C, Atherton H, Car M, Heneghan CJ, Car J. Email for clinical communication between healthcare professionals. Cochrane Database of Systematic Reviews. 2015;(2). doi: 10.1002/14651858.CD007979.pub3.

37. Gurol‐Urganci I, de Jongh T, Vodopivec‐Jamsek V, Atun R, Car J. Mobile phone messaging reminders for attendance at healthcare appointments. Cochrane Database of Systematic Reviews. 2013;(12). doi: 10.1002/14651858.CD007458.pub3.

38. Gurol‐Urganci I, de Jongh T, Vodopivec‐Jamsek V, Car J, Atun R. Mobile phone messaging for communicating results of medical investigations. Cochrane Database of Systematic Reviews. 2012;(6). doi: 10.1002/14651858.CD007456.pub2.

39. Gurusamy KS, Vaughan J, Davidson BR. Formal education of patients about to undergo laparoscopic cholecystectomy. Cochrane Database of Systematic Reviews. 2014;(2). doi: 10.1002/14651858.CD009933.pub2.

40. Horvath T, Azman H, Kennedy GE, Rutherford GW. Mobile phone text messaging for promoting adherence to antiretroviral therapy in patients with HIV infection. Cochrane Database of Systematic Reviews. 2012;(3). doi: 10.1002/14651858.CD009756.

41. Inglis SC, Clark RA, Dierckx R, Prieto‐Merino D, Cleland JGF. Structured telephone support or non‐invasive telemonitoring for patients with heart failure. Cochrane Database of Systematic Reviews. 2015;(10). doi: 10.1002/14651858.CD007228.pub3.

42. Jacobson Vann JC, Jacobson RM, Coyne‐Beasley T, Asafu‐Adjei JK, Szilagyi PG. Patient reminder and recall interventions to improve immunization rates. Cochrane Database of Systematic Reviews. 2018;(1). doi: 10.1002/14651858.CD003941.pub3.

43. Janjua S, Banchoff E, Threapleton CJD, Prigmore S, Fletcher J, Disler RT. Digital interventions for the management of chronic obstructive pulmonary disease. Cochrane Database of Systematic Reviews. 2021;(4). doi: 10.1002/14651858.CD013246.pub2.

44. Janjua S, Carter D, Threapleton CJD, Prigmore S, Disler RT. Telehealth interventions: remote monitoring and consultations for people with chronic obstructive pulmonary disease (COPD). Cochrane Database of Systematic Reviews. 2021;(7). doi: 10.1002/14651858.CD013196.pub2.

45. Jawad A, Jawad I, Alwan NA. Interventions using social networking sites to promote contraception in women of reproductive age. Cochrane Database of Systematic Reviews. 2019;(3). doi: 10.1002/14651858.CD012521.pub2.

46. Kaner EFS, Beyer FR, Garnett C, Crane D, Brown J, Muirhead C, Redmore J, O'Donnell A, Newham JJ, de Vocht F *et al*. Personalised digital interventions for reducing hazardous and harmful alcohol consumption in community‐dwelling populations. Cochrane Database of Systematic Reviews. 2017;(9). doi: 10.1002/14651858.CD011479.pub2.

47. Kauppi K, Välimäki M, Hätönen HM, Kuosmanen LM, Warwick‐Smith K, Adams CE. Information and communication technology based prompting for treatment compliance for people with serious mental illness. Cochrane Database of Systematic Reviews. 2014;(6). doi: 10.1002/14651858.CD009960.pub2.

48. Kew KM, Cates CJ. Home telemonitoring and remote feedback between clinic visits for asthma. Cochrane Database of Systematic Reviews. 2016;(8). doi: 10.1002/14651858.CD011714.pub2.

49. Kew KM, Cates CJ. Remote versus face‐to‐face check‐ups for asthma. Cochrane Database of Systematic Reviews. 2016;(4). doi: 10.1002/14651858.CD011715.pub2.

50. Khan F, Amatya B, Kesselring J, Galea M. Telerehabilitation for persons with multiple sclerosis. Cochrane Database of Systematic Reviews. 2015;(4). doi: 10.1002/14651858.CD010508.pub2.

51. Kuster AT, Dalsbø TK, Luong Thanh BY, Agarwal A, Durand‐Moreau QV, Kirkehei I. Computer‐based versus in‐person interventions for preventing and reducing stress in workers. Cochrane Database of Systematic Reviews. 2017;(8). doi: 10.1002/14651858.CD011899.pub2.

52. Laver KE, Adey‐Wakeling Z, Crotty M, Lannin NA, George S, Sherrington C. Telerehabilitation services for stroke. Cochrane Database of Systematic Reviews. 2020;(1). doi: 10.1002/14651858.CD010255.pub3.

53. Laver KE, Lange B, George S, Deutsch JE, Saposnik G, Crotty M. Virtual reality for stroke rehabilitation. Cochrane Database of Systematic Reviews. 2017;(11). doi: 10.1002/14651858.CD008349.pub4.

54. Leon N, Balakrishna Y, Hohlfeld A, Odendaal WA, Schmidt BM, Zweigenthal V, Anstey Watkins J, Daniels K. Routine Health Information System (RHIS) improvements for strengthened health system management. Cochrane Database of Systematic Reviews. 2020;(8). doi: 10.1002/14651858.CD012012.pub2.

55. Linden M, Hawley C, Blackwood B, Evans J, Anderson V, O'Rourke C. Technological aids for the rehabilitation of memory and executive functioning in children and adolescents with acquired brain injury. Cochrane Database of Systematic Reviews. 2016;(7). doi: 10.1002/14651858.CD011020.pub2.

56. Lopez LM, Stockton LL, Chen M, Steiner MJ, Gallo MF. Behavioral interventions for improving dual‐method contraceptive use. Cochrane Database of Systematic Reviews. 2014;(3). doi: 10.1002/14651858.CD010915.pub2.

57. Lynch EA, Jones TM, Simpson DB, Fini NA, Kuys SS, Borschmann K, Kramer S, Johnson L, Callisaya ML, Mahendran N *et al*. Activity monitors for increasing physical activity in adult stroke survivors. Cochrane Database of Systematic Reviews. 2018;(7). doi: 10.1002/14651858.CD012543.pub2.

58. Malaguti C, Dal Corso S, Janjua S, Holland AE. Supervised maintenance programmes following pulmonary rehabilitation compared to usual care for chronic obstructive pulmonary disease. Cochrane Database of Systematic Reviews. 2021;(8). doi: 10.1002/14651858.CD013569.pub2.

59. Manyande A, Cyna AM, Yip P, Chooi C, Middleton P. Non‐pharmacological interventions for assisting the induction of anaesthesia in children. Cochrane Database of Systematic Reviews. 2015;(7). doi: 10.1002/14651858.CD006447.pub3.

60. Marcano Belisario JS, Huckvale K, Greenfield G, Car J, Gunn LH. Smartphone and tablet self management apps for asthma. Cochrane Database of Systematic Reviews. 2013;(11). doi: 10.1002/14651858.CD010013.pub2.

61. Mayo‐Wilson E, Montgomery P. Media‐delivered cognitive behavioural therapy and behavioural therapy (self‐help) for anxiety disorders in adults. Cochrane Database of Systematic Reviews. 2013;(9). doi: 10.1002/14651858.CD005330.pub4.

62. McCabe C, McCann M, Brady AM. Computer and mobile technology interventions for self‐management in chronic obstructive pulmonary disease. Cochrane Database of Systematic Reviews. 2017;(5). doi: 10.1002/14651858.CD011425.pub2.

63. McLean S, Chandler D, Nurmatov U, Liu JLY, Pagliari C, Car J, Sheikh A. Telehealthcare for asthma. Cochrane Database of Systematic Reviews. 2010;(10). doi: 10.1002/14651858.CD007717.pub2.

64. McLean S, Nurmatov U, Liu JLY, Pagliari C, Car J, Sheikh A. Telehealthcare for chronic obstructive pulmonary disease. Cochrane Database of Systematic Reviews. 2011;(7). doi: 10.1002/14651858.CD007718.pub2.

65. Murray E, Burns J, See Tai S, Lai R, Nazareth I. Interactive Health Communication Applications for people with chronic disease. Cochrane Database of Systematic Reviews. 2005;(4). doi: 10.1002/14651858.CD004274.pub4.

66. Murtagh EM, Murphy MH, Milton K, Roberts NW, O'Gorman CSM, Foster C. Interventions outside the workplace for reducing sedentary behaviour in adults under 60 years of age. Cochrane Database of Systematic Reviews. 2020;(7). doi: 10.1002/14651858.CD012554.pub2.

67. Nagendran M, Gurusamy KS, Aggarwal R, Loizidou M, Davidson BR. Virtual reality training for surgical trainees in laparoscopic surgery. Cochrane Database of Systematic Reviews. 2013;(8). doi: 10.1002/14651858.CD006575.pub3.

68. Noone C, McSharry J, Smalle M, Burns A, Dwan K, Devane D, Morrissey EC. Video calls for reducing social isolation and loneliness in older people: a rapid review. Cochrane Database of Systematic Reviews. 2020;(5). doi: 10.1002/14651858.CD013632.

69. Odendaal WA, Anstey Watkins J, Leon N, Goudge J, Griffiths F, Tomlinson M, Daniels K. Health workers’ perceptions and experiences of using mHealth technologies to deliver primary healthcare services: a qualitative evidence synthesis. Cochrane Database of Systematic Reviews. 2020;(3). doi: 10.1002/14651858.CD011942.pub2.

70. Olthuis JV, Watt MC, Bailey K, Hayden JA, Stewart SH. Therapist‐supported Internet cognitive behavioural therapy for anxiety disorders in adults. Cochrane Database of Systematic Reviews. 2016;(3). doi: 10.1002/14651858.CD011565.pub2.

71. Pal K, Eastwood SV, Michie S, Farmer AJ, Barnard ML, Peacock R, Wood B, Inniss JD, Murray E. Computer‐based diabetes self‐management interventions for adults with type 2 diabetes mellitus. Cochrane Database of Systematic Reviews. 2013;(3). doi: 10.1002/14651858.CD008776.pub2.

72. Palmer MJ, Henschke N, Bergman H, Villanueva G, Maayan N, Tamrat T, Mehl GL, Glenton C, Lewin S, Fønhus MS *et al*. Targeted client communication via mobile devices for improving maternal, neonatal, and child health. Cochrane Database of Systematic Reviews. 2020;(8). doi: 10.1002/14651858.CD013679.

73. Palmer MJ, Henschke N, Villanueva G, Maayan N, Bergman H, Glenton C, Lewin S, Fønhus MS, Tamrat T, Mehl GL *et al*. Targeted client communication via mobile devices for improving sexual and reproductive health. Cochrane Database of Systematic Reviews. 2020;(8). doi: 10.1002/14651858.CD013680.

74. Palmer MJ, Machiyama K, Woodd S, Gubijev A, Barnard S, Russell S, Perel P, Free C. Mobile phone‐based interventions for improving adherence to medication prescribed for the primary prevention of cardiovascular disease in adults. Cochrane Database of Systematic Reviews. 2021;(3). doi: 10.1002/14651858.CD012675.pub3.

75. Petkovic J, Duench S, Trawin J, Dewidar O, Pardo Pardo J, Simeon R, DesMeules M, Gagnon D, Hatcher Roberts J, Hossain A *et al*. Behavioural interventions delivered through interactive social media for health behaviour change, health outcomes, and health equity in the adult population. Cochrane Database of Systematic Reviews. 2021;(5). doi: 10.1002/14651858.CD012932.pub2.

76. Piromchai P, Avery A, Laopaiboon M, Kennedy G, O'Leary S. Virtual reality training for improving the skills needed for performing surgery of the ear, nose or throat. Cochrane Database of Systematic Reviews. 2015;(9). doi: 10.1002/14651858.CD010198.pub2.

77. Pollock A, Farmer SE, Brady MC, Langhorne P, Mead GE, Mehrholz J, van Wijck F. Interventions for improving upper limb function after stroke. Cochrane Database of Systematic Reviews. 2014;(11). doi: 10.1002/14651858.CD010820.pub2.

78. Posadzki P, Mastellos N, Ryan R, Gunn LH, Felix LM, Pappas Y, Gagnon MP, Julious SA, Xiang L, Oldenburg B *et al*. Automated telephone communication systems for preventive healthcare and management of long‐term conditions. Cochrane Database of Systematic Reviews. 2016;(12). doi: 10.1002/14651858.CD009921.pub2.

79. Raman P, Shepherd E, Dowswell T, Middleton P, Crowther CA. Different methods and settings for glucose monitoring for gestational diabetes during pregnancy. Cochrane Database of Systematic Reviews. 2017;(10). doi: 10.1002/14651858.CD011069.pub2.

80. Reeves S, Pelone F, Harrison R, Goldman J, Zwarenstein M. Interprofessional collaboration to improve professional practice and healthcare outcomes. Cochrane Database of Systematic Reviews. 2017;(6). doi: 10.1002/14651858.CD000072.pub3.

81. Roberts MT, Lloyd J, Välimäki M, Ho GWK, Freemantle M, Békefi AZ. Video games for people with schizophrenia. Cochrane Database of Systematic Reviews. 2021;(2). doi: 10.1002/14651858.CD012844.pub2.

82. Sawmynaden P, Atherton H, Majeed A, Car J. Email for the provision of information on disease prevention and health promotion. Cochrane Database of Systematic Reviews. 2012;(11). doi: 10.1002/14651858.CD007982.pub2.

83. Shojania KG, Jennings A, Ramsay CR, Grimshaw JM, Kwan JL, Lo L. The effects of on‐screen, point of care computer reminders on processes and outcomes of care. Cochrane Database of Systematic Reviews. 2009;(3). doi: 10.1002/14651858.CD001096.pub2.

84. Simon N, Robertson L, Lewis C, Roberts NP, Bethell A, Dawson S, Bisson JI. Internet‐based cognitive and behavioural therapies for post‐traumatic stress disorder (PTSD) in adults. Cochrane Database of Systematic Reviews. 2021;(5). doi: 10.1002/14651858.CD011710.pub3.

85. Smith C, Gold J, Ngo TD, Sumpter C, Free C. Mobile phone‐based interventions for improving contraception use. Cochrane Database of Systematic Reviews. 2015;(6). doi: 10.1002/14651858.CD011159.pub2.

86. Smith S, Calthorpe R, Herbert S, Smyth AR. Digital technology for monitoring adherence to inhaled therapies in people with cystic fibrosis. Cochrane Database of Systematic Reviews. 2023;(2). doi: 10.1002/14651858.CD013733.pub2.

87. Stevenson JK, Campbell ZC, Webster AC, Chow CK, Tong A, Craig JC, Campbell KL, Lee VWS. eHealth interventions for people with chronic kidney disease. Cochrane Database of Systematic Reviews. 2019;(8). doi: 10.1002/14651858.CD012379.pub2.

88. Tailor V, Ludden S, Bossi M, Bunce C, Greenwood JA, Dahlmann-Noor A. Binocular versus standard occlusion or blurring treatment for unilateral amblyopia in children aged three to eight years. Cochrane Database of Systematic Reviews. 2022;(2). doi: 10.1002/14651858.CD011347.pub3.

89. Tan K, Lai NM. Telemedicine for the support of parents of high‐risk newborn infants. Cochrane Database of Systematic Reviews. 2012;(6). doi: 10.1002/14651858.CD006818.pub2.

90. Taylor GMJ, Dalili MN, Semwal M, Civljak M, Sheikh A, Car J. Internet‐based interventions for smoking cessation. Cochrane Database of Systematic Reviews. 2017;(9). doi: 10.1002/14651858.CD007078.pub5.

91. Thabrew H, Stasiak K, Hetrick SE, Wong S, Huss JH, Merry SN. E‐Health interventions for anxiety and depression in children and adolescents with long‐term physical conditions. Cochrane Database of Systematic Reviews. 2018;(8). doi: 10.1002/14651858.CD012489.pub2.

92. Treanor CJ, McMenamin UC, O'Neill RF, Cardwell CR, Clarke MJ, Cantwell MM, Donnelly M. Non‐pharmacological interventions for cognitive impairment due to systemic cancer treatment. Cochrane Database of Systematic Reviews. 2016;(8). doi: 10.1002/14651858.CD011325.pub2.

93. Välimäki M, Hätönen HM, Lahti ME, Kurki M, Hottinen A, Metsäranta K, Riihimäki T, Adams CE. Virtual reality for treatment compliance for people with serious mental illness. Cochrane Database of Systematic Reviews. 2014;(10). doi: 10.1002/14651858.CD009928.pub2.

94. Vaona A, Banzi R, Kwag KH, Rigon G, Cereda D, Pecoraro V, Tramacere I, Moja L. E‐learning for health professionals. Cochrane Database of Systematic Reviews. 2018;(1). doi: 10.1002/14651858.CD011736.pub2.

95. Vijayaraghavan M, Elser H, Frazer K, Lindson N, Apollonio D. Interventions to reduce tobacco use in people experiencing homelessness. Cochrane Database of Systematic Reviews. 2020;(12). doi: 10.1002/14651858.CD013413.pub2.

96. Vodopivec‐Jamsek V, de Jongh T, Gurol‐Urganci I, Atun R, Car J. Mobile phone messaging for preventive health care. Cochrane Database of Systematic Reviews. 2012;(12). doi: 10.1002/14651858.CD007457.pub2.

97. Wei I, Pappas Y, Car J, Sheikh A, Majeed A. Computer‐assisted versus oral‐and‐written dietary history taking for diabetes mellitus. Cochrane Database of Systematic Reviews. 2011;(12). doi: 10.1002/14651858.CD008488.pub2.

98. Whittaker R, McRobbie H, Bullen C, Rodgers A, Gu Y, Dobson R. Mobile phone text messaging and app‐based interventions for smoking cessation. Cochrane Database of Systematic Reviews. 2019;(10). doi: 10.1002/14651858.CD006611.pub5.

99. Wieland LS, Falzon L, Sciamanna CN, Trudeau KJ, Brodney Folse S, Schwartz JE, Davidson KW. Interactive computer‐based interventions for weight loss or weight maintenance in overweight or obese people. Cochrane Database of Systematic Reviews. 2012;(8). doi: 10.1002/14651858.CD007675.pub2.

100. Wong CH, Smith S, Kansra S. Digital technology for early identification of exacerbations in people with cystic fibrosis. Cochrane Database of Systematic Reviews. 2023;(4). doi: 10.1002/14651858.CD014606.pub2.

101. Meyer B, Atherton H, Sawmynaden P, Car J. Email for communicating results of diagnostic medical investigations to patients. Cochrane Database of Systematic Reviews. 2012;(8). doi: 10.1002/14651858.CD007980.pub2.

102. Martin S, Kelly G, Kernohan WG, McCreight B, Nugent C. Smart home technologies for health and social care support. Cochrane Database of Systematic Reviews. 2008;(4). doi: 10.1002/14651858.CD006412.pub2.

103. Pappas Y, Wei I, Car J, Majeed A, Sheikh A. Computer‐assisted versus oral‐and‐written family history taking for identifying people with elevated risk of type 2 diabetes mellitus. Cochrane Database of Systematic Reviews. 2011;(12). doi: 10.1002/14651858.CD008489.pub2.

104. Lawrence S, De Silva M, Henley R. Sports and games for post‐traumatic stress disorder (PTSD). Cochrane Database of Systematic Reviews. 2010;(1). doi: 10.1002/14651858.CD007171.pub2.

105. Badawy SM, Morrone K, Thompson A, Palermo TM. Computer and mobile technology interventions to promote medication adherence and disease management in people with thalassemia. Cochrane Database of Systematic Reviews. 2019;(6). doi: 10.1002/14651858.CD012900.pub2.

106. Atherton H, Sawmynaden P, Meyer B, Car J. Email for the coordination of healthcare appointments and attendance reminders. Cochrane Database of Systematic Reviews. 2012;(8). doi: 10.1002/14651858.CD007981.pub2.

107. Westergaard LW, Bossuyt PMM, Van der Veen F, van Wely M. Human menopausal gonadotropin versus recombinant follicle stimulation hormone for ovarian stimulation in assisted reproductive cycles. Cochrane Database of Systematic Reviews. 2011;(2). doi: 10.1002/14651858.CD003973.pub2.

108. Daya S, Maheshwari A, Siristatidis CS, Bhattacharya S, Gibreel AF. Gonadotrophin‐releasing hormone agonist protocols for pituitary desensitization in in vitro fertilization and gamete intrafallopian transfer cycles. Cochrane Database of Systematic Reviews. 2000;(1). doi: 10.1002/14651858.CD001299.

109. Archambault PM, van de Belt TH, Kuziemsky C, Plaisance A, Dupuis A, McGinn CA, Francois R, Gagnon MP, Turgeon AF, Horsley T *et al*. Collaborative writing applications in healthcare: effects on professional practice and healthcare outcomes. Cochrane Database of Systematic Reviews. 2017;(5). doi: 10.1002/14651858.CD011388.pub2.

110. Hoe VCW, Urquhart DM, Kelsall HL, Zamri EN, Sim MR. Ergonomic interventions for preventing work‐related musculoskeletal disorders of the upper limb and neck among office workers. Cochrane Database of Systematic Reviews. 2018;(10). doi: 10.1002/14651858.CD008570.pub3.

111. Farquhar C, Marjoribanks J. Assisted reproductive technology: an overview of Cochrane Reviews. Cochrane Database of Systematic Reviews. 2018;(8). doi: 10.1002/14651858.CD010537.pub5.

112. Heus P, Verbeek JH, Tikka C. Optical correction of refractive error for preventing and treating eye symptoms in computer users. Cochrane Database of Systematic Reviews. 2018;(4). doi: 10.1002/14651858.CD009877.pub2.

113. Prabhakar H, Rath S, Kalaivani M, Bhanderi N. Adrenaline with lidocaine for digital nerve blocks. Cochrane Database of Systematic Reviews. 2015;(3). doi: 10.1002/14651858.CD010645.pub2.

114. O'Connor D, Page MJ, Marshall SC, Massy‐Westropp N. Ergonomic positioning or equipment for treating carpal tunnel syndrome. Cochrane Database of Systematic Reviews. 2012;(1). doi: 10.1002/14651858.CD009600.

115. Pandian Z, Gibreel A, Bhattacharya S. In vitro fertilisation for unexplained subfertility. Cochrane Database of Systematic Reviews. 2015;(11). doi: 10.1002/14651858.CD003357.pub4.

116. Cantineau AEP, Cohlen BJ, Heineman MJ, Marjoribanks J, Farquhar C. Intrauterine insemination versus fallopian tube sperm perfusion for non‐tubal infertility. Cochrane Database of Systematic Reviews. 2013;(10). doi: 10.1002/14651858.CD001502.pub4.

117. Wilson C, Willis C, Hendrikz JK, Le Brocque R, Bellamy N. Speed cameras for the prevention of road traffic injuries and deaths. Cochrane Database of Systematic Reviews. 2010;(11). doi: 10.1002/14651858.CD004607.pub4.

118. Vaona A, Pappas Y, Grewal RS, Ajaz M, Majeed A, Car J. Training interventions for improving telephone consultation skills in clinicians. Cochrane Database of Systematic Reviews. 2017;(1). doi: 10.1002/14651858.CD010034.pub2.

119. Zhan L, Yang LJ, Huang Y, He Q, Liu GJ. Continuous chest compression versus interrupted chest compression for cardiopulmonary resuscitation of non‐asphyxial out‐of‐hospital cardiac arrest. Cochrane Database of Systematic Reviews. 2017;(3). doi: 10.1002/14651858.CD010134.pub2.

120. Allen VB, Gurusamy KS, Takwoingi Y, Kalia A, Davidson BR. Diagnostic accuracy of laparoscopy following computed tomography (CT) scanning for assessing the resectability with curative intent in pancreatic and periampullary cancer. Cochrane Database of Systematic Reviews. 2016;(7). doi: 10.1002/14651858.CD009323.pub3.

121. Barone DG, Lawrie TA, Hart MG. Image guided surgery for the resection of brain tumours. Cochrane Database of Systematic Reviews. 2014;(1). doi: 10.1002/14651858.CD009685.pub2.

122. Benstoem C, Stoppe C, Liakopoulos OJ, Ney J, Hasenclever D, Meybohm P, Goetzenich A. Remote ischaemic preconditioning for coronary artery bypass grafting (with or without valve surgery). Cochrane Database of Systematic Reviews. 2017;(5). doi: 10.1002/14651858.CD011719.pub3.

123. Bonney A, Malouf R, Marchal C, Manners D, Fong KM, Marshall HM, Irving LB, Manser R. Impact of low‐dose computed tomography (LDCT) screening on lung cancer‐related mortality. Cochrane Database of Systematic Reviews. 2022;(8). doi: 10.1002/14651858.CD013829.pub2.

124. Dinnes J, Ferrante di Ruffano L, Takwoingi Y, Cheung ST, Nathan P, Matin RN, Chuchu N, Chan SA, Durack A, Bayliss SE *et al*. Ultrasound, CT, MRI, or PET‐CT for staging and re‐staging of adults with cutaneous melanoma. Cochrane Database of Systematic Reviews. 2019;(7). doi: 10.1002/14651858.CD012806.pub2.

125. Eggerding V, Reijman M, Scholten R, Verhaar JAN, Meuffels DE. Computer‐assisted surgery for knee ligament reconstruction. Cochrane Database of Systematic Reviews. 2014;(9). doi: 10.1002/14651858.CD007601.pub4.

126. Ferrante di Ruffano L, Takwoingi Y, Dinnes J, Chuchu N, Bayliss SE, Davenport C, Matin RN, Godfrey K, O'Sullivan C, Gulati A *et al*. Computer‐assisted diagnosis techniques (dermoscopy and spectroscopy‐based) for diagnosing skin cancer in adults. Cochrane Database of Systematic Reviews. 2018;(12). doi: 10.1002/14651858.CD013186.

127. Grivell RM, Alfirevic Z, Gyte GML, Devane D. Antenatal cardiotocography for fetal assessment. Cochrane Database of Systematic Reviews. 2015;(9). doi: 10.1002/14651858.CD007863.pub4.

128. Hwang EC, Jung JH, Borofsky M, Kim MH, Dahm P. Aquablation of the prostate for the treatment of lower urinary tract symptoms in men with benign prostatic hyperplasia. Cochrane Database of Systematic Reviews. 2019;(2). doi: 10.1002/14651858.CD013143.pub2.

129. Lee ASY, Law J, Gibbon FE. Electropalatography for articulation disorders associated with cleft palate. Cochrane Database of Systematic Reviews. 2009;(3). doi: 10.1002/14651858.CD006854.pub2.

130. Liang F, Liu S, Liu G, Liu H, Wang Q, Song B, Yao L. Remote ischaemic preconditioning versus no remote ischaemic preconditioning for vascular and endovascular surgical procedures. Cochrane Database of Systematic Reviews. 2023;(1). doi: 10.1002/14651858.CD008472.pub3.

131. Nadarevic T, Giljaca V, Colli A, Fraquelli M, Casazza G, Miletic D, Štimac D. Computed tomography for the diagnosis of hepatocellular carcinoma in adults with chronic liver disease. Cochrane Database of Systematic Reviews. 2021;(10). doi: 10.1002/14651858.CD013362.pub2.

132. Walsh T, Macey R, Riley P, Glenny AM, Schwendicke F, Worthington HV, Clarkson JE, Ricketts D, Su TL, Sengupta A. Imaging modalities to inform the detection and diagnosis of early caries. Cochrane Database of Systematic Reviews. 2021;(3). doi: 10.1002/14651858.CD014545.

133. Zhao W, Zhang J, Sadowsky MG, Meng R, Ding Y, Ji X. Remote ischaemic conditioning for preventing and treating ischaemic stroke. Cochrane Database of Systematic Reviews. 2018;(7). doi: 10.1002/14651858.CD012503.pub2.

134. Ilic D, Neuberger MM, Djulbegovic M, Dahm P. Screening for prostate cancer. Cochrane Database of Systematic Reviews. 2013;(1). doi: 10.1002/14651858.CD004720.pub3.

135. Alexander S, Boulvain M, Ceysens G, Haelterman E, Zhang WH. Repeat digital cervical assessment in pregnancy for identifying women at risk of preterm labour. Cochrane Database of Systematic Reviews. 2010;(6). doi: 10.1002/14651858.CD005940.pub2.

136. Lin PT, Wang SH, Chi CC. Low molecular weight heparin for prevention of microvascular occlusion in digital replantation. Cochrane Database of Systematic Reviews. 2020;(4). doi: 10.1002/14651858.CD009894.pub3.

137. Beishon LC, Elliott E, Hietamies TM, Mc Ardle R, O'Mahony A, Elliott AR, Quinn TJ. Diagnostic test accuracy of remote, multidomain cognitive assessment (telephone and video call) for dementia. Cochrane Database of Systematic Reviews. 2022;(4). doi: 10.1002/14651858.CD013724.pub2.

138. Chuchu N, Dinnes J, Takwoingi Y, Matin RN, Bayliss SE, Davenport C, Moreau JF, Bassett O, Godfrey K, O'Sullivan C *et al*. Teledermatology for diagnosing skin cancer in adults. Cochrane Database of Systematic Reviews. 2018;(12). doi: 10.1002/14651858.CD013193.

139. Chuchu N, Takwoingi Y, Dinnes J, Matin RN, Bassett O, Moreau JF, Bayliss SE, Davenport C, Godfrey K, O'Connell S *et al*. Smartphone applications for triaging adults with skin lesions that are suspicious for melanoma. Cochrane Database of Systematic Reviews. 2018;(12). doi: 10.1002/14651858.CD013192.

140. McCleery J, Laverty J, Quinn TJ. Diagnostic test accuracy of telehealth assessment for dementia and mild cognitive impairment. Cochrane Database of Systematic Reviews. 2021;(7). doi: 10.1002/14651858.CD013786.pub2.

141. Young T, Hopewell S. Methods for obtaining unpublished data. Cochrane Database of Systematic Reviews. 2011;(11). doi: 10.1002/14651858.MR000027.pub2.

142. Treweek S, Pitkethly M, Cook J, Fraser C, Mitchell E, Sullivan F, Jackson C, Taskila TK, Gardner H. Strategies to improve recruitment to randomised trials. Cochrane Database of Systematic Reviews. 2018;(2). doi: 10.1002/14651858.MR000013.pub6.

143. Xyrichis A, Iliopoulou K, Mackintosh NJ, Bench S, Terblanche M, Philippou J, Sandall J. Healthcare stakeholders’ perceptions and experiences of factors affecting the implementation of critical care telemedicine (CCT): qualitative evidence synthesis. Cochrane Database of Systematic Reviews. 2021;(2). doi: 10.1002/14651858.CD012876.pub2.

144. Barth J, Jacob T, Daha I, Critchley JA. Psychosocial interventions for smoking cessation in patients with coronary heart disease. Cochrane Database of Systematic Reviews. 2015;(7). doi: 10.1002/14651858.CD006886.pub2.

145. Bunn F, Byrne G, Kendall S. Telephone consultation and triage: effects on health care use and patient satisfaction. Cochrane Database of Systematic Reviews. 2004;(3). doi: 10.1002/14651858.CD004180.pub2.

146. Corry M, Neenan K, Brabyn S, Sheaf G, Smith V. Telephone interventions, delivered by healthcare professionals, for providing education and psychosocial support for informal caregivers of adults with diagnosed illnesses. Cochrane Database of Systematic Reviews. 2019;(5). doi: 10.1002/14651858.CD012533.pub2.

147. Dale J, Caramlau IO, Lindenmeyer A, Williams SM. Peer support telephone calls for improving health. Cochrane Database of Systematic Reviews. 2008;(4). doi: 10.1002/14651858.CD006903.pub2.

148. Gentry S, van‐Velthoven M, Tudor Car L, Car J. Telephone delivered interventions for reducing morbidity and mortality in people with HIV infection. Cochrane Database of Systematic Reviews. 2013;(5). doi: 10.1002/14651858.CD009189.pub2.

149. Kobayashi S, Hanada N, Matsuzaki M, Takehara K, Ota E, Sasaki H, Nagata C, Mori R. Assessment and support during early labour for improving birth outcomes. Cochrane Database of Systematic Reviews. 2017;(4). doi: 10.1002/14651858.CD011516.pub2.

150. Lavender T, Richens Y, Milan SJ, Smyth RMD, Dowswell T. Telephone support for women during pregnancy and the first six weeks postpartum. Cochrane Database of Systematic Reviews. 2013;(7). doi: 10.1002/14651858.CD009338.pub2.

151. Lins S, Hayder‐Beichel D, Rücker G, Motschall E, Antes G, Meyer G, Langer G. Efficacy and experiences of telephone counselling for informal carers of people with dementia. Cochrane Database of Systematic Reviews. 2014;(9). doi: 10.1002/14651858.CD009126.pub2.

152. Matkin W, Ordóñez‐Mena JM, Hartmann‐Boyce J. Telephone counselling for smoking cessation. Cochrane Database of Systematic Reviews. 2019;(5). doi: 10.1002/14651858.CD002850.pub4.

153. Mistiaen P, Poot E. Telephone follow‐up, initiated by a hospital‐based health professional, for postdischarge problems in patients discharged from hospital to home. Cochrane Database of Systematic Reviews. 2006;(4). doi: 10.1002/14651858.CD004510.pub3.

154. Ream E, Hughes AE, Cox A, Skarparis K, Richardson A, Pedersen VH, Wiseman T, Forbes A, Bryant A. Telephone interventions for symptom management in adults with cancer. Cochrane Database of Systematic Reviews. 2020;(6). doi: 10.1002/14651858.CD007568.pub2.

155. Richards J, Thorogood M, Hillsdon M, Foster C. Face‐to‐face versus remote and web 2.0 interventions for promoting physical activity. Cochrane Database of Systematic Reviews. 2013;(9). doi: 10.1002/14651858.CD010393.pub2.

156. Tudor Car L, Gentry S, van‐Velthoven M, Car J. Telephone communication of HIV testing results for improving knowledge of HIV infection status. Cochrane Database of Systematic Reviews. 2013;(1). doi: 10.1002/14651858.CD009192.pub2.

157. van‐Velthoven M, Tudor Car L, Gentry S, Car J. Telephone delivered interventions for preventing HIV infection in HIV‐negative persons. Cochrane Database of Systematic Reviews. 2013;(5). doi: 10.1002/14651858.CD009190.pub2.

158. Whitford HM, Wallis SK, Dowswell T, West HM, Renfrew MJ. Breastfeeding education and support for women with twins or higher order multiples. Cochrane Database of Systematic Reviews. 2017;(2). doi: 10.1002/14651858.CD012003.pub2.

159. Akl EA, Kairouz VF, Sackett KM, Erdley WS, Mustafa RA, Fiander M, Gabriel C, Schünemann H. Educational games for health professionals. Cochrane Database of Systematic Reviews. 2013;(3). doi: 10.1002/14651858.CD006411.pub4.

160. Bhoopathi PS, Sheoran R. Educational games for mental health professionals. Cochrane Database of Systematic Reviews. 2006;(2). doi: 10.1002/14651858.CD001471.pub2.

161. Galaal K, Bryant A, Deane KHO, Al-Khaduri M, Lopes AD. Interventions for reducing anxiety in women undergoing colposcopy. Cochrane Database of Systematic Reviews. 2011;(12). doi: 10.1002/14651858.CD006013.pub3.

162. Sjøstrand Å, Kefalianos E, Hofslundsengen H, Guttormsen LS, Kirmess M, Lervåg A, Hulme C, Bottegaard Næss KA. Non‐pharmacological interventions for stuttering in children six years and younger. Cochrane Database of Systematic Reviews. 2021;(9). doi: 10.1002/14651858.CD013489.pub2.

163. Verbeek JH, Rajamaki B, Ijaz S, Sauni R, Toomey E, Blackwood B, Tikka C, Ruotsalainen JH, Kilinc Balci FS. Personal protective equipment for preventing highly infectious diseases due to exposure to contaminated body fluids in healthcare staff. Cochrane Database of Systematic Reviews. 2020;(5). doi: 10.1002/14651858.CD011621.pub5.

164. Arditi C, Rège‐Walther M, Durieux P, Burnand B. Computer‐generated reminders delivered on paper to healthcare professionals: effects on professional practice and healthcare outcomes. Cochrane Database of Systematic Reviews. 2017;(7). doi: 10.1002/14651858.CD001175.pub4.

165. Edwards PJ, Roberts I, Clarke MJ, DiGuiseppi C, Wentz R, Kwan I, Cooper R, Felix LM, Pratap S. Methods to increase response to postal and electronic questionnaires. Cochrane Database of Systematic Reviews. 2009;(3). doi: 10.1002/14651858.MR000008.pub4.

166. Marcano Belisario JS, Jamsek J, Huckvale K, O'Donoghue J, Morrison CP, Car J. Comparison of self‐administered survey questionnaire responses collected using mobile apps versus other methods. Cochrane Database of Systematic Reviews. 2015;(7). doi: 10.1002/14651858.MR000042.pub2.

167. Vasudevan L, Glenton C, Henschke N, Maayan N, Eyers J, Fønhus MS, Tamrat T, Mehl GL, Lewin S. Birth and death notification via mobile devices: a mixed methods systematic review. Cochrane Database of Systematic Reviews. 2021;(7). doi: 10.1002/14651858.CD012909.pub2.

168. Bittner AK, Yoshinaga PD, Rittiphairoj T, Li T. Telerehabilitation for people with low vision. Cochrane Database of Systematic Reviews. 2023;(1). doi: 10.1002/14651858.CD011019.pub4.

169. Handford C, Tynan AM, Rackal JM, Glazier R. Setting and organization of care for persons living with HIV/AIDS. Cochrane Database of Systematic Reviews. 2006;(3). doi: 10.1002/14651858.CD004348.pub2.

170. Tan K, Dear PRF, Newell SJ. Clinical decision support systems for neonatal care. Cochrane Database of Systematic Reviews. 2005;(2). doi: 10.1002/14651858.CD004211.pub2.
