# Supplementary material for: Online dissemination of Cochrane reviews on digital health technologies: a cross-sectional study
Source: Syst Rev. 2024 May 15;13:133. doi: 10.1186/s13643-024-02557-6 (PMC11095012; doi:10.1186/s13643-024-02557-6)
Supplement: Supplementary file 5 — Additional file 5. Altmetric data. [file 13643_2024_2557_MOESM5_ESM.pdf]

[1] Aali, Kariotis and Shokraneh (2020) Avatar Therapy for people with schizophrenia or related disorders

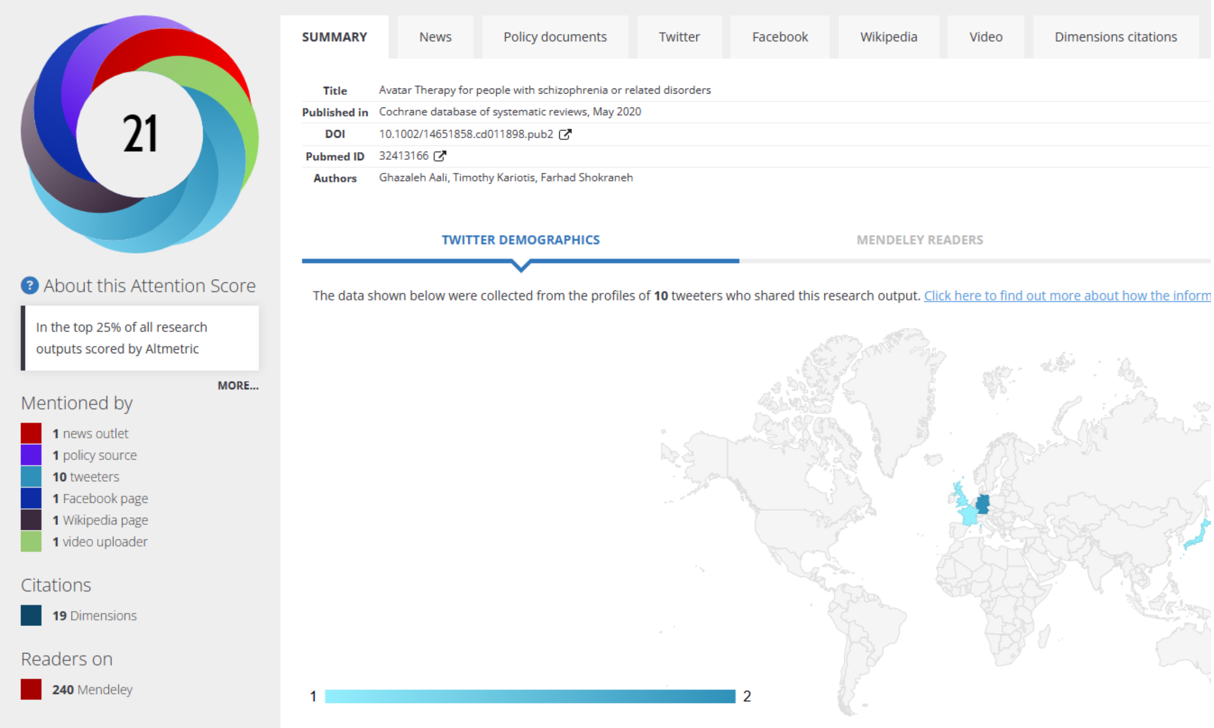

21

About this Attention Score

In the top 25% of all research outputs scored by Altmetric

MORE...

Mentioned by

1 news outlet
1 policy source
10 tweeters
1 Facebook page
1 Wikipedia page
1 video uploader

Citations

19 Dimensions

Readers on

240 Mendeley

SUMMARY | News | Policy documents | Twitter | Facebook | Wikipedia | Video | Dimensions citations

Title	Avatar Therapy for people with schizophrenia or related disorders
Published in	Cochrane database of systematic reviews, May 2020
DOI	10.1002/14651858.cd011898.pub2
Pubmed ID	32413166
Authors	Ghazaleh Aali, Timothy Kariotis, Farhad Shokraneh

TWITTER DEMOGRAPHICS | MENDELEY READERS

The data shown below were collected from the profiles of **10** tweeters who shared this research output. Click here to find out more about how the inform

1 2

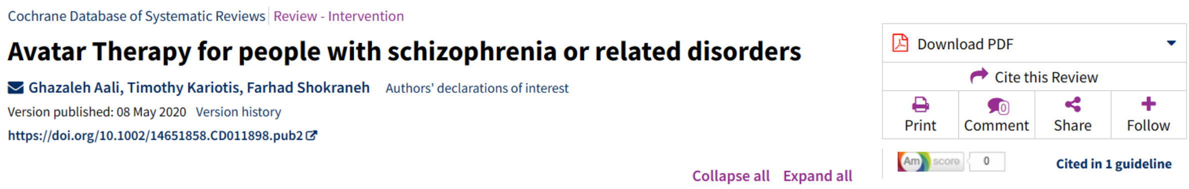

Cochrane Database of Systematic Reviews | Review - Intervention

Avatar Therapy for people with schizophrenia or related disorders

Ghazaleh Aali, Timothy Kariotis, Farhad Shokraneh Authors' declarations of interest

Version published: 08 May 2020 Version history

https://doi.org/10.1002/14651858.CD011898.pub2

Collapse all Expand all

Download PDF

Cite this Review

Print | Comment | Share | Follow

score 0

Cited in 1 guideline

[2] Adler, Martin, Mariani, Tajer, Owolabi, Free, Serrano, Casas and Perel (2017) Mobile phone text messaging to improve medication adherence in secondary prevention of cardiovascular disease

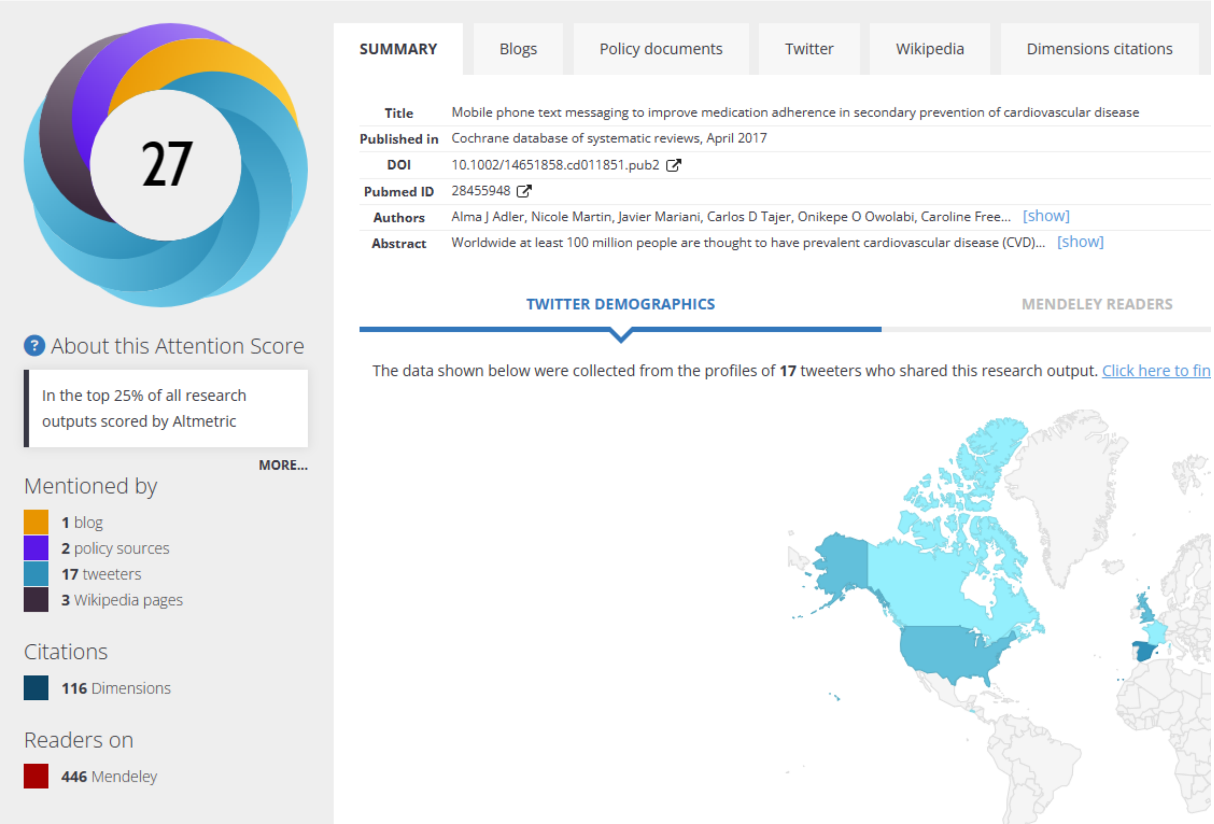

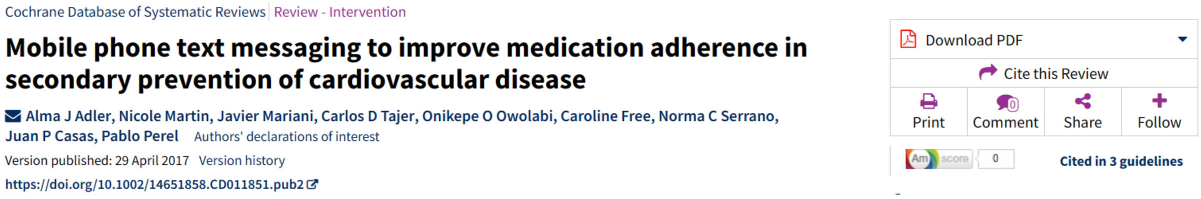

[3] Agarwal, Glenton, Henschke, Tamrat, Bergman, Følnhus, Mehl and Lewin (2020) Tracking health commodity inventory and notifying stock levels via mobile devices: a mixed methods systematic review

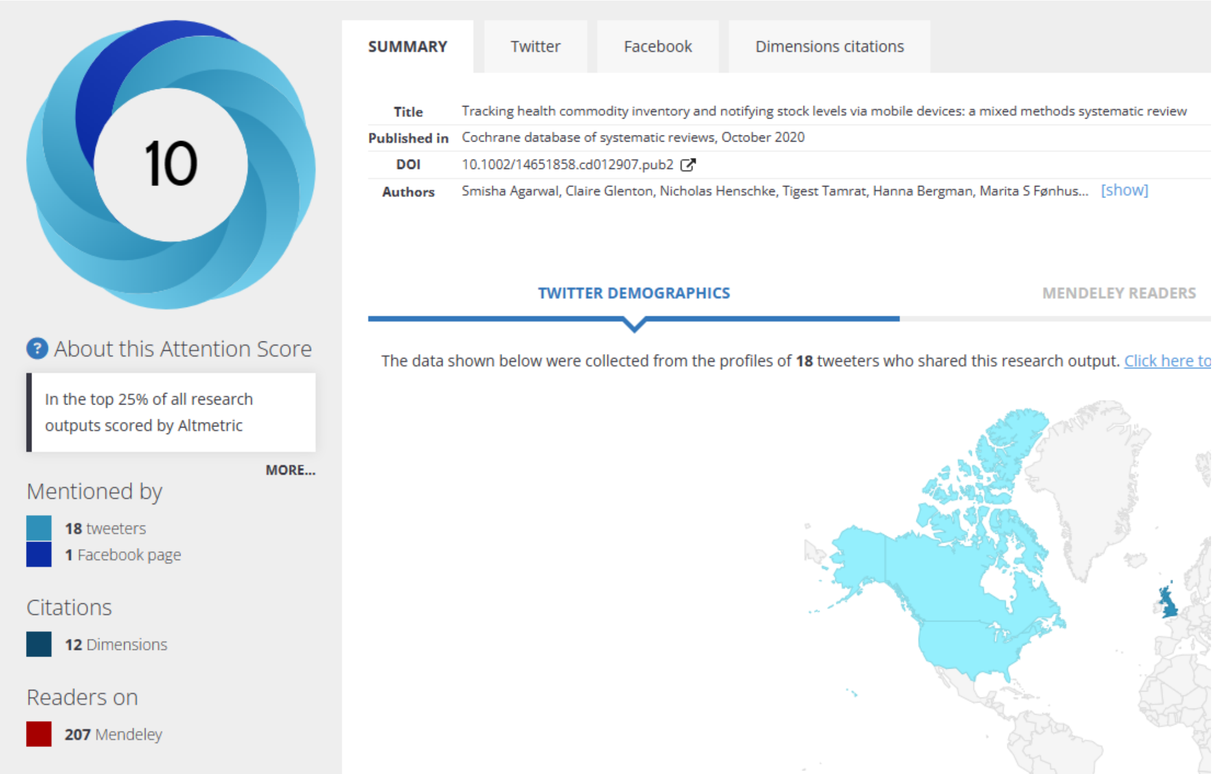

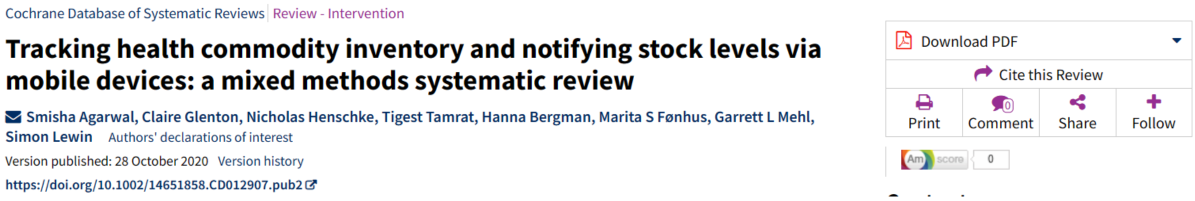

[4] Agarwal, Glenton, Tamrat, Henschke, Maayan, Fønhus, Mehl and Lewin (2021) Decision-support tools via mobile devices to improve quality of care in primary healthcare settings

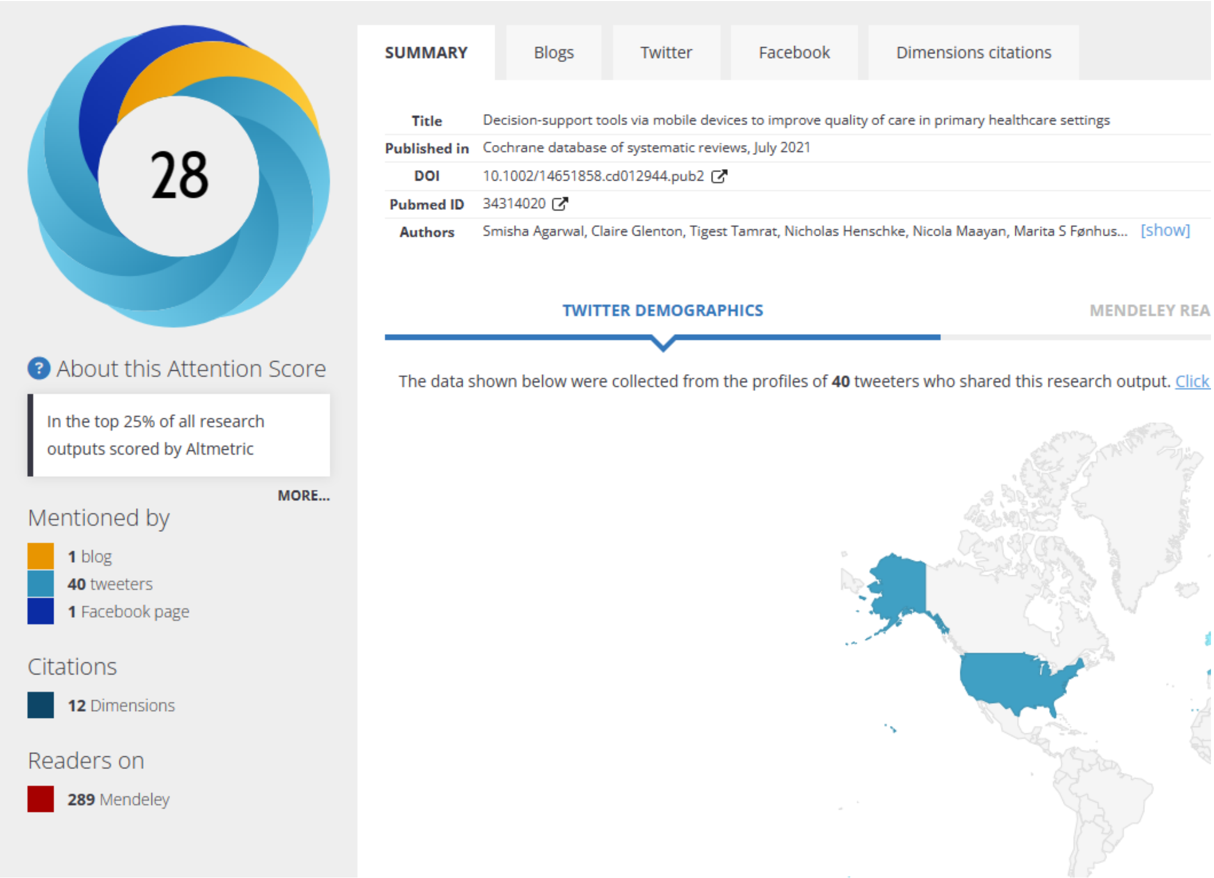

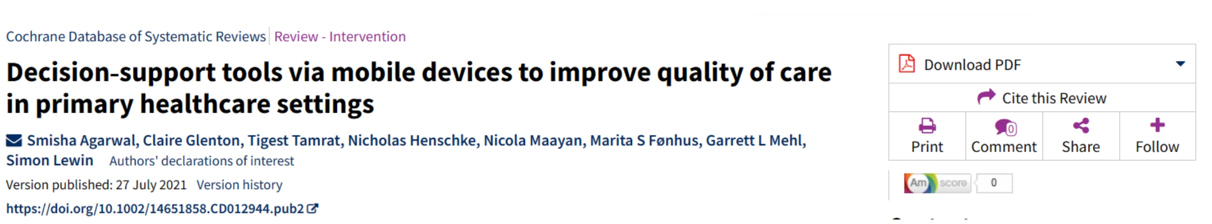

[5] Allida, Du, Xu, Prichard, Chang, Hickman, Davidson and Inglis (2020) mHealth education interventions in heart failure

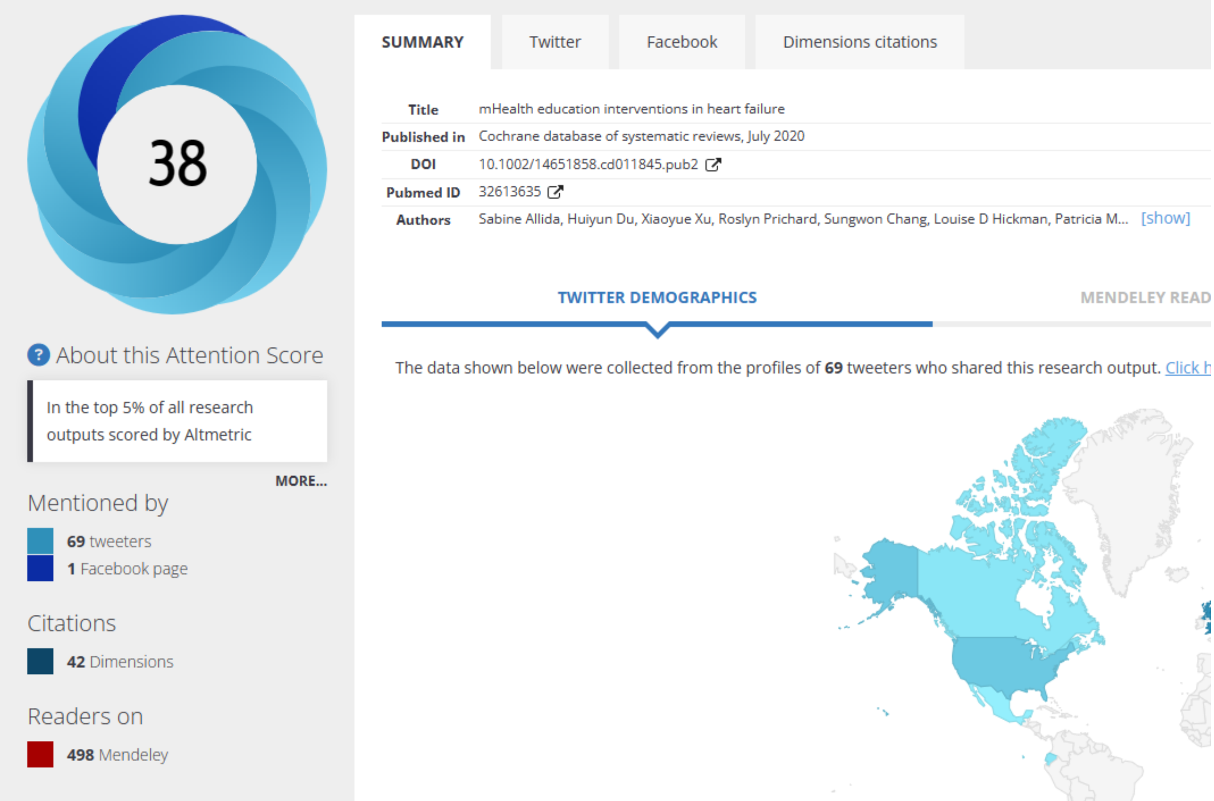

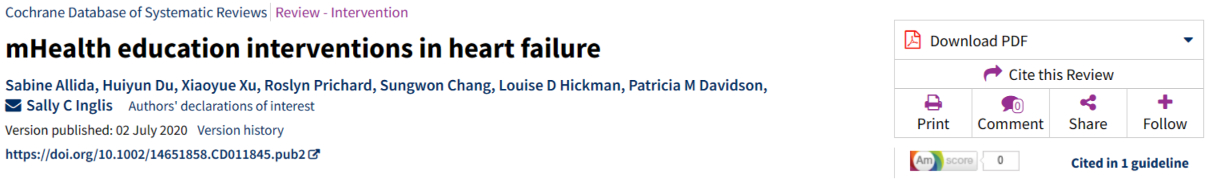

[6] Ames, Glenton, Lewin, Tamrat, Akama and Leon (2019) Clients' perceptions and experiences of targeted digital communication accessible via mobile devices for reproductive, maternal, newborn, child, and adolescent health: a qualitative evidence synthesis

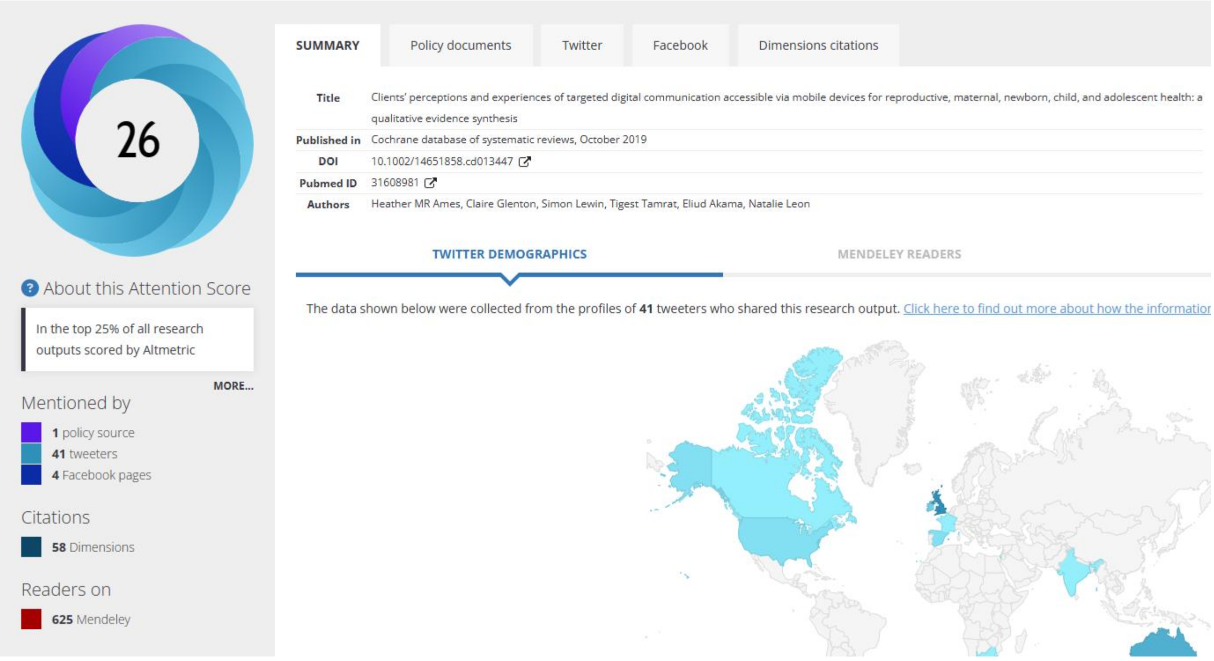

Cochrane Database of Systematic Reviews | Review - Qualitative

Clients' perceptions and experiences of targeted digital communication accessible via mobile devices for reproductive, maternal, newborn, child, and adolescent health: a qualitative evidence synthesis

Heather MR Ames, Claire Glenton, Simon Lewin, Tigest Tamrat, Eliud Akama, Natalie Leon Authors' declarations of interest

Version published: 14 October 2019 Version history

Download PDF
Cite this Review
Print Comment Share Follow
Am score 0 Cited in 3 guidelines

Contents

[7] Ammenwerth, Neyer, Hörbst, Mueller, Siebert and Schnell-Inderst (2021) Adult patient access to electronic health records

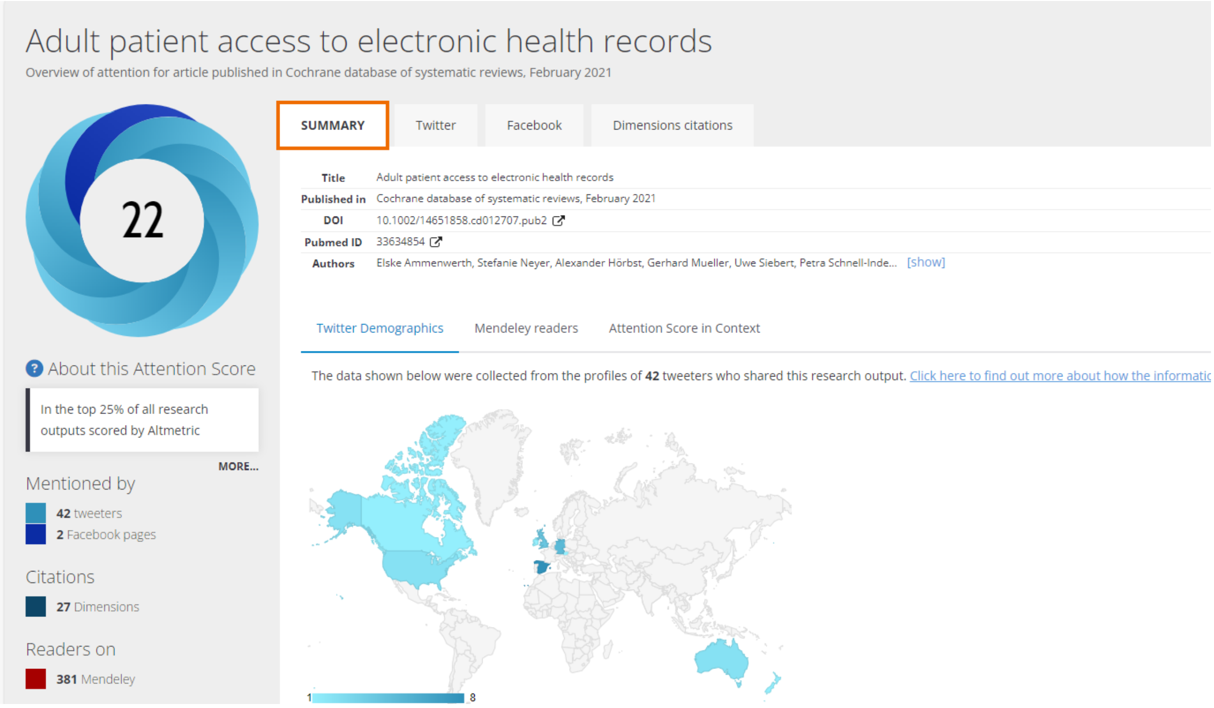

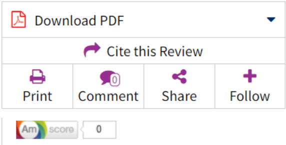

[8] Anglemyer, Moore, Parker, Chambers, Grady, Chiu, Parry, Wilczynska, Flemyng and Bero (2020) Digital contact tracing technologies in epidemics: a rapid review

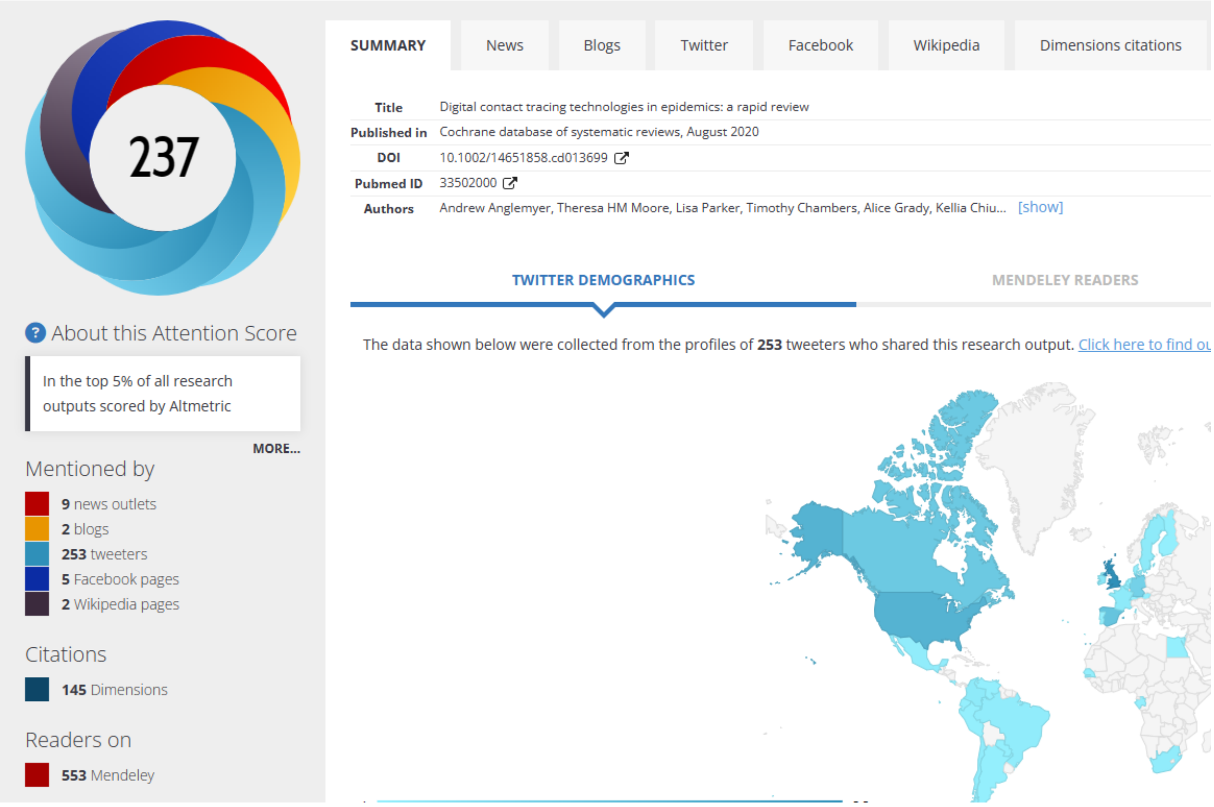

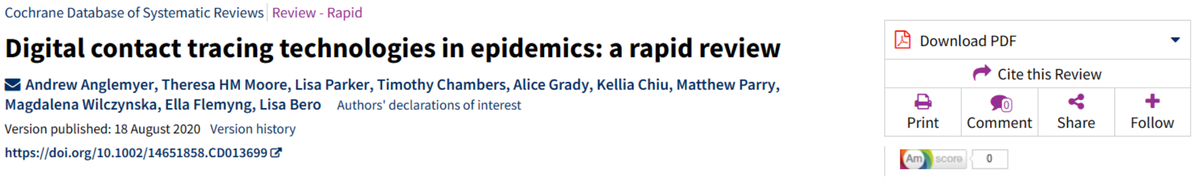

[9] Archambault, van de Belt, Kuziemsky, Plaisance, Dupuis, McGinn, Francois, Gagnon, Turgeon, Horsley and et al. (2017) Collaborative writing applications in healthcare: effects on professional practice and healthcare outcomes

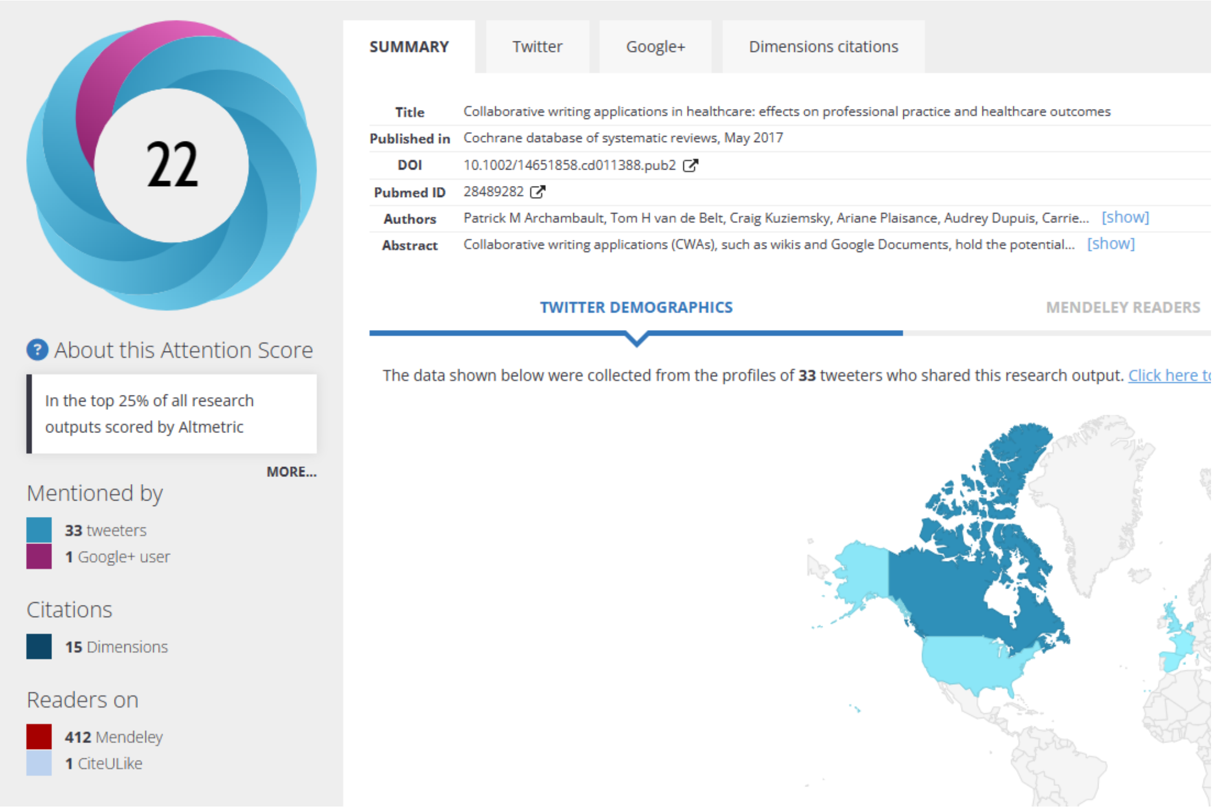

Cochrane Database of Systematic Reviews | Review - Intervention

Collaborative writing applications in healthcare: effects on professional practice and healthcare outcomes

Patrick M Archambault, Tom H van de Belt, Craig Kuziemsky, Ariane Plaisance, Audrey Dupuis, Carrie A McGinn, Rebecca Francois, Marie-Pierre Gagnon, Alexis F Turgeon, Tanya Horsley, William Witteman, Julien Poitras, Jean Lapointe, Kevin Brand, Jean Lachaine, France Légaré Authors' declarations of interest

Version published: 10 May 2017 Version history

Download PDF
Cite this Review
Print Comment Share Follow
0

[10] Arditi, Rège-Walther, Durieux and Burnand (2017) Computer-generated reminders delivered on paper to healthcare professionals: effects on professional practice and healthcare outcomes

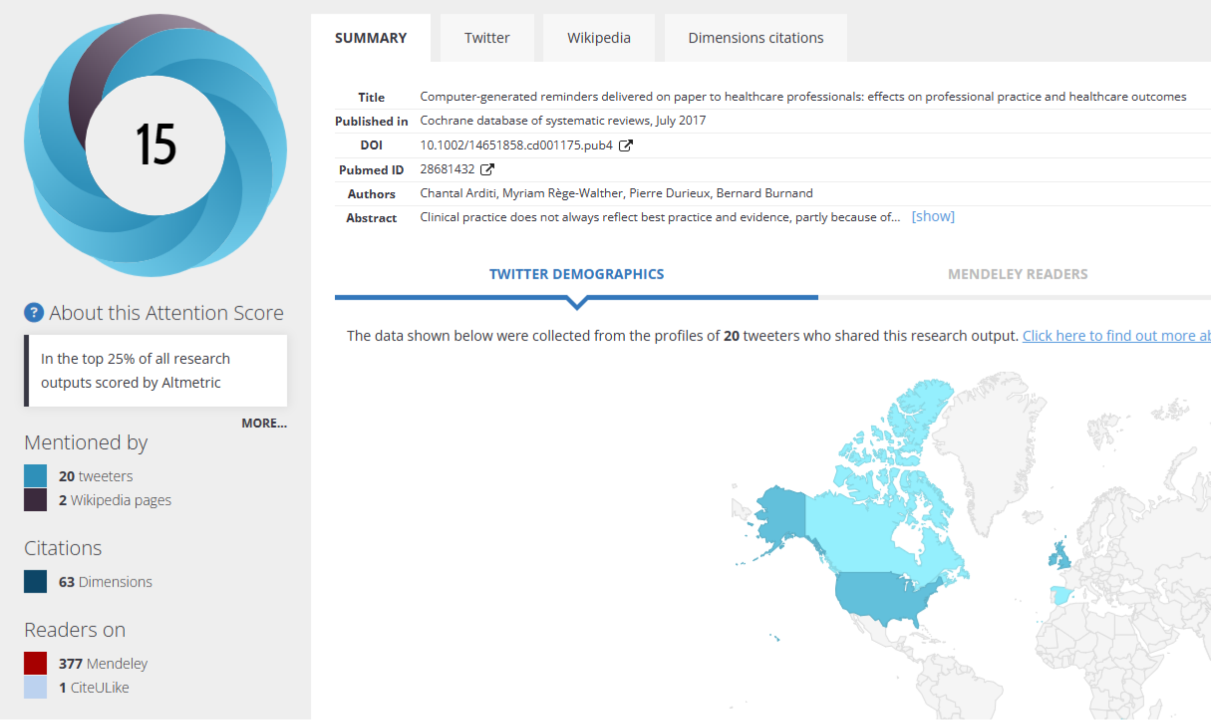
15

About this Attention Score

In the top 25% of all research outputs scored by Altmetric

MORE...

Mentioned by

20 tweeters

2 Wikipedia pages

Citations

63 Dimensions

Readers on

377 Mendeley

1 CiteULike

SUMMARY | Twitter | Wikipedia | Dimensions citations

**Title**	Computer-generated reminders delivered on paper to healthcare professionals: effects on professional practice and healthcare outcomes
**Published in**	Cochrane database of systematic reviews, July 2017
**DOI**	10.1002/14651858.cd001175.pub4
**Pubmed ID**	28681432
**Authors**	Chantal Arditi, Myriam Rège-Walther, Pierre Durieux, Bernard Burnand
**Abstract**	Clinical practice does not always reflect best practice and evidence, partly because of... [show]

TWITTER DEMOGRAPHICS | MENDELEY READERS

The data shown below were collected from the profiles of **20** tweeters who shared this research output. Click here to find out more al

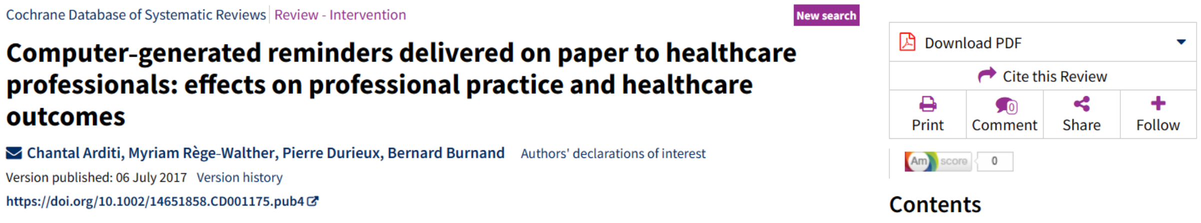
Cochrane Database of Systematic Reviews | Review - Intervention

New search

Computer-generated reminders delivered on paper to healthcare professionals: effects on professional practice and healthcare outcomes

Chantal Arditi, Myriam Rège-Walther, Pierre Durieux, Bernard Burnand Authors' declarations of interest

Version published: 06 July 2017 Version history

https://doi.org/10.1002/14651858.CD001175.pub4

Download PDF

Cite this Review

Print | Comment | Share | Follow

0

Contents

[11] Atherton, Sawmynaden, Meyer and Car (2012) Email for the coordination of healthcare appointments and attendance reminders

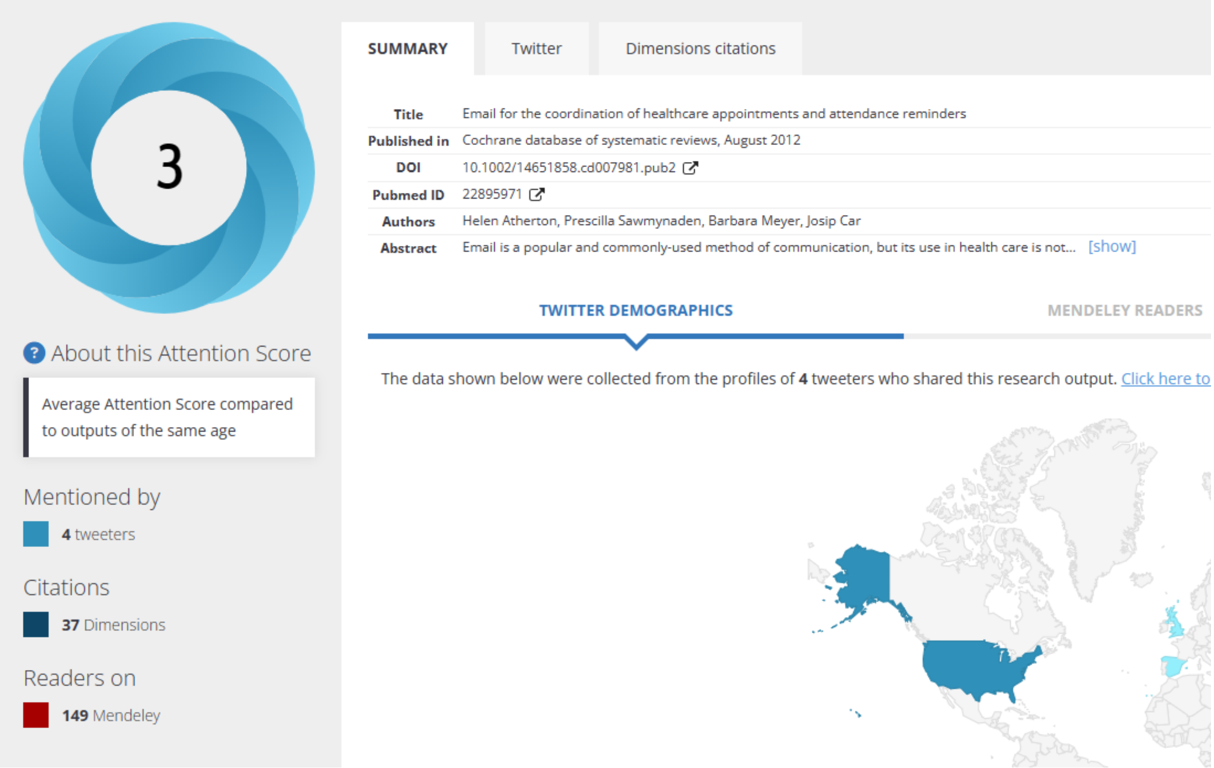

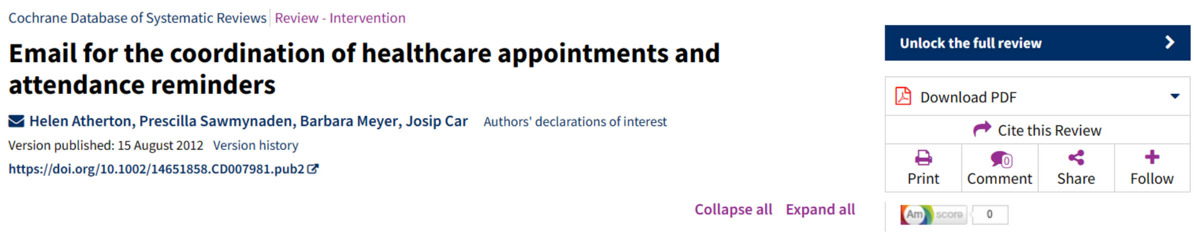

[12] Atherton, Sawmynaden, Sheikh, Majeed and Car (2012) Email for clinical communication between patients/caregivers and healthcare professionals

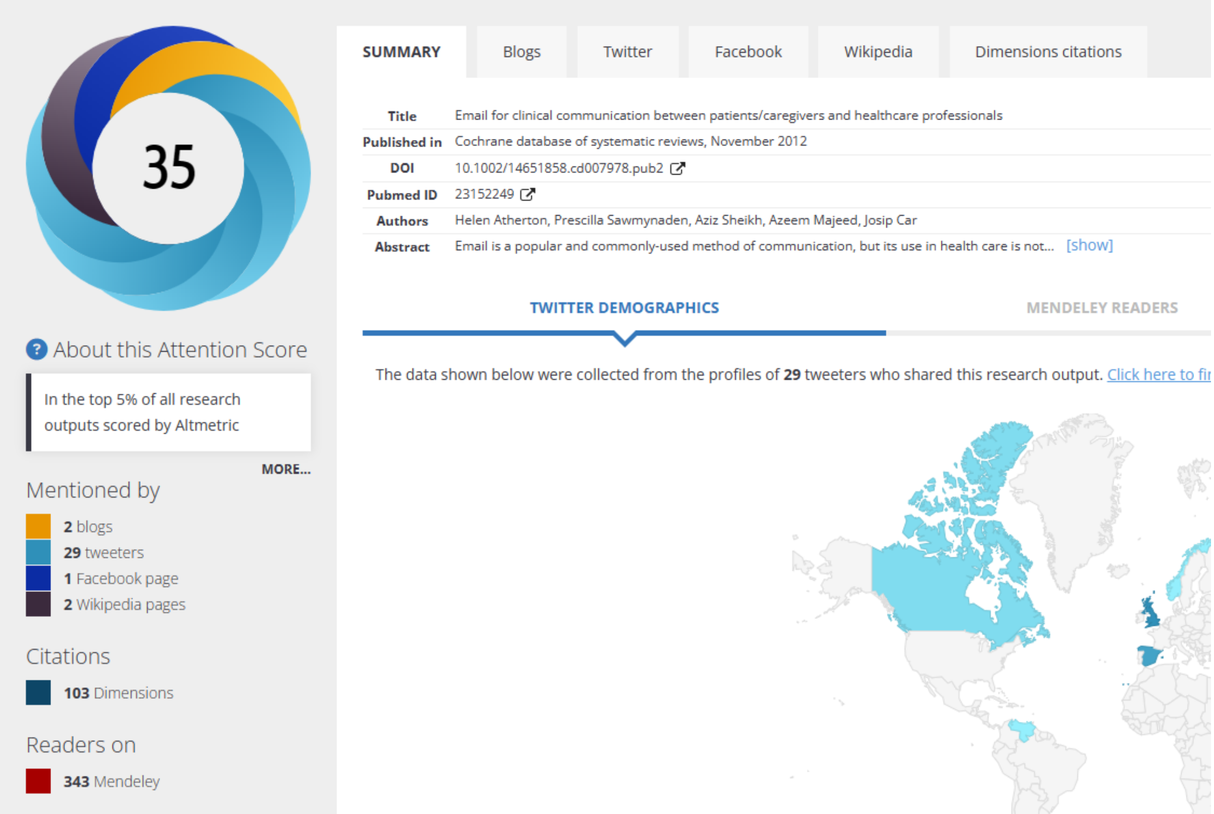

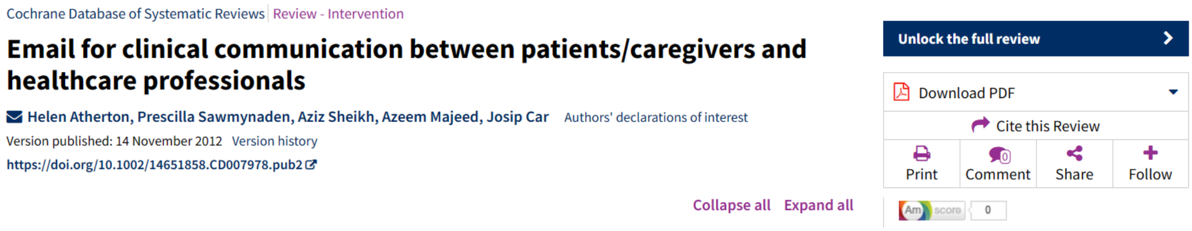

[13] Badawy, Morrone, Thompson and Palermo (2019) Computer and mobile technology interventions to promote medication adherence and disease management in people with thalassemia

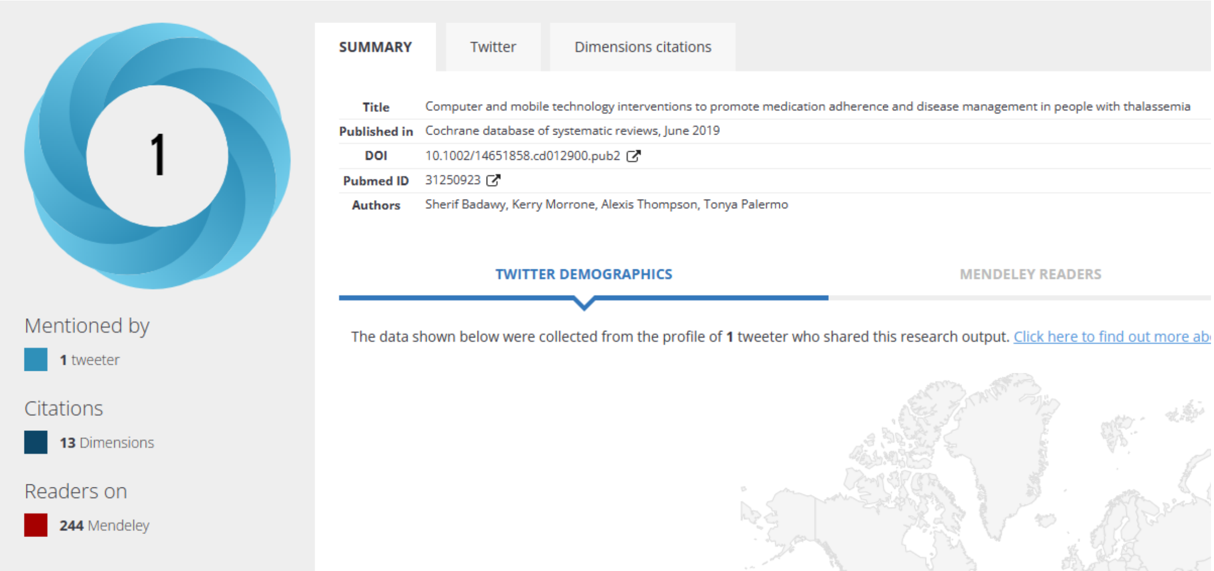

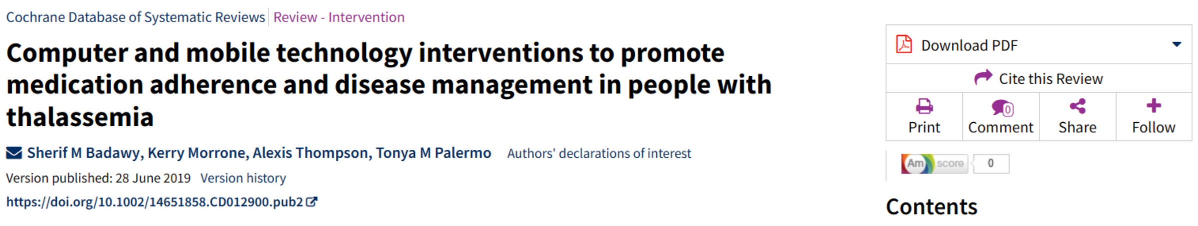

[14] Bahar-Fuchs, Martyr, Goh, Sabates and Clare (2019) Cognitive training for people with mild to moderate dementia

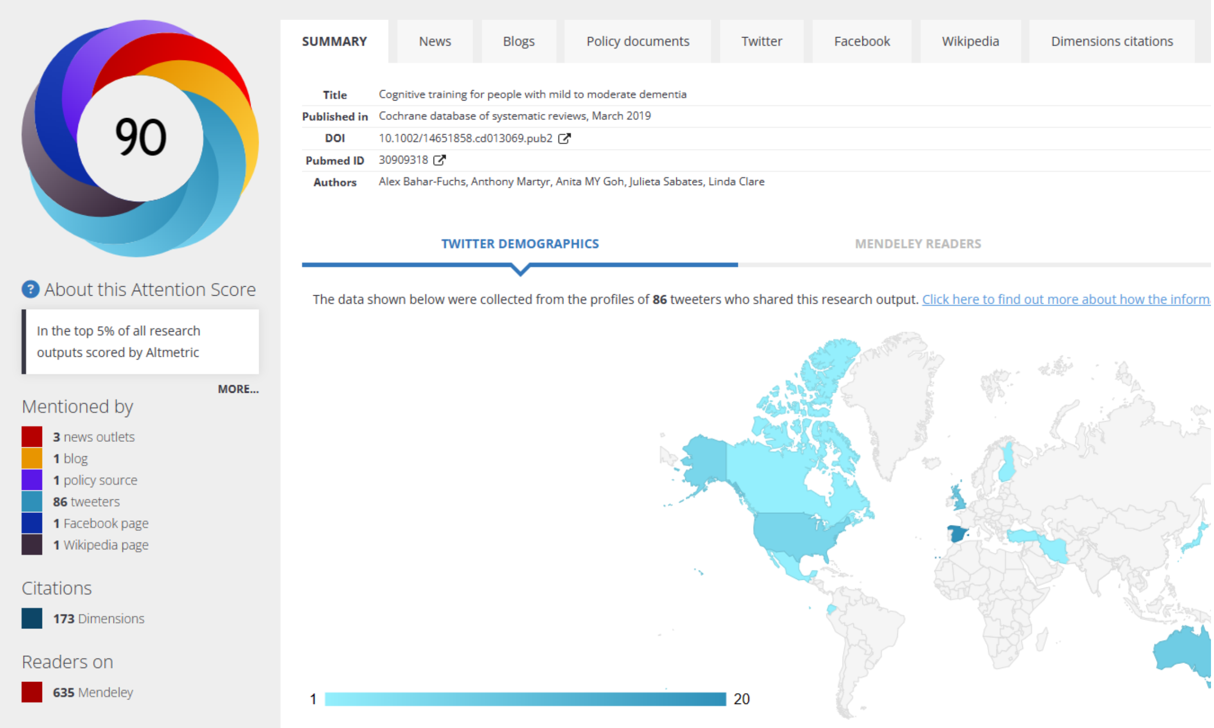

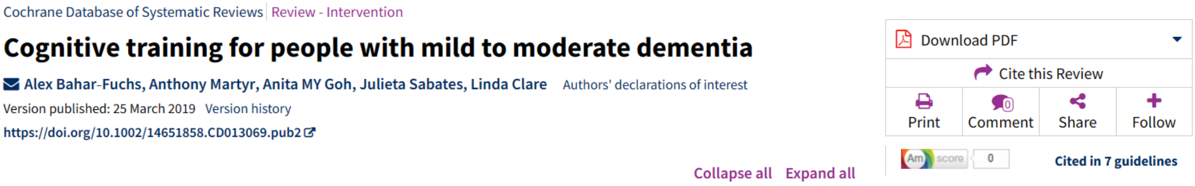

[15] Bailey, Murray, Rait, Mercer, Morris, Peacock, Cassell and Nazareth (2010) Interactive computer-based interventions for sexual health promotion

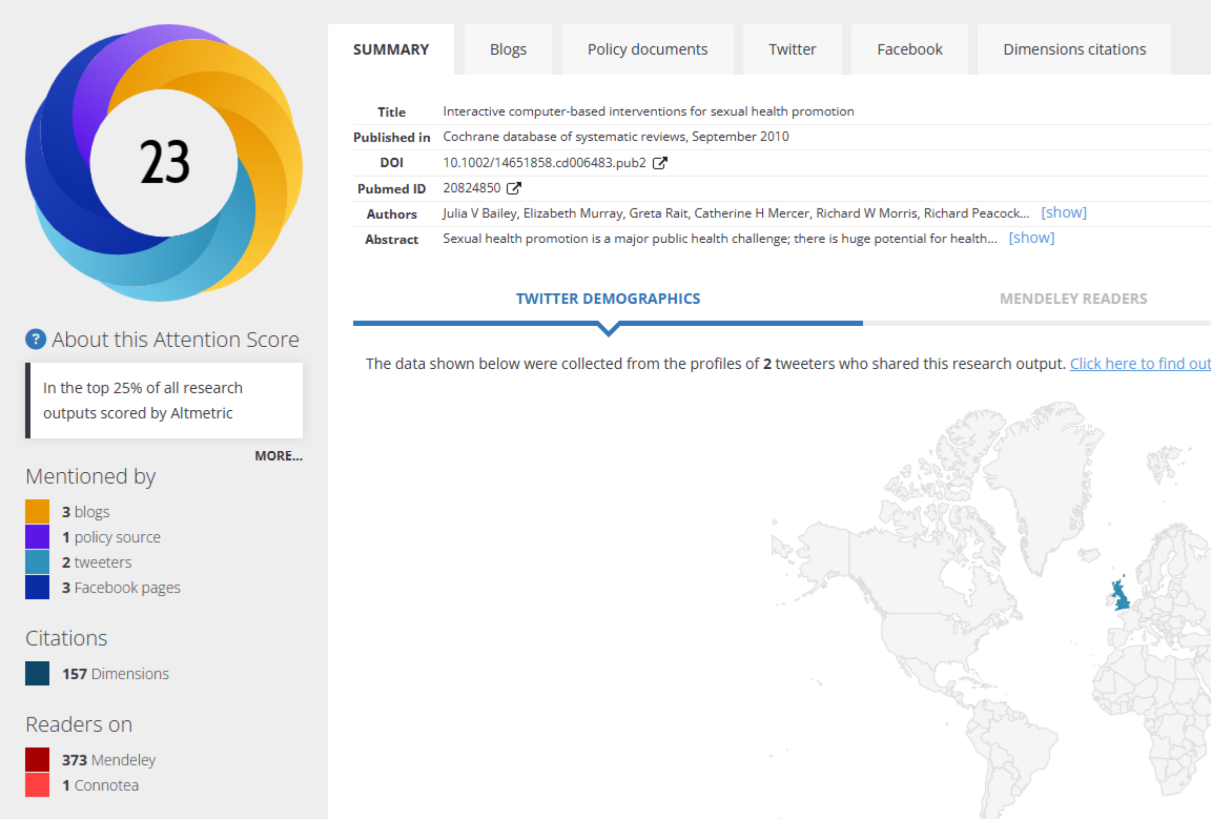

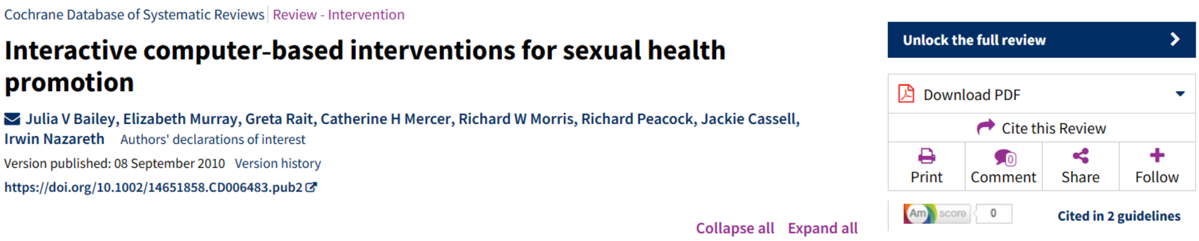

[16] Barth, Jacob, Daha and Critchley (2015) Psychosocial interventions for smoking cessation in patients with coronary heart disease

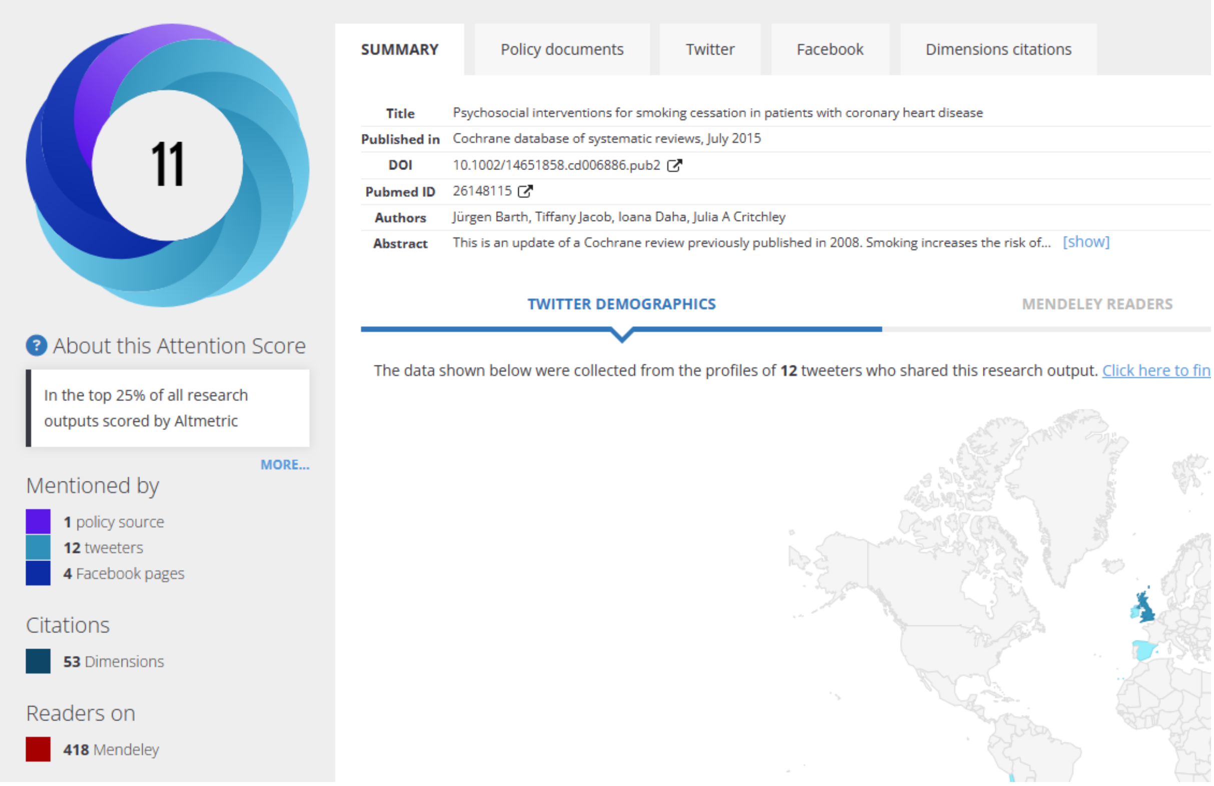

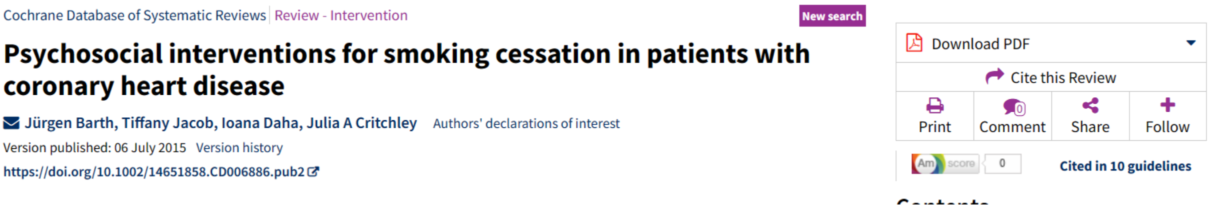

[17] Beishon, Elliott, Hietamies, Mc Ardle, O'Mahony, Elliott and Quinn (2022) Diagnostic test accuracy of remote, multidomain cognitive assessment (telephone and video call) for dementia

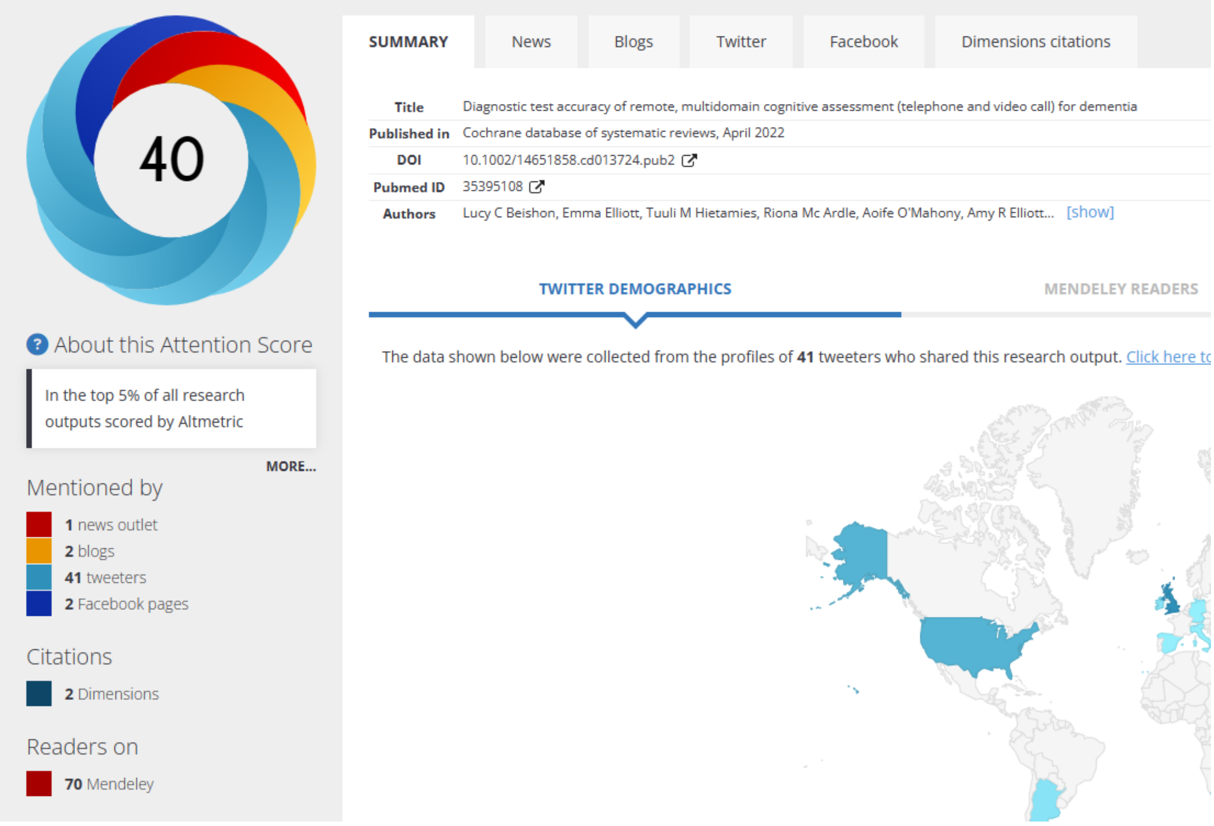

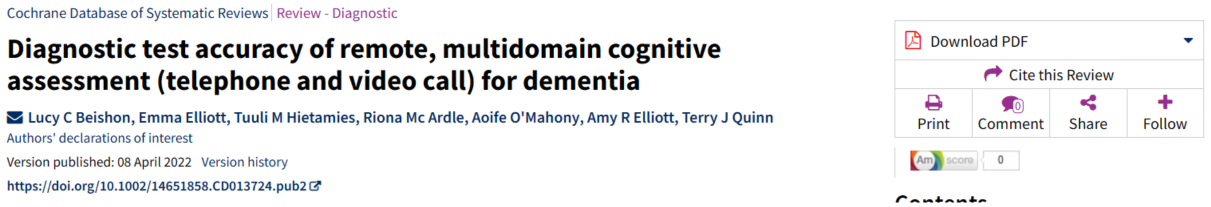

[18] Bittner, Yoshinaga, Rittiphairoj and Li (2023) Telerehabilitation for people with low vision

We haven't seen any mentions of this research output yet!

?

This is an Altmetric details page, which will display any online attention and activity that we pick up for this research output. Altmetric looks for relevant mentions in social media sites, newspapers, policy documents, blogs, Wikipedia, and many other sources.

You can look at a populated Details Page that demonstrates the types of attention we track at Altmetric.

So far, we haven't found any mentions of this research output. Please check back again later to see if anything has changed.

If you think we might be missing a mention, please fill in this form in this form and a member of our team will get back to you. You can also reach us on Twitter at @altmetric.

Want to be alerted when we find any activity for this research output? Click on the button below to receive an e-mail alert when this research output is shared for the first time.

Alert me when this research output is mentioned

Cochrane Database of Systematic Reviews | Review - Intervention

New search | Conclusions changed

Telerehabilitation for people with low vision

Ava K Bittner, Patrick D Yoshinaga, Thanitsara Rittiphairoj, Tianjing Li Authors' declarations of interest

Version published: 13 January 2023 Version history

https://doi.org/10.1002/14651858.CD011019.pub4

Collapse all Expand all

Unlock the full review

Download PDF

Cite this Review

Print | Comment | Share | Follow

Am score 0

Abstract

[19] Bunn, Byrne and Kendall (2004) Telephone consultation and triage: effects on health care use and patient satisfaction

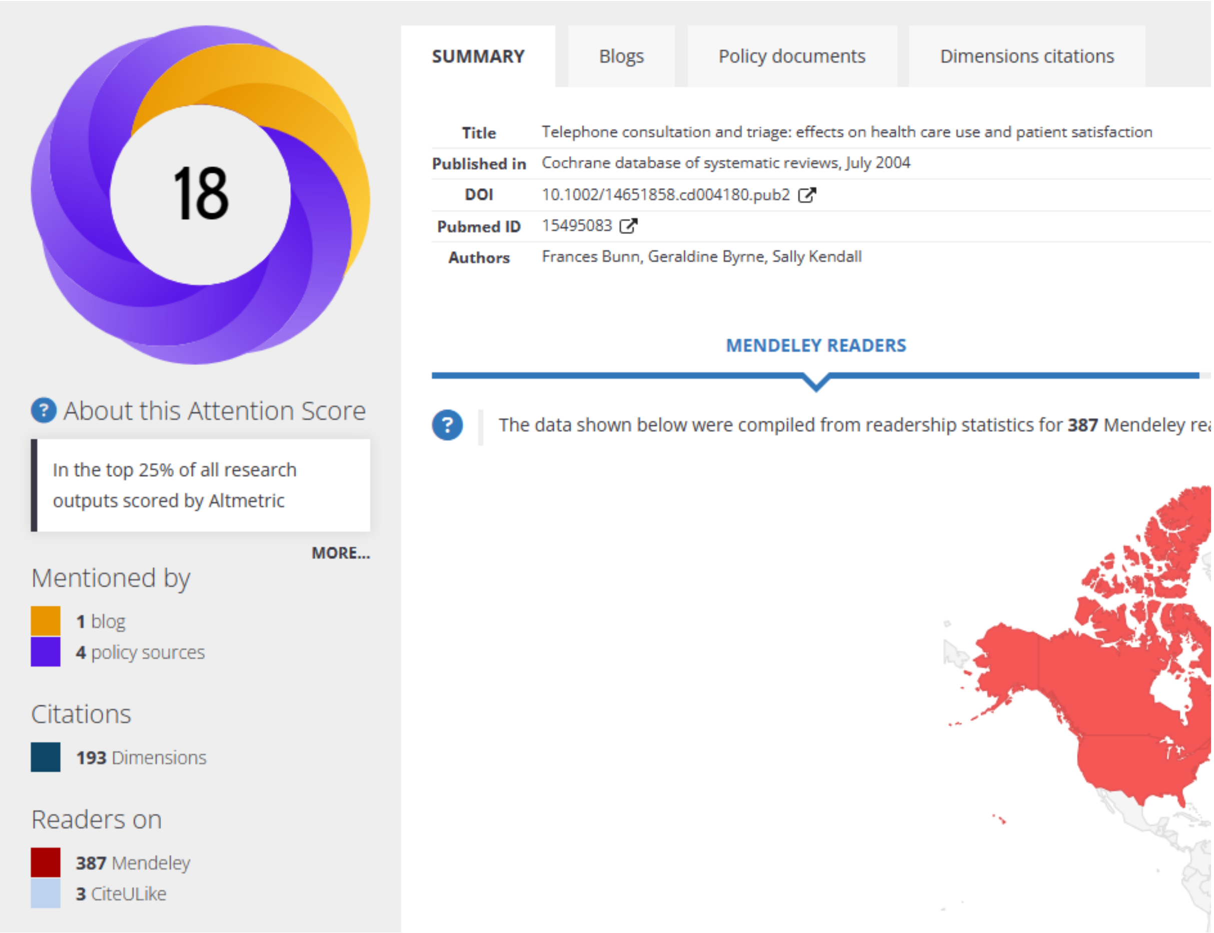

Cochrane Database of Systematic Reviews | Review - Intervention

Telephone consultation and triage: effects on health care use and patient satisfaction

Frances Bunn, Geraldine Byrne, Sally Kendall   Authors' declarations of interest

Version published: 19 July 2004   Version history

https://doi.org/10.1002/14651858.CD004180.pub2

Unlock the full review

Download PDF

Cite this Review

Print   Comment   Share   Follow

Collapse all   Expand all

[20] Boyle, Solberg and Fiore (2014) Use of electronic health records to support smoking cessation

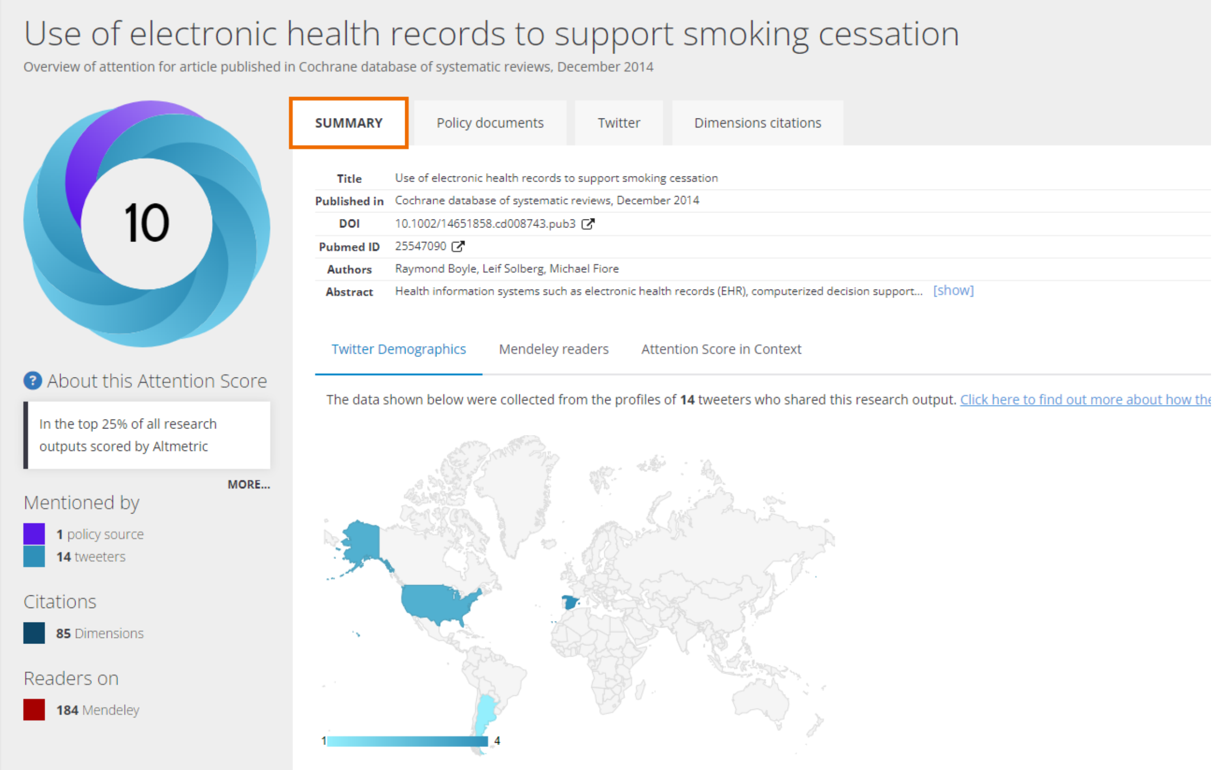

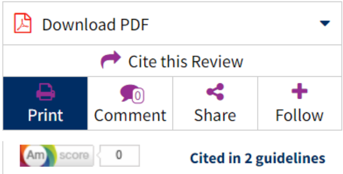

[21] Burge, Cox, Abramson and Holland (2020) Interventions for promoting physical activity in people with chronic obstructive pulmonary disease (COPD)

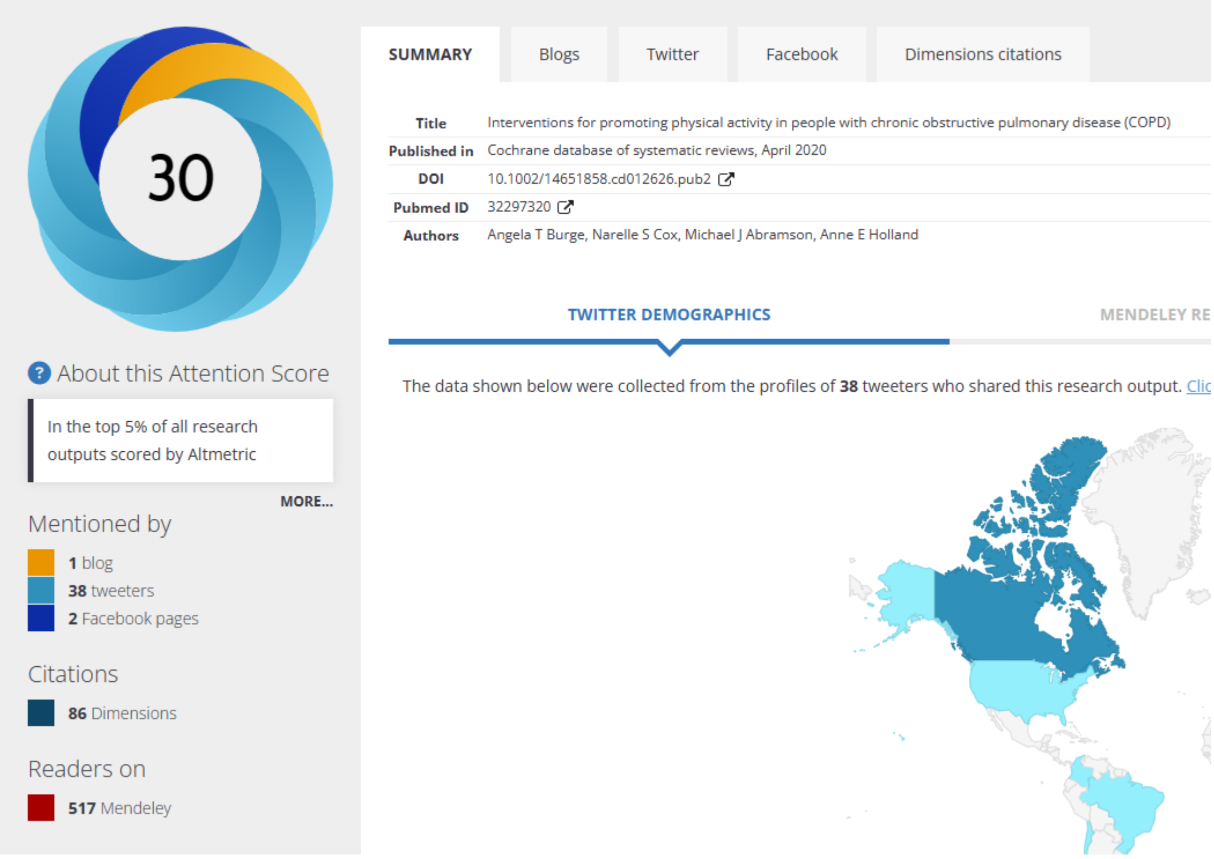

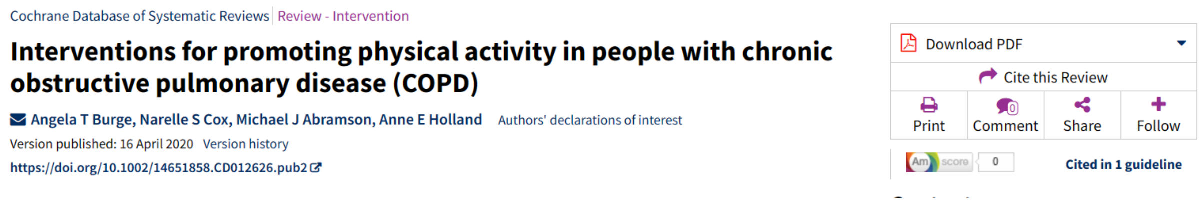

[22] Car, Lang, Colledge, Ung and Majeed (2011) Interventions for enhancing consumers' online health literacy

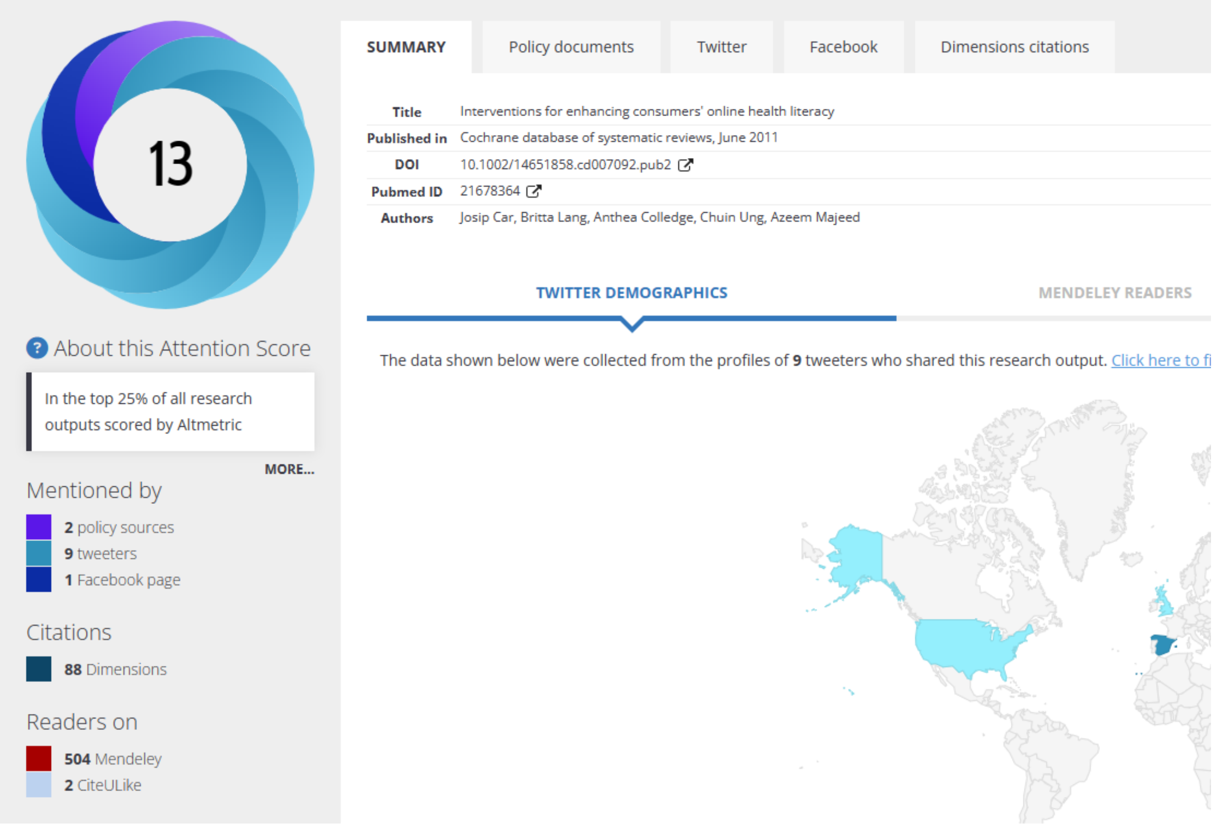

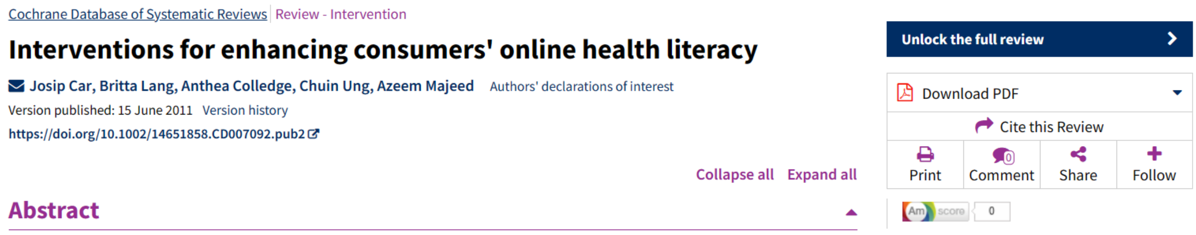

[23] Chan, De Simoni, Wileman, Holliday, Newby, Chisari, Ali, Zhu, Padakanti, Pinprachanan and et al. (2022) Digital interventions to improve adherence to maintenance medication in asthma

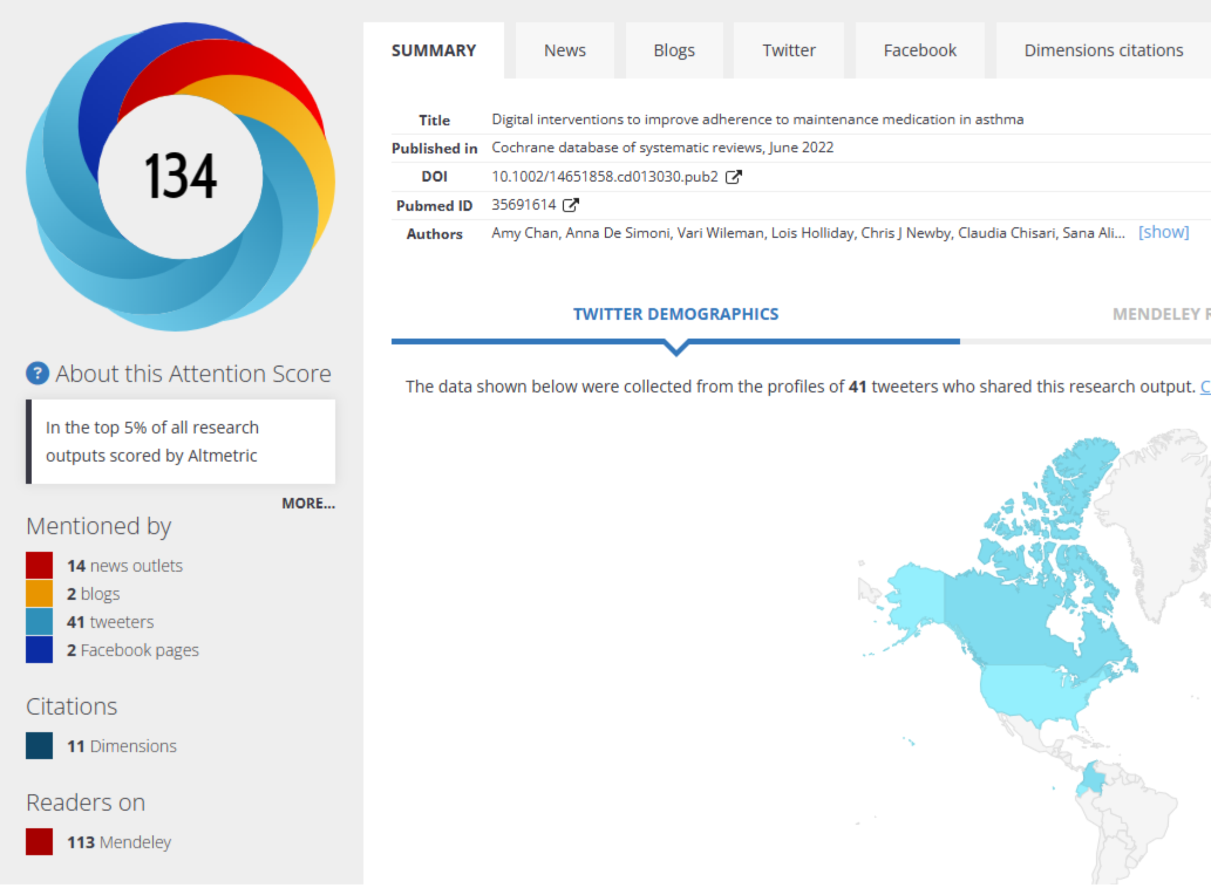

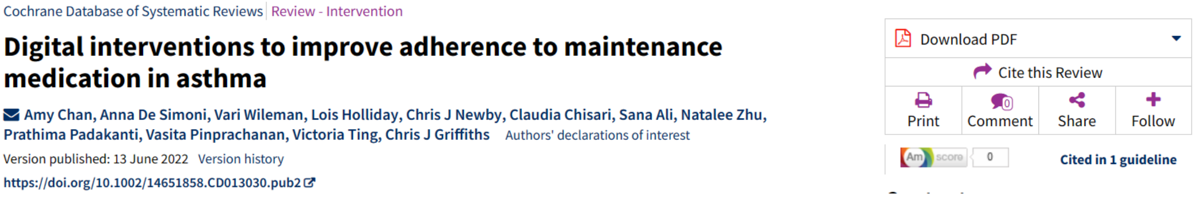

[24] Chuchu, Dinnes, Takwoingi, Matin, Bayliss, Davenport, Moreau, Bassett, Godfrey, O'Sullivan and et al. (2018) Teledermatology for diagnosing skin cancer in adults

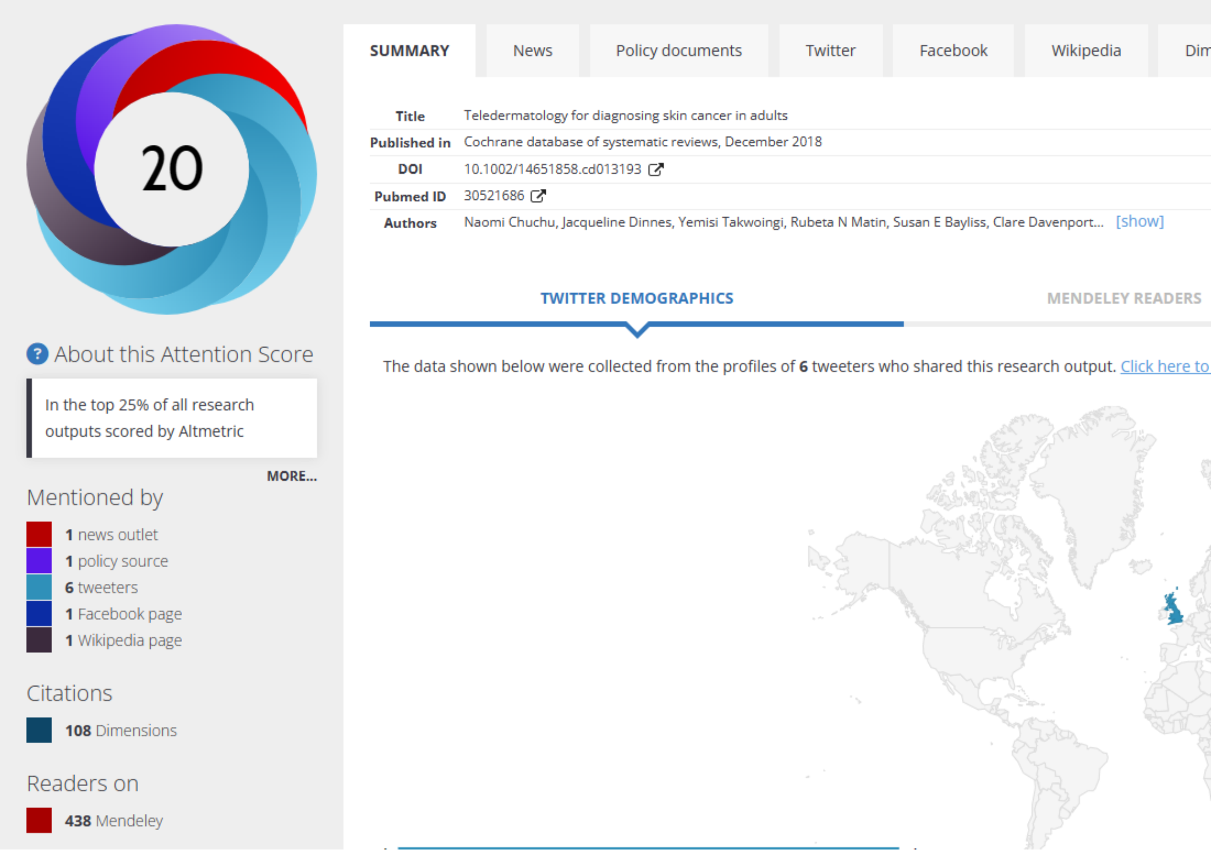

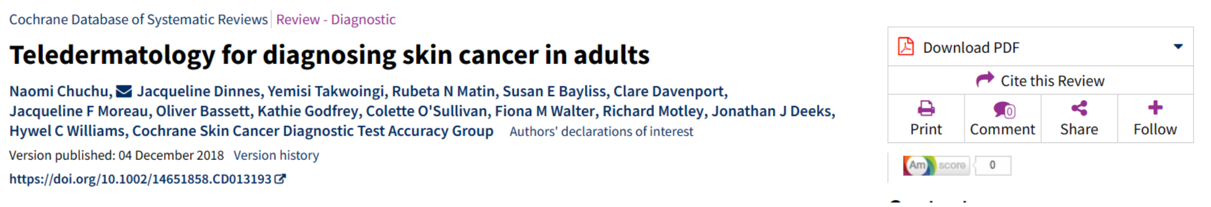

[25] Chuchu, Takwoingi, Dinnes, Matin, Bassett, Moreau, Bayliss, Davenport, Godfrey, O'Connell and et al. (2018) Smartphone applications for triaging adults with skin lesions that are suspicious for melanoma

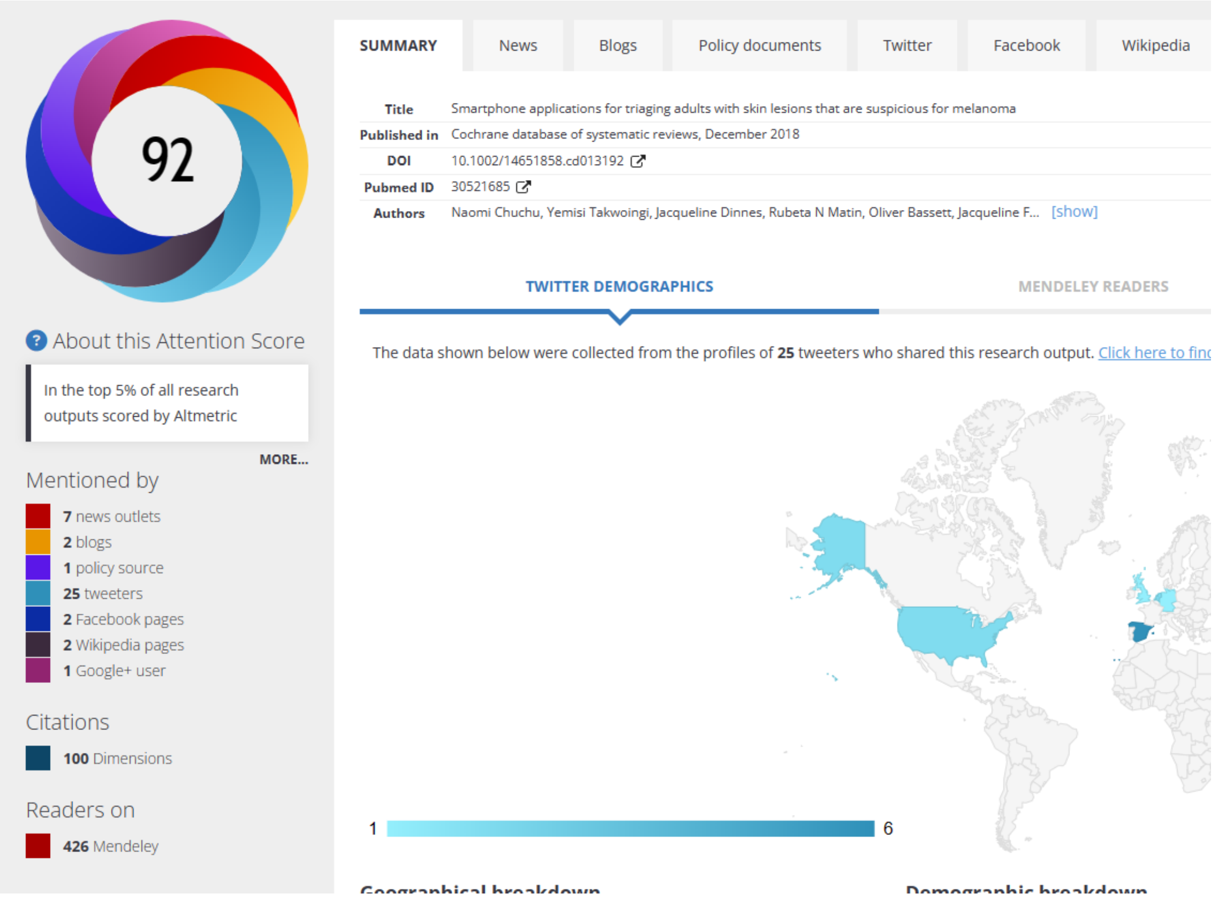

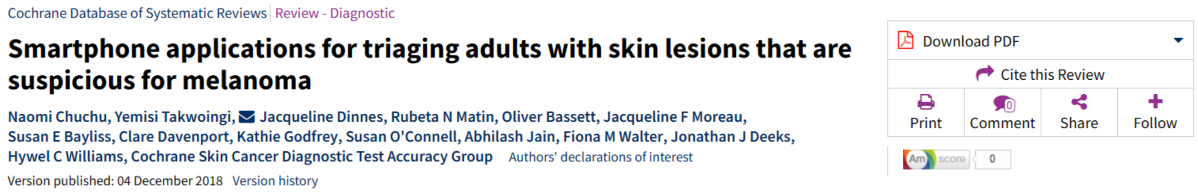

[26] Corry, Neenan, Brabyn, Sheaf and Smith (2019) Telephone interventions, delivered by healthcare professionals, for providing education and psychosocial support for informal caregivers of adults with diagnosed illnesses

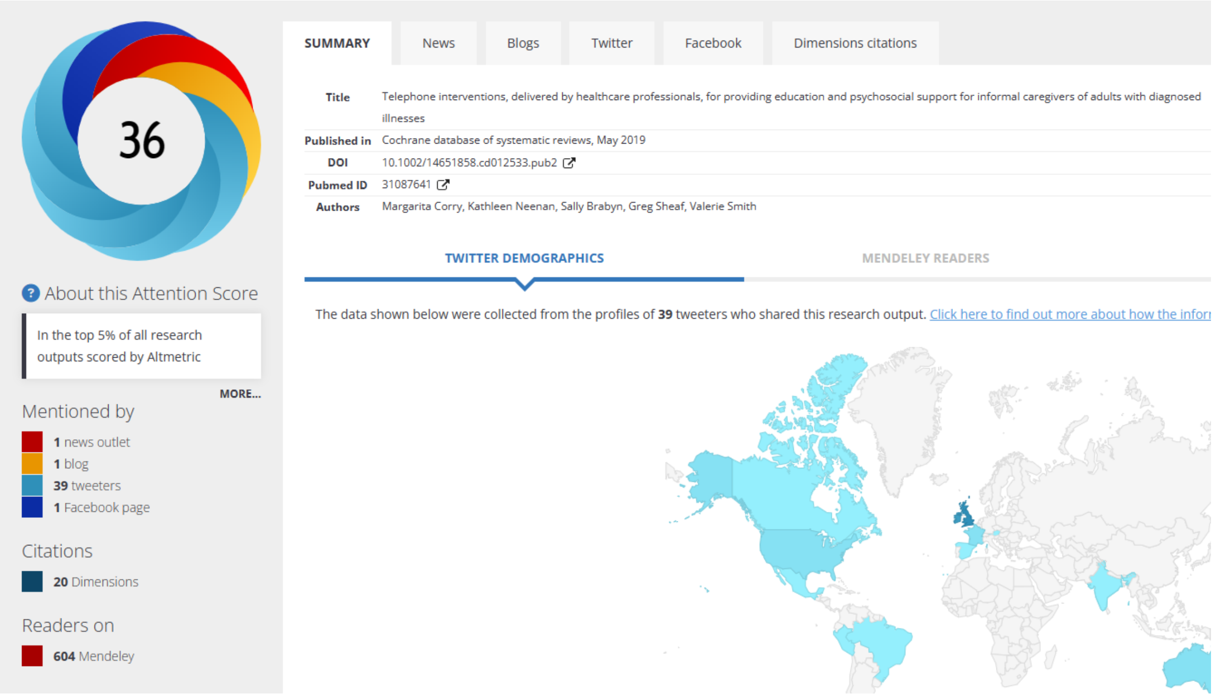

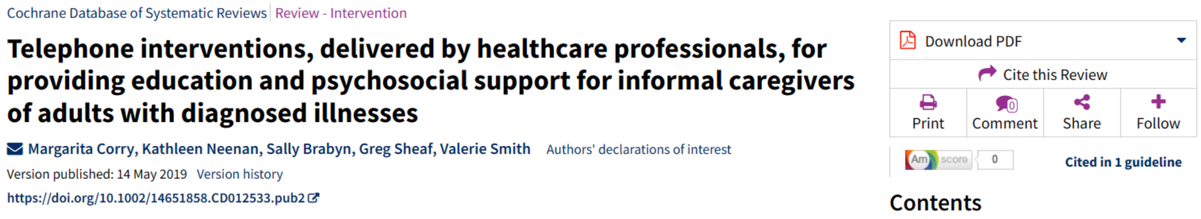

[27] Cox, Dal Corso, Hansen, McDonald, Hill, Zanaboni, Alison, O'Halloran, Macdonald and Holland (2021) Telerehabilitation for chronic respiratory disease

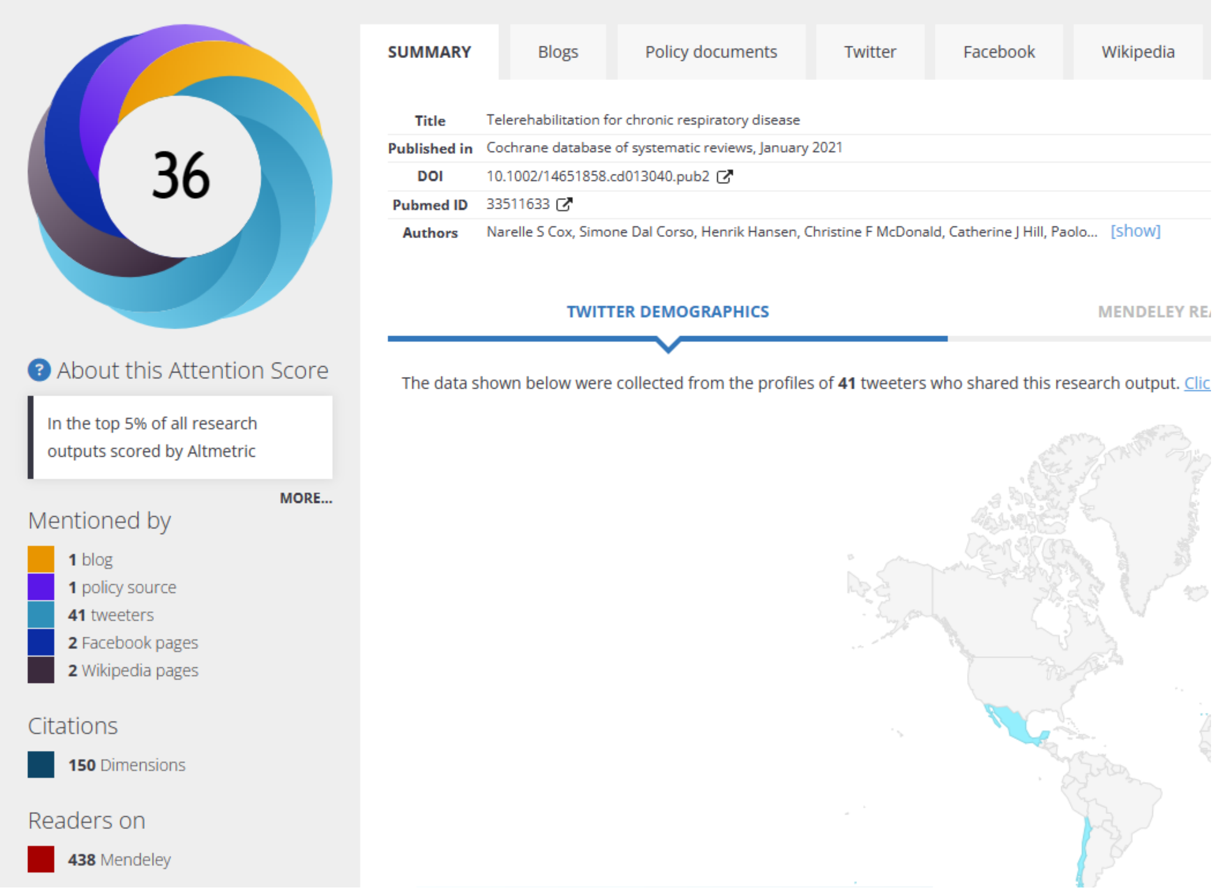

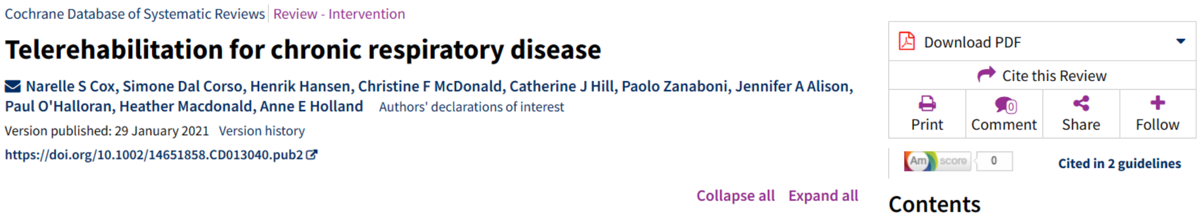

[28] Dale, Caramlau, Lindenmeyer and Williams (2008) Peer support telephone calls for improving health

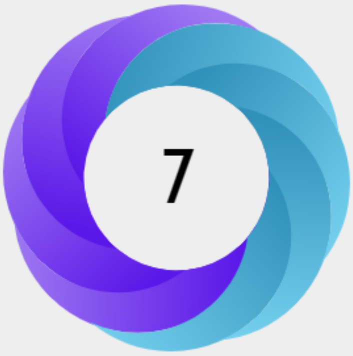

About this Attention Score

In the top 25% of all research outputs scored by Altmetric

MORE...

Mentioned by

2 policy sources
2 tweeters

Citations

109 Dimensions

Readers on

435 Mendeley

SUMMARY | Policy documents | Twitter | Dimensions citations

**Title**	Peer support telephone calls for improving health
**Published in**	Cochrane database of systematic reviews, October 2008
**DOI**	10.1002/14651858.cd006903.pub2
**Pubmed ID**	18843736
**Authors**	Jeremy Dale, Isabela O Caramlau, Antje Lindenmeyer, Susan M Williams

TWITTER DEMOGRAPHICS

The data shown below were collected from the profiles of **2** tweeters who shared this re

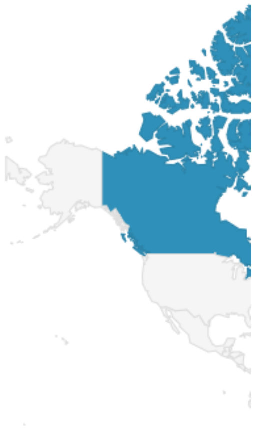

Cochrane Database of Systematic Reviews | Review - Intervention

Peer support telephone calls for improving health

Jeremy Dale, Isabela O Caramlau, Antje Lindenmeyer, Susan M Williams Authors' declarations of interest

Version published: 08 October 2008 Version history

https://doi.org/10.1002/14651858.CD006903.pub2

Unlock the full review

Download PDF

Cite this Review

Print | Comment | Share | Follow

0

Cited in 2 guidelines

Collapse all Expand all

Abstract

[29] Datta, Daruvala and Kumar (2020) Psychological interventions for psychosis in adolescents

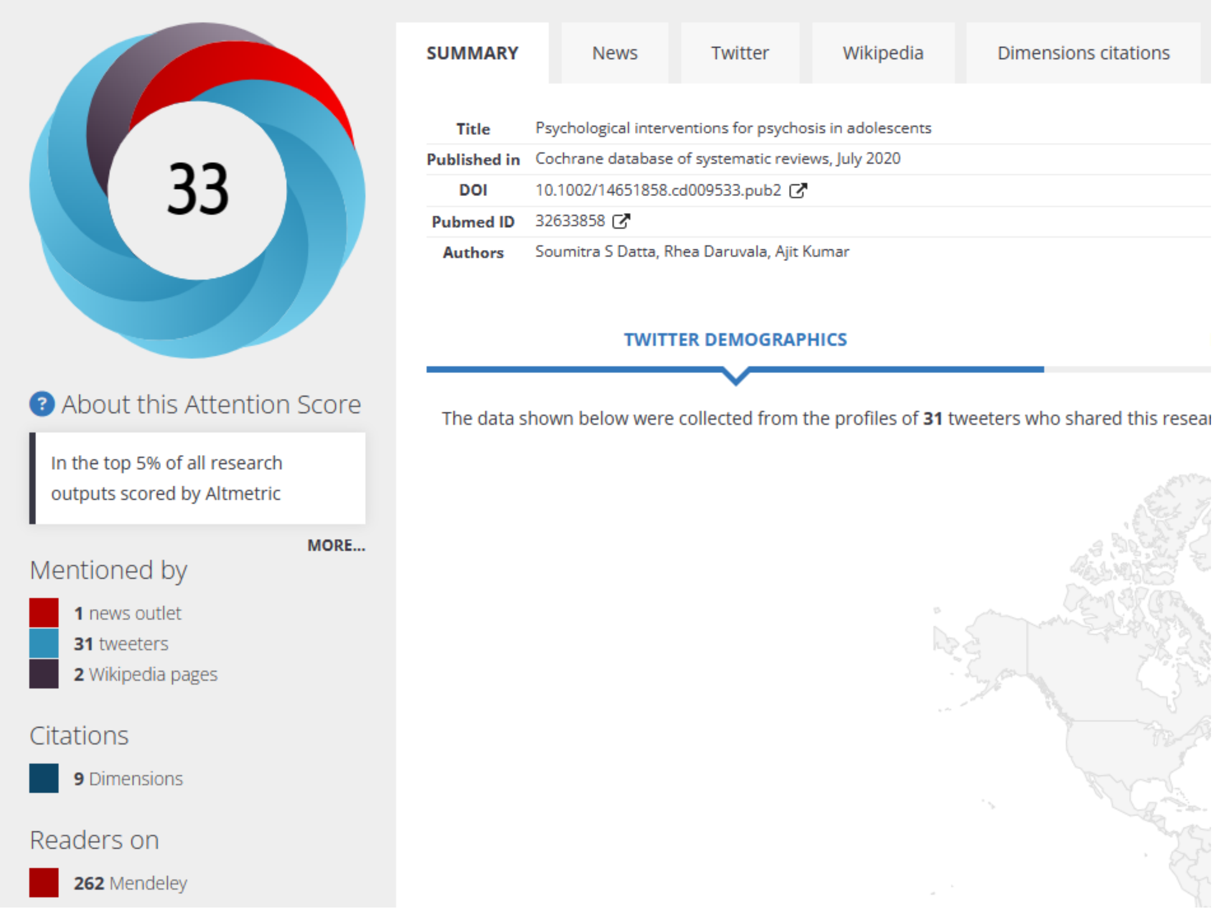

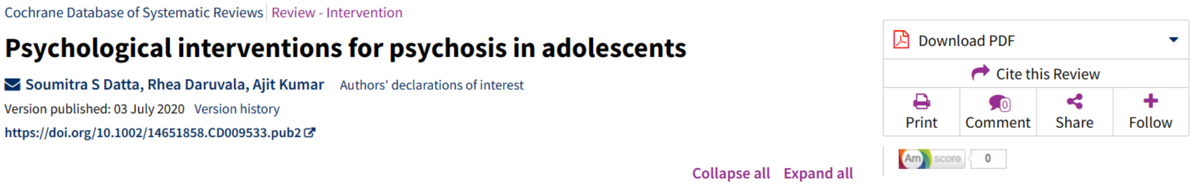

[30] de Jongh, Gurol-Urganci, Vodopivec-Jamsek, Car and Atun (2012) Mobile phone messaging for facilitating self-management of long-term illnesses

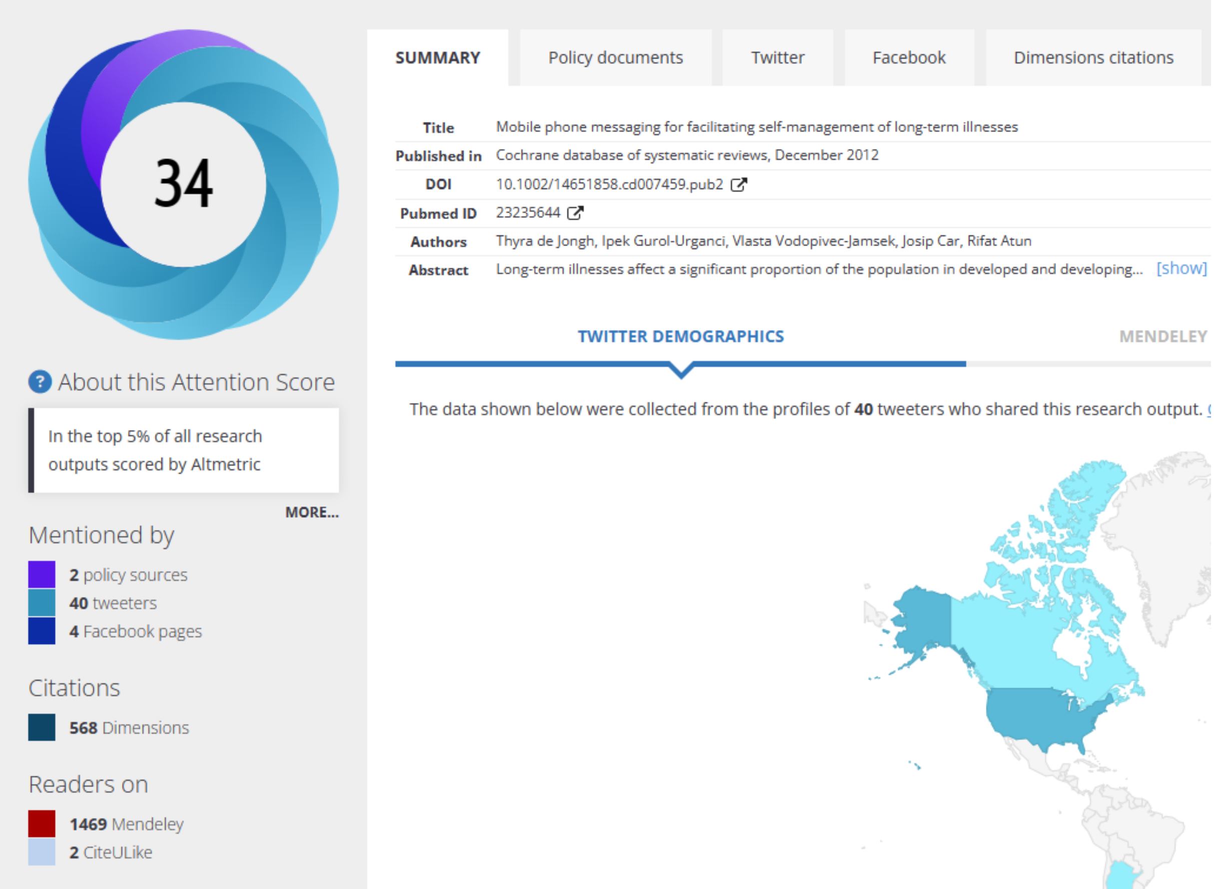

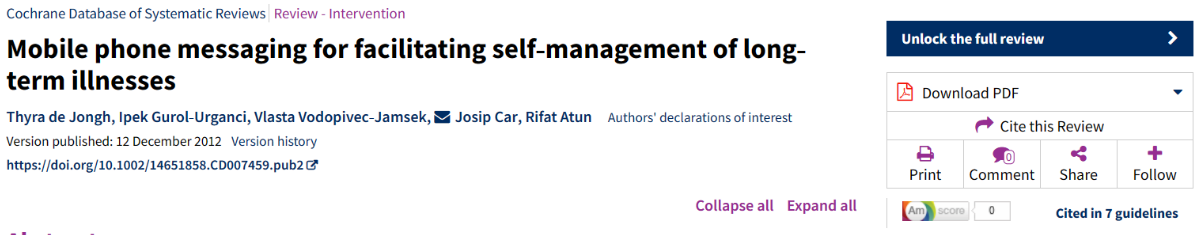

[31] Dennett, Janjua, Stovold, Harrison, McDonnell and Holland (2021) Tailored or adapted interventions for adults with chronic obstructive pulmonary disease and at least one other long-term condition: a mixed methods review

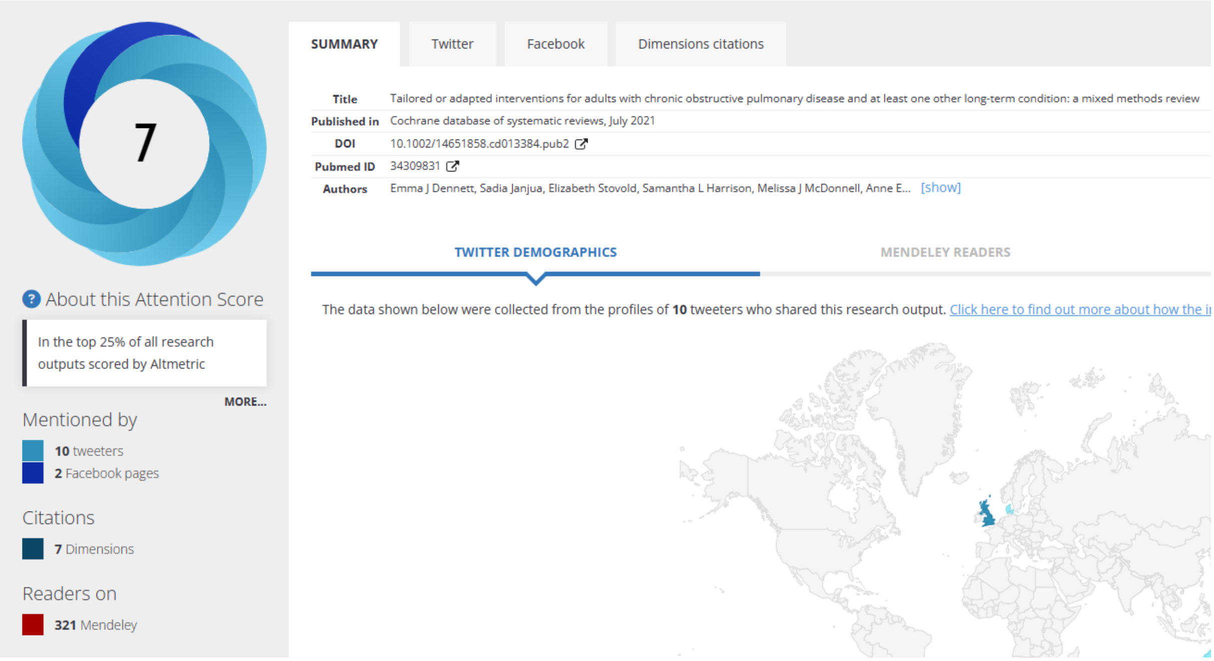

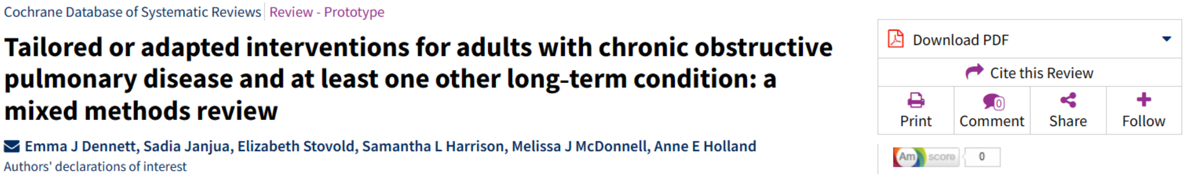

[32] Devi, Singh, Powell, Fulton, Igbinedion and Rees (2015) Internet-based interventions for the secondary prevention of coronary heart disease

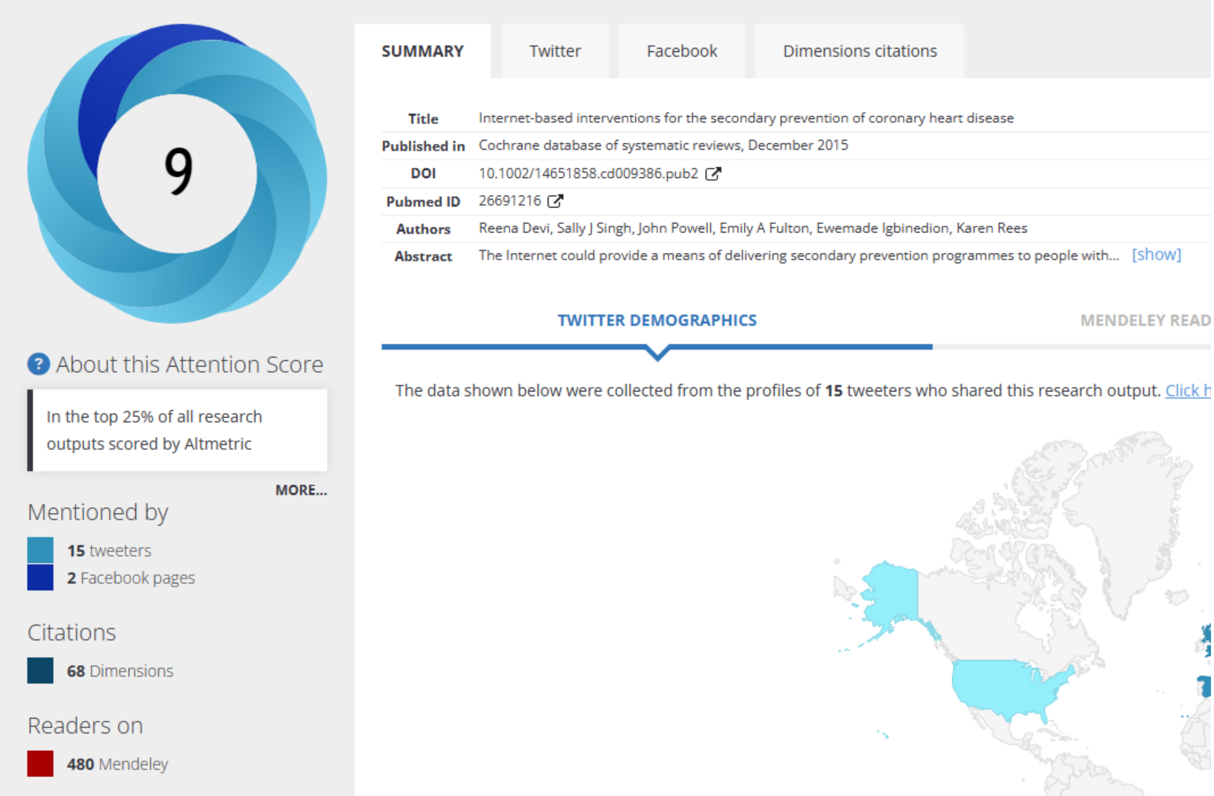
SUMMARY | Twitter | Facebook | Dimensions citations

Title	Internet-based interventions for the secondary prevention of coronary heart disease
Published in	Cochrane database of systematic reviews, December 2015
DOI	10.1002/14651858.cd009386.pub2
Pubmed ID	26691216
Authors	Reena Devi, Sally J Singh, John Powell, Emily A Fulton, Ewemade Igbinedion, Karen Rees
Abstract	The Internet could provide a means of delivering secondary prevention programmes to people with... [show]

TWITTER DEMOGRAPHICS MENDELEY READ

The data shown below were collected from the profiles of **15** tweeters who shared this research output. Click h

9

About this Attention Score

In the top 25% of all research outputs scored by Altmetric

MORE...

Mentioned by

15 tweeters

2 Facebook pages

Citations

68 Dimensions

Readers on

480 Mendeley

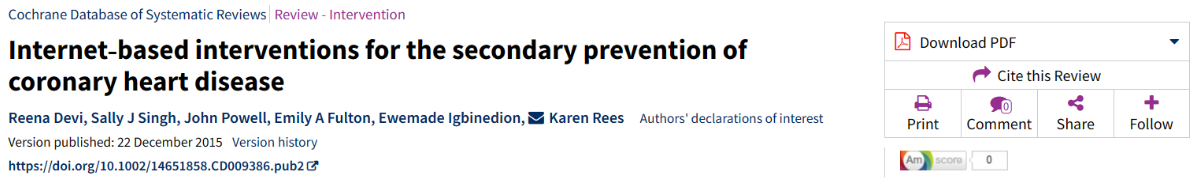
Cochrane Database of Systematic Reviews | Review - Intervention

Internet-based interventions for the secondary prevention of coronary heart disease

Reena Devi, Sally J Singh, John Powell, Emily A Fulton, Ewemade Igbinedion, Karen Rees Authors' declarations of interest

Version published: 22 December 2015 Version history

https://doi.org/10.1002/14651858.CD009386.pub2

Download PDF

Cite this Review

Print | Comment | Share | Follow

score 0

[33] Eccleston, Fisher, Craig, Duggan, Rosser and Keogh (2014) Psychological therapies (Internet-delivered) for the management of chronic pain in adults

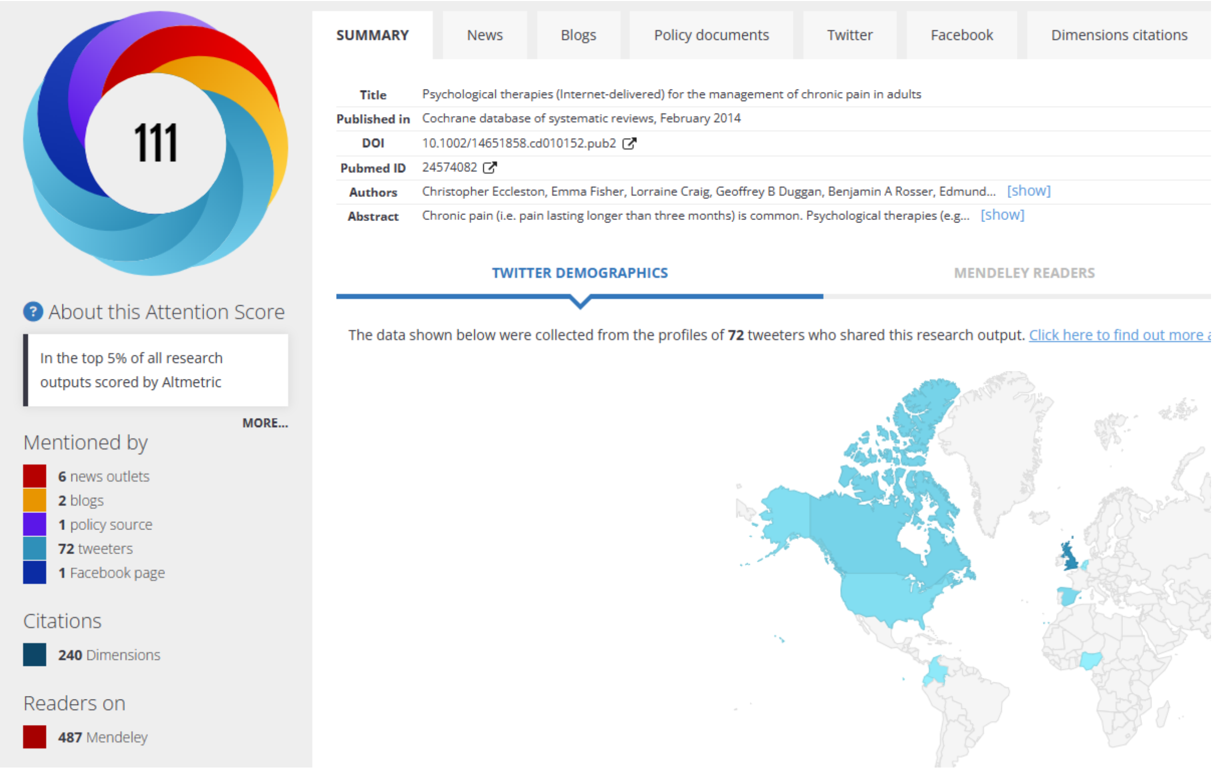

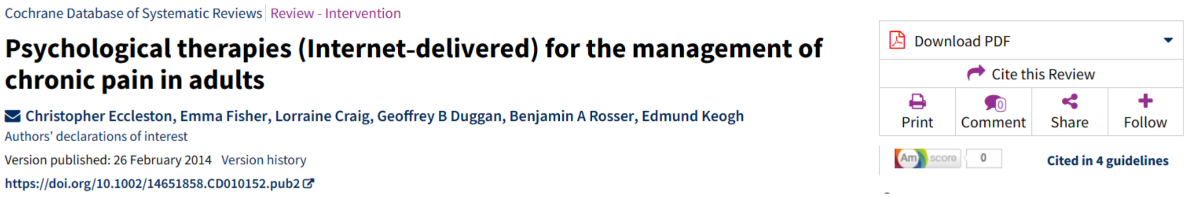

[34] Eccleston, Fisher, Thomas, Hearn, Derry, Stannard, Knaggs and Moore (2017) Interventions for the reduction of prescribed opioid use in chronic non-cancer pain

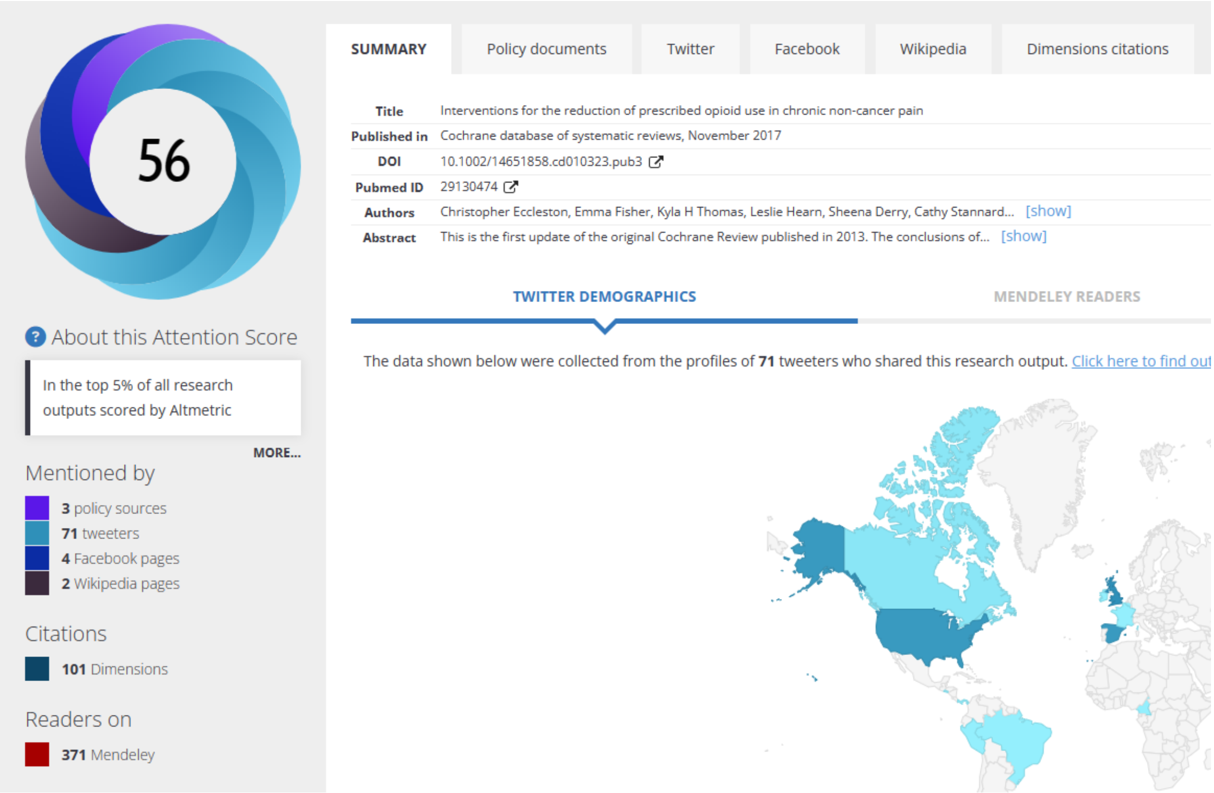

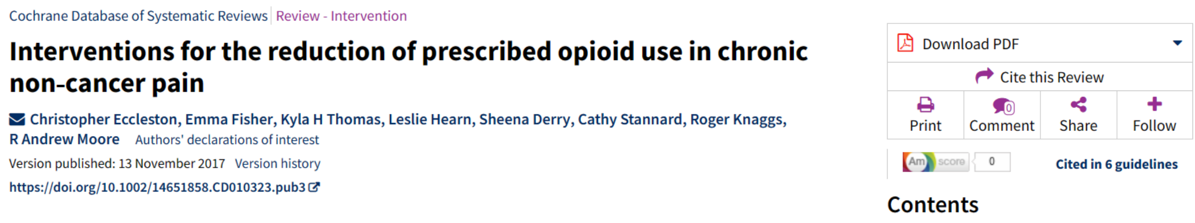

[35] Fiander, McGowan, Grad, Pluye, Hannes, Labrecque, Roberts, Salzwedel, Welch and Tugwell (2015) Interventions to increase the use of electronic health information by healthcare practitioners to improve clinical practice and patient outcomes

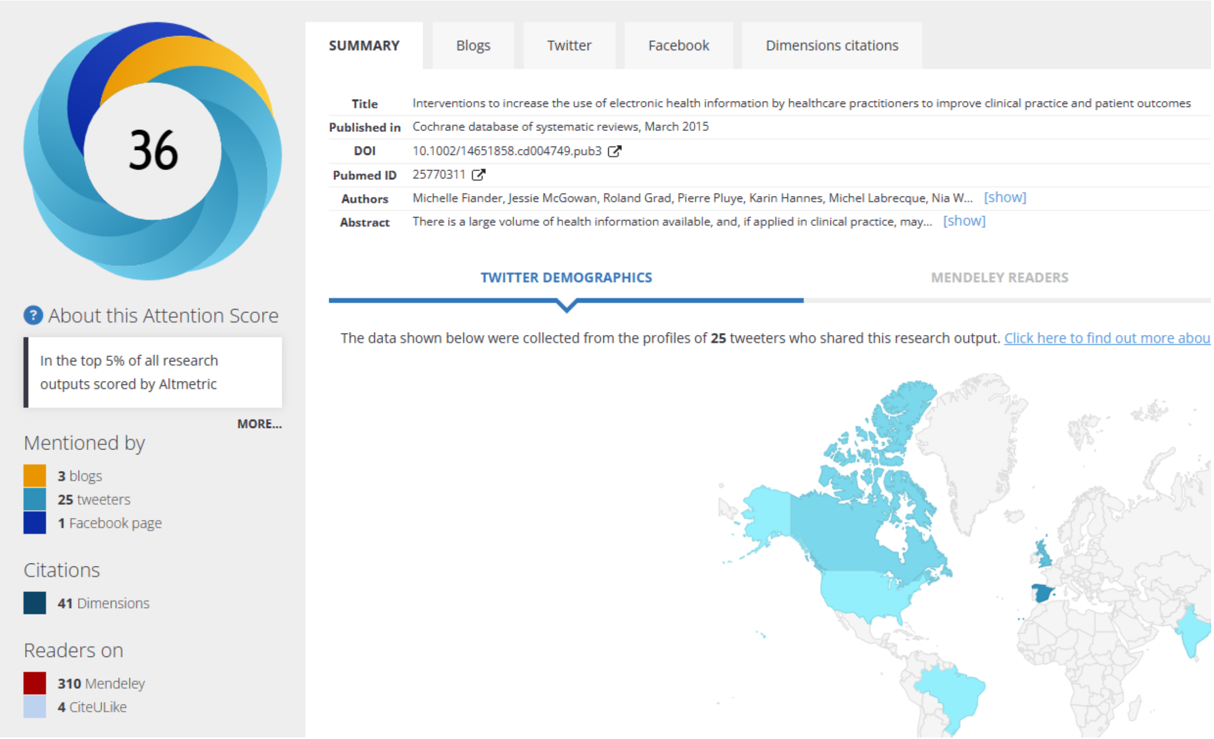

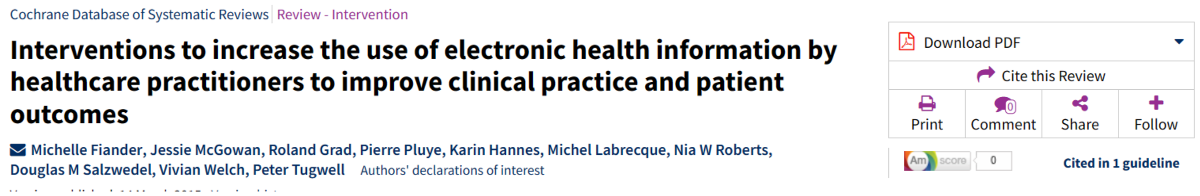

[36] Fisher, Law, Dudeney, Eccleston and Palermo (2019) Psychological therapies (remotely delivered) for the management of chronic and recurrent pain in children and adolescents

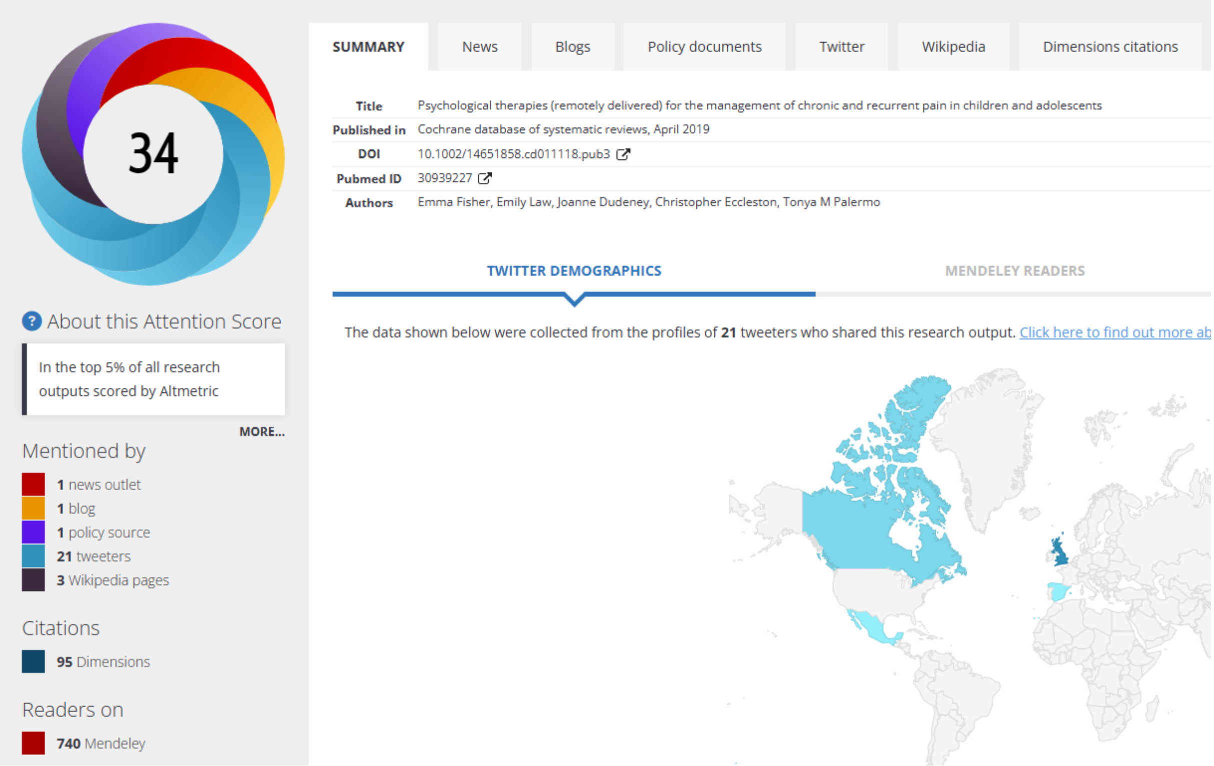

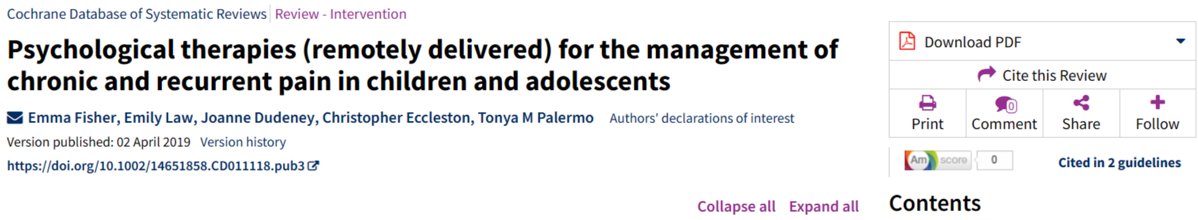

[37] Fleming, Strydom, Katsaros, MacDonald, Curatolo, Fudalej and Pandis (2016) Non-pharmacological interventions for alleviating pain during orthodontic treatment

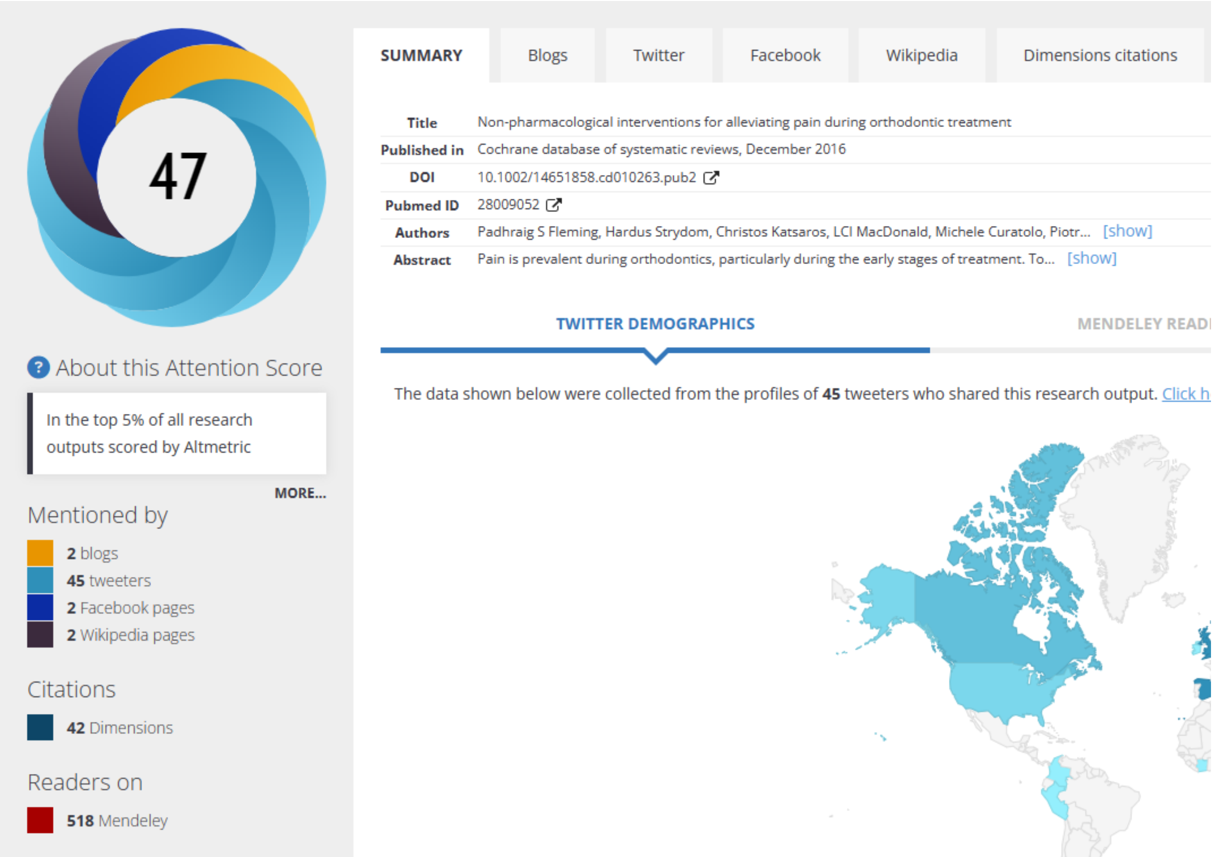

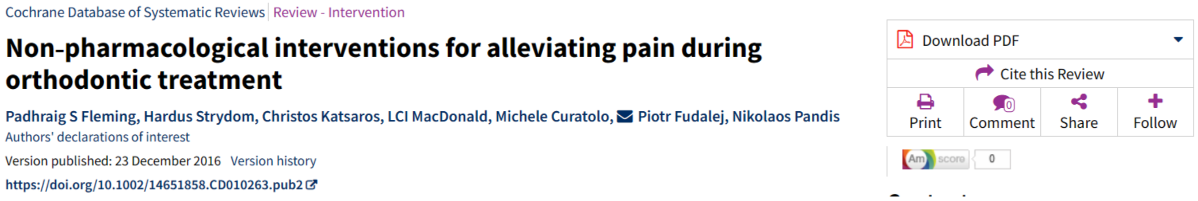

[38] Flodgren, Rachas, Farmer, Inzitari and Shepperd (2015) Interactive telemedicine: effects on professional practice and health care outcomes

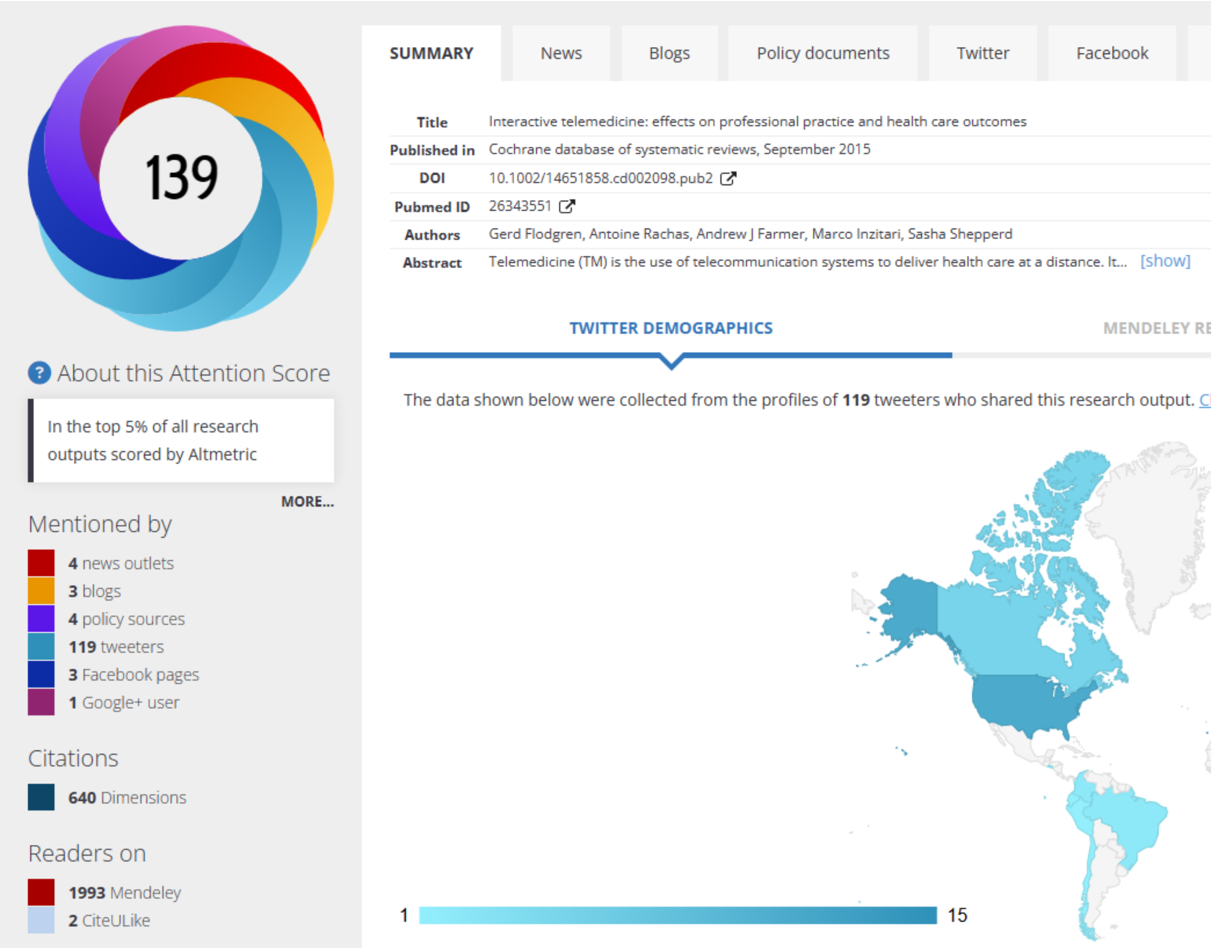

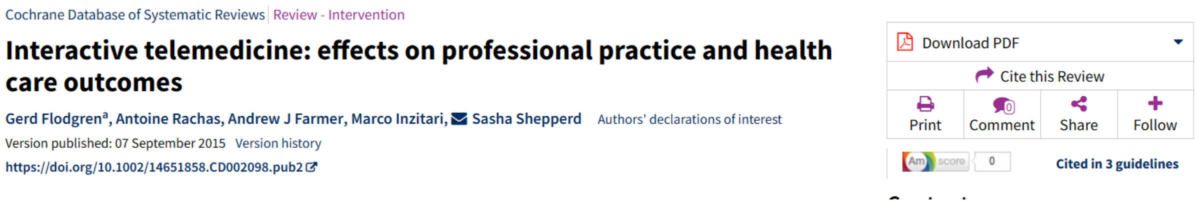

[39] Foster, Richards, Thorogood and Hillsdon (2013) Remote and web 2.0 interventions for promoting physical activity

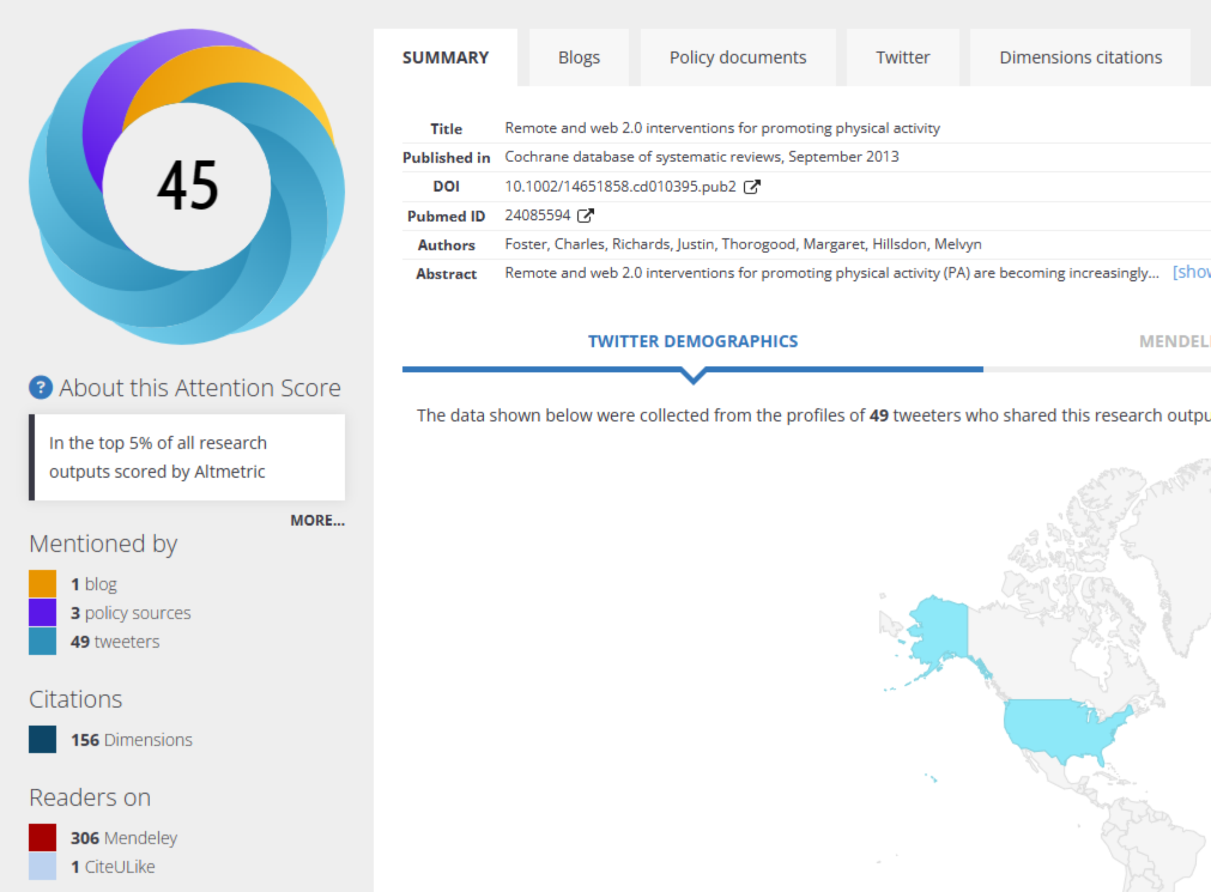

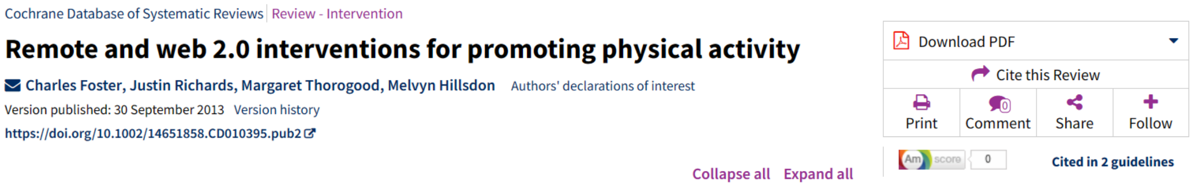

[40] Galaal, Bryant, Deane, Al-Khaduri and Lopes (2011) Interventions for reducing anxiety in women undergoing colposcopy

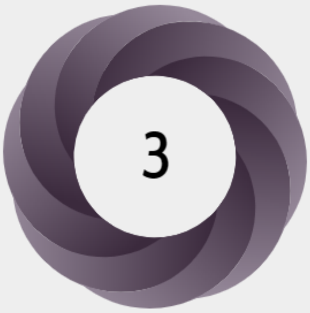

About this Attention Score

Average Attention Score compared to outputs of the same age

MORE...

Mentioned by

2 Wikipedia pages

Citations

69 Dimensions

Readers on

283 Mendeley

SUMMARY | Wikipedia | Dimensions citations

**Title**	Interventions for reducing anxiety in women undergoing colposcopy
**Published in**	Cochrane database of systematic reviews, December 2011
**DOI**	10.1002/14651858.cd006013.pub3
**Pubmed ID**	22161395
**Authors**	Khadra Galaal, Andrew Bryant, Katherine HO Deane, Maha Al-Khaduri, Alberto D Lopes
**Abstract**	Prior to the development of cervical cancer abnormal cervical cells can be detected on a cervical... [show]

MENDELEY READERS

The data shown below were compiled from readership statistics for **283** Mendeley readers of this rese

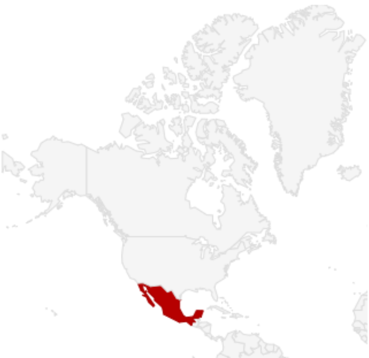

Cochrane Database of Systematic Reviews | Review - Intervention

Interventions for reducing anxiety in women undergoing colposcopy

Khadra Galaal, Andrew Bryant, Katherine HO Deane, Maha Al-Khaduri, Alberto D Lopes Authors' declarations of interest

Version published: 07 December 2011 Version history

https://doi.org/10.1002/14651858.CD006013.pub3

Collapse all Expand all

Abstract

Unlock the full review

Download PDF

Cite this Review

Print | Comment | Share | Follow

score 0

Cited in 1 guideline

[41] Gates, Rutjes, Di Nisio, Karim, Chong, March, Martínez and Vernooij (2019) Computerised cognitive training for maintaining cognitive function in cognitively healthy people in midlife

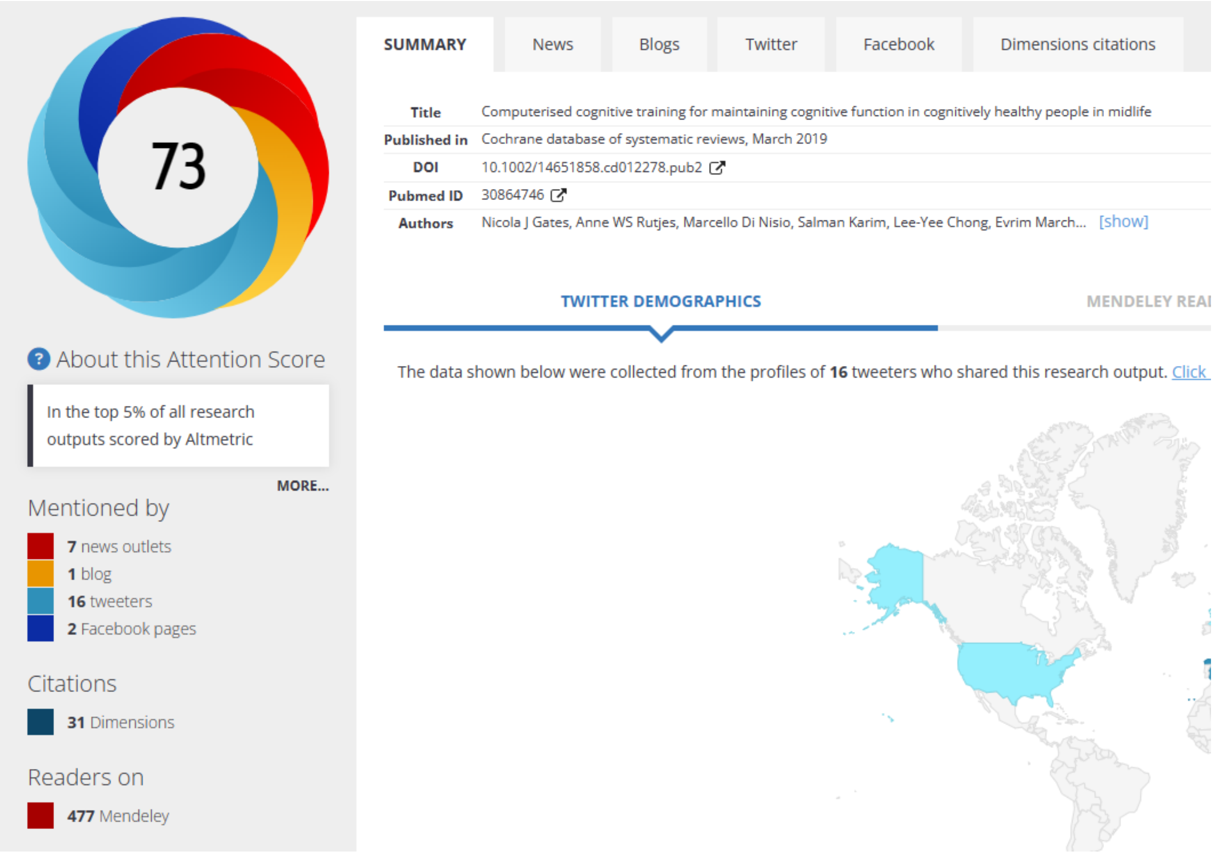

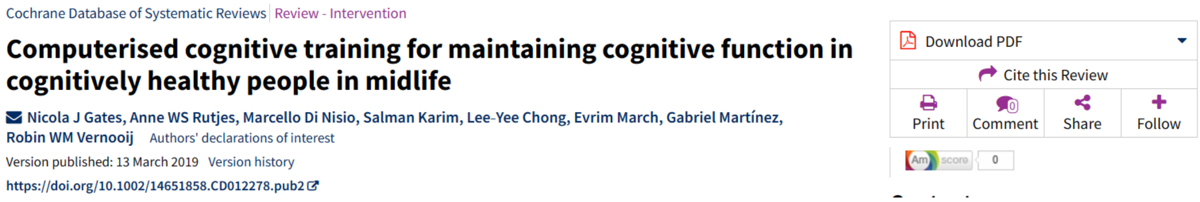

[42] Gates, Rutjes, Di Nisio, Karim, Chong, March, Martínez and Vernooij (2020) Computerised cognitive training for 12 or more weeks for maintaining cognitive function in cognitively healthy people in late life

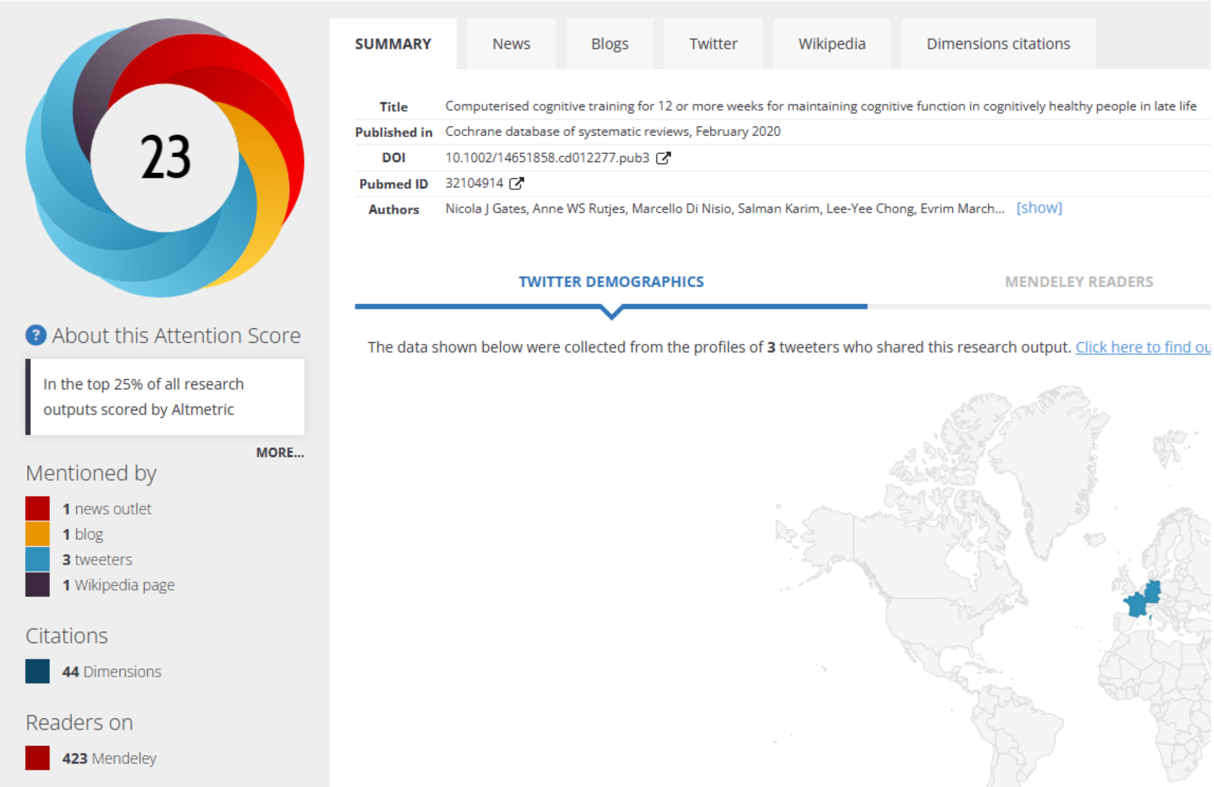
23

About this Attention Score

In the top 25% of all research outputs scored by Altmetric

MORE...

Mentioned by

1 news outlet
1 blog
3 tweeters
1 Wikipedia page

Citations

44 Dimensions

Readers on

423 Mendeley

SUMMARY | News | Blogs | Twitter | Wikipedia | Dimensions citations

Title	Computerised cognitive training for 12 or more weeks for maintaining cognitive function in cognitively healthy people in late life
Published in	Cochrane database of systematic reviews, February 2020
DOI	10.1002/14651858.cd012277.pub3
Pubmed ID	32104914
Authors	Nicola J Gates, Anne WS Rutjes, Marcello Di Nisio, Salman Karim, Lee-Yee Chong, Evrim March... [show]

TWITTER DEMOGRAPHICS | MENDELEY READERS

The data shown below were collected from the profiles of 3 tweeters who shared this research output. Click here to find ou

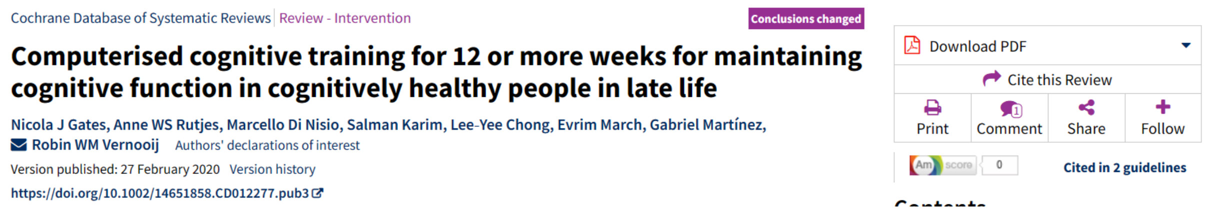
Cochrane Database of Systematic Reviews | Review - Intervention

Conclusions changed

Computerised cognitive training for 12 or more weeks for maintaining cognitive function in cognitively healthy people in late life

Nicola J Gates, Anne WS Rutjes, Marcello Di Nisio, Salman Karim, Lee-Yee Chong, Evrim March, Gabriel Martínez, Robin WM Vernooij   Authors' declarations of interest

Version published: 27 February 2020   Version history

https://doi.org/10.1002/14651858.CD012277.pub3

Download PDF

Cite this Review

Print | Comment | Share | Follow

Am score 0

Cited in 2 guidelines

[43] Gates, Vernooij, Di Nisio, Karim, March, Martínez and Rutjes (2019) Computerised cognitive training for preventing dementia in people with mild cognitive impairment

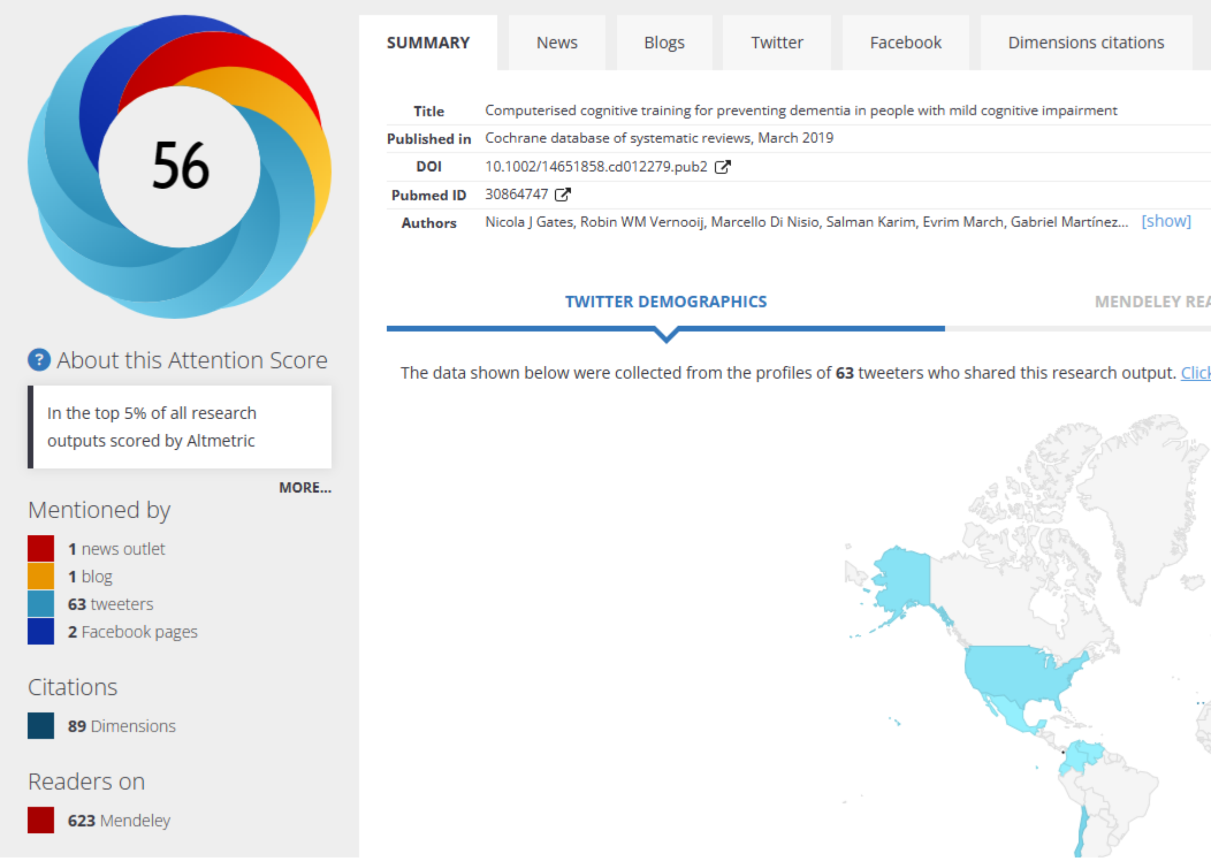

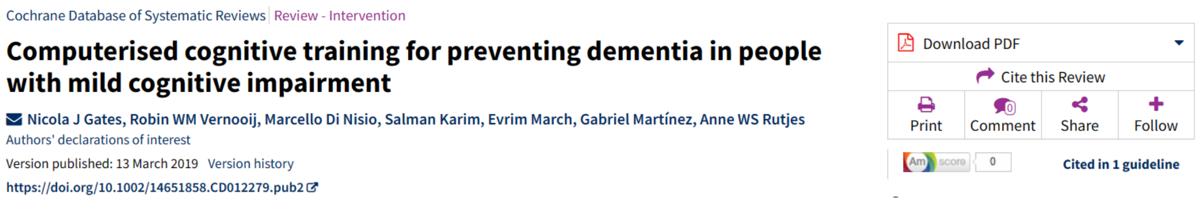

[44] Gavine, Shinwell, Buchanan, Farre, Wade, Lynn, Marshall, Cumming, Dare and McFadden (2022) Support for healthy breastfeeding mothers with healthy term babies

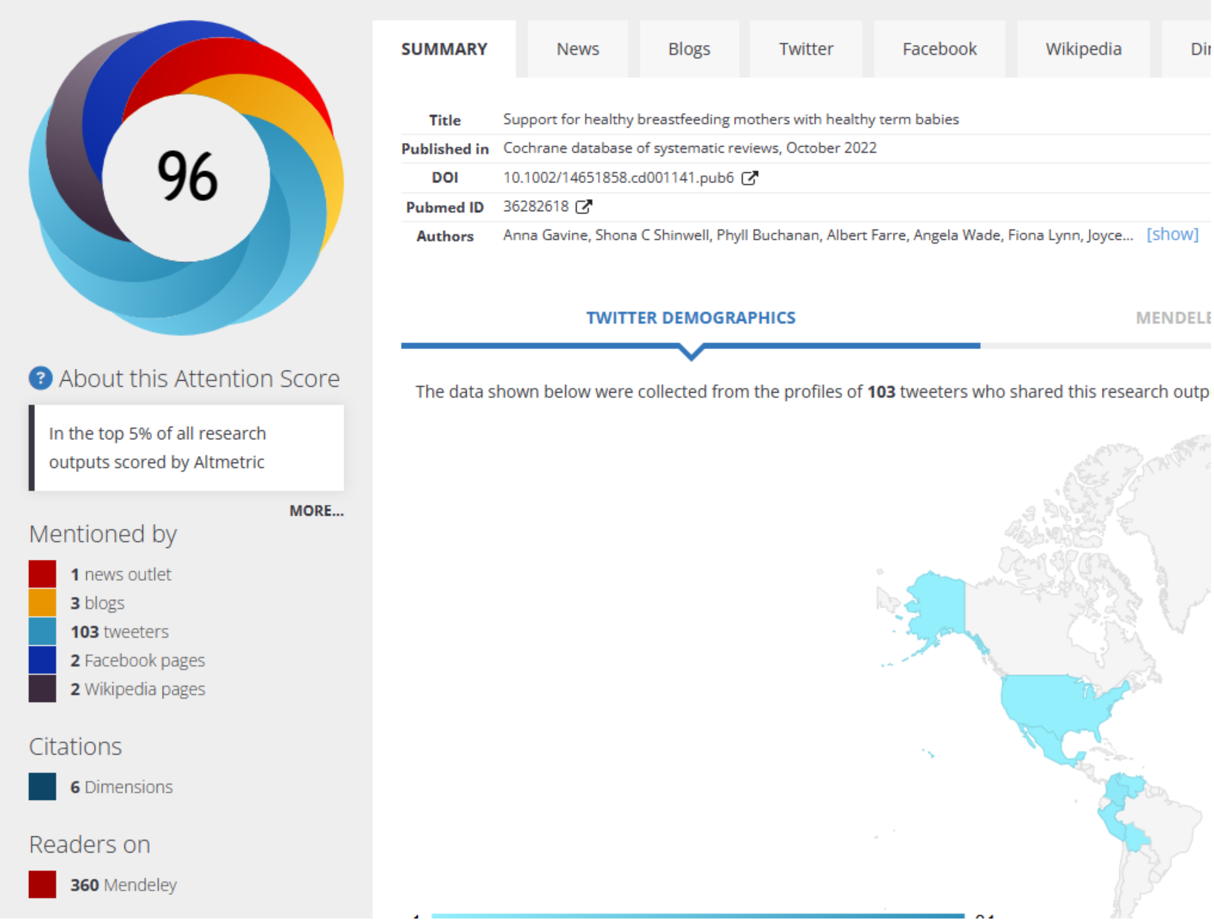

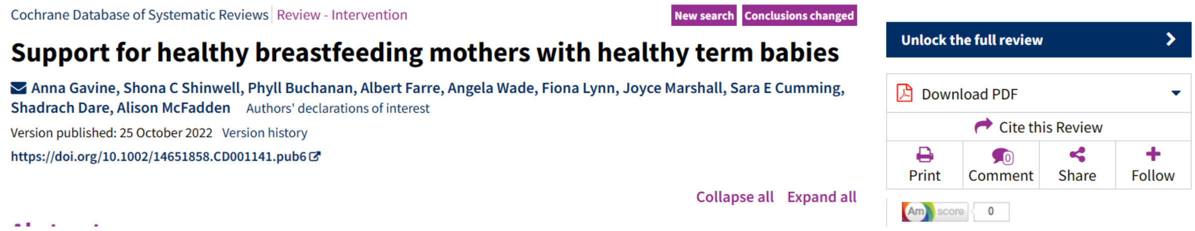

[45] Gentry, van-Velthoven, Tudor Car and Car (2013) Telephone delivered interventions for reducing morbidity and mortality in people with HIV infection

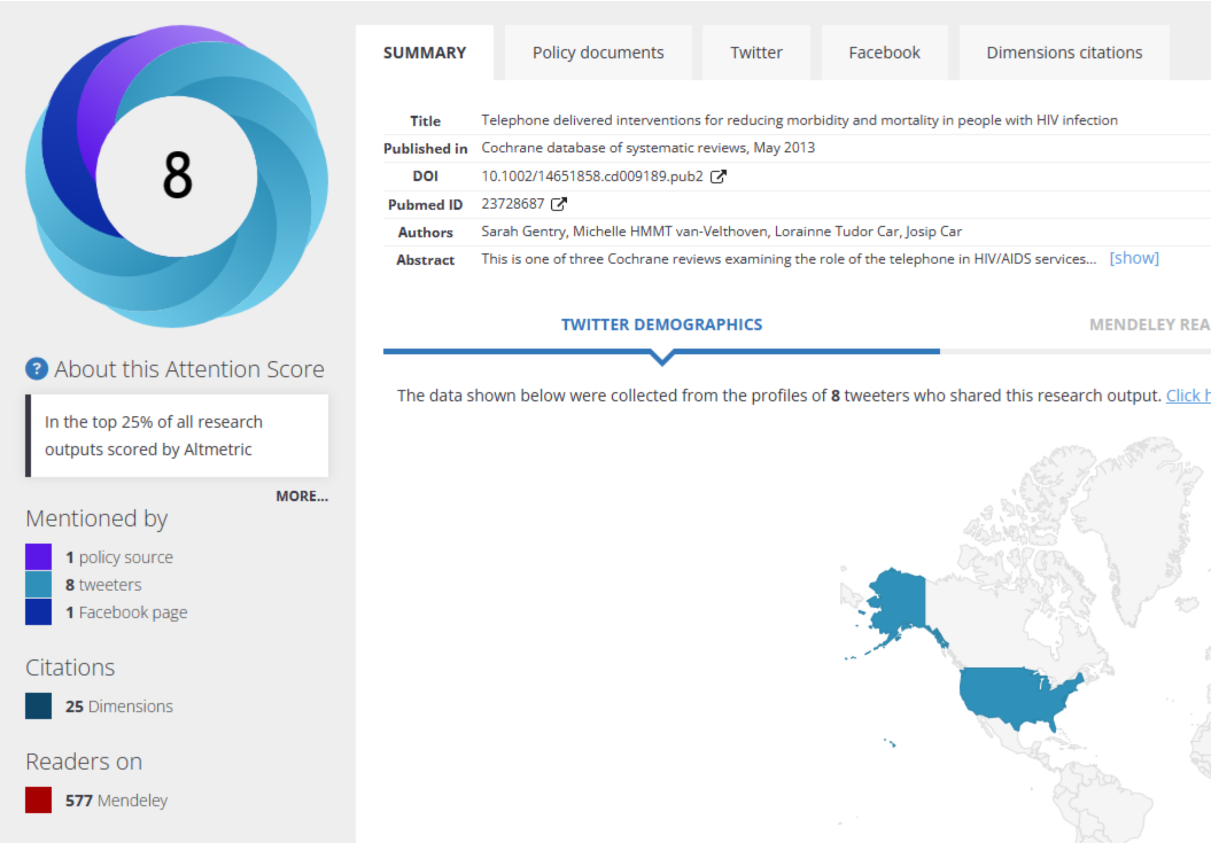

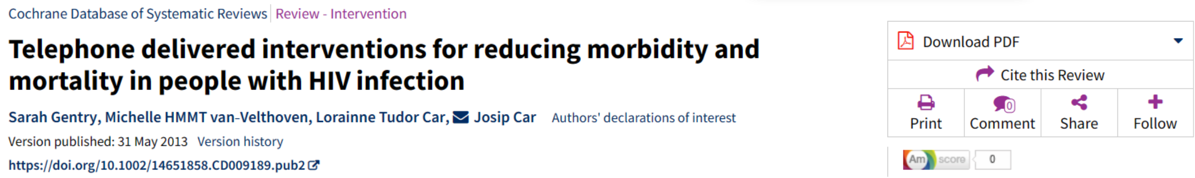

[46] Gillaizeau, Chan, Trinquart, Colombet, Walton, Rège-Walther, Burnand and Durieux (2013) Computerized advice on drug dosage to improve prescribing practice

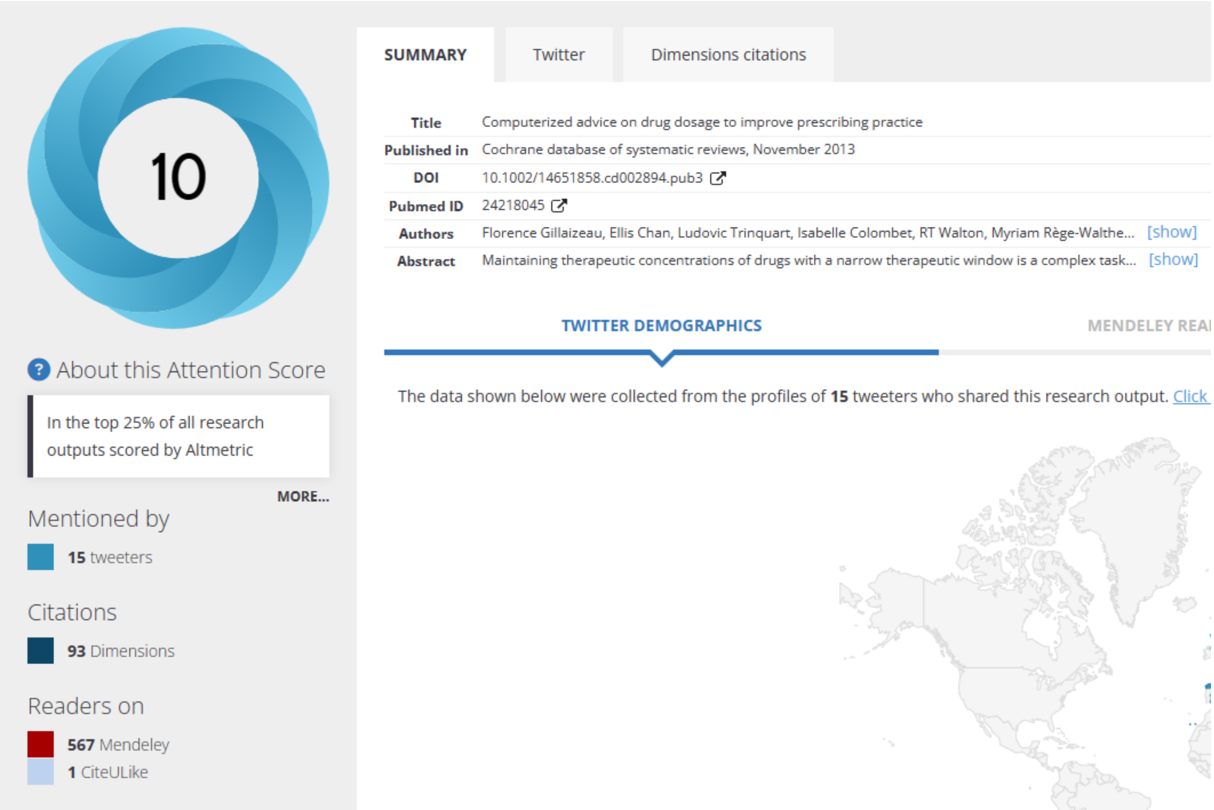

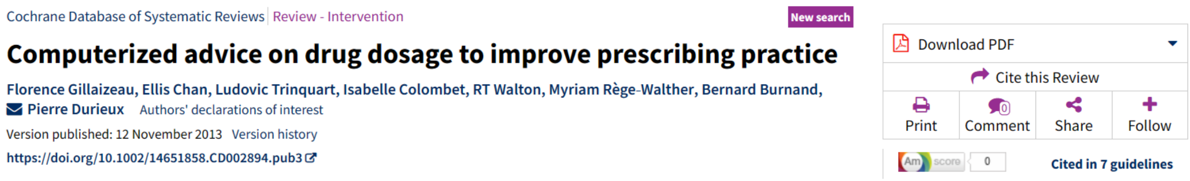

[47] Gonçalves-Bradley, J Maria, Ricci-Cabello, Villanueva, Fønhus, Glenton, Lewin, Henschke, Buckley, Mehl and et al. (2020) Mobile technologies to support healthcare provider to healthcare provider communication and management of care

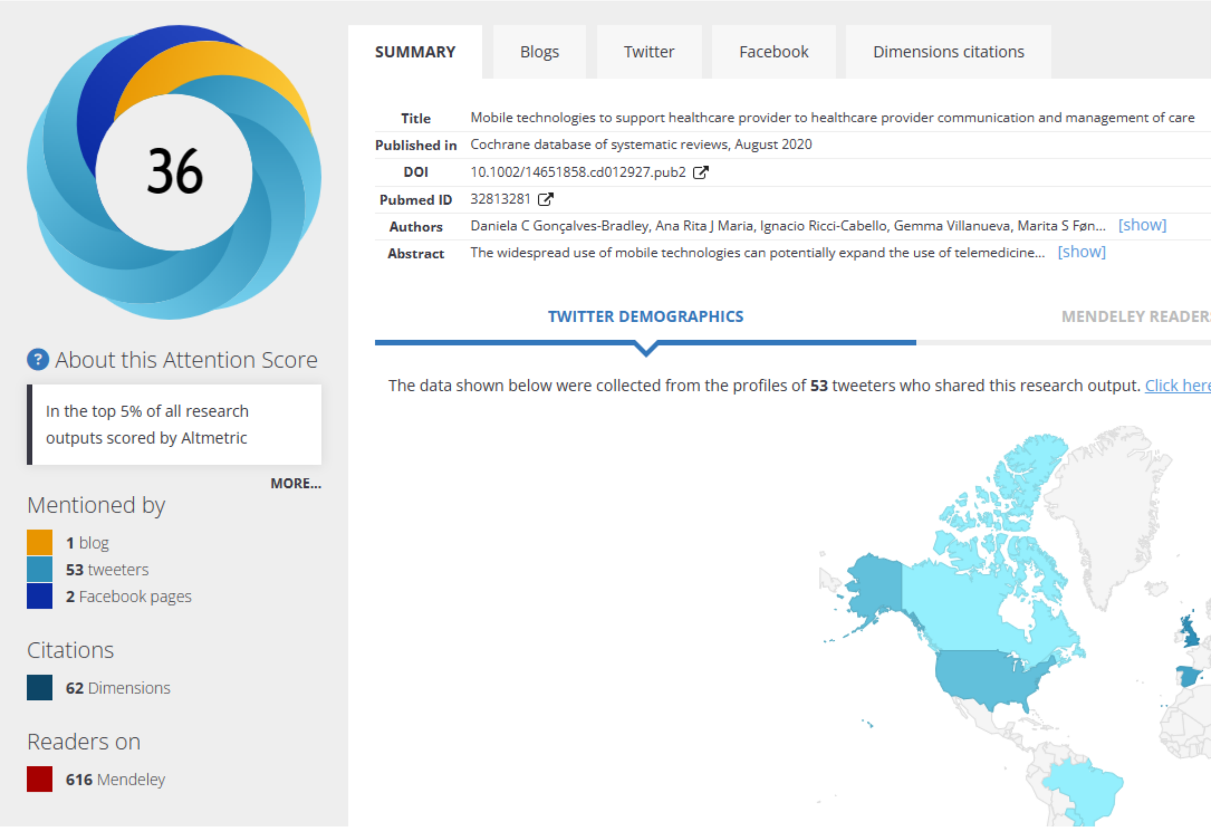

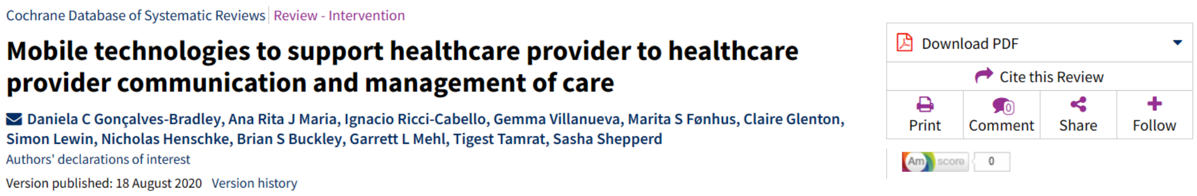

[48] González-Fraile, Ballesteros, Rueda, Santos-Zorrozúa, Solà and McCleery (2021) Remotely delivered information, training and support for informal caregivers of people with dementia

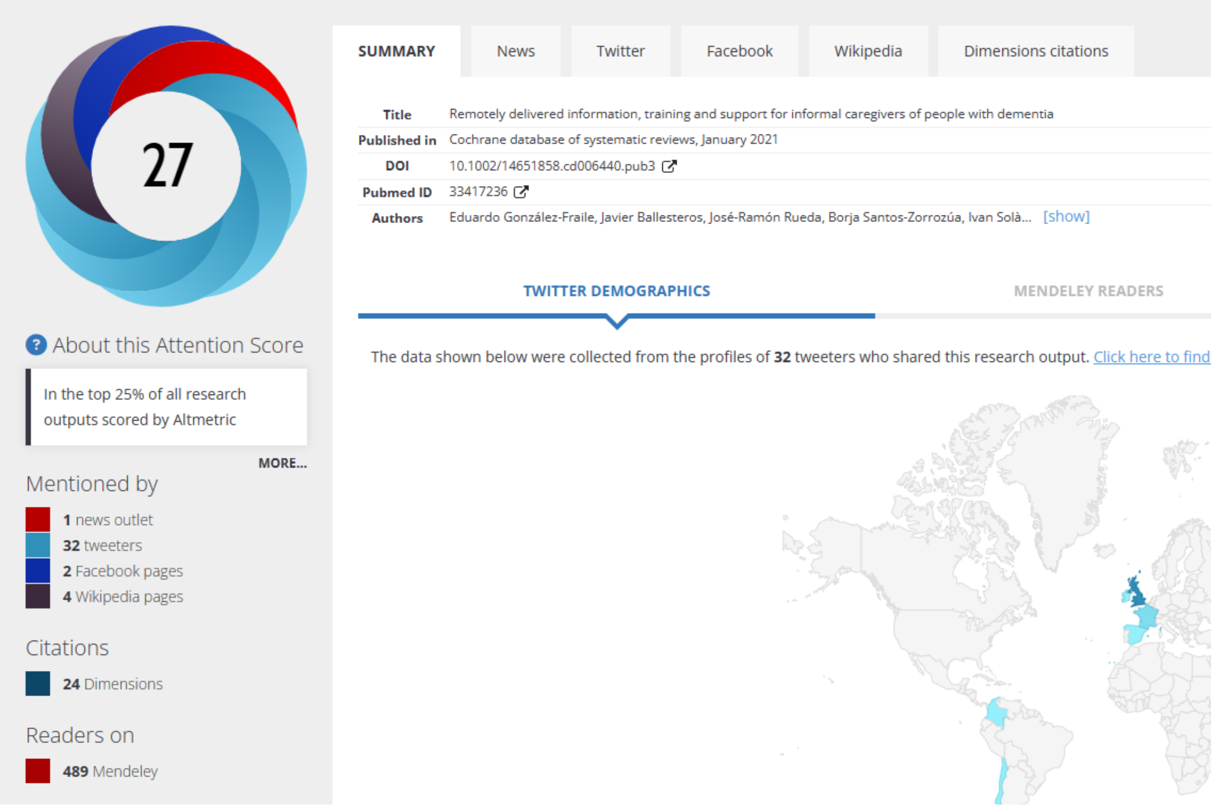

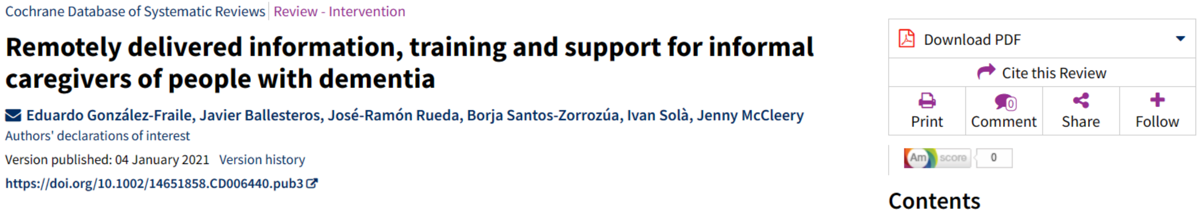

[49] Gordon, Sinopoulou, Lakunina, Gjuladin-Hellon, Bracewell and Akobeng (2023) Remote care through telehealth for people with inflammatory bowel disease

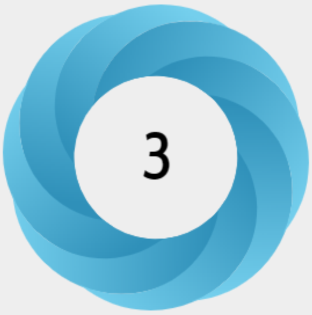

About this Attention Score

Above-average Attention Score compared to outputs of the same age (58th percentile)

Mentioned by

4 tweeters

SUMMARY   Twitter

Title	Remote care through telehealth for people with inflammatory bowel disease
Published in	Cochrane database of systematic reviews, May 2023
DOI	10.1002/14651858.cd014821.pub2
Authors	Morris Gordon, Vassiliki Sinopoulou, Svetlana Lakunina, Teuta Gjuladin-Hellon, Kelly Bracewell... [show]

TWITTER DEMOGRAPHICS

The data shown below were collected from the profiles of **4** tweeters who shared this research output. Click

Cochrane Database of Systematic Reviews | Review - Intervention

Remote care through telehealth for people with inflammatory bowel disease

Morris Gordon, Vassiliki Sinopoulou, Svetlana Lakunina, Teuta Gjuladin-Hellon, Kelly Bracewell, Anthony K Akobeng

Authors' declarations of interest

Version published: 04 May 2023 Version history

https://doi.org/10.1002/14651858.CD014821.pub2

Download PDF

Cite this Review

Print | Comment | Share | Follow

score 0

[50] Goyder, Atherton, Car, Heneghan and Car (2015) Email for clinical communication between healthcare professionals

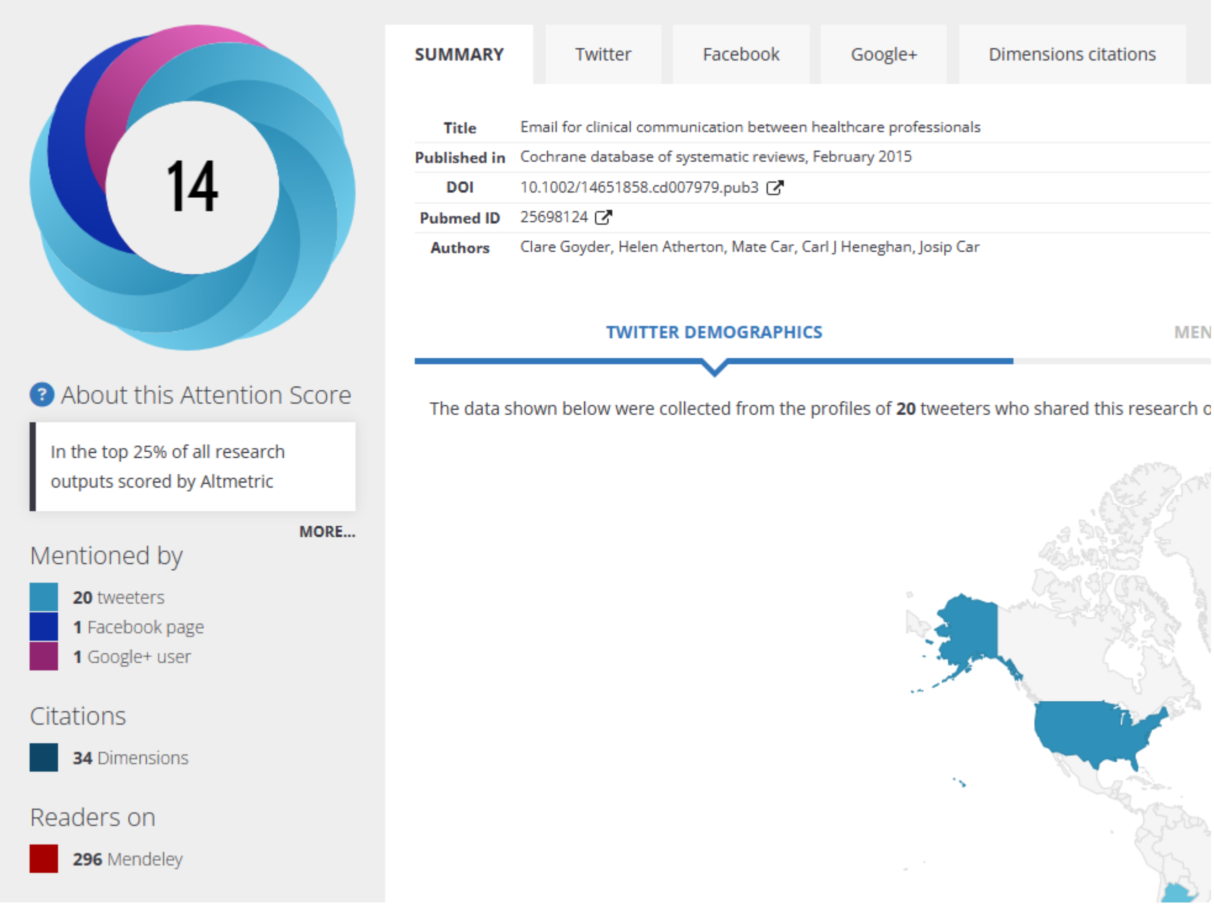

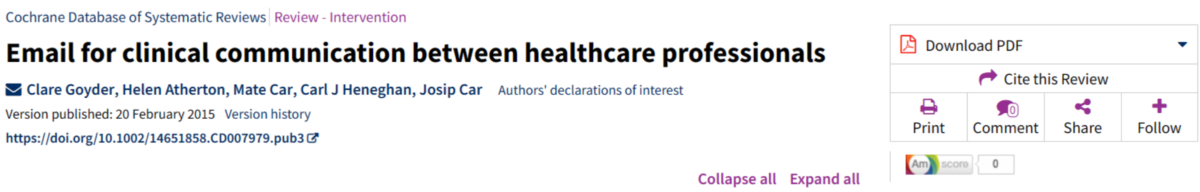

[51] Gurol-Urganci, de Jongh, Vodopivec-Jamsek, Atun and Car (2013) Mobile phone messaging reminders for attendance at healthcare appointments

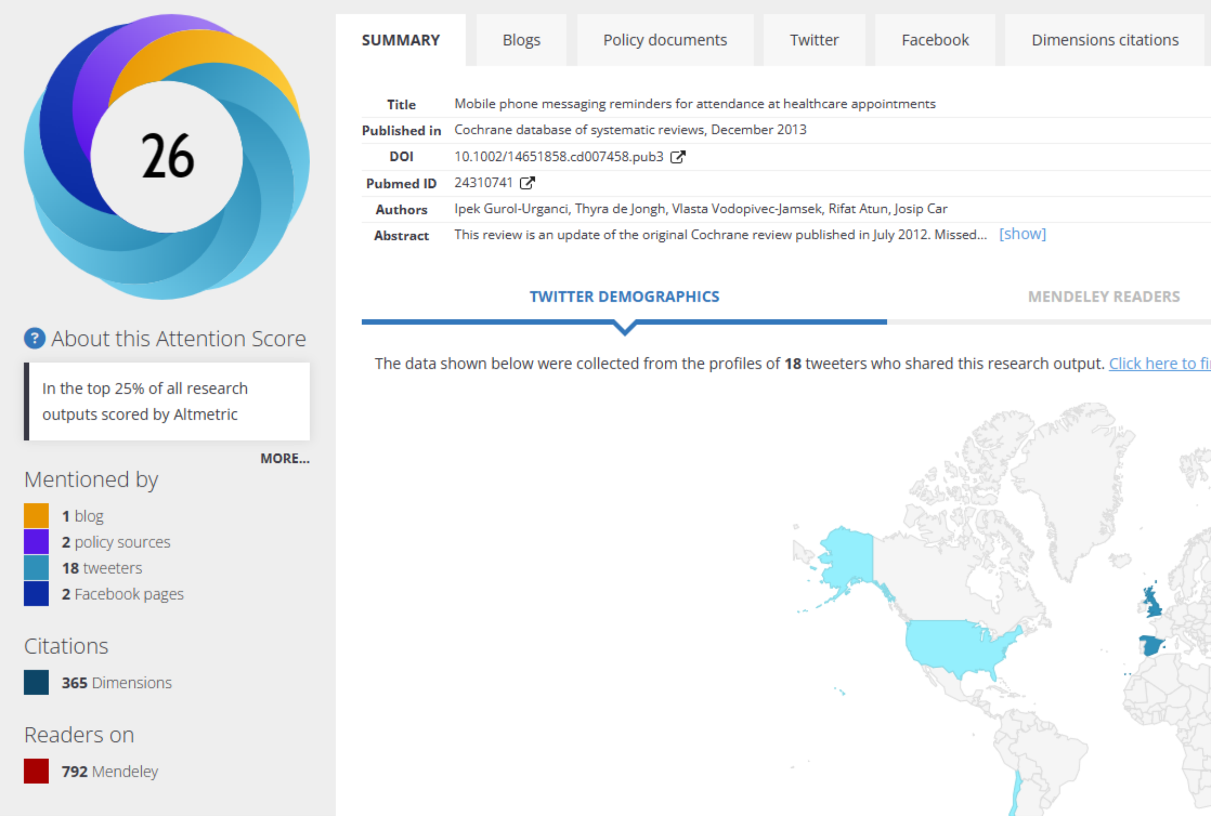

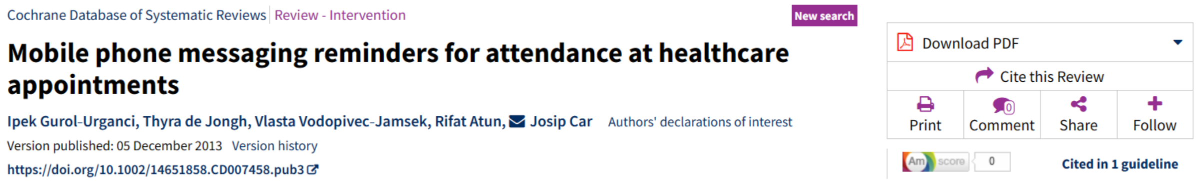

[52] Gurol-Urganci, de Jongh, Vodopivec-Jamsek, Car and Atun (2012) Mobile phone messaging for communicating results of medical investigations

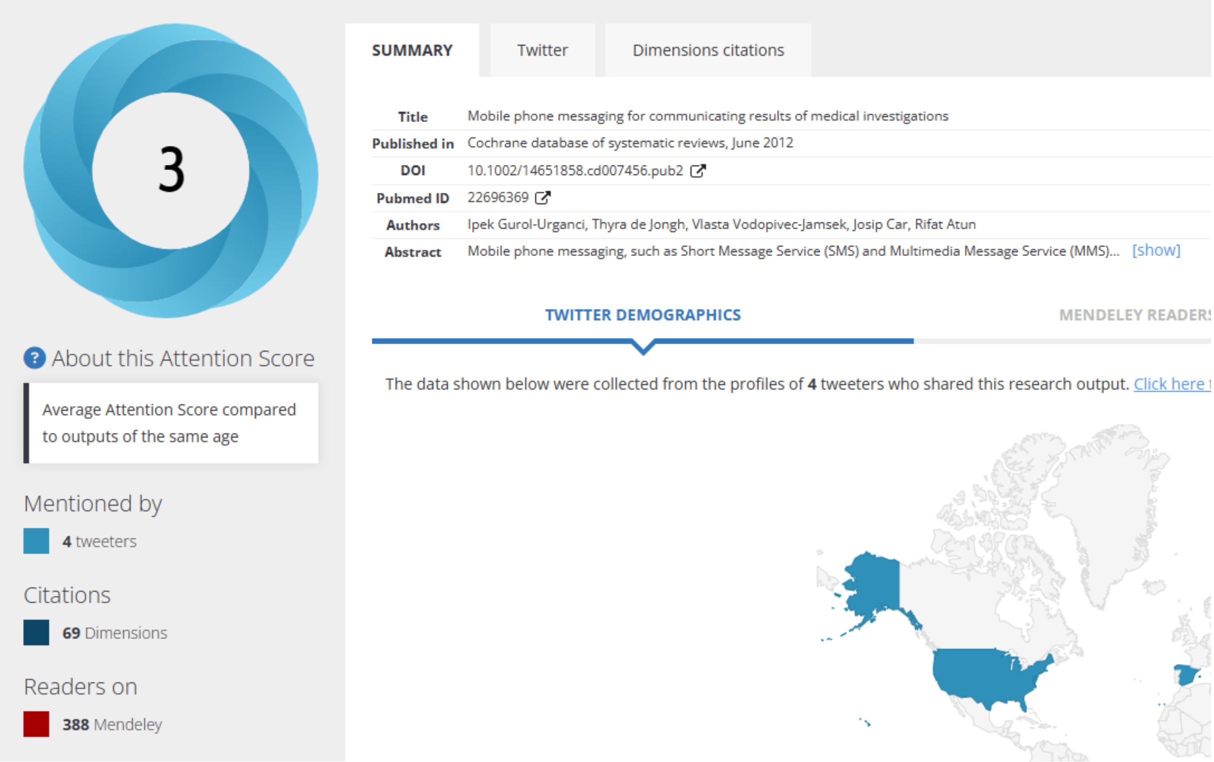

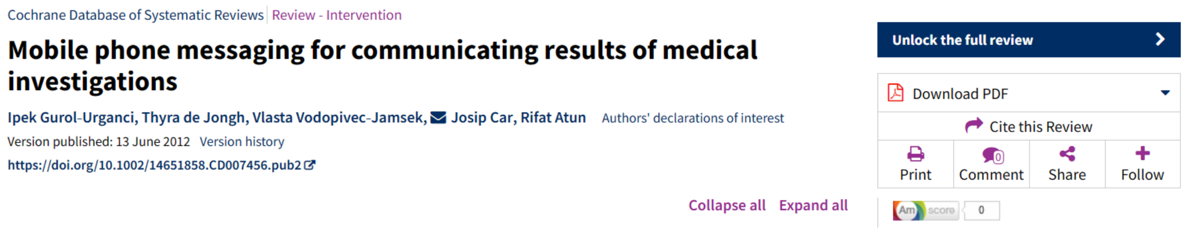

[53] Gurusamy, Vaughan and Davidson (2014) Formal education of patients about to undergo laparoscopic cholecystectomy

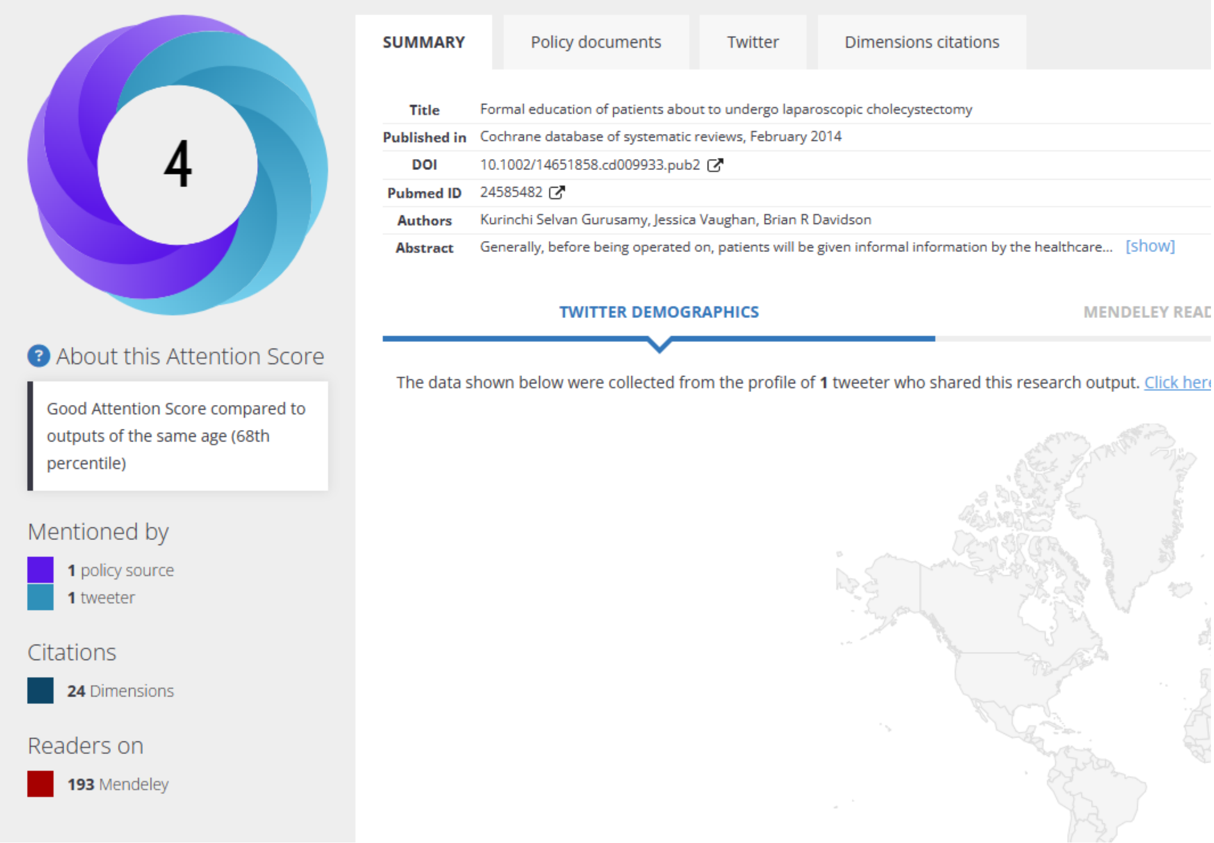

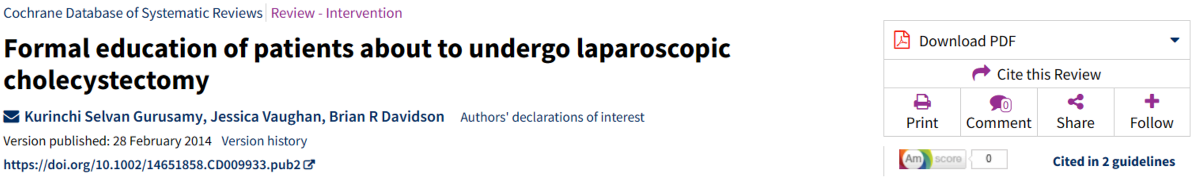

[54] Handford, Tynan, Rackal and Glazier (2006) Setting and organization of care for persons living with HIV/AIDS

?

Citations

54 Dimensions

Readers on

247 Mendeley

SUMMARY | Dimensions citations

**Title**	Setting and organization of care for persons living with HIV/AIDS
**Published in**	Cochrane database of systematic reviews, July 2006
**DOI**	10.1002/14651858.cd004348.pub2
**Pubmed ID**	16856042
**Authors**	Curtis Handford, Anne-Marie Tynan, Julia M Rackal, Richard Glazier
**Abstract**	Treating the world's 40.3 million persons currently infected with HIV/AIDS is an international... [show]

ME

The data shown below were compiled from readership statistics for **247** Mendeley readers of this

Cochrane Database of Systematic Reviews | Review - Intervention

Setting and organization of care for persons living with HIV/AIDS

Curtis Handford, Anne-Marie Tynan, Julia M Rackal, Richard Glazier   Authors' declarations of interest

Version published: 19 July 2006   Version history

https://doi.org/10.1002/14651858.CD004348.pub2

Collapse all   Expand all

Abstract

Unlock the full review

Download PDF

Cite this Review

Print | Comment | Share | Follow

score 0

[55] Horvath, Azman, Kennedy and Rutherford (2012) Mobile phone text messaging for promoting adherence to antiretroviral therapy in patients with HIV infection

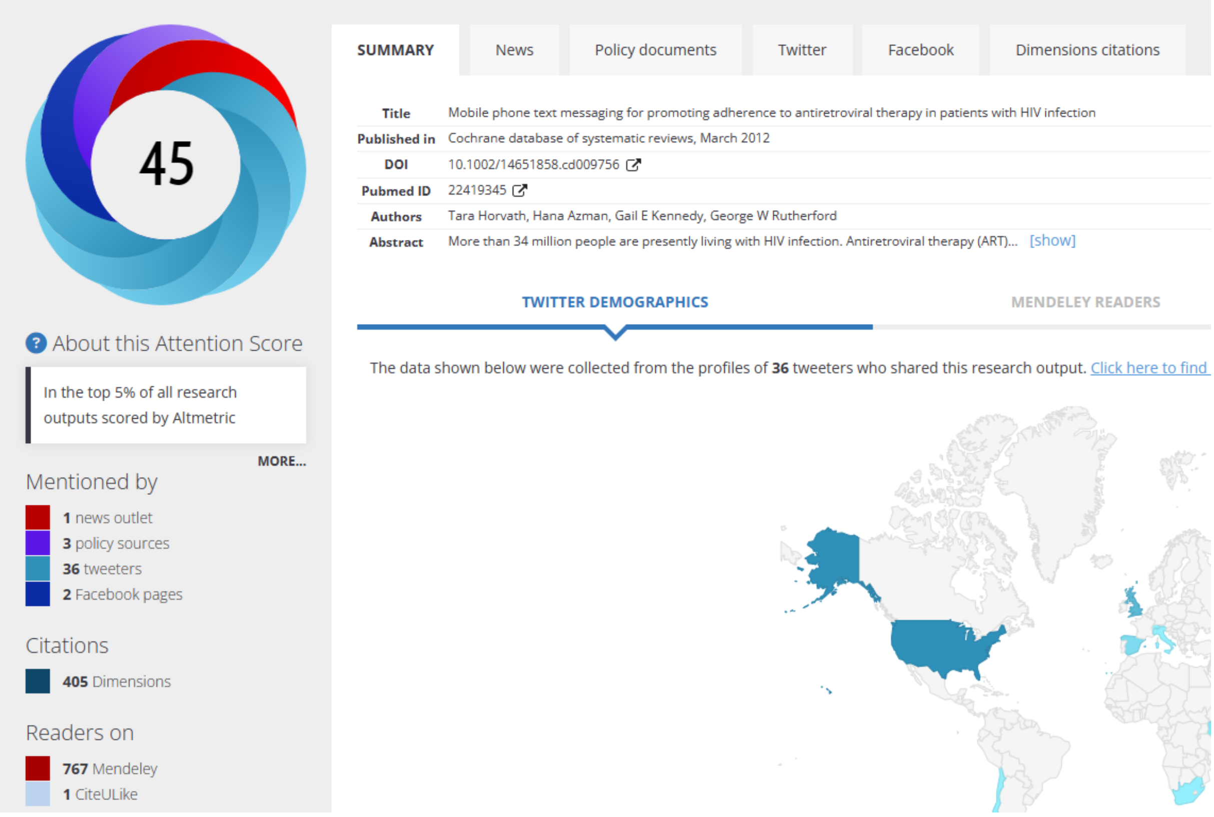

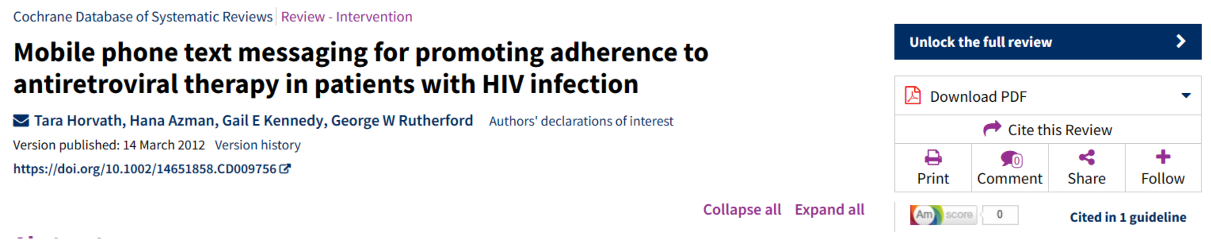

[56] Inglis, Clark, Dierckx, Prieto-Merino and Cleland (2015) Structured telephone support or non-invasive telemonitoring for patients with heart failure

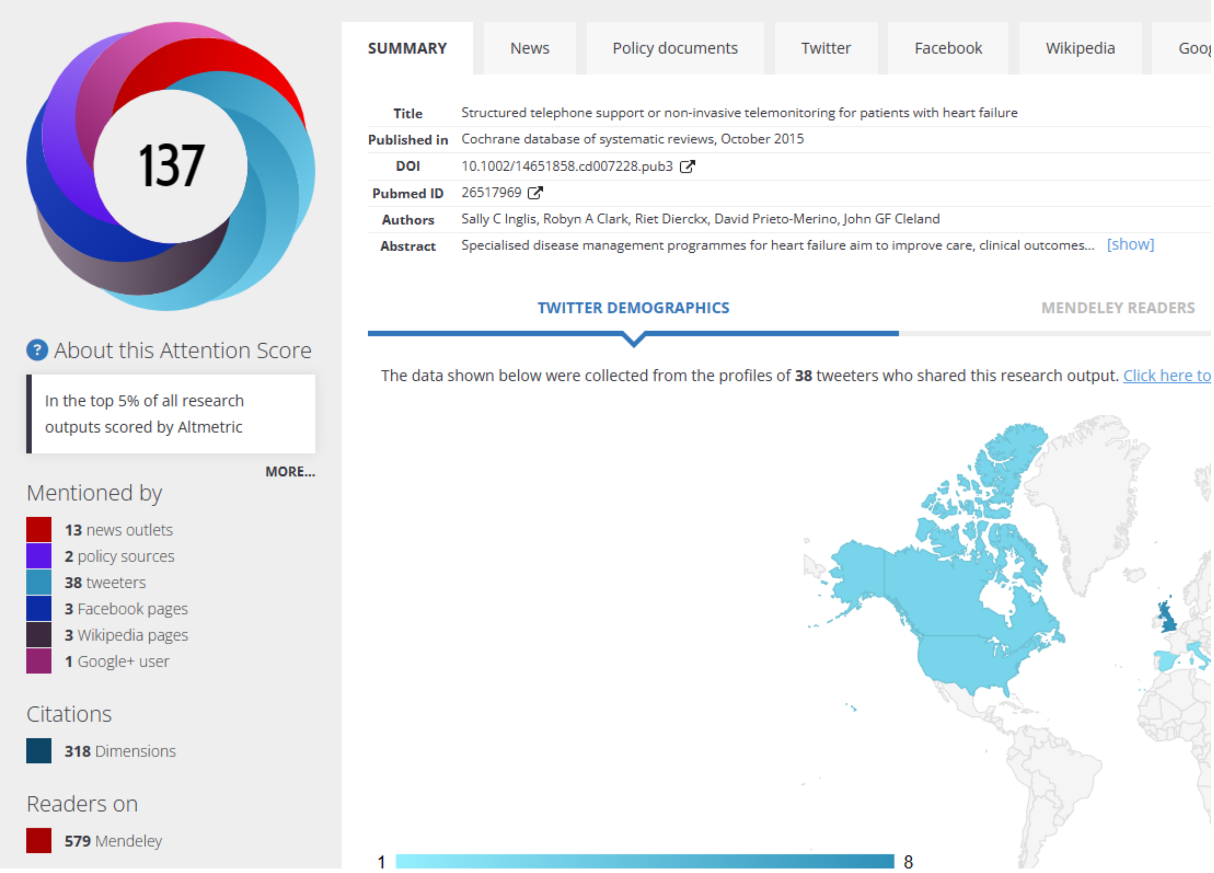

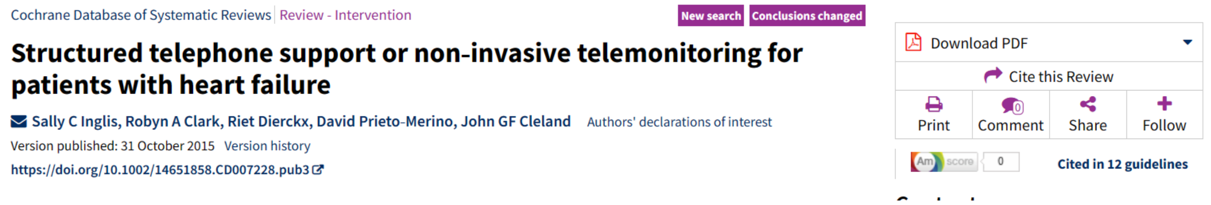

[57] Jacobson Vann, Jacobson, Coyne-Beasley, Asafu-Adjei and Szilagyi (2018) Patient reminder and recall interventions to improve immunization rates

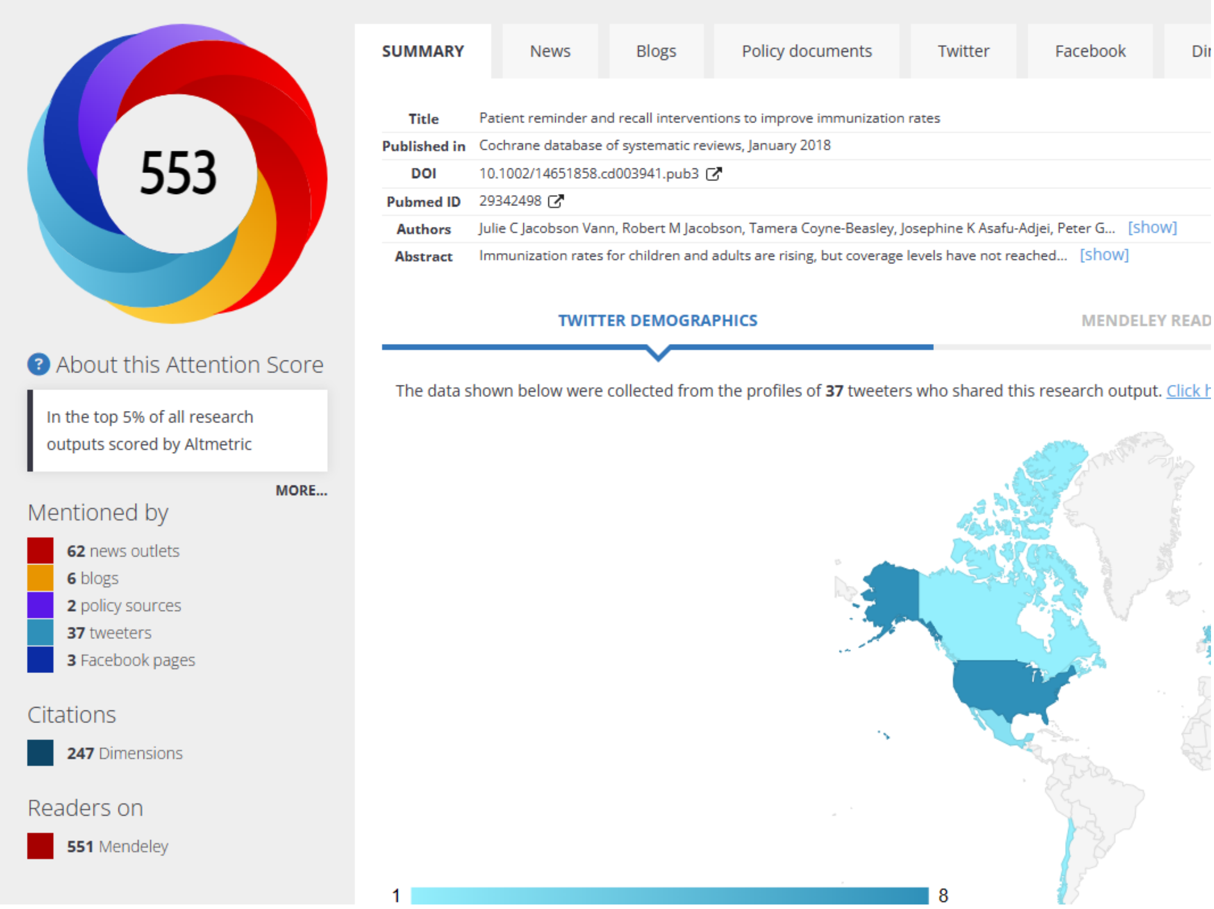

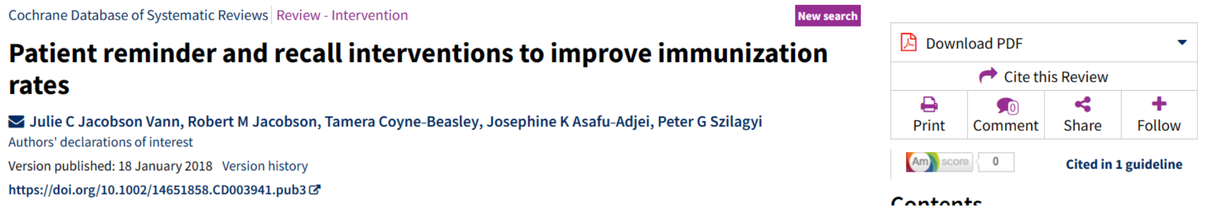

[58] Janjua, Banchoff, Threapleton, Prigmore, Fletcher and Disler (2021) Digital interventions for the management of chronic obstructive pulmonary disease

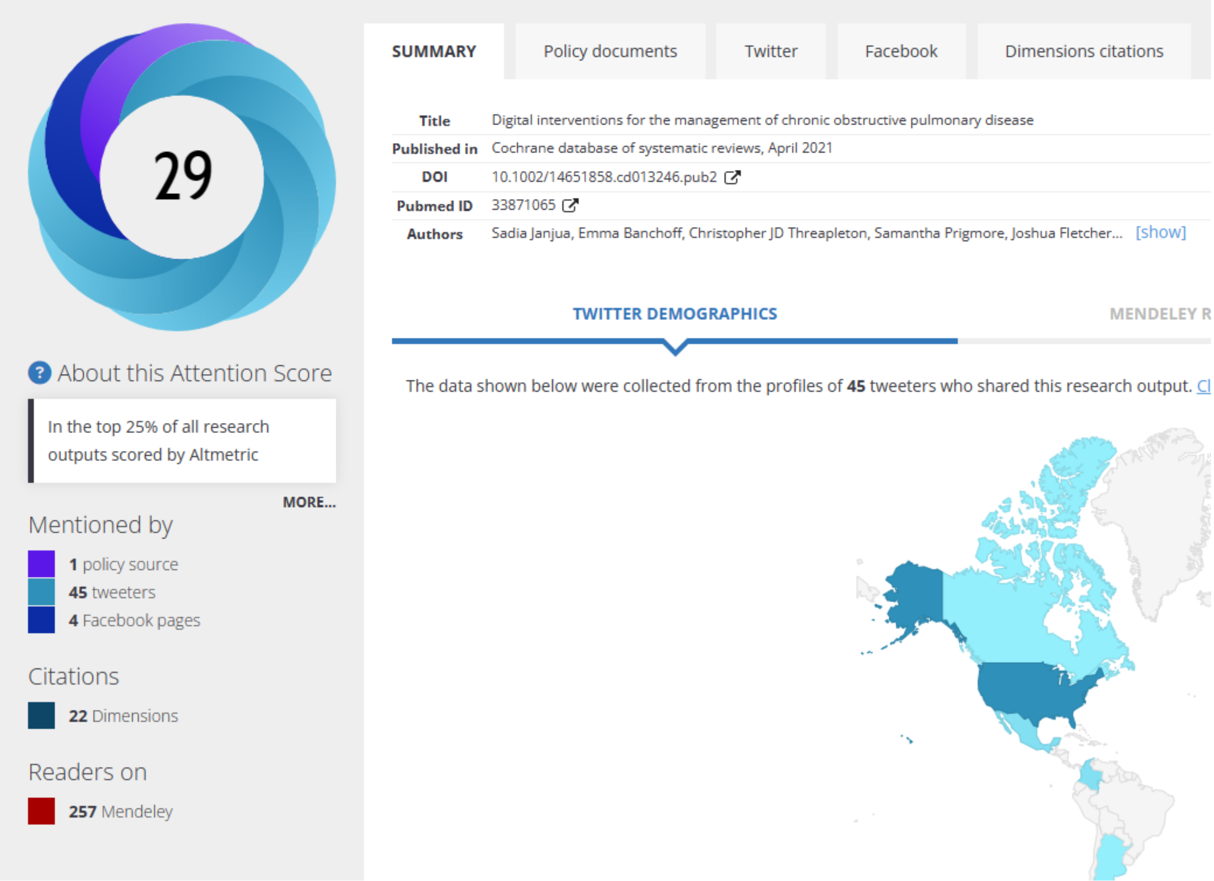

Cochrane Database of Systematic Reviews Review - Intervention

Digital interventions for the management of chronic obstructive pulmonary disease

Sadia Janjua, Emma Banchoff, Christopher JD Threapleton, Samantha Prigmore, Joshua Fletcher, Rebecca T Disler

Authors' declarations of interest

Version published: 19 April 2021 Version history

https://doi.org/10.1002/14651858.CD013246.pub2

Download PDF

Cite this Review

Print Comment Share Follow

score 0

[59] Janjua, Carter, Threapleton, Prigmore and Disler (2021) Telehealth interventions: remote monitoring and consultations for people with chronic obstructive pulmonary disease (COPD)

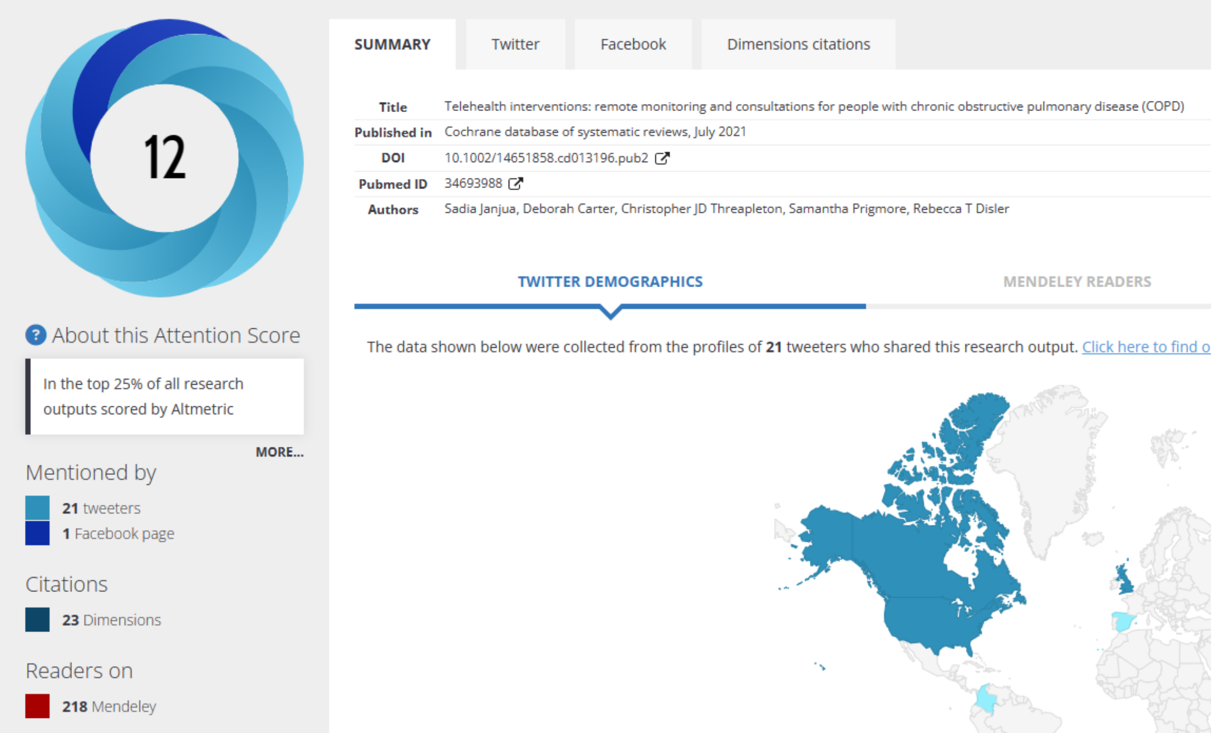

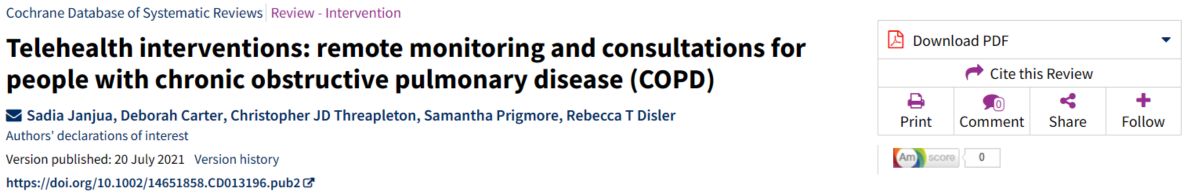

[60] Jawad, Jawad and Alwan (2019) Interventions using social networking sites to promote contraception in women of reproductive age

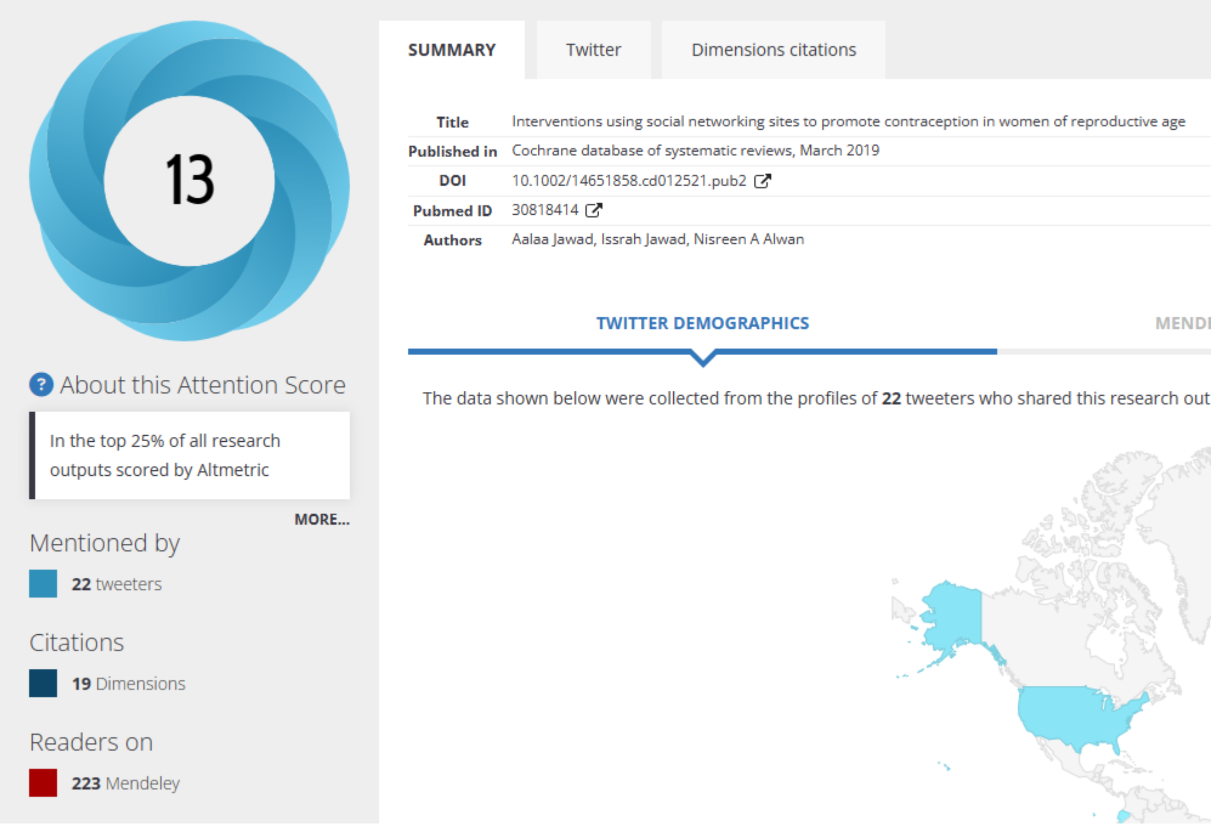

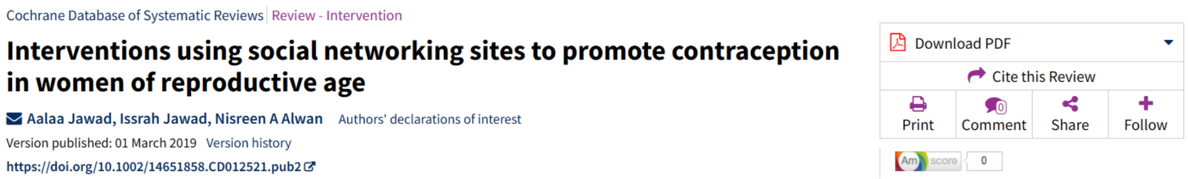

[61] Kaner, Beyer, Garnett, Crane, Brown, Muirhead, Redmore, O'Donnell, Newham, de Vocht and et al. (2017) Personalised digital interventions for reducing hazardous and harmful alcohol consumption in community-dwelling populations

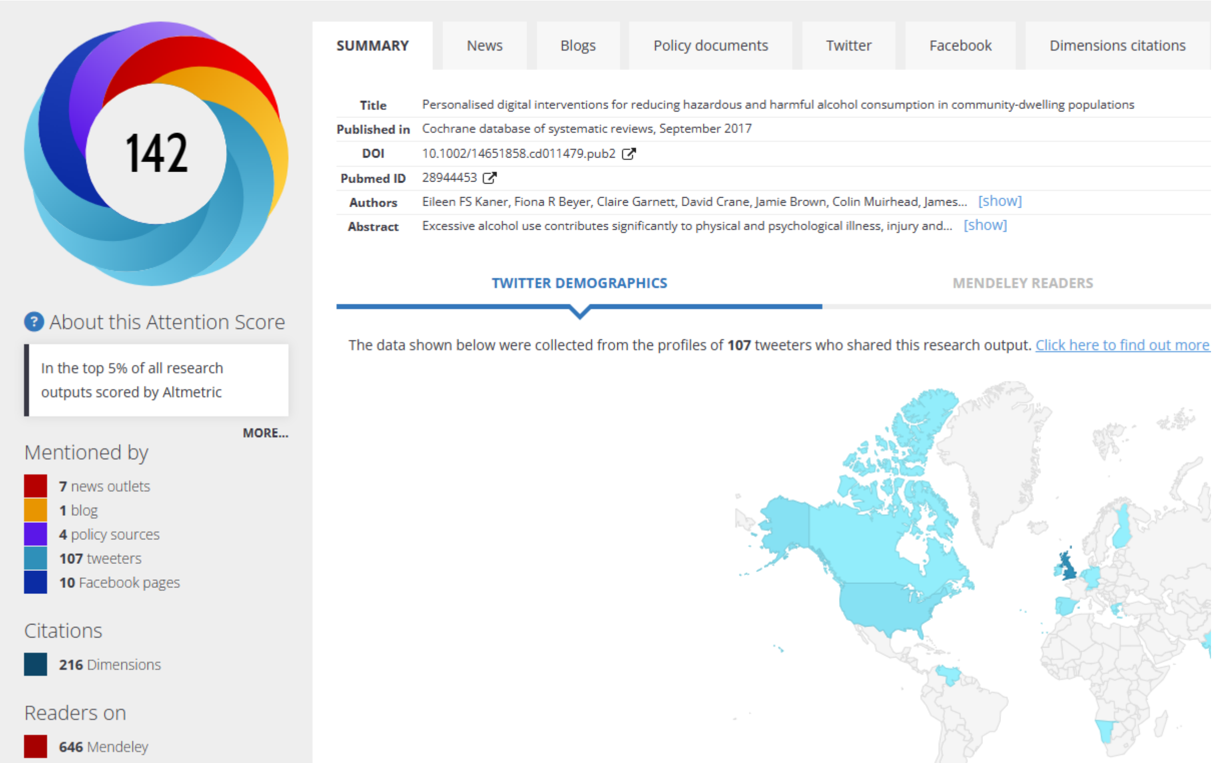

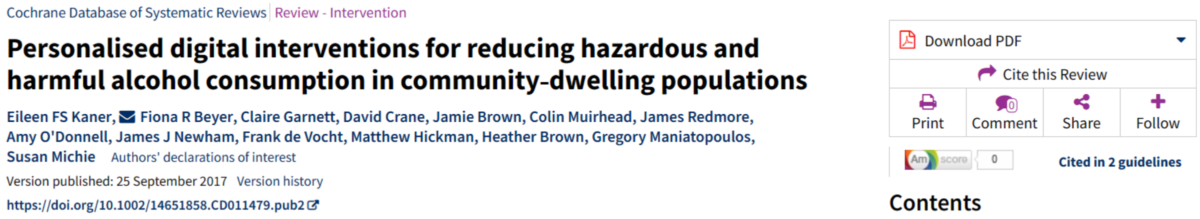

[62] Kauppi, Välimäki, Hätönen, Kuosmanen, Warwick-Smith and Adams (2014) Information and communication technology based prompting for treatment compliance for people with serious mental illness

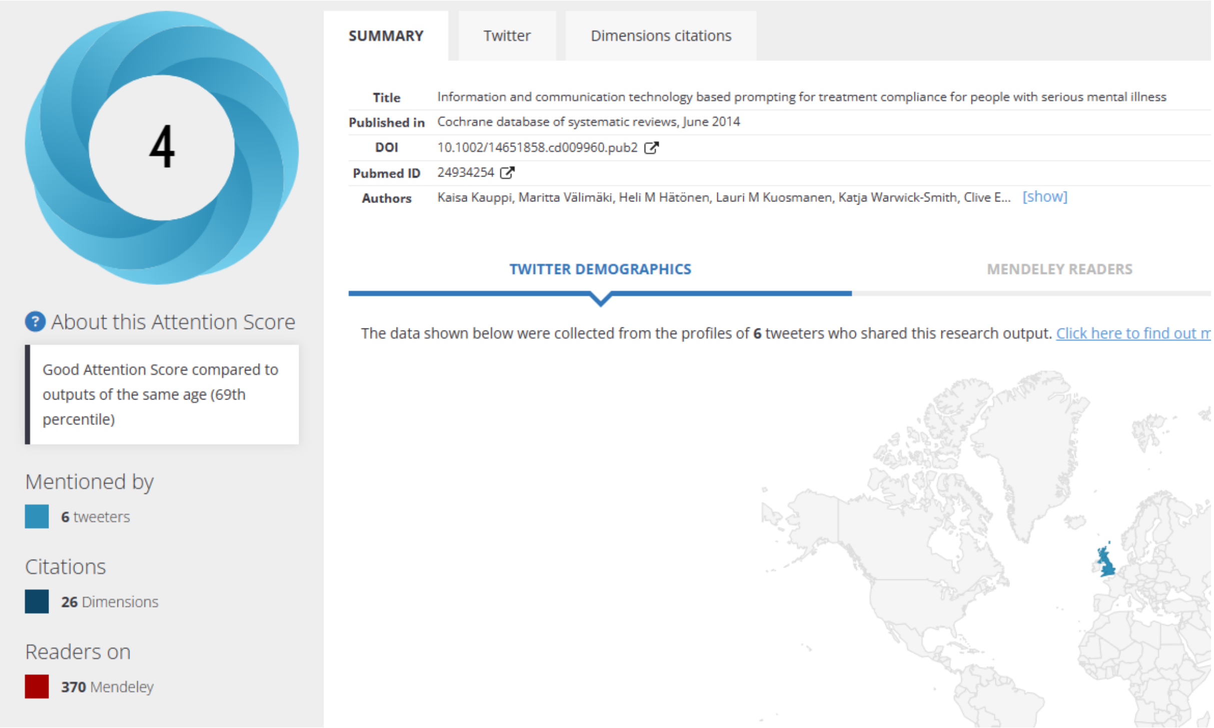

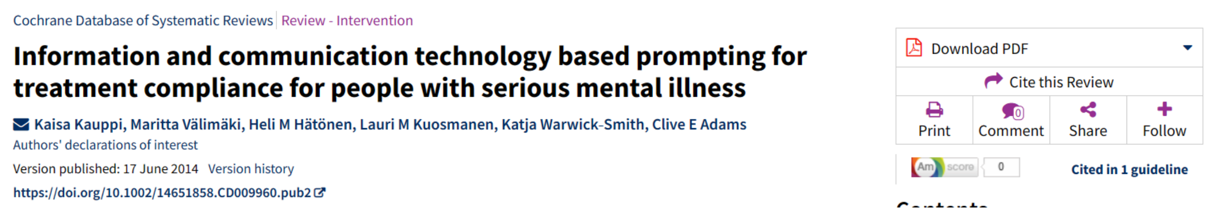

[63] Kew and Cates (2016) Home telemonitoring and remote feedback between clinic visits for asthma

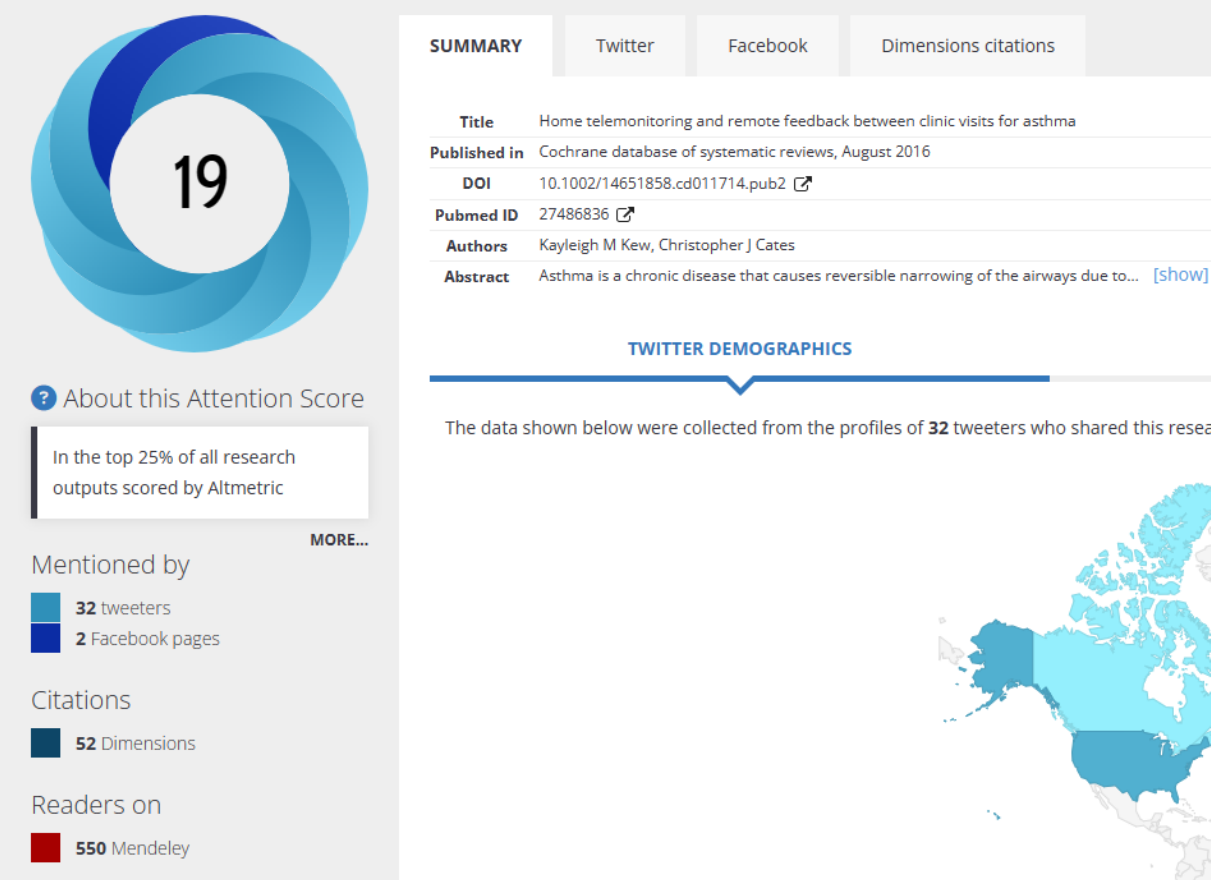

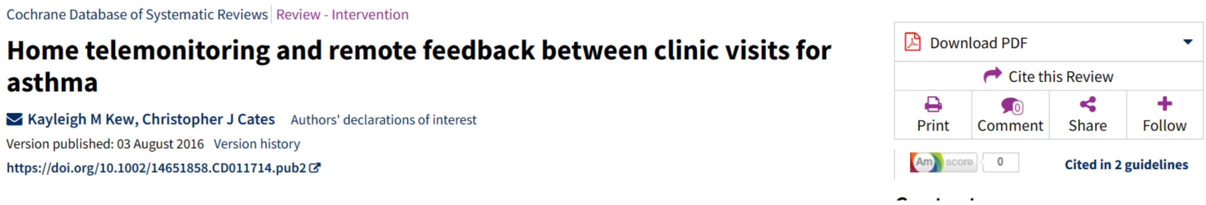

[64] Kew and Cates (2016) Remote versus face-to-face check-ups for asthma

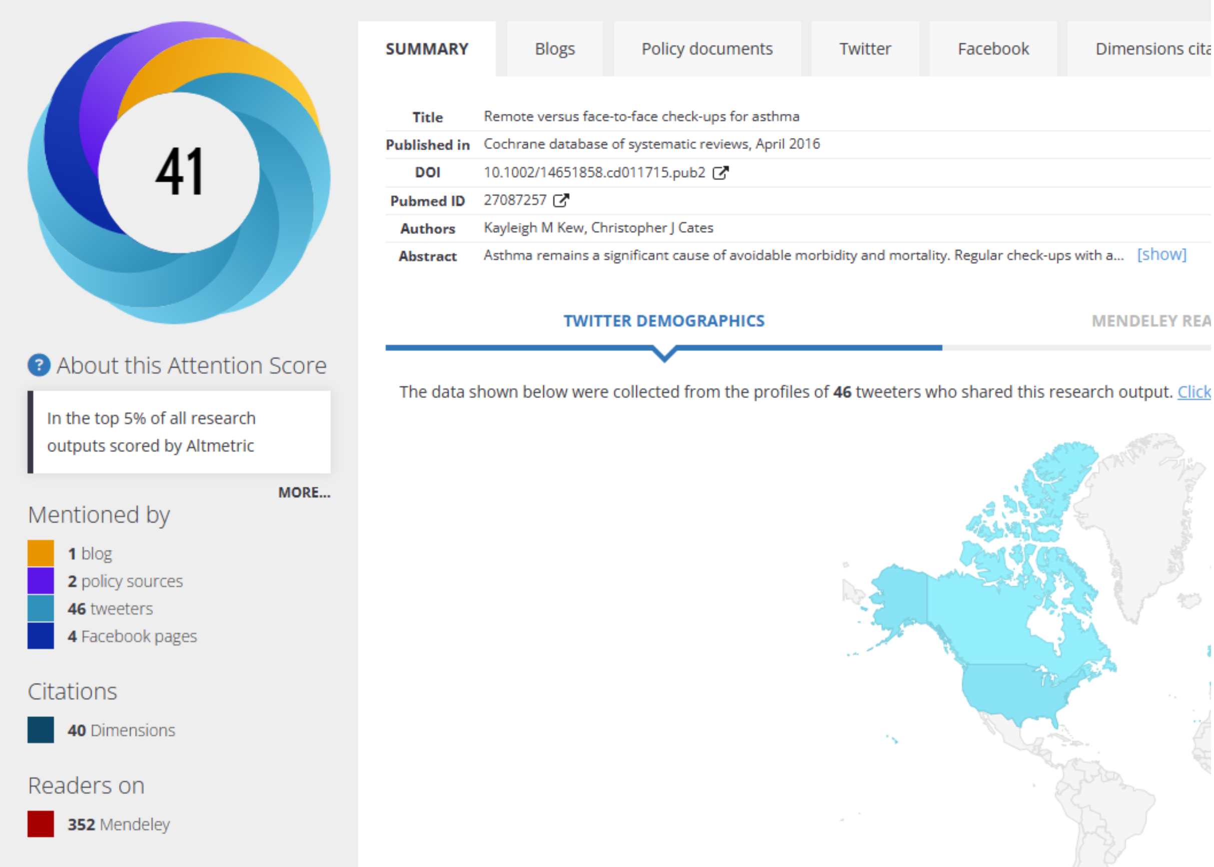

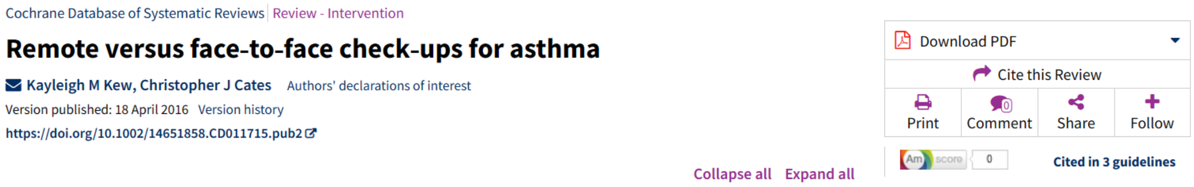

[65] Khan, Amatya, Kesselring and Galea (2015) Telerehabilitation for persons with multiple sclerosis

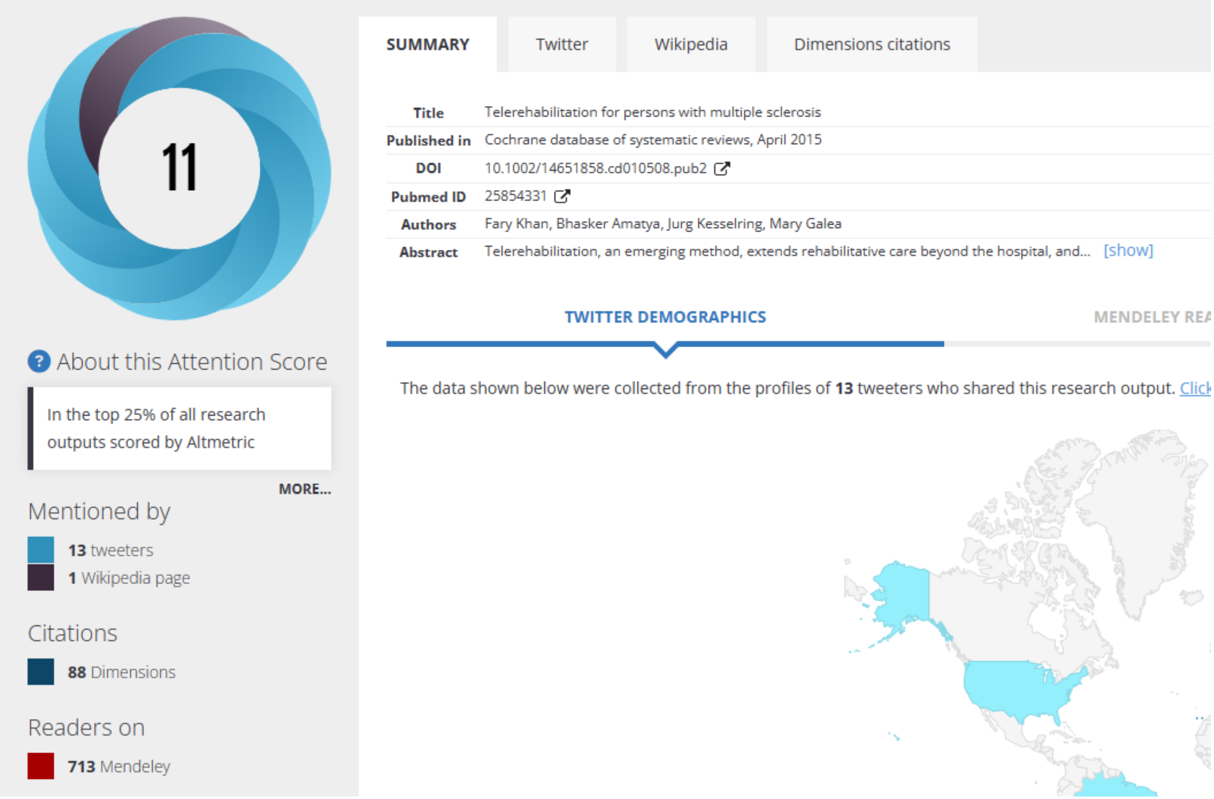

11

About this Attention Score

In the top 25% of all research outputs scored by Altmetric

MORE...

Mentioned by

13 tweeters

1 Wikipedia page

Citations

88 Dimensions

Readers on

713 Mendeley

SUMMARY | Twitter | Wikipedia | Dimensions citations

**Title**	Telerehabilitation for persons with multiple sclerosis
**Published in**	Cochrane database of systematic reviews, April 2015
**DOI**	10.1002/14651858.cd010508.pub2
**Pubmed ID**	25854331
**Authors**	Fary Khan, Bhasker Amatya, Jurg Kesselring, Mary Galea
**Abstract**	Telerehabilitation, an emerging method, extends rehabilitative care beyond the hospital, and... [show]

TWITTER DEMOGRAPHICS MENDELEY REA

The data shown below were collected from the profiles of **13** tweeters who shared this research output. Click

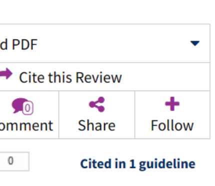

Cochrane Database of Systematic Reviews | Review - Intervention

Telerehabilitation for persons with multiple sclerosis

Fary Khan, Bhasker Amatya, Jurg Kesselring, Mary Galea Authors' declarations of interest

Version published: 09 April 2015 Version history

https://doi.org/10.1002/14651858.CD010508.pub2

Download PDF

Cite this Review

Print Comment Share Follow

0

Cited in 1 guideline

Collapse all Expand all

[66] Kobayashi, Hanada, Matsuzaki, Takehara, Ota, Sasaki, Nagata and Mori (2017) Assessment and support during early labour for improving birth outcomes

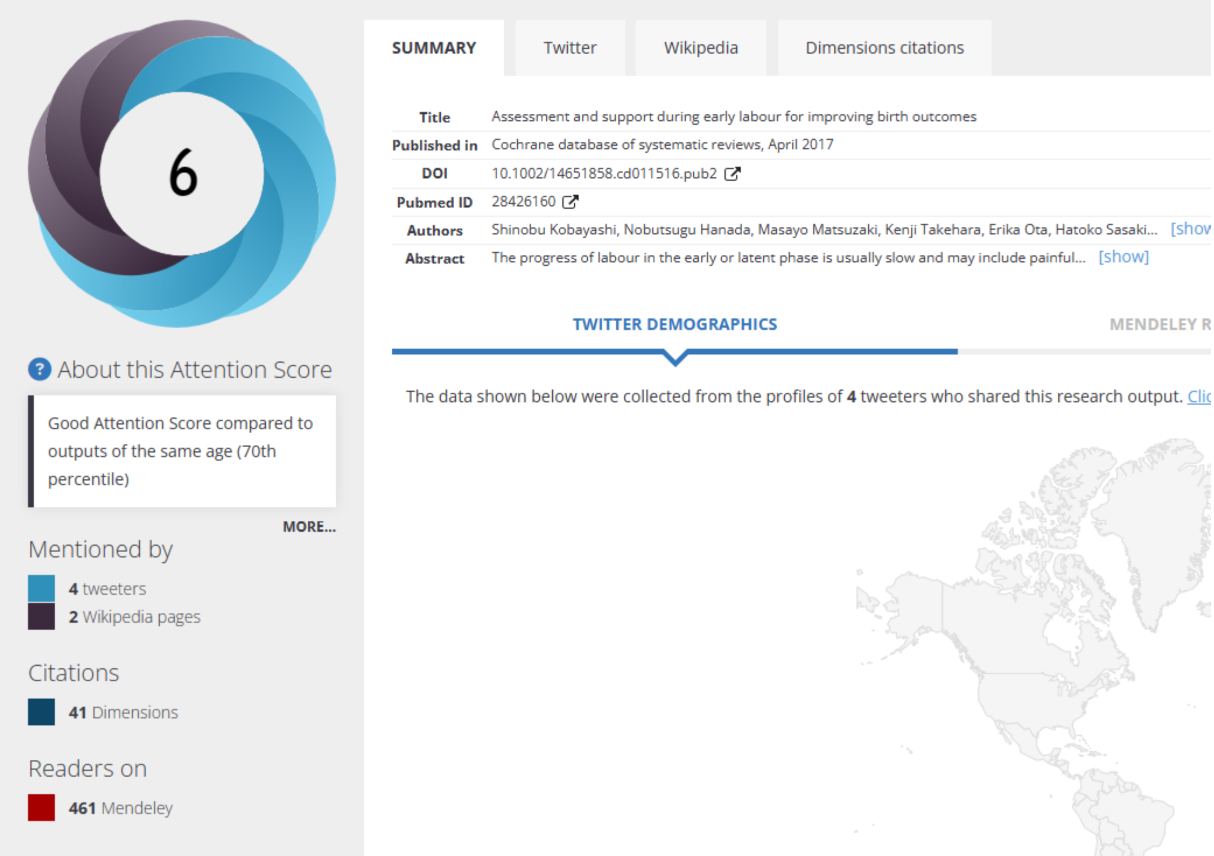

Cochrane Database of Systematic Reviews | Review - Intervention

Assessment and support during early labour for improving birth outcomes

Shinobu Kobayashi, Nobutsugu Hanada, Masayo Matsuzaki, Kenji Takehara, Erika Ota, Hatoko Sasaki, Chie Nagata, Rintaro Mori Authors' declarations of interest

Version published: 20 April 2017 Version history
https://doi.org/10.1002/14651858.CD011516.pub2

Download PDF
Cite this Review
Print Comment Share Follow
0
Cited in 4 guidelines

[67] Kuster, Dalsbø, Luong Thanh, Agarwal, Durand-Moreau and Kirkehei (2017) Computer-based versus in-person interventions for preventing and reducing stress in workers

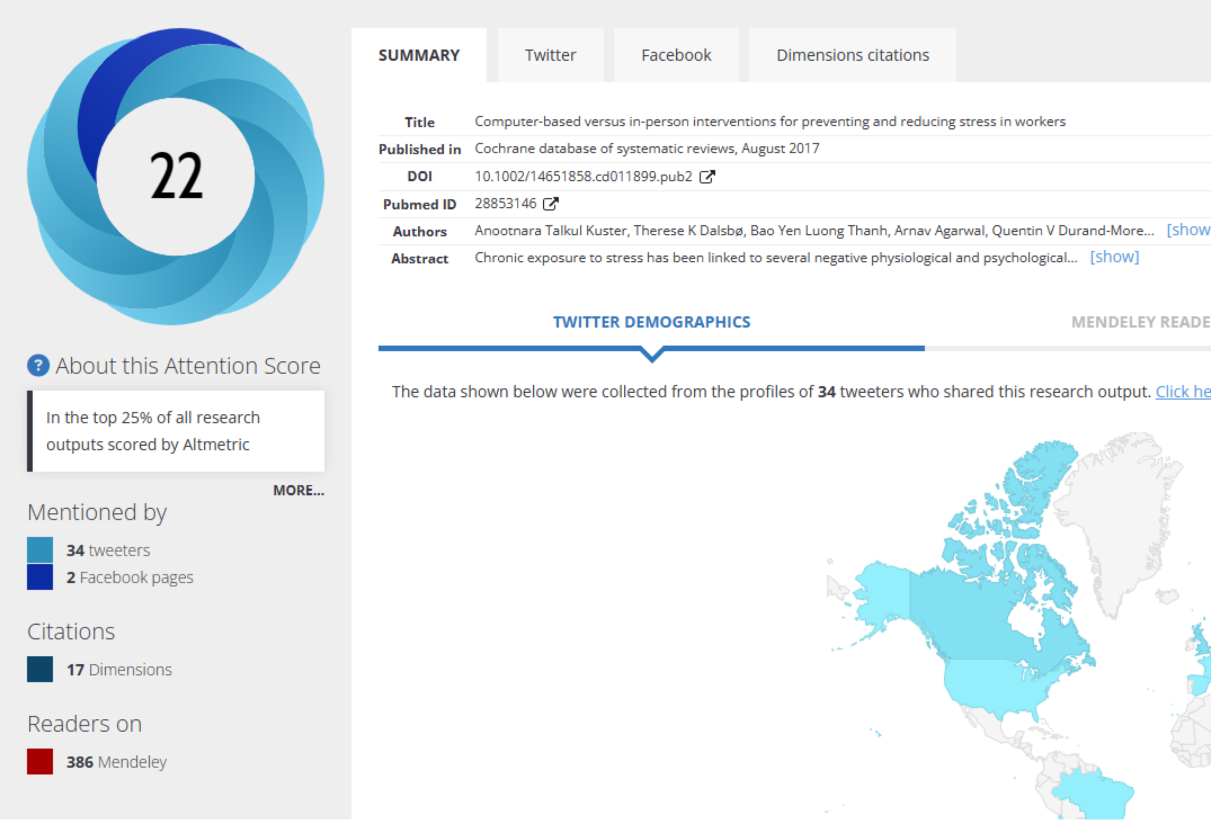

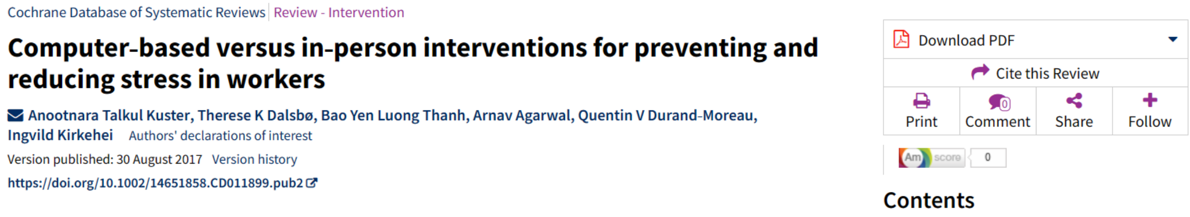

[68] Lavender, Richens, Milan, Smyth and Dowswell (2013) Telephone support for women during pregnancy and the first six weeks postpartum

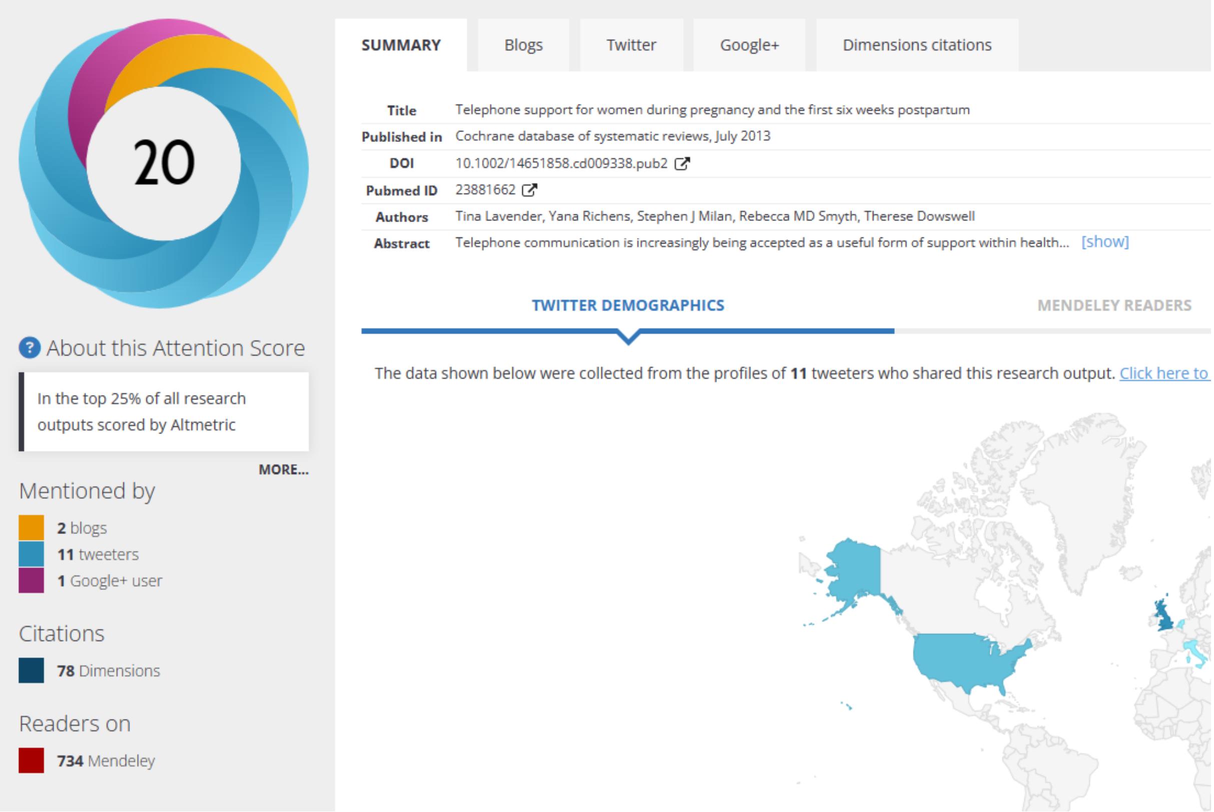

Cochrane Database of Systematic Reviews | Review - Intervention

Telephone support for women during pregnancy and the first six weeks postpartum

Tina Lavender, Yana Richens, Stephen J Milan, Rebecca MD Smyth, Therese Dowswell Authors' declarations of interest

Version published: 18 July 2013 Version history

https://doi.org/10.1002/14651858.CD009338.pub2

Download PDF
Cite this Review
Print Comment Share Follow
0
Cited in 4 guidelines

[69] Laver, Adey-Wakeling, Crotty, Lannin, George and Sherrington (2020) Telerehabilitation services for stroke

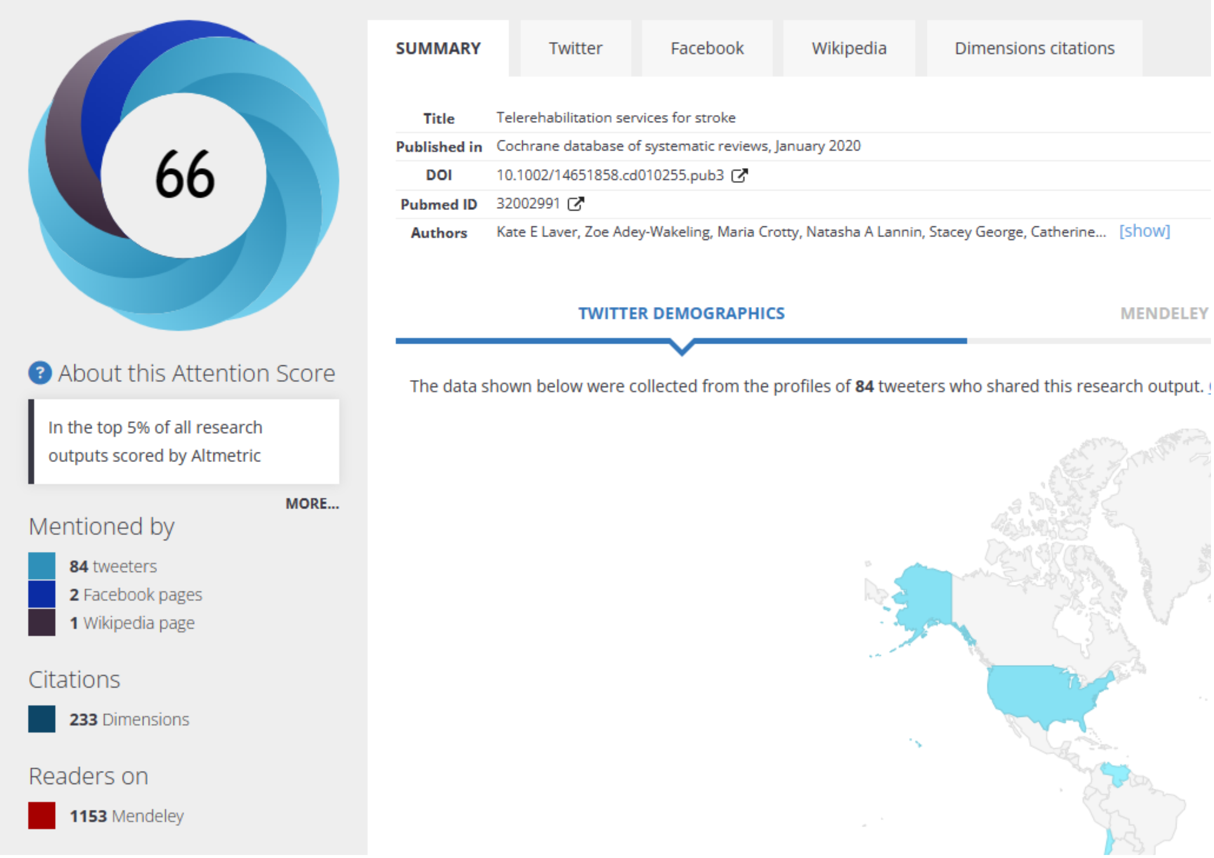

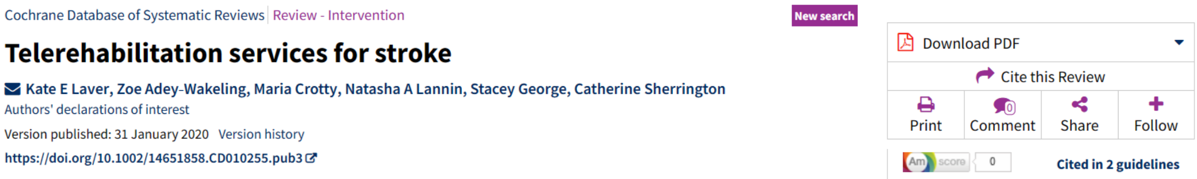

[70] Laver, Lange, George, Deutsch, Saposnik and Crotty (2017) Virtual reality for stroke rehabilitation

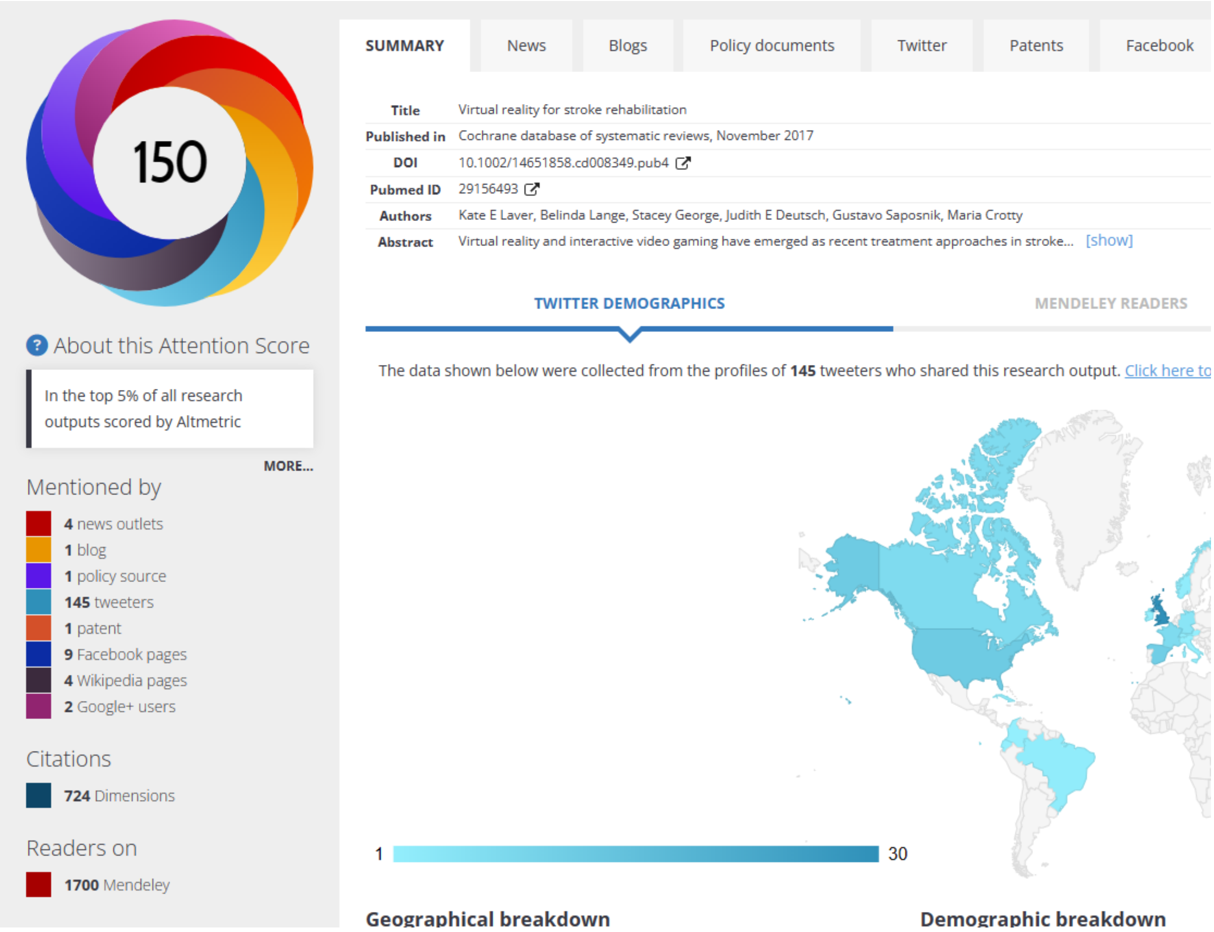

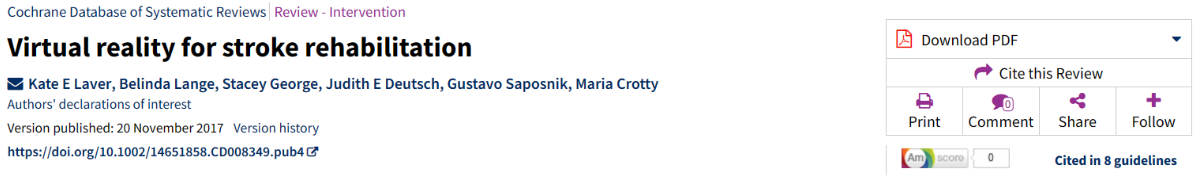

[71] Leon, Balakrishna, Hohlfeld, Odendaal, Schmidt, Zweigenthal, Anstey Watkins and Daniels (2020) Routine Health Information System (RHIS) improvements for strengthened health system management

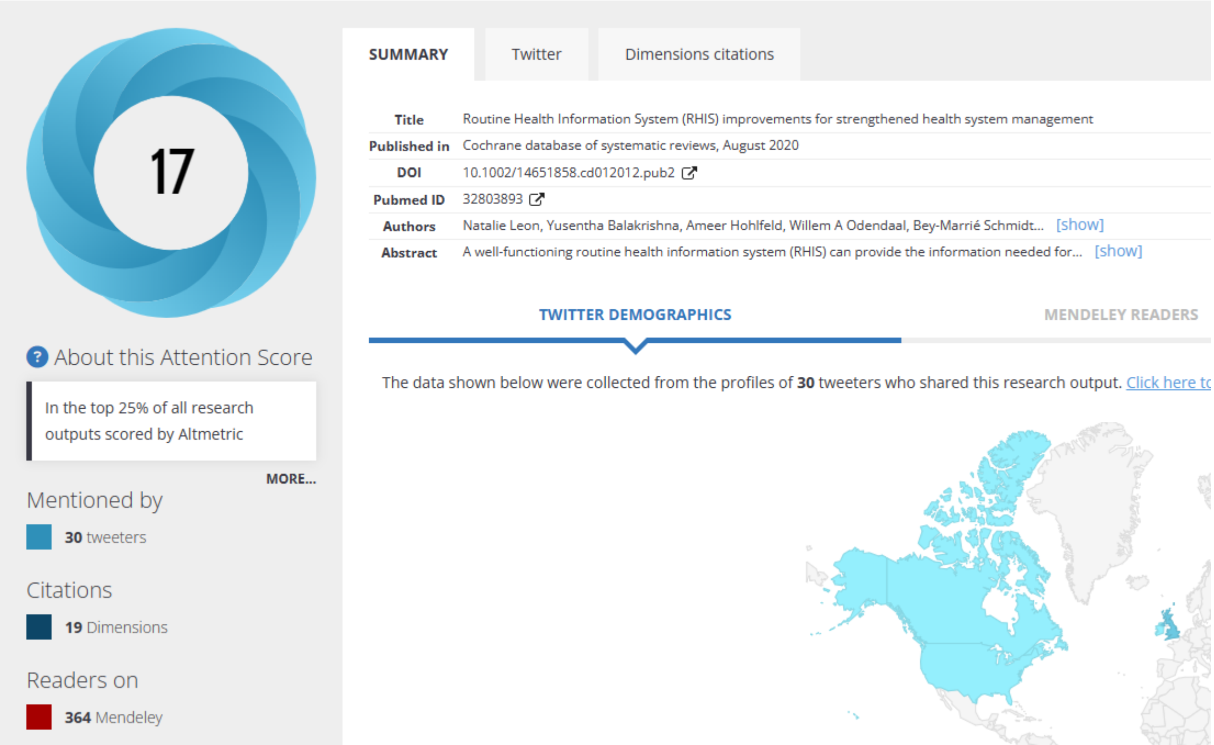

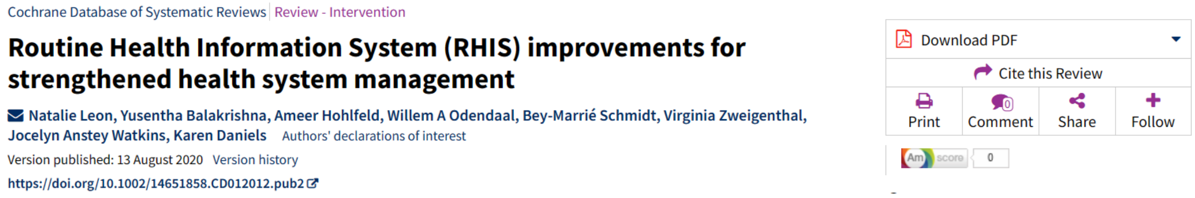

[72] Linden, Hawley, Blackwood, Evans, Anderson and O'Rourke (2016) Technological aids for the rehabilitation of memory and executive functioning in children and adolescents with acquired brain injury

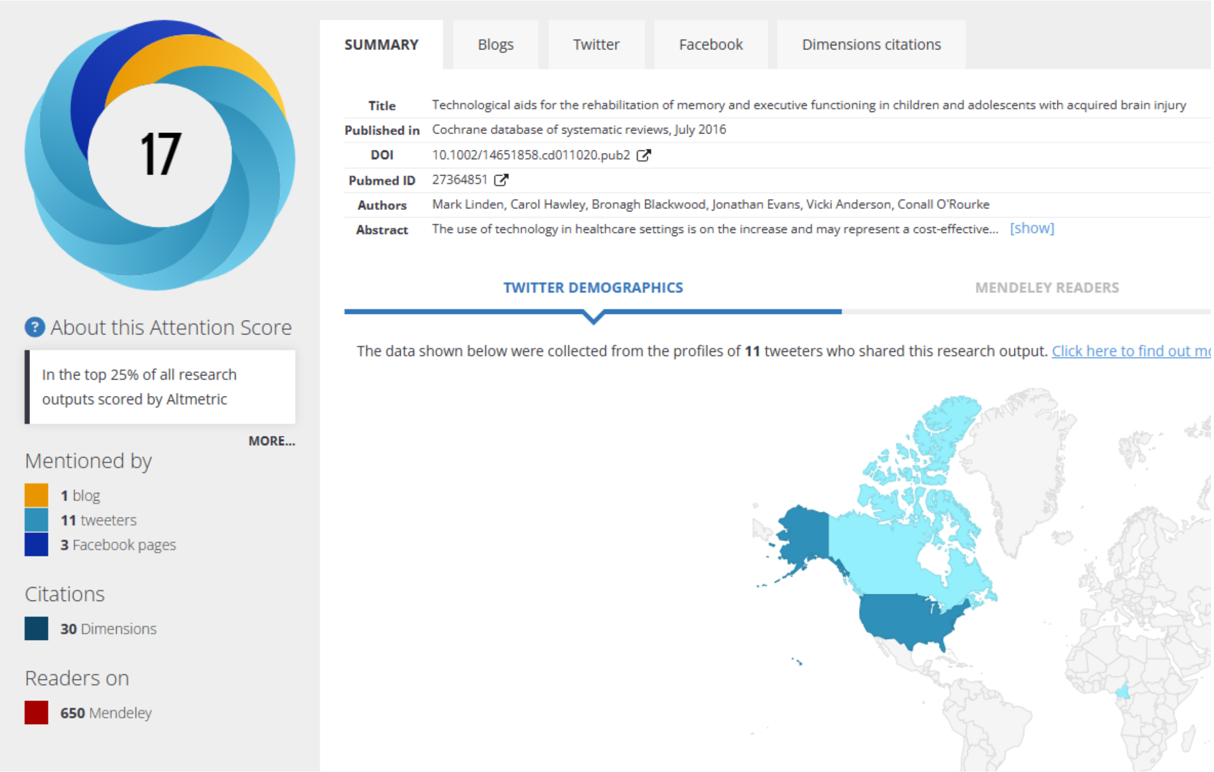

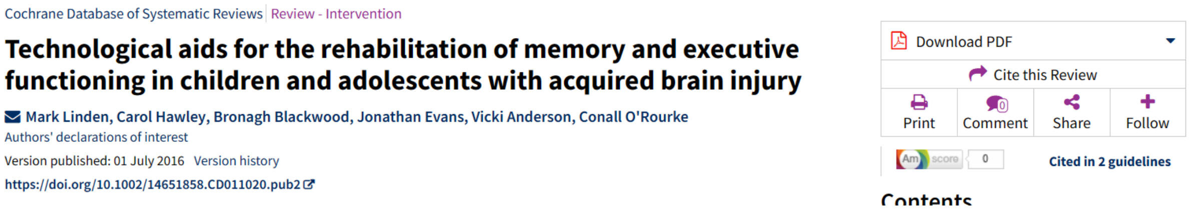

[73] Lins, Hayder-Beichel, Rücker, Motschall, Antes, Meyer and Langer (2014) Efficacy and experiences of telephone counselling for informal carers of people with dementia

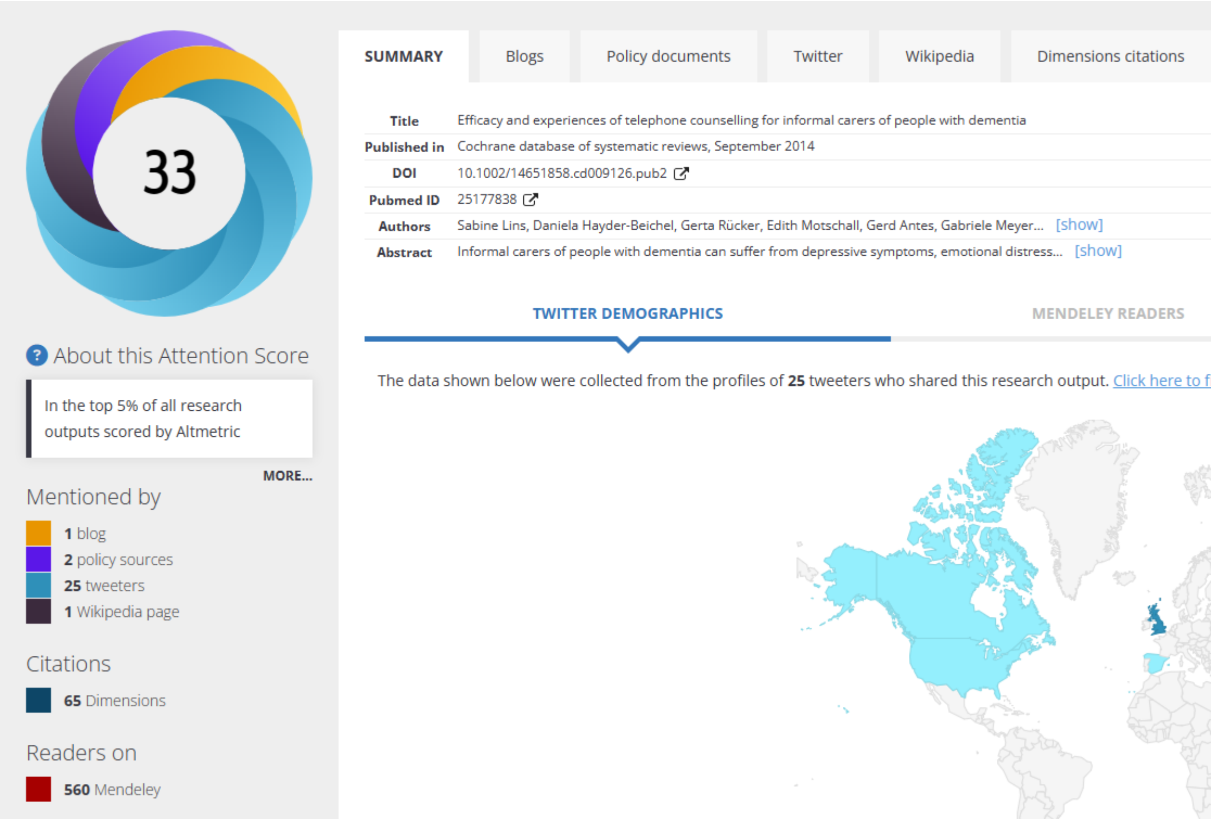

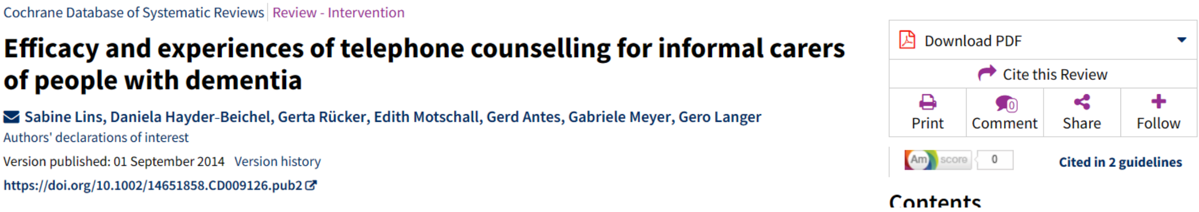

[74] Lopez, Stockton, Chen, Steiner and Gallo (2014) Behavioral interventions for improving dual-method contraceptive use

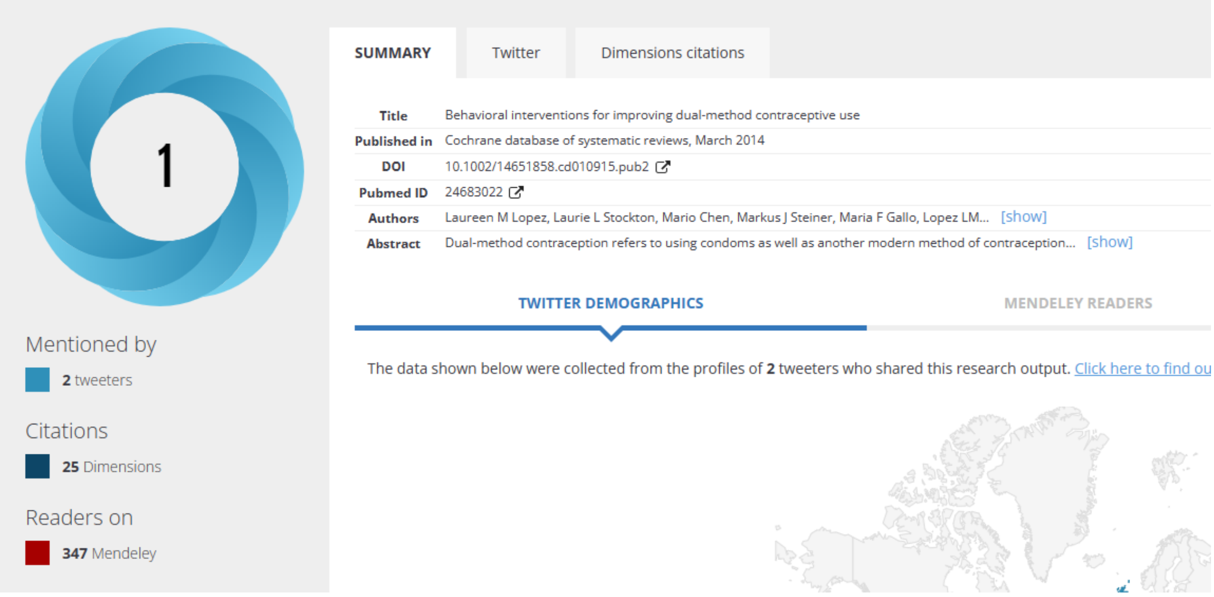

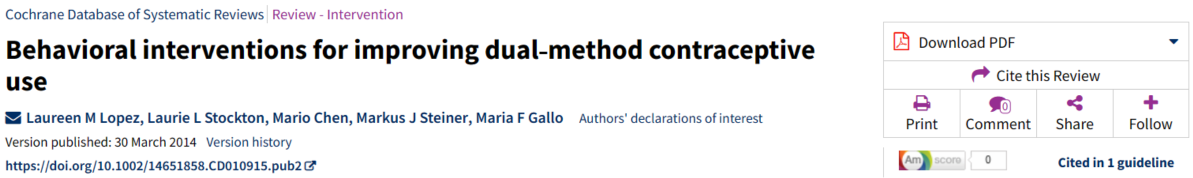

[75] Lynch, Jones, Simpson, Fini, Kuys, Borschmann, Kramer, Johnson, Callisaya, Mahendran and et al. (2018) Activity monitors for increasing physical activity in adult stroke survivors

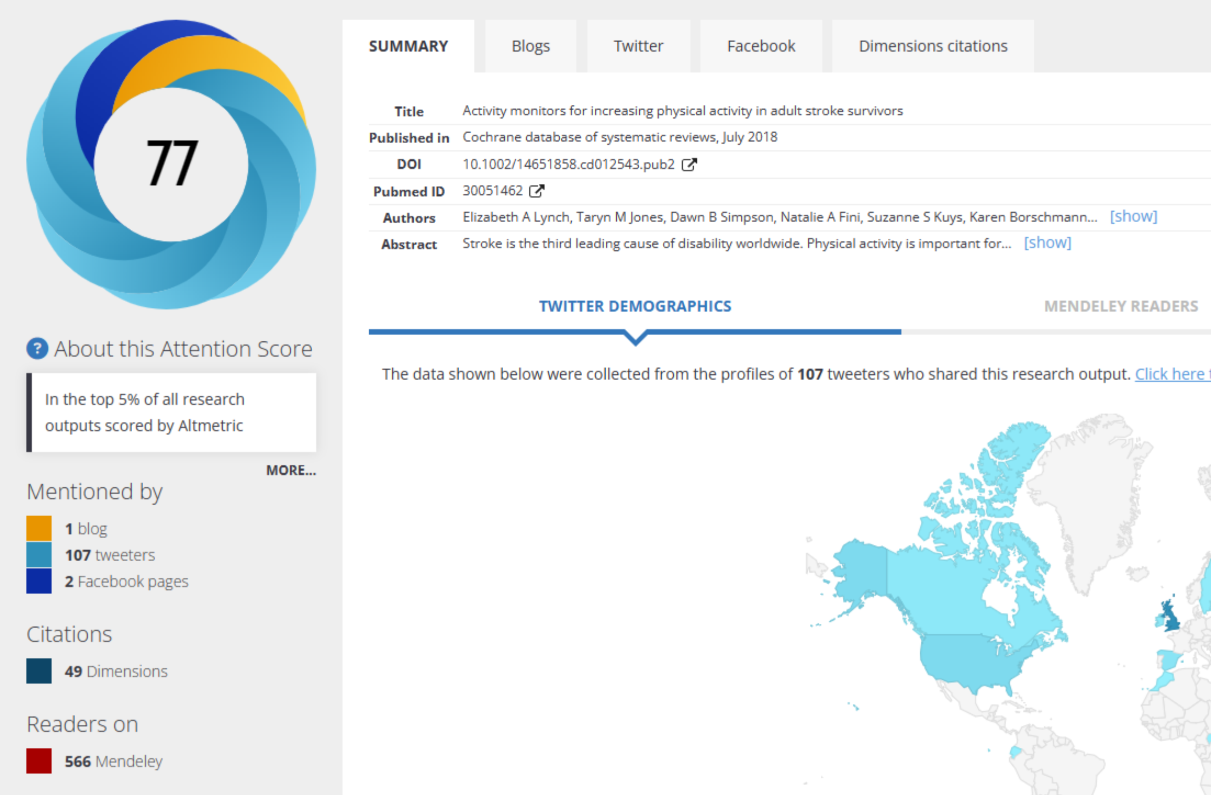

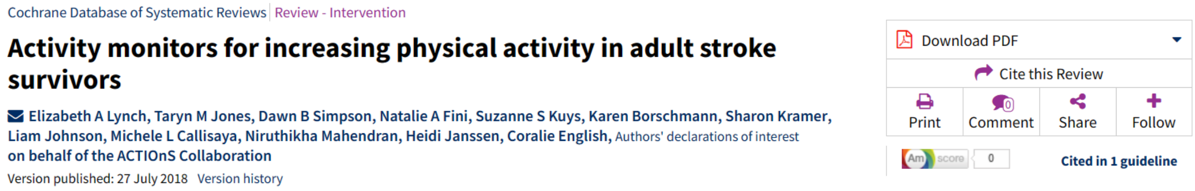

[76] Malaguti, Dal Corso, Janjua and Holland (2021) Supervised maintenance programmes following pulmonary rehabilitation compared to usual care for chronic obstructive pulmonary disease

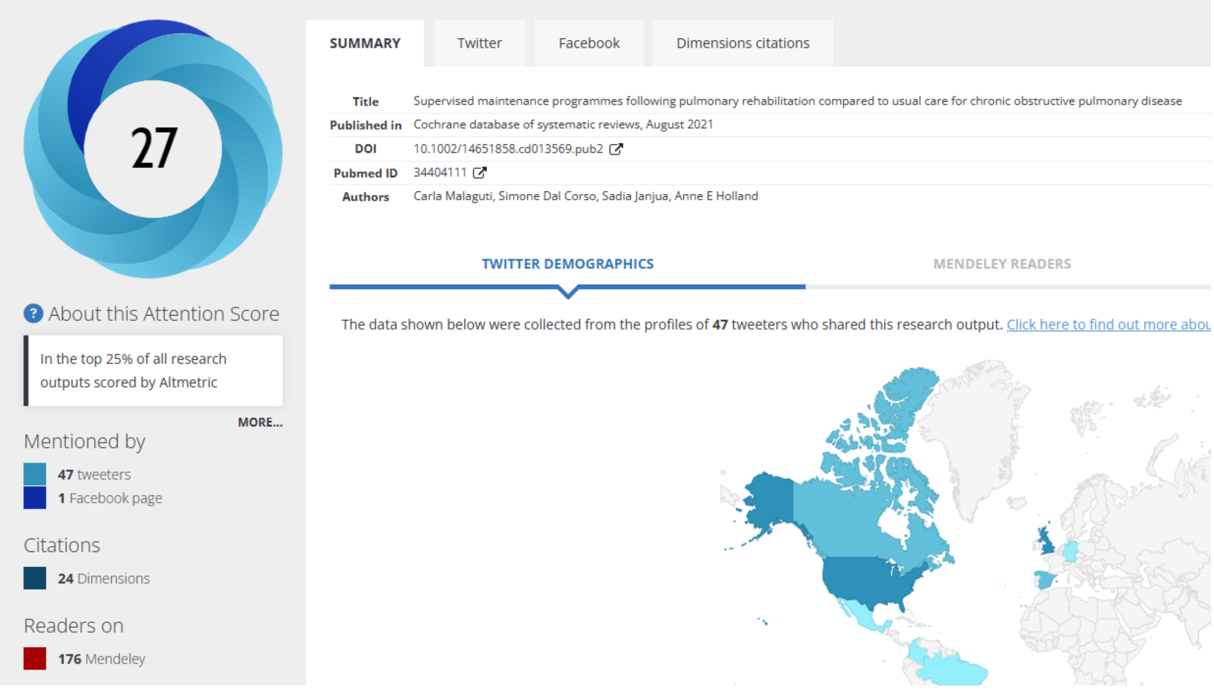

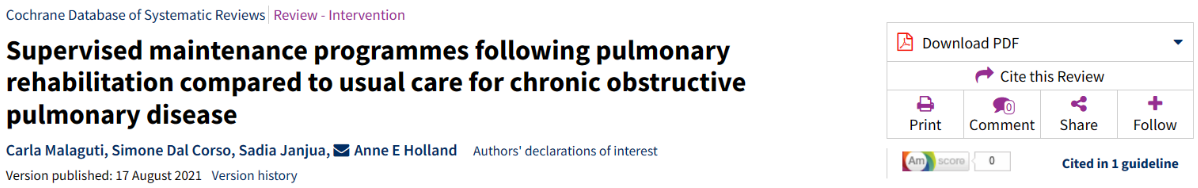

[77] Manyande, Cyna, Yip, Chooi and Middleton (2015) Non-pharmacological interventions for assisting the induction of anaesthesia in children

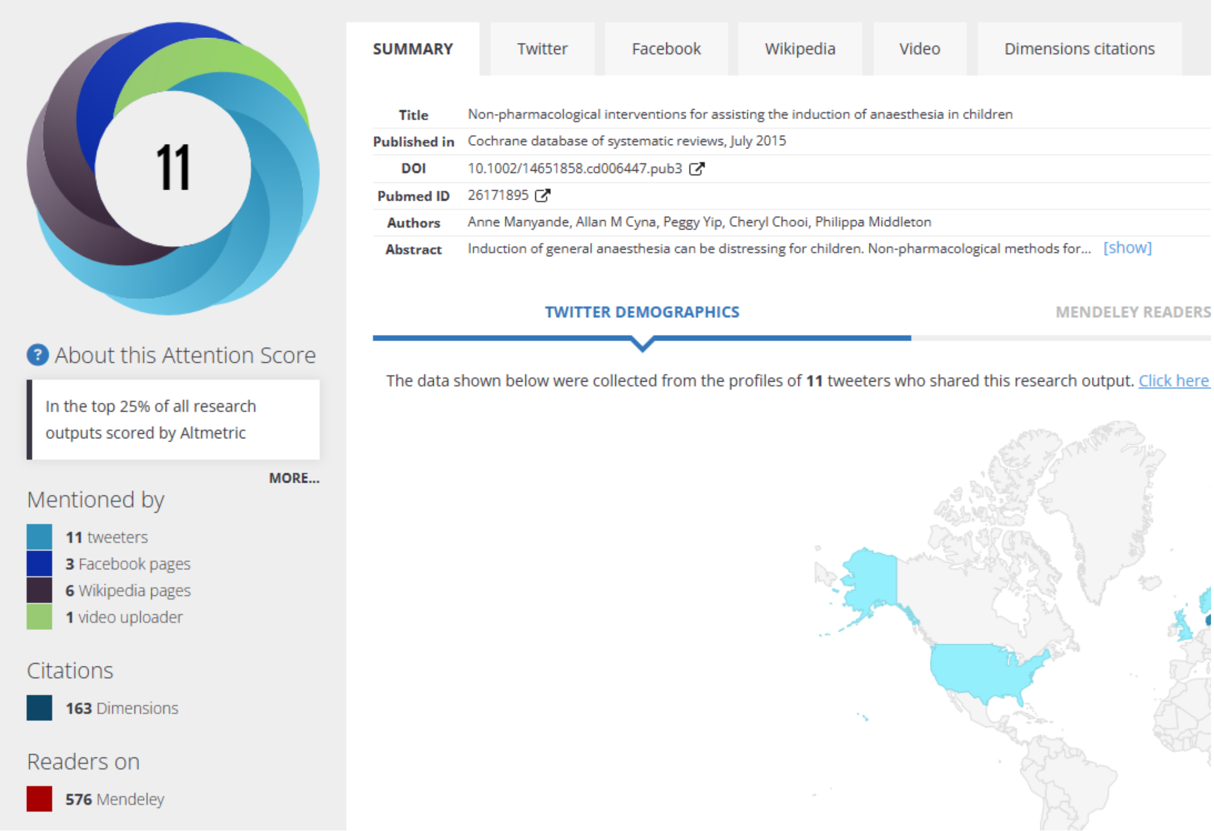

11

About this Attention Score

In the top 25% of all research outputs scored by Altmetric

MORE...

Mentioned by

11 tweeters
3 Facebook pages
6 Wikipedia pages
1 video uploader

Citations

163 Dimensions

Readers on

576 Mendeley

SUMMARY | Twitter | Facebook | Wikipedia | Video | Dimensions citations

Title	Non-pharmacological interventions for assisting the induction of anaesthesia in children
Published in	Cochrane database of systematic reviews, July 2015
DOI	10.1002/14651858.cd006447.pub3
Pubmed ID	26171895
Authors	Anne Manyande, Allan M Cyna, Peggy Yip, Cheryl Chooi, Philippa Middleton
Abstract	Induction of general anaesthesia can be distressing for children. Non-pharmacological methods for... [show]

TWITTER DEMOGRAPHICS   MENDELEY READERS

The data shown below were collected from the profiles of **11** tweeters who shared this research output. Click here

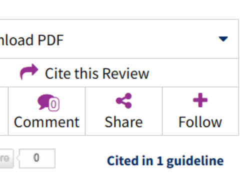

Cochrane Database of Systematic Reviews | Review - Intervention

New search

Non-pharmacological interventions for assisting the induction of anaesthesia in children

Anne Manyande, Allan M Cyna, Peggy Yip, Cheryl Chooi, Philippa Middleton   Authors' declarations of interest

Version published: 14 July 2015   Version history

https://doi.org/10.1002/14651858.CD006447.pub3

Download PDF

Cite this Review

Print | Comment | Share | Follow

0   Cited in 1 guideline

[78] Marcano Belisario, Huckvale, Greenfield, Car and Gunn (2013) Smartphone and tablet self management apps for asthma

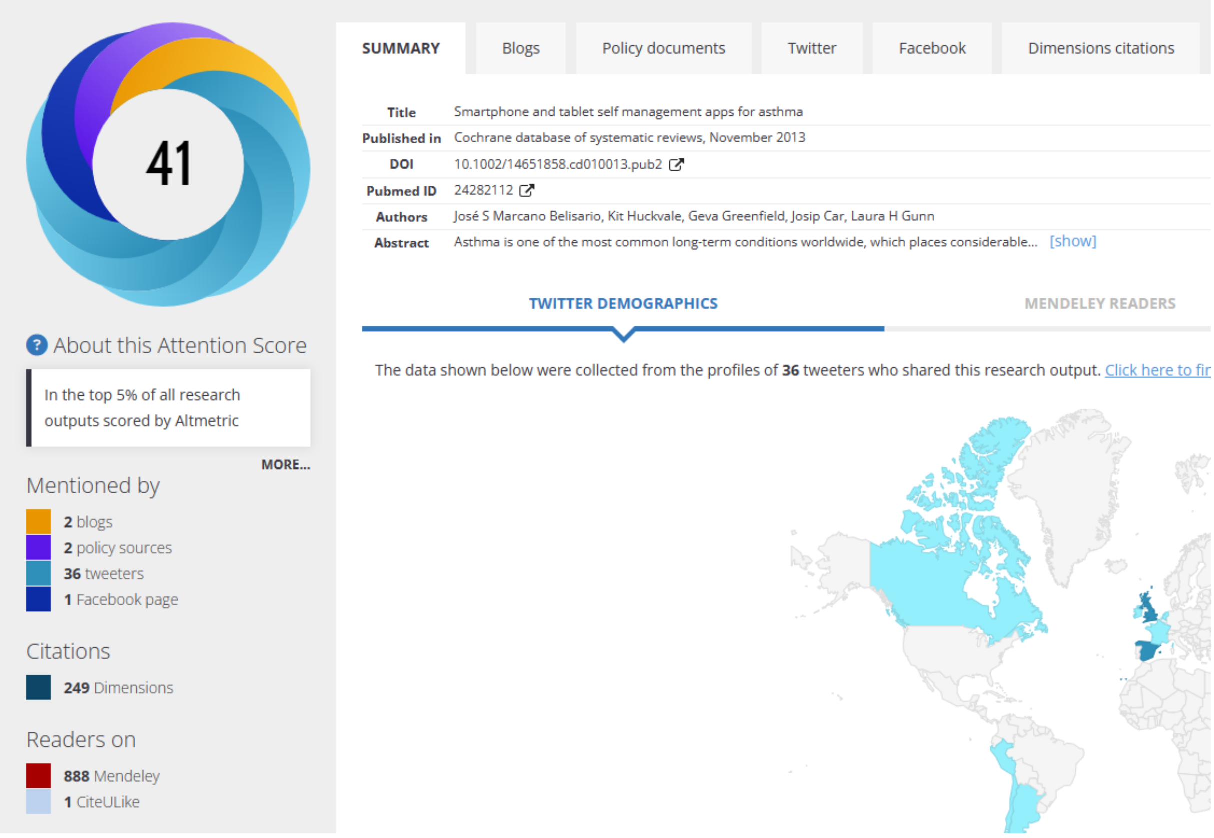

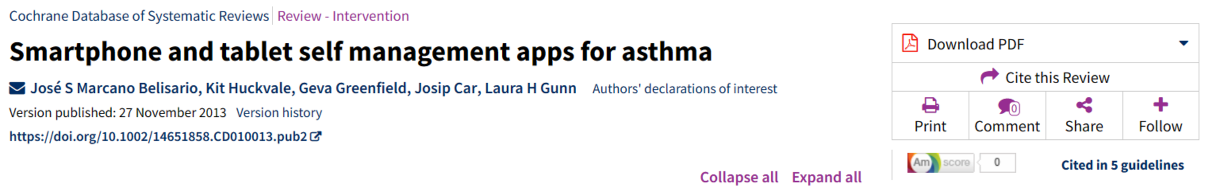

[79] Marcano Belisario, Jamsek, Huckvale, O'Donoghue, Morrison and Car (2015) Comparison of self-administered survey questionnaire responses collected using mobile apps versus other methods

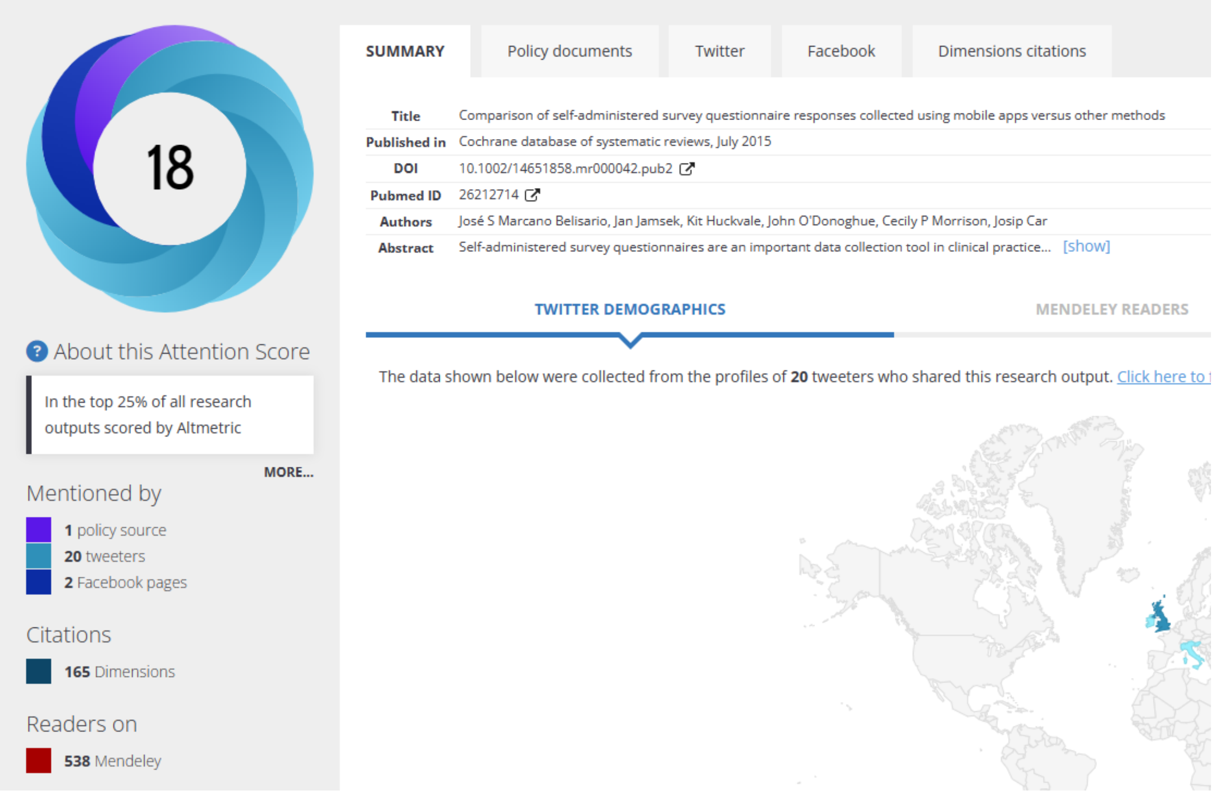

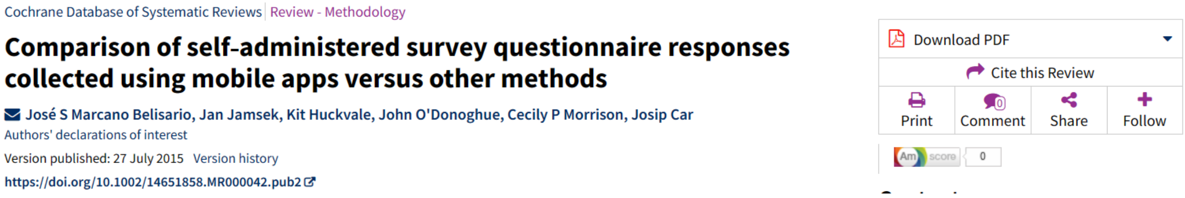

[80] Martin, Kelly, Kernohan, McCreight and Nugent (2008) Smart home technologies for health and social care support

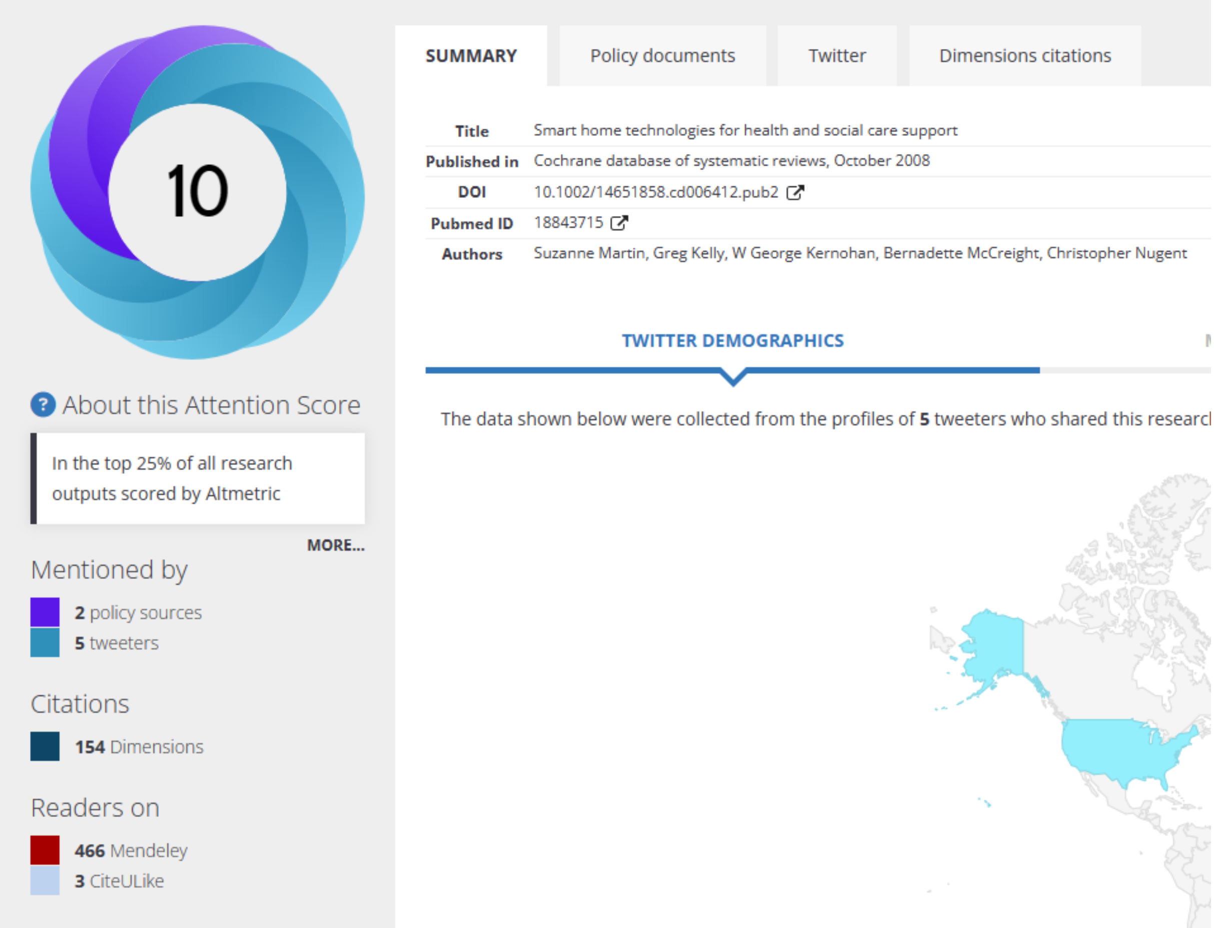

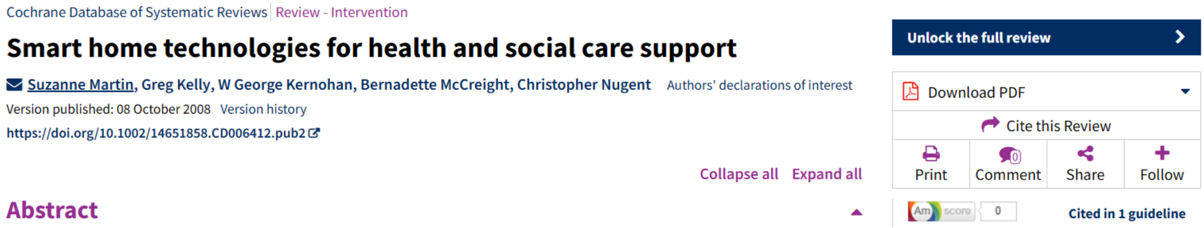

[81] Matkin, Ordóñez-Mena and Hartmann-Boyce (2019) Telephone counselling for smoking cessation

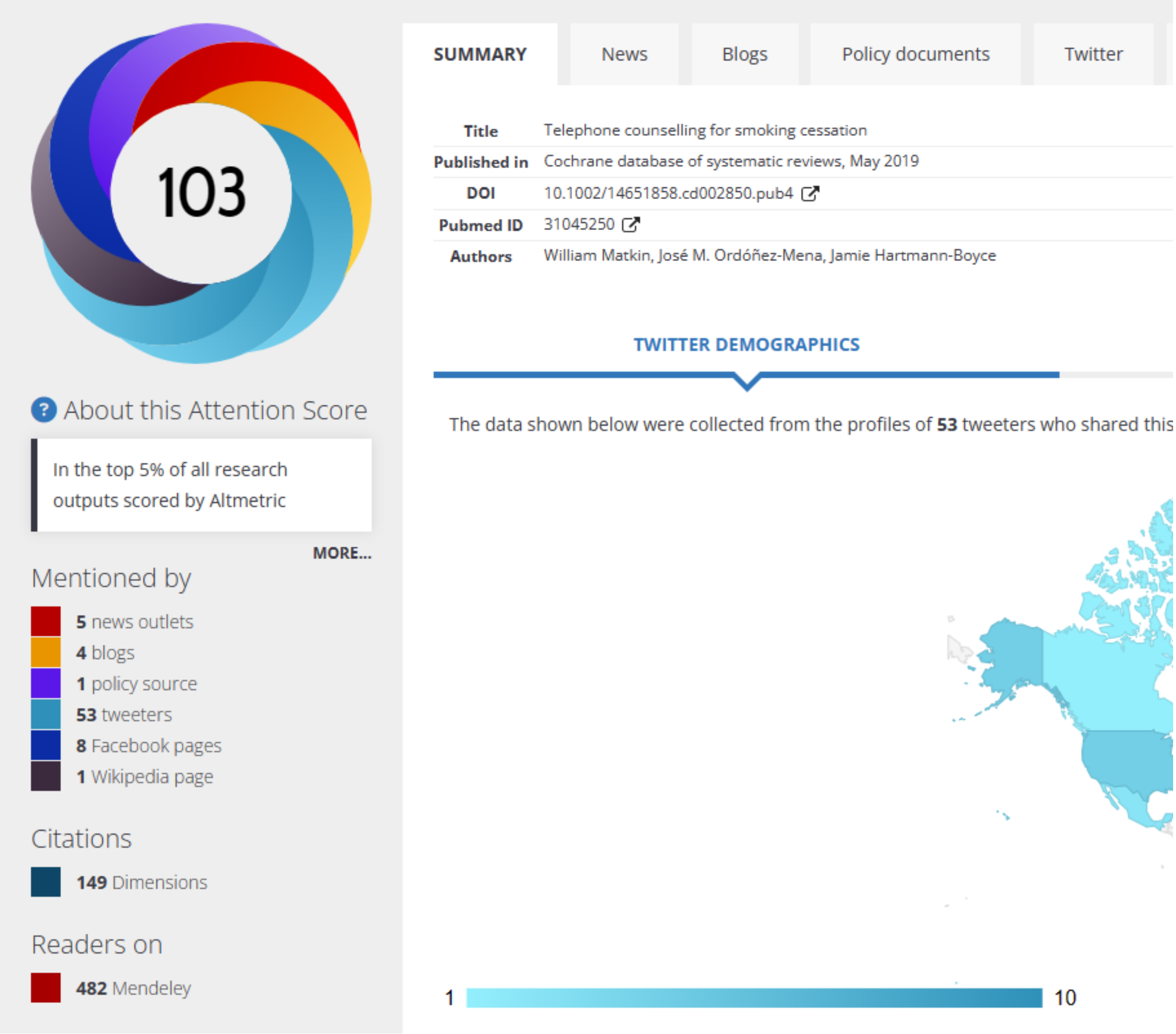

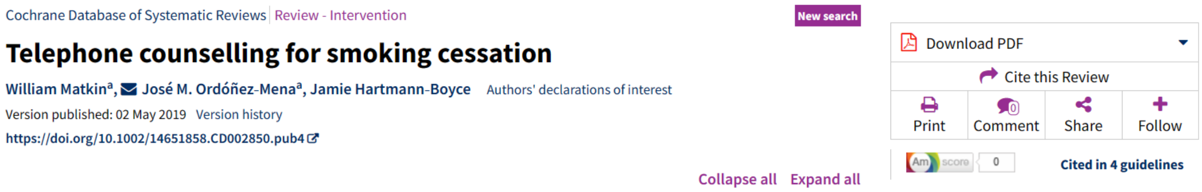

[82] Mayo-Wilson and Montgomery (2013) Media-delivered cognitive behavioural therapy and behavioural therapy (self-help) for anxiety disorders in adults

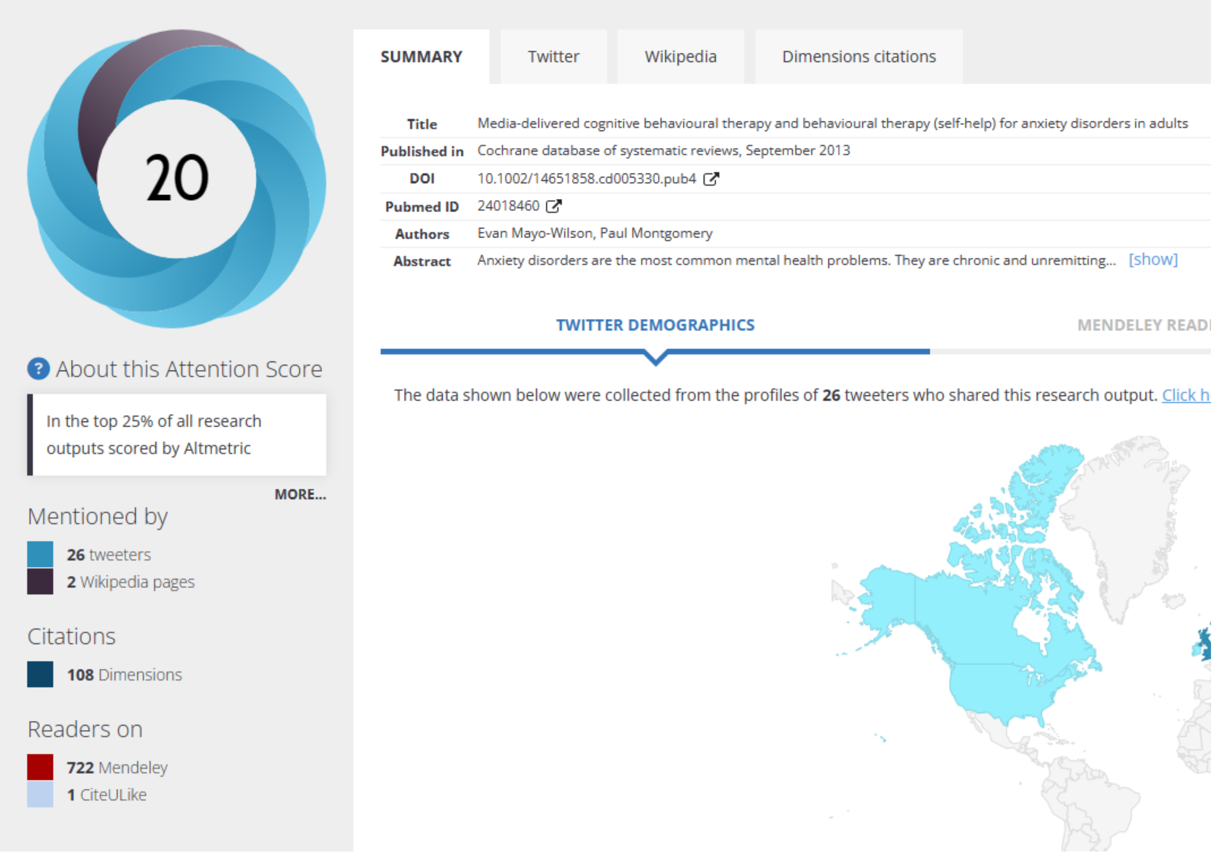

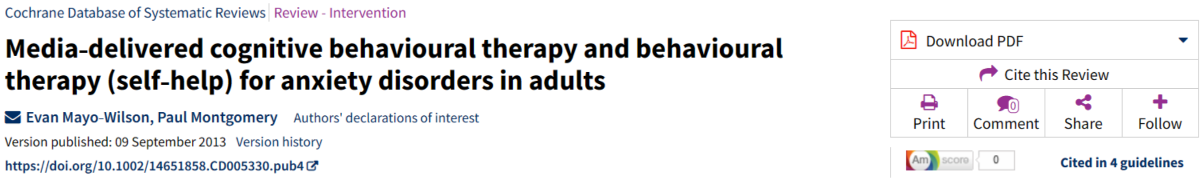

[83] McCabe, McCann and Brady (2017) Computer and mobile technology interventions for self-management in chronic obstructive pulmonary disease

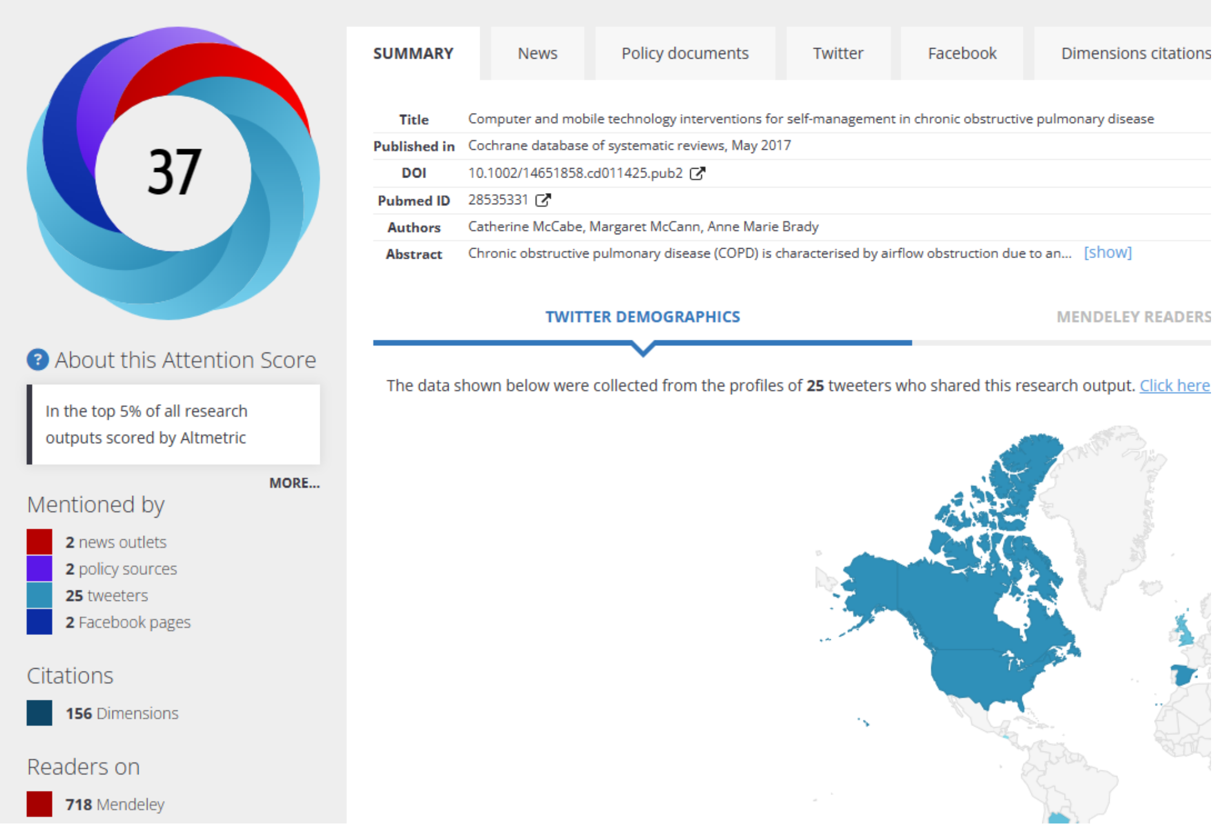

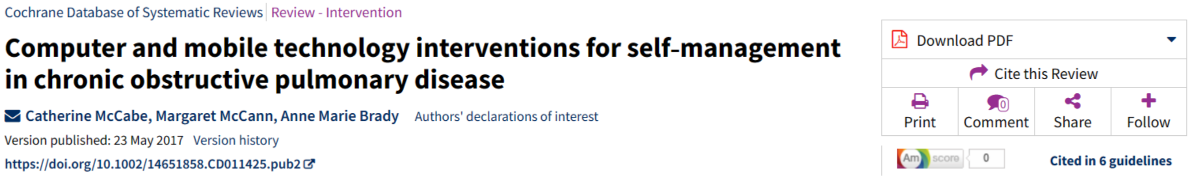

[84] McCleery, Laverty and Quinn (2021) Diagnostic test accuracy of telehealth assessment for dementia and mild cognitive impairment

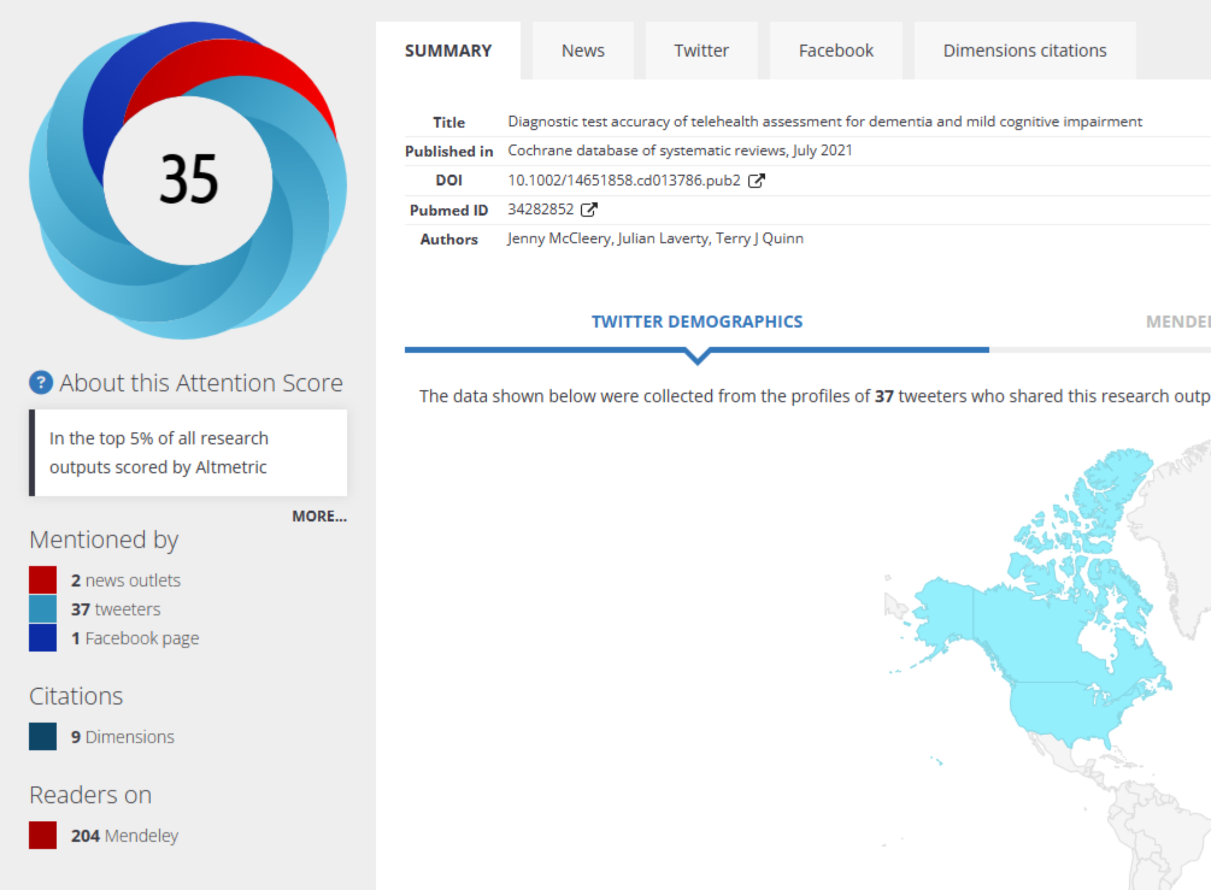

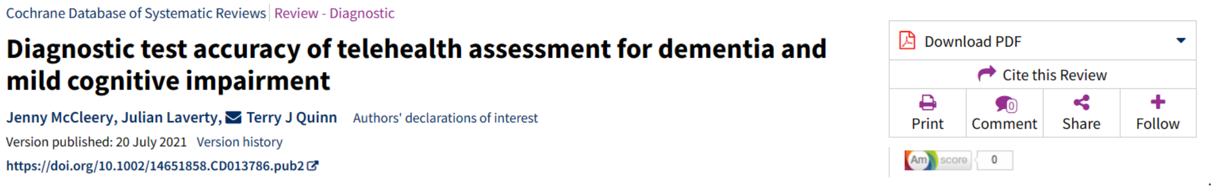

[85] McLean, Chandler, Nurmatov, Liu, Pagliari, Car and Sheikh (2010) Telehealthcare for asthma

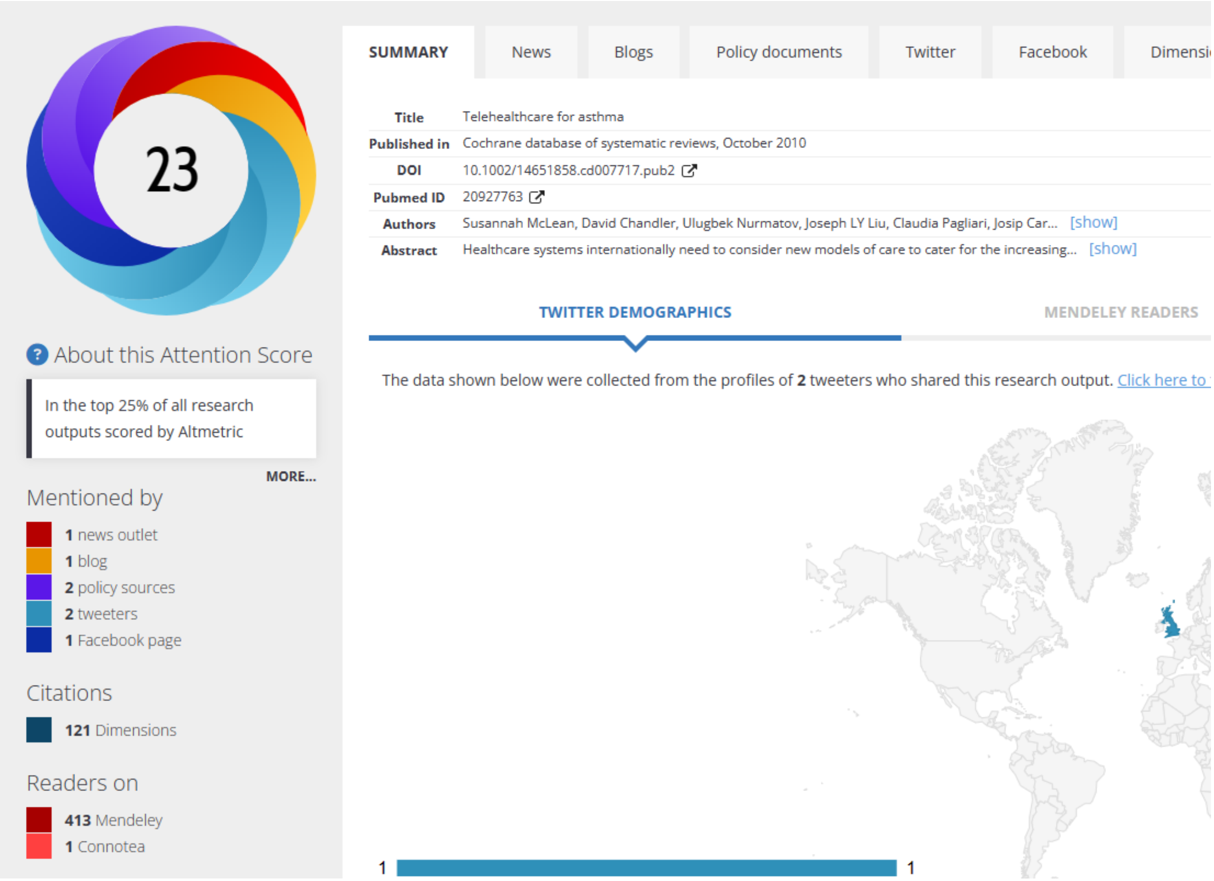

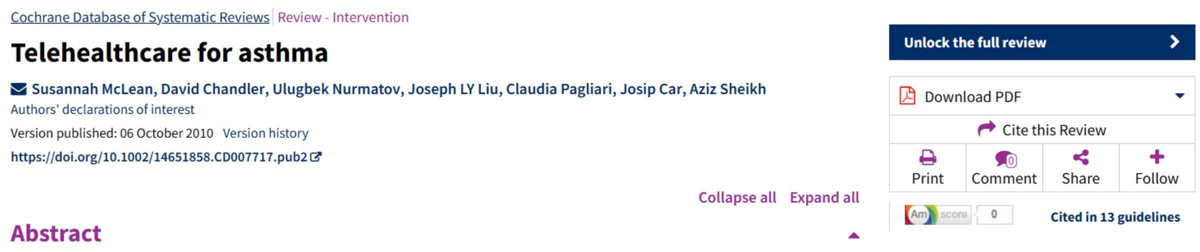

[86] McLean, Nurmatov, Liu, Pagliari, Car and Sheikh (2011) Telehealthcare for chronic obstructive pulmonary disease

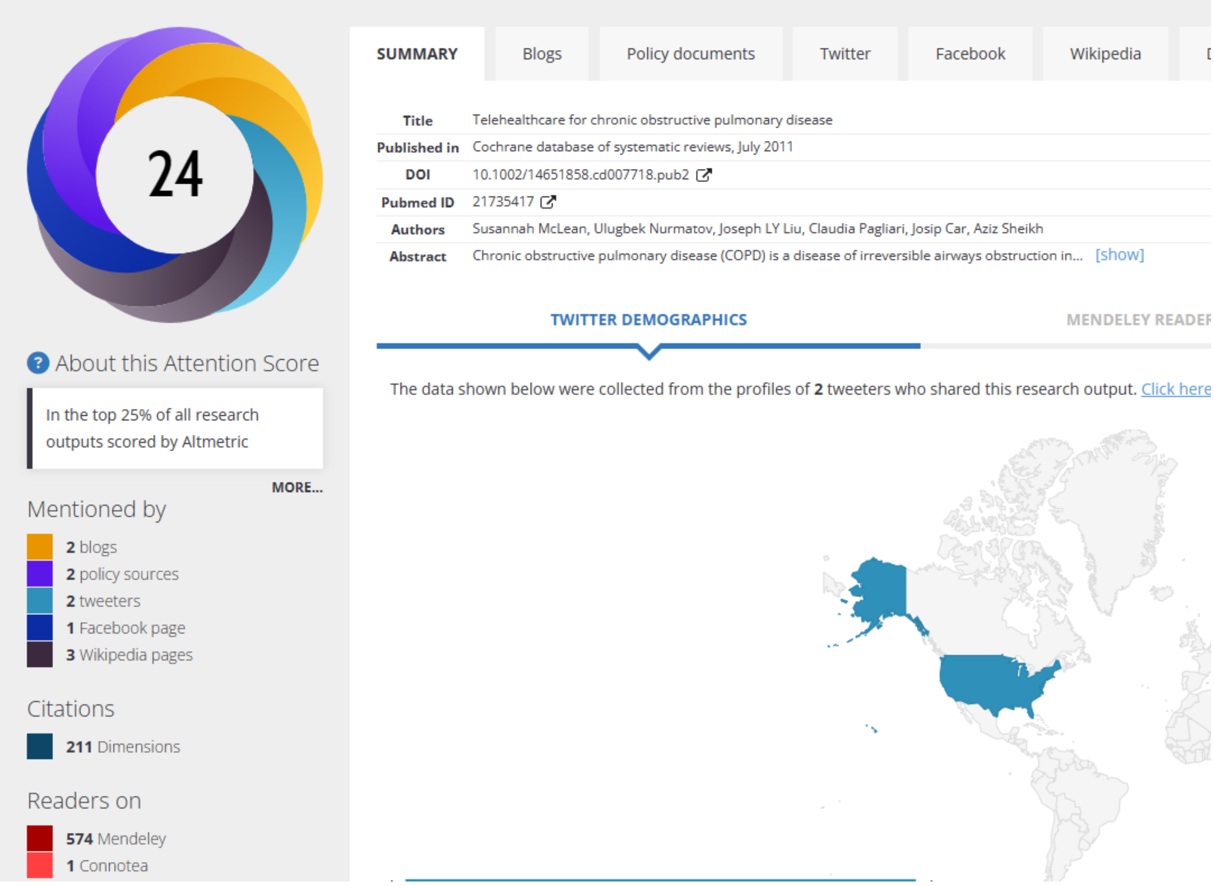

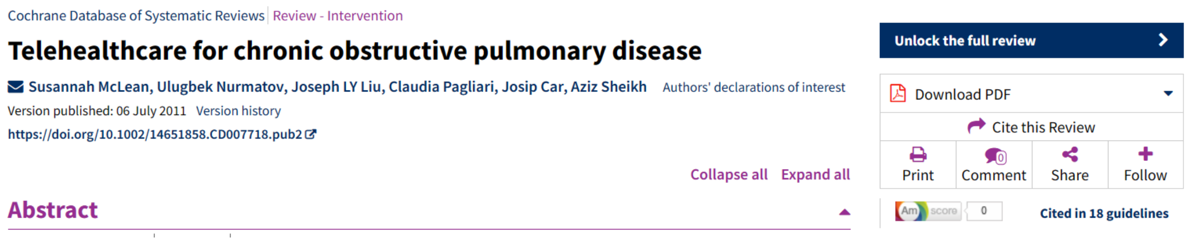

[87] Meyer, Atherton, Sawmynaden and Car (2012) Email for communicating results of diagnostic medical investigations to patients

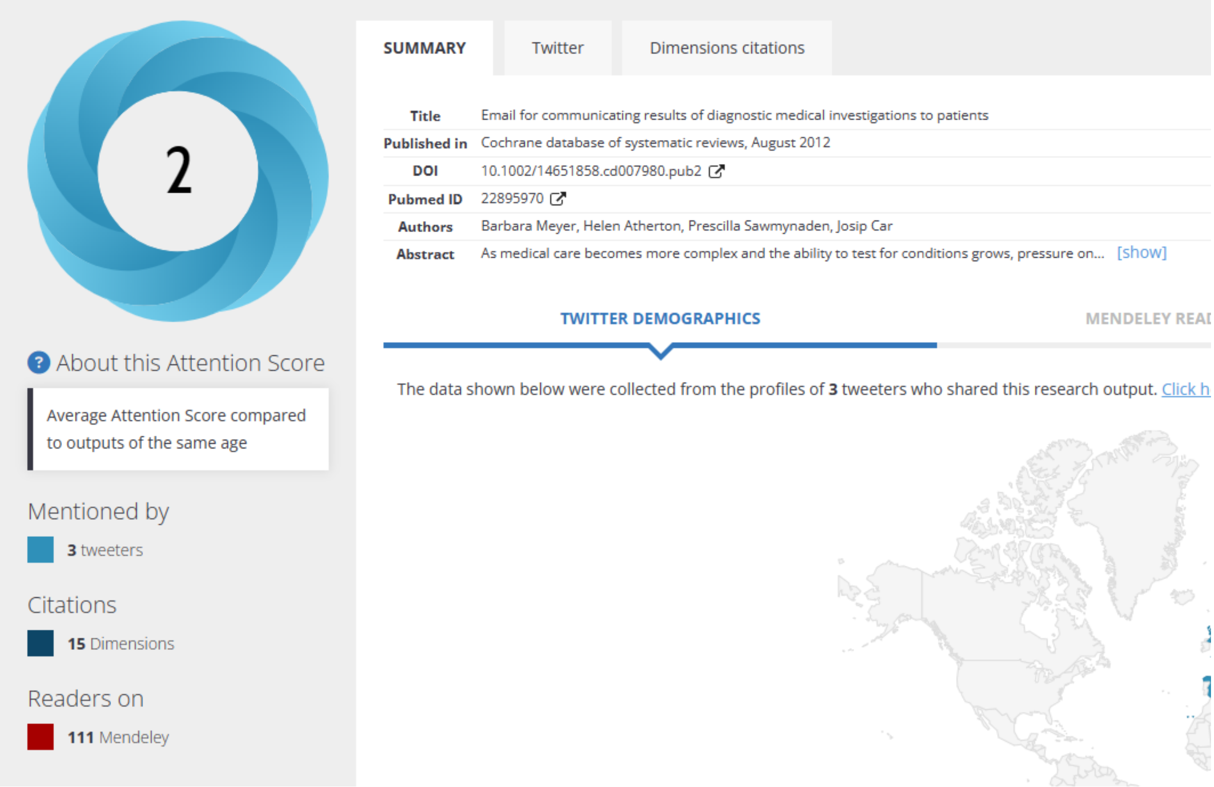

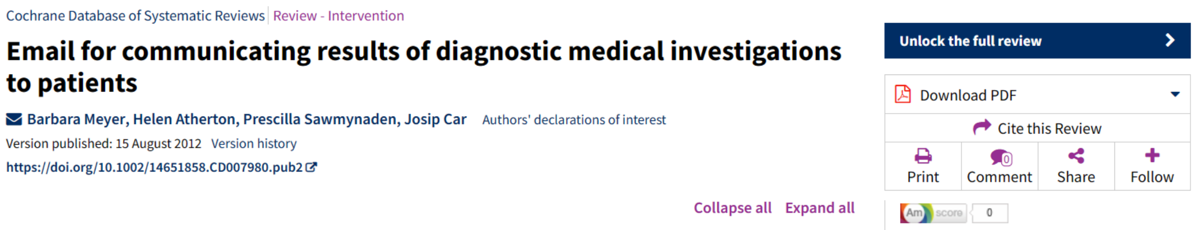

[88] Mistiaen and Poot (2006) Telephone follow-up, initiated by a hospital-based health professional, for postdischarge problems in patients discharged from hospital to home

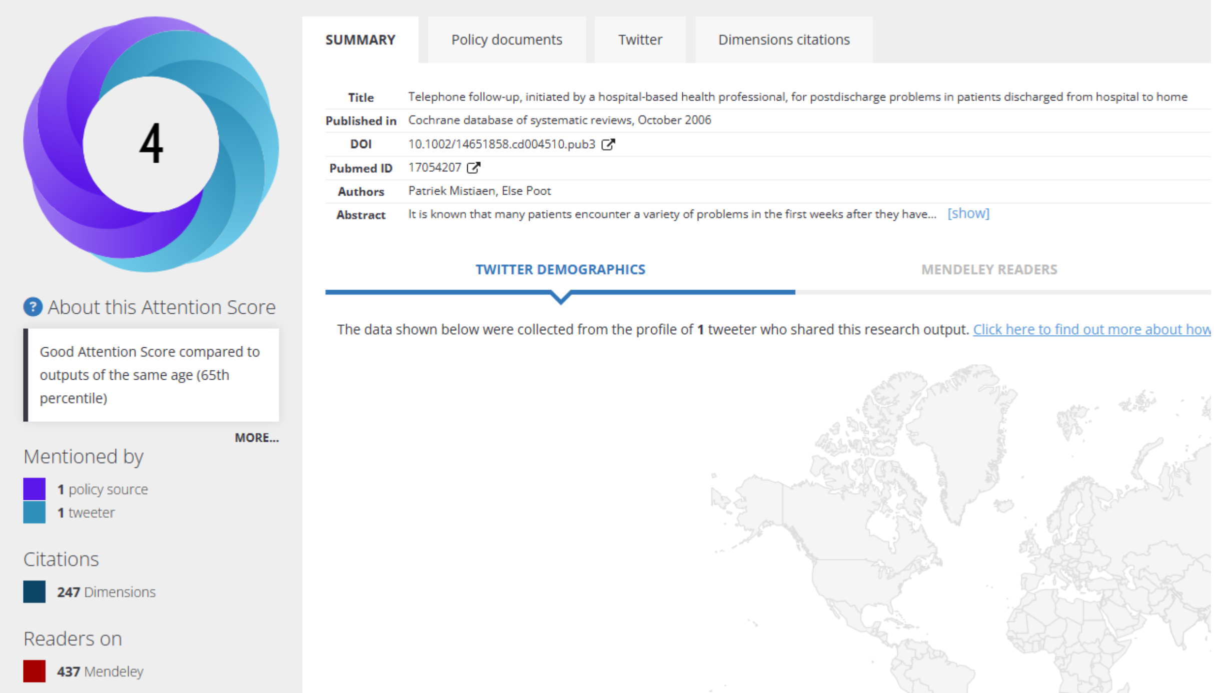

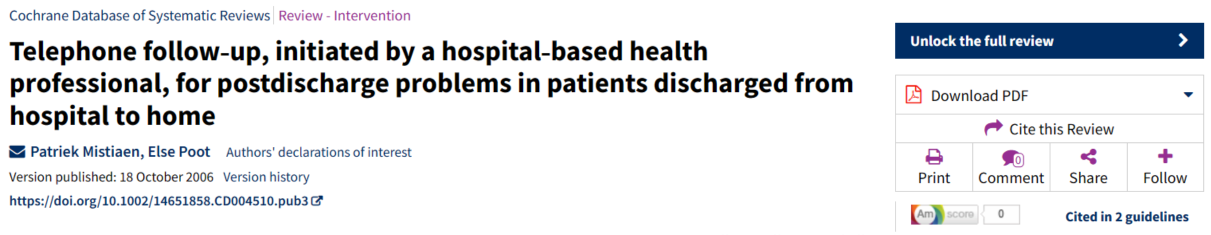

[89] Murray, Burns, See Tai, Lai and Nazareth (2005) Interactive Health Communication Applications for people with chronic disease

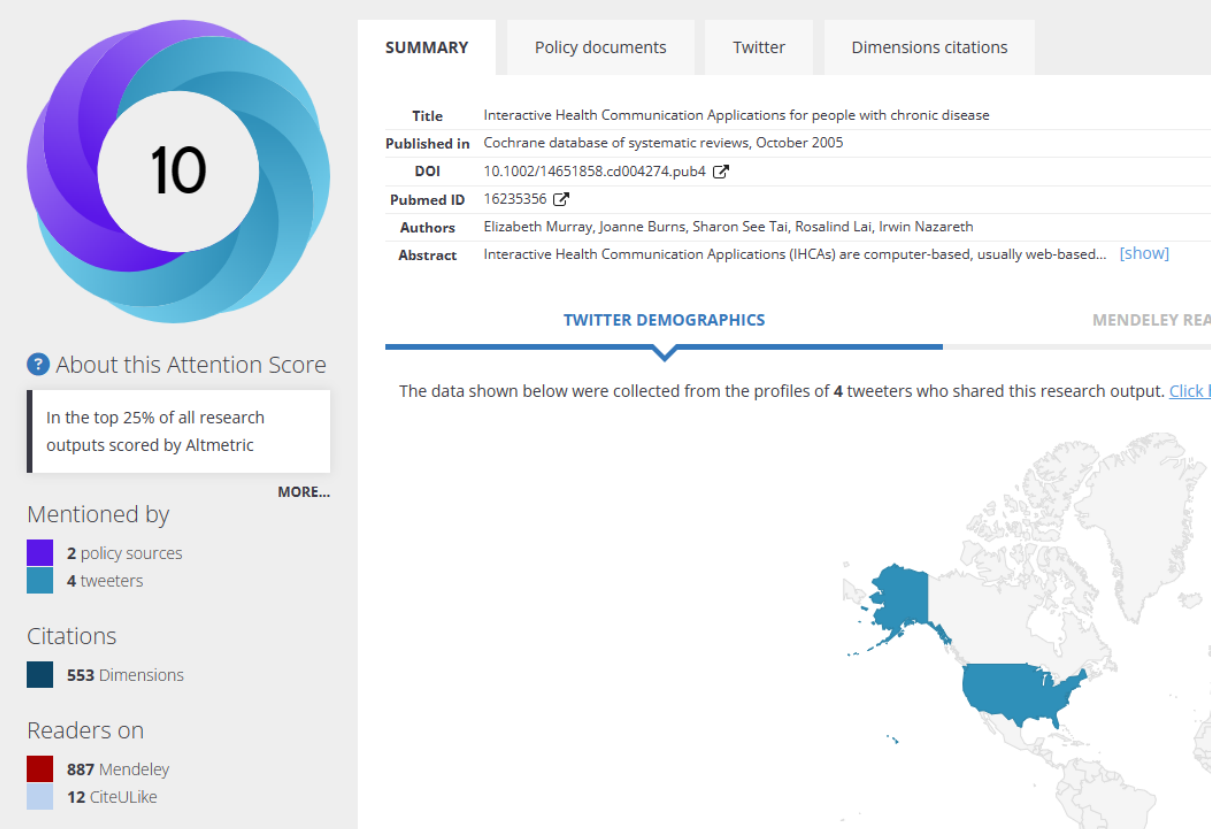

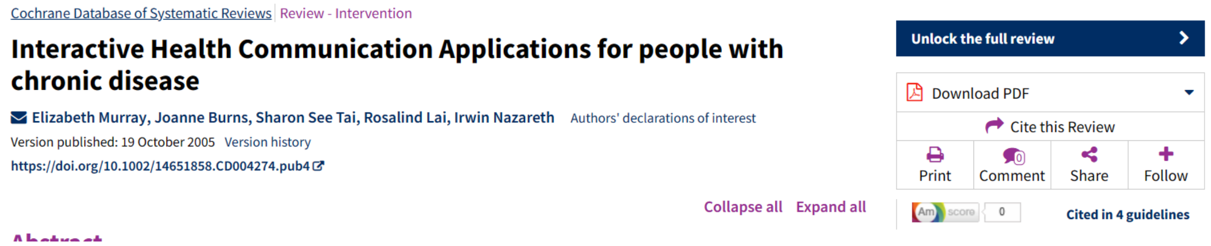

[90] Murtagh, Murphy, Milton, Roberts, O'Gorman and Foster (2020) Interventions outside the workplace for reducing sedentary behaviour in adults under 60 years of age

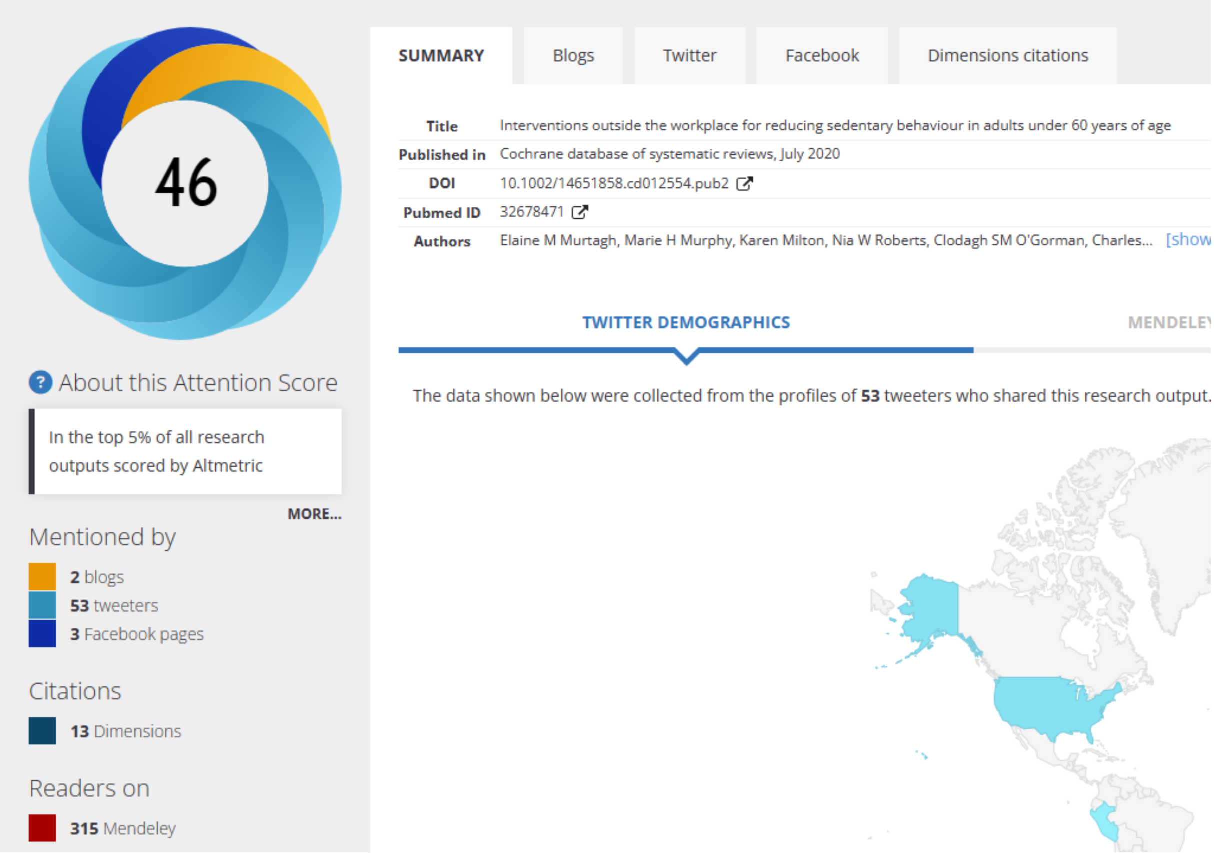

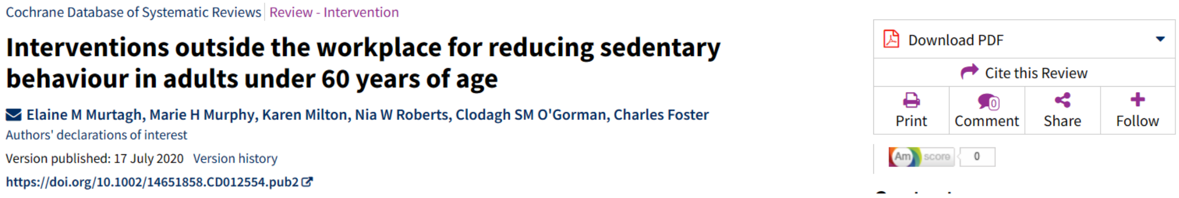

[91] Nagendran, Gurusamy, Aggarwal, Loizidou and Davidson (2013) Virtual reality training for surgical trainees in laparoscopic surgery

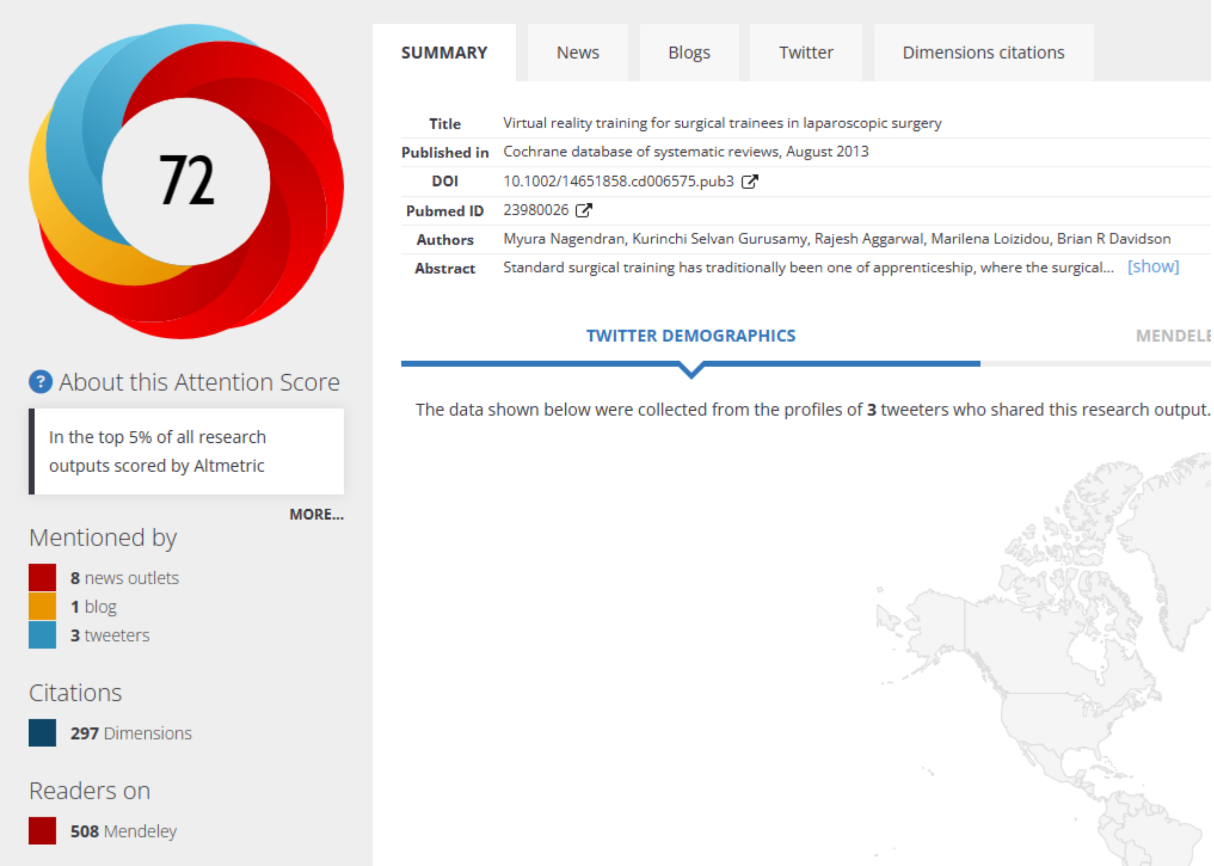

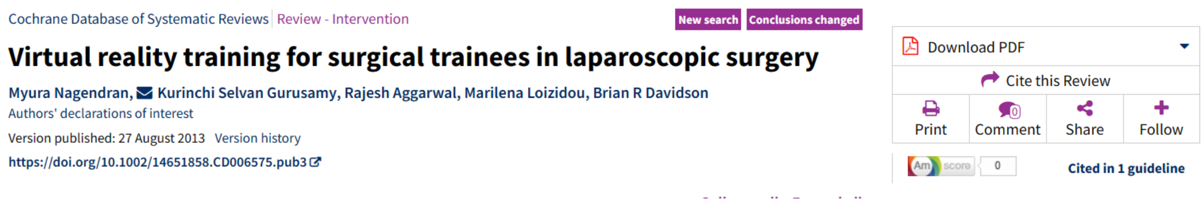

[92] Noone, McSharry, Smalle, Burns, Dwan, Devane and Morrissey (2020) Video calls for reducing social isolation and loneliness in older people: a rapid review

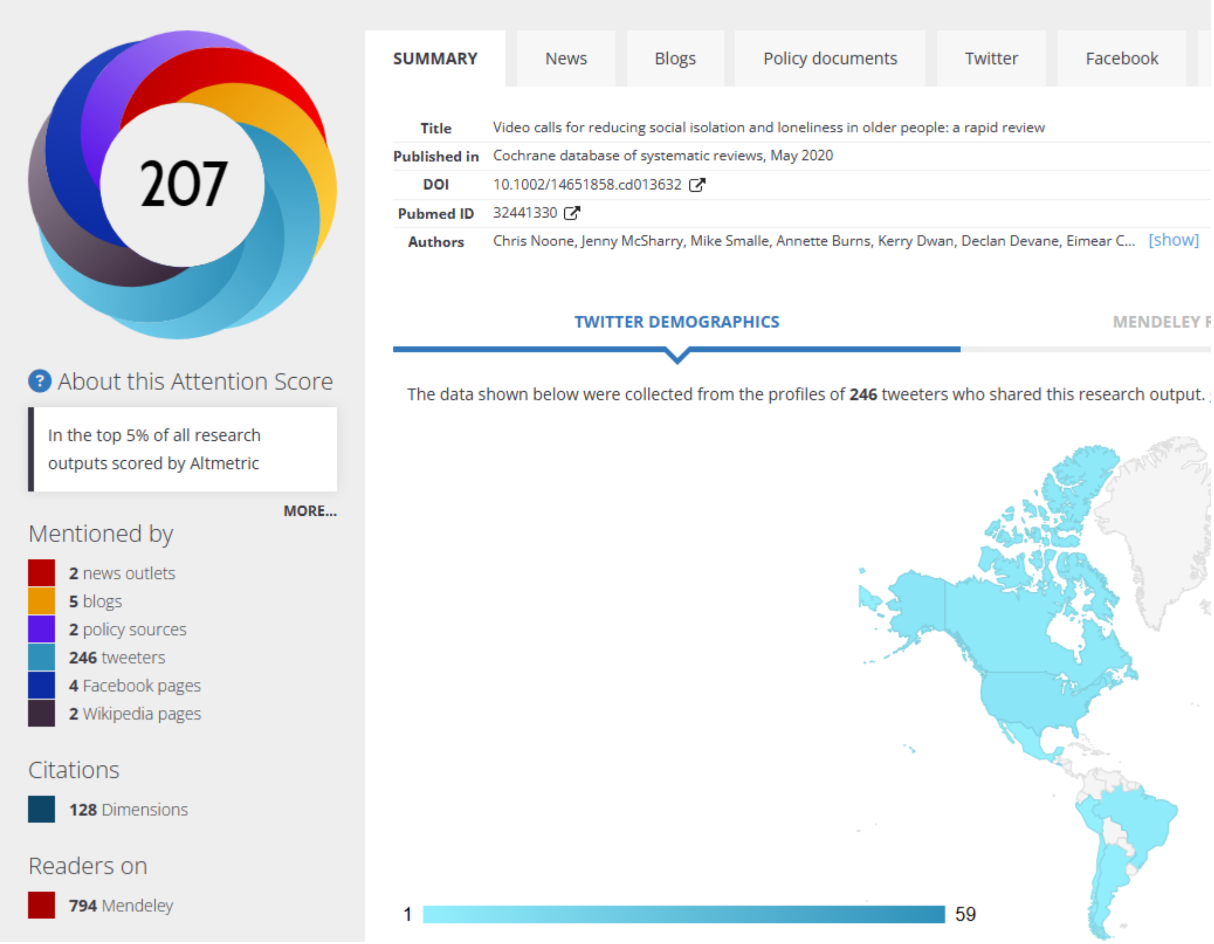

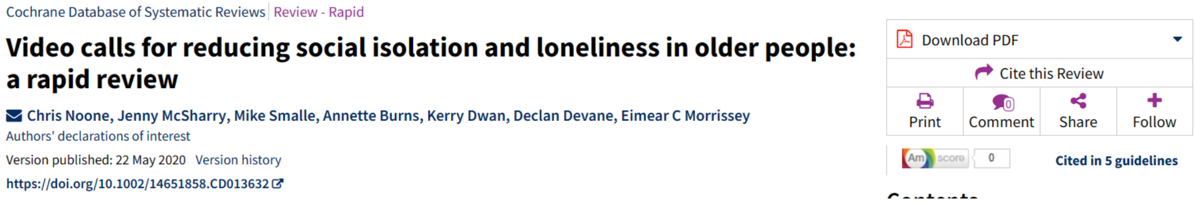

[93] Odendaal, Anstey Watkins, Leon, Goudge, Griffiths, Tomlinson and Daniels (2020) Health workers' perceptions and experiences of using mHealth technologies to deliver primary healthcare services: a qualitative evidence synthesis

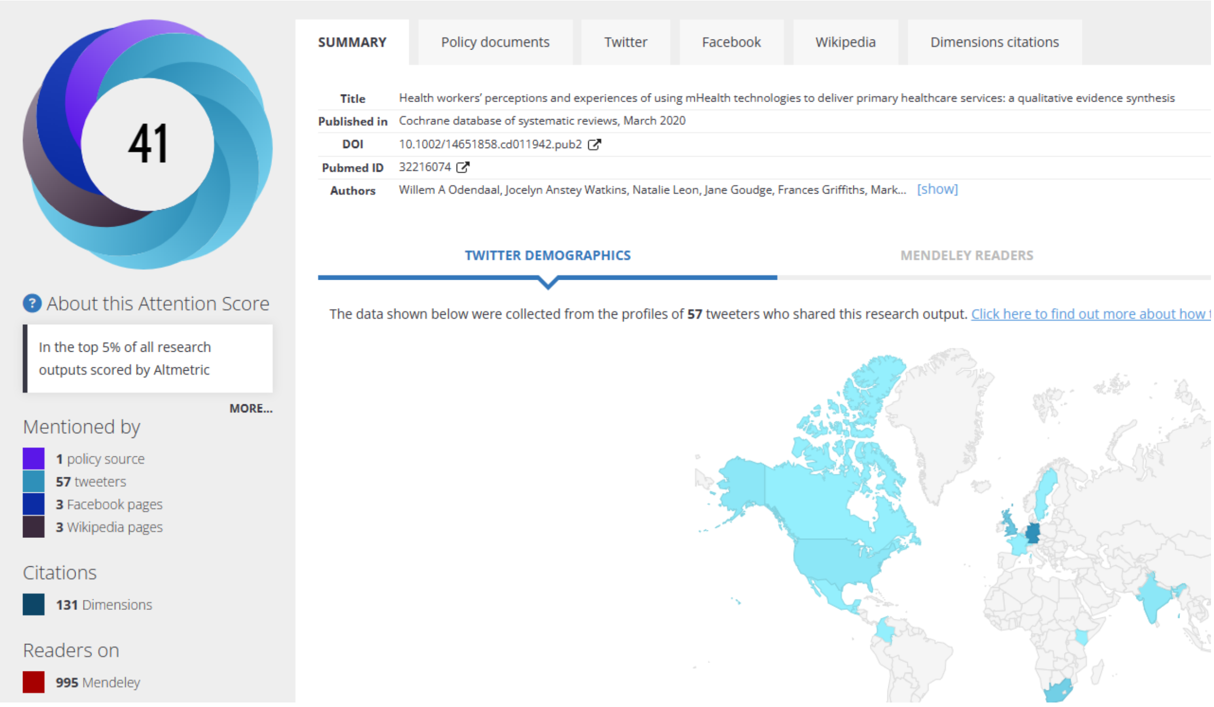

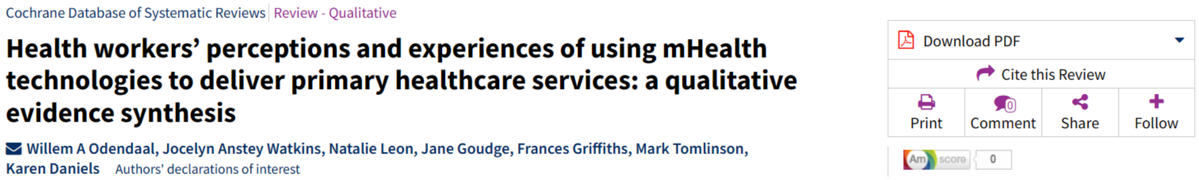

[94] Olthuis, Watt, Bailey, Hayden and Stewart (2016) Therapist-supported Internet cognitive behavioural therapy for anxiety disorders in adults

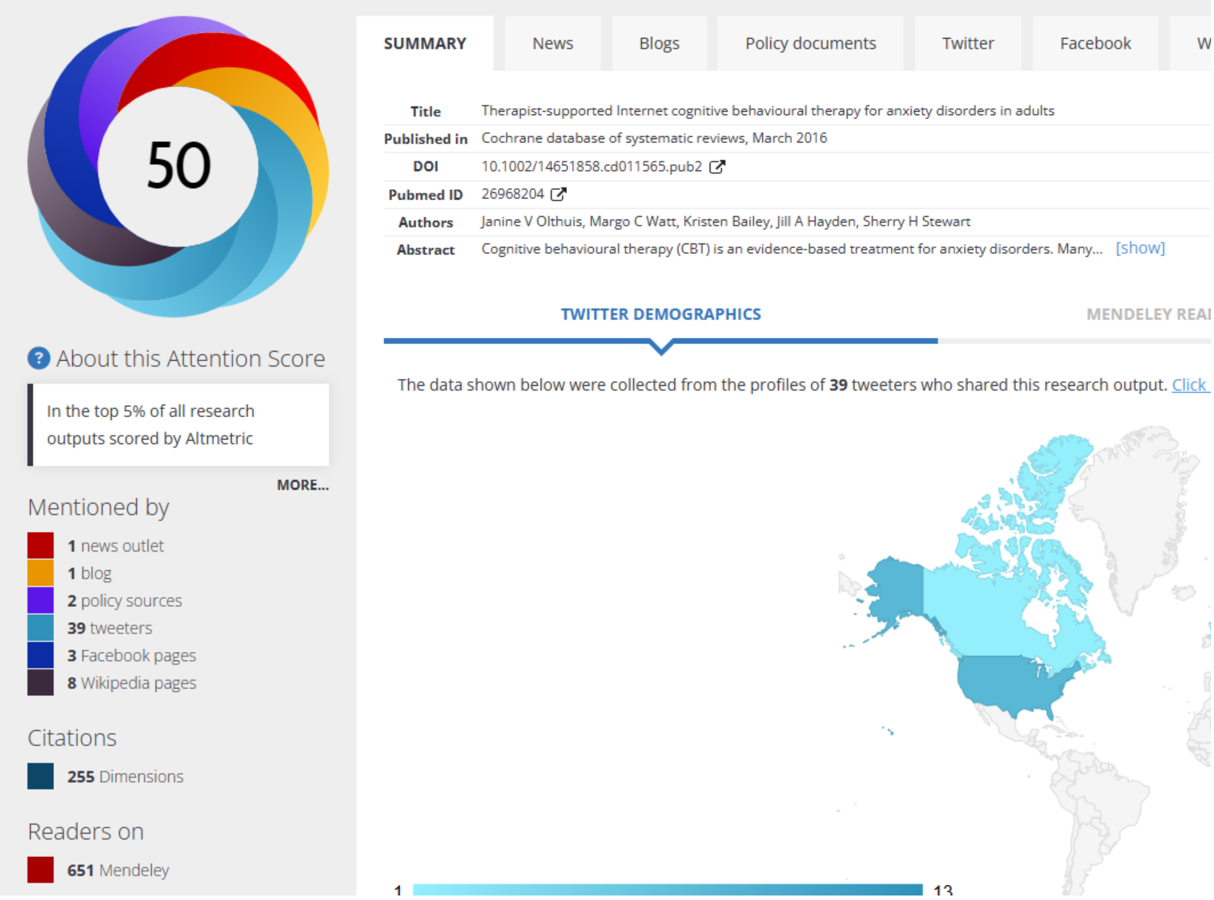

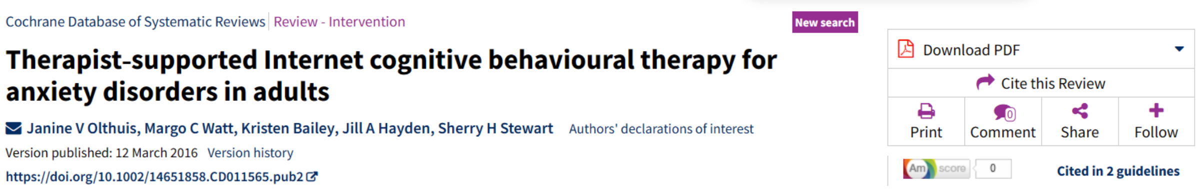

[95] Pal, Eastwood, Michie, Farmer, Barnard, Peacock, Wood, Inniss and Murray (2013) Computer-based diabetes self-management interventions for adults with type 2 diabetes mellitus

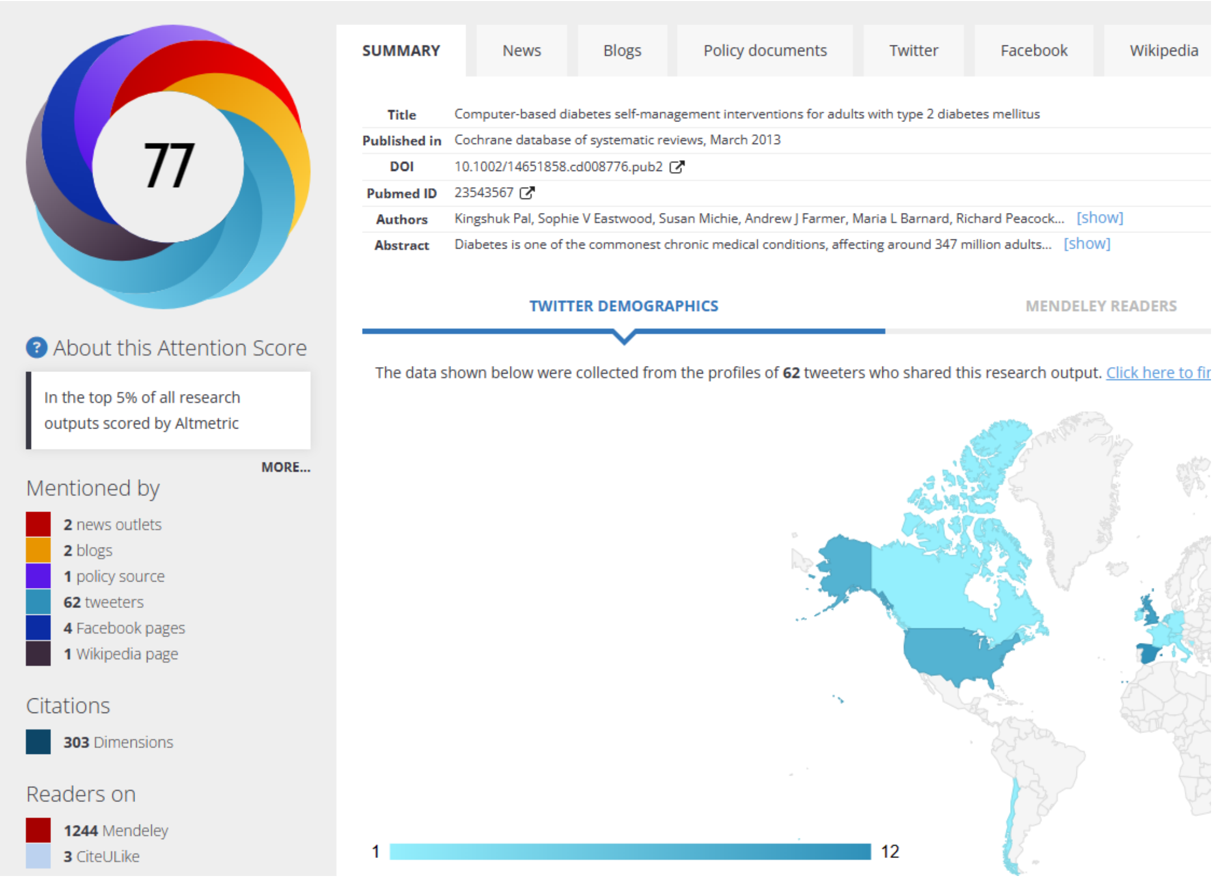

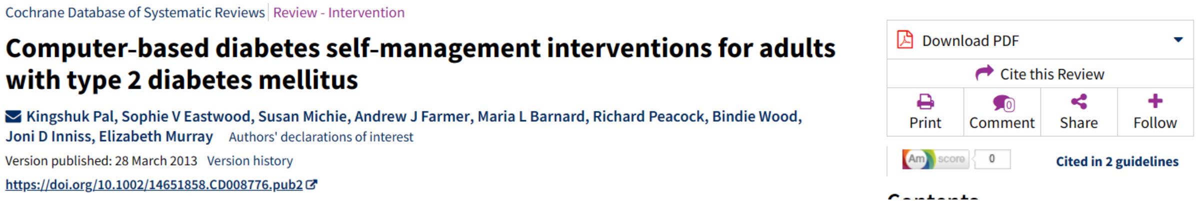

[96] Palmer, Henschke, Bergman, Villanueva, Maayan, Tamrat, Mehl, Glenton, Lewin, Fønhus and et al. (2020) Targeted client communication via mobile devices for improving maternal, neonatal, and child health

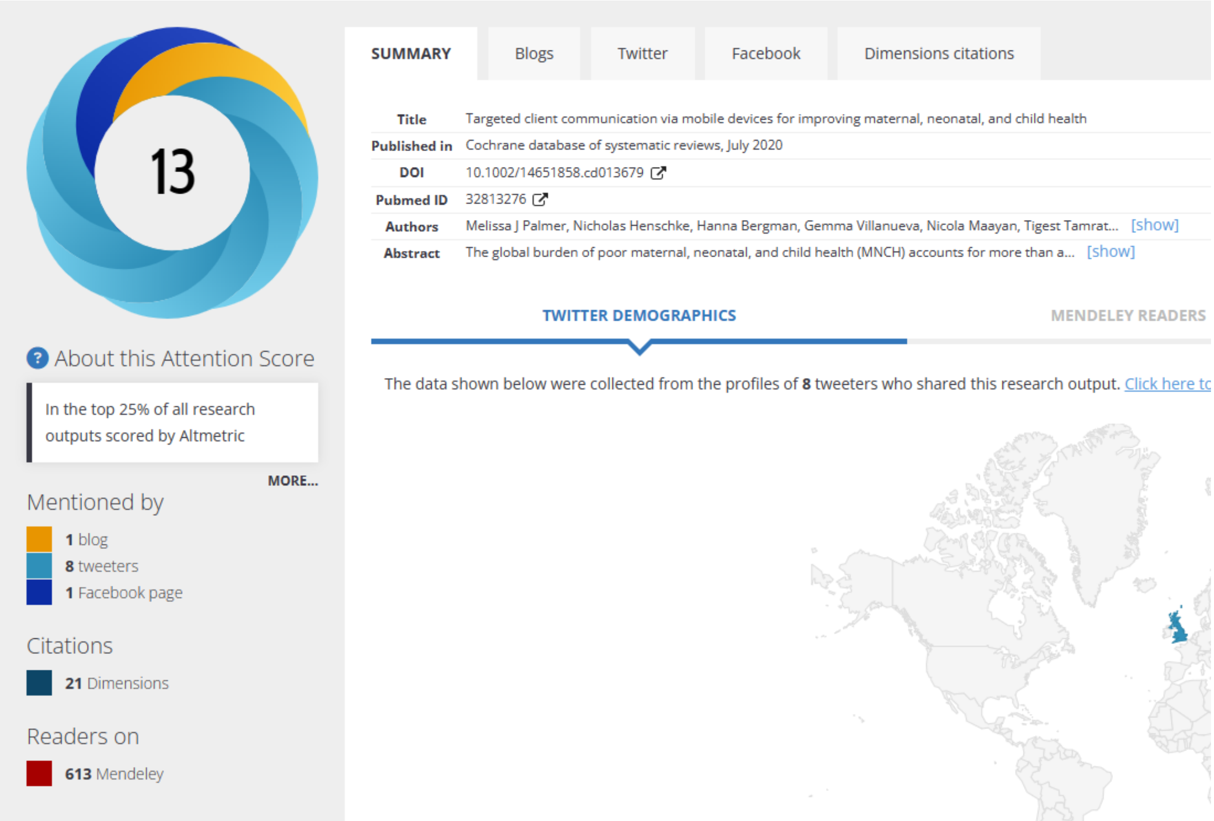

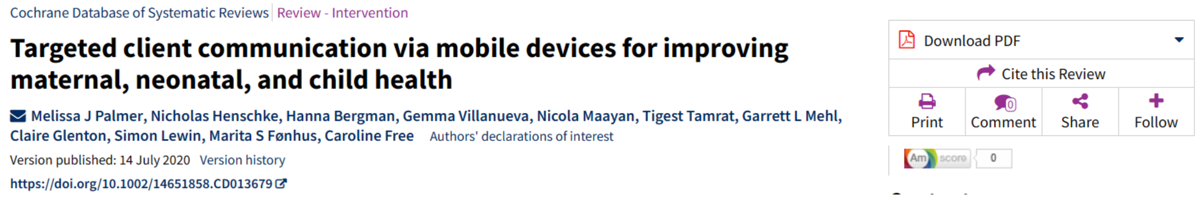

[97] Palmer, Henschke, Villanueva, Maayan, Bergman, Glenton, Lewin, Fønhus, Tamrat, Mehl and et al. (2020) Targeted client communication via mobile devices for improving sexual and reproductive health

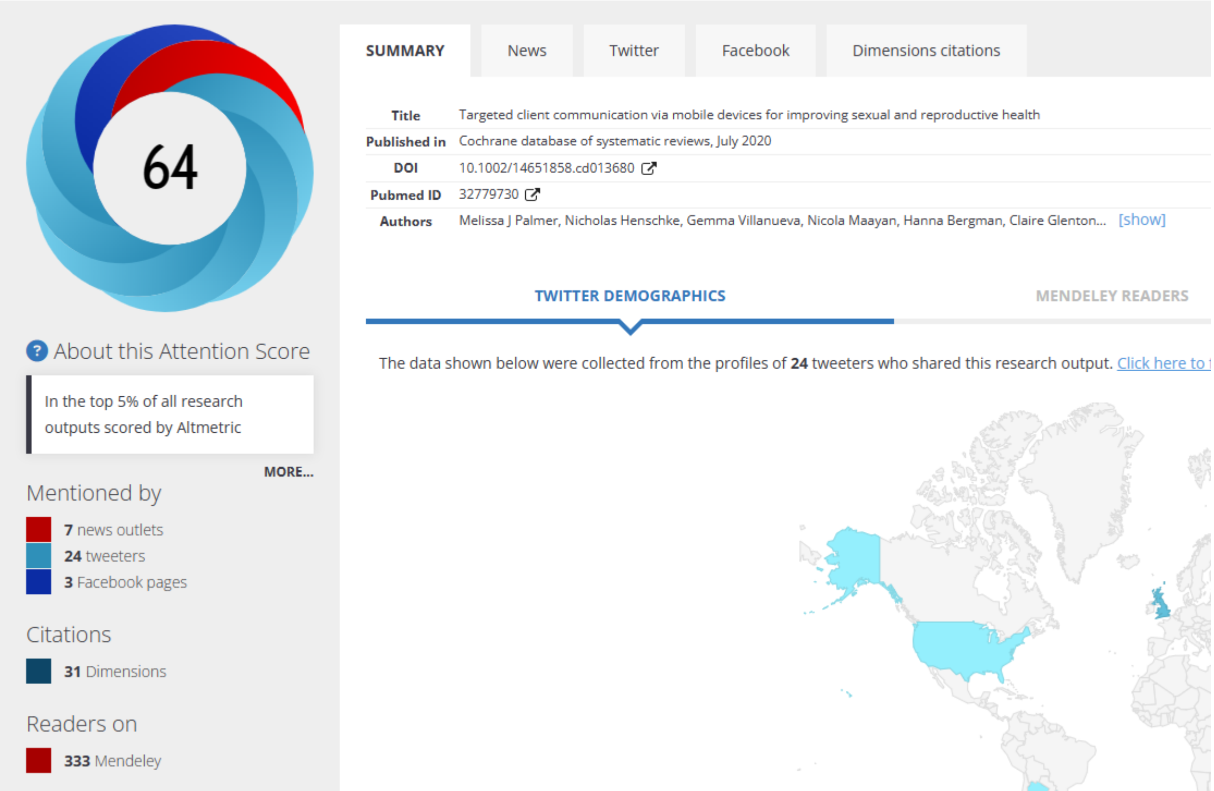

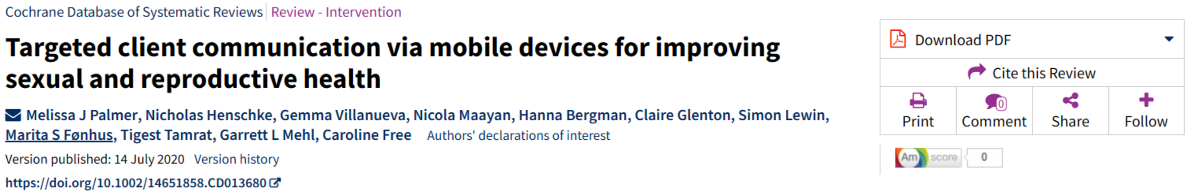

[98] Palmer, Machiyama, Woodd, Gubijev, Barnard, Russell, Perel and Free (2021) Mobile phone-based interventions for improving adherence to medication prescribed for the primary prevention of cardiovascular disease in adults

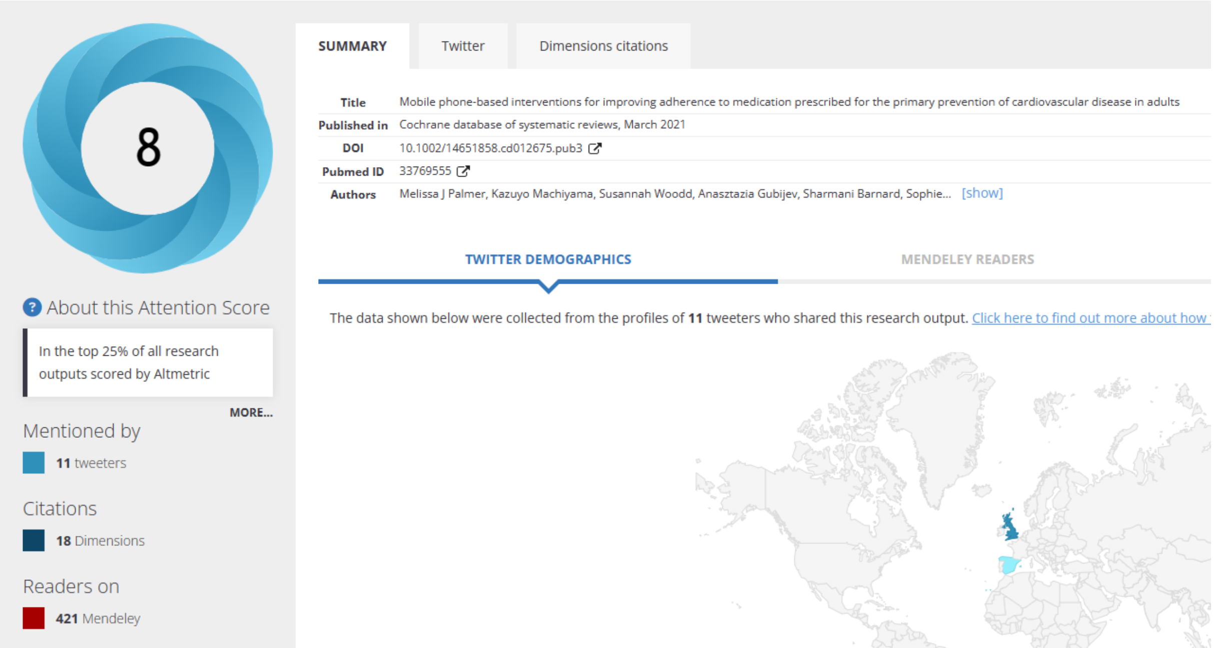

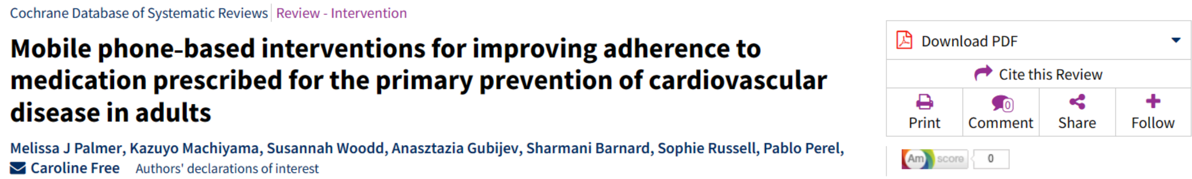

[99] Pappas, Wei, Car, Majeed and Sheikh (2011) Computer-assisted versus oral-and-written family history taking for identifying people with elevated risk of type 2 diabetes mellitus

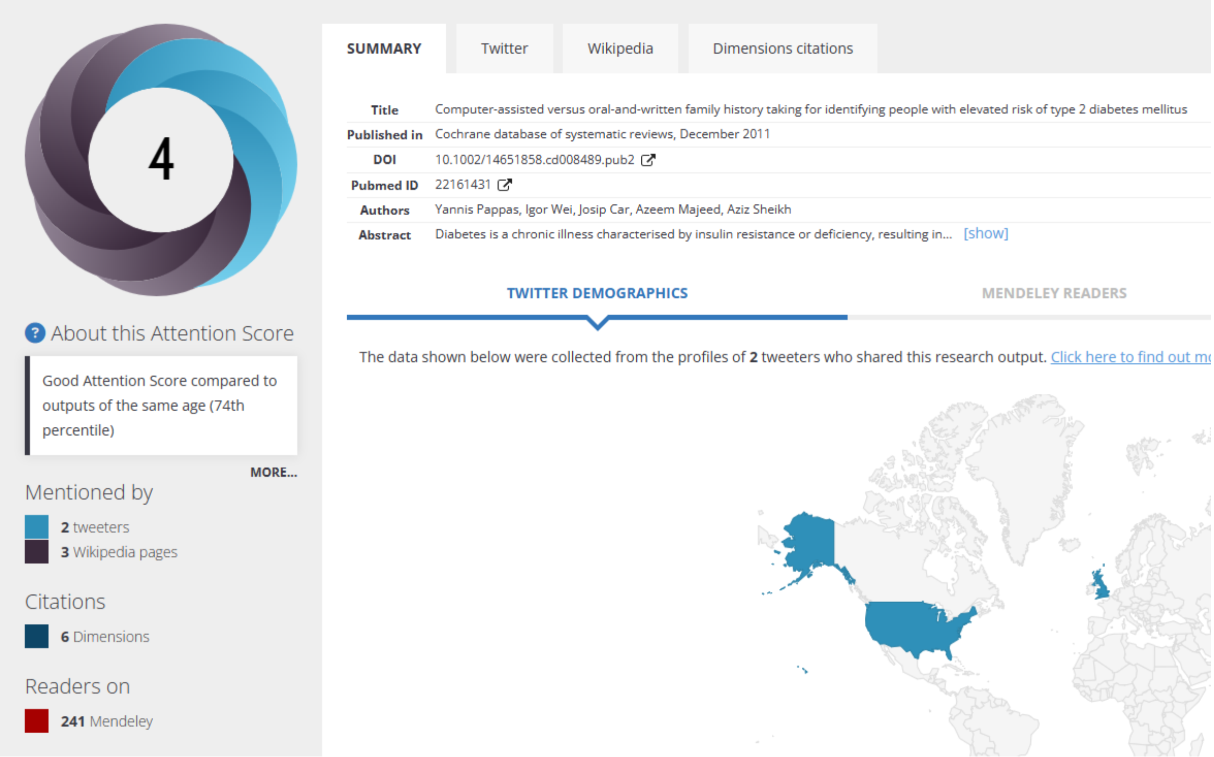

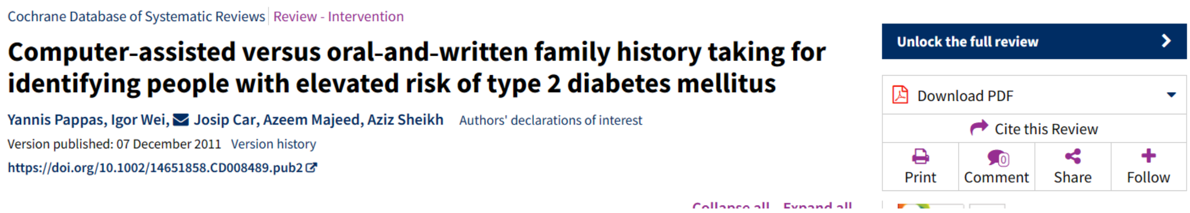

[100] Petkovic, Duench, Trawin, Dewidar, Pardo Pardo, Simeon, DesMeules, Gagnon, Hatcher Roberts, Hossain and et al. (2021) Behavioural interventions delivered through interactive social media for health behaviour change, health outcomes, and health equity in the adult population

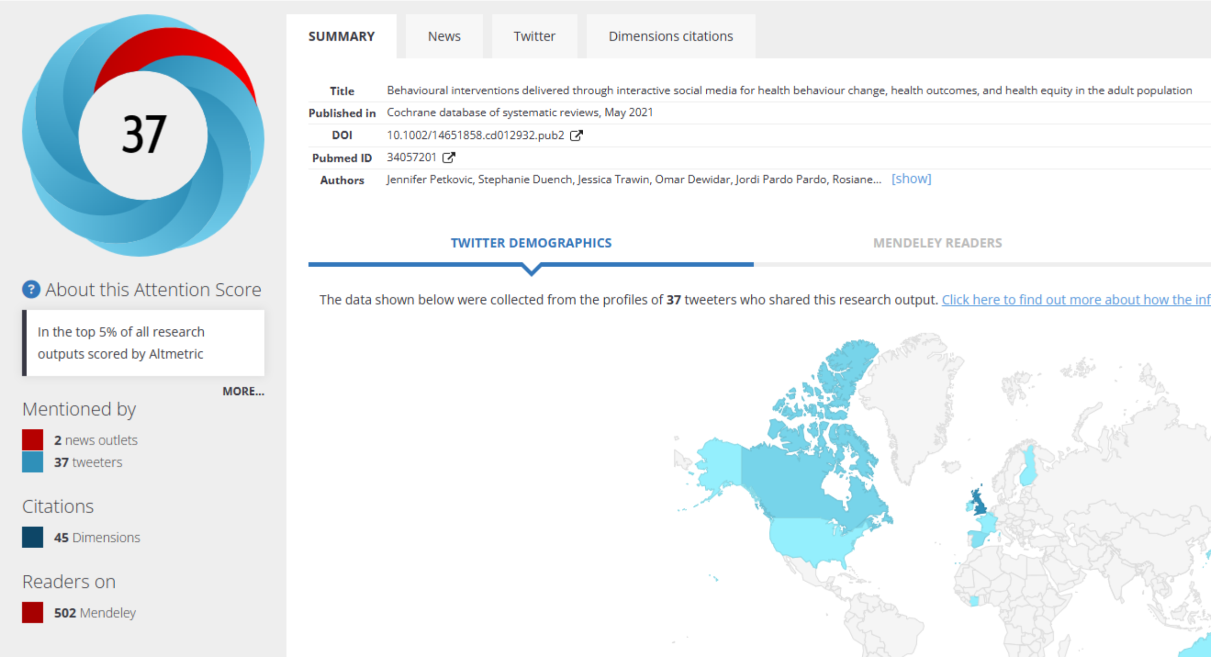

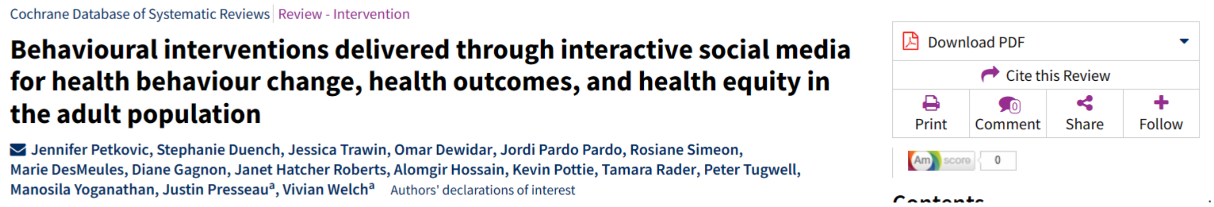

[101] Piromchai, Avery, Laopaiboon, Kennedy and O'Leary (2015) Virtual reality training for improving the skills needed for performing surgery of the ear, nose or throat

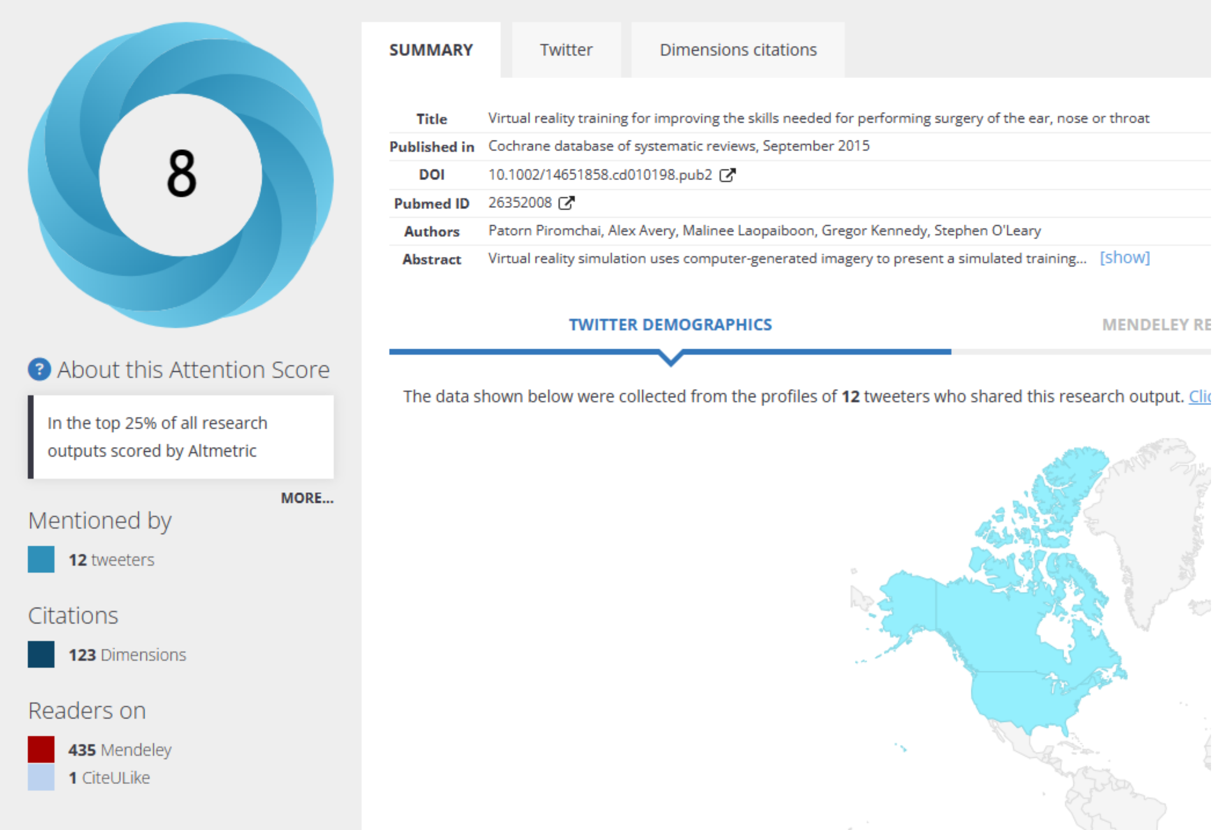

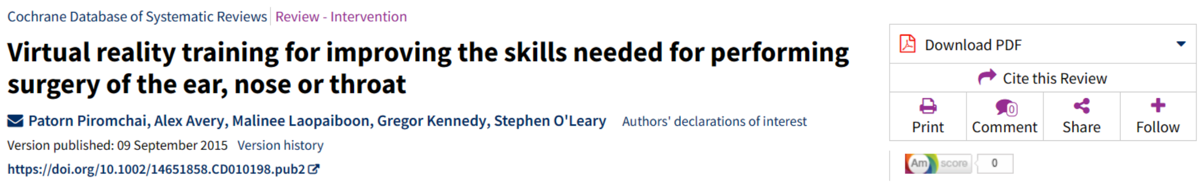

[102] Pollock, Farmer, Brady, Langhorne, Mead, Mehrholz and van Wijck (2014) Interventions for improving upper limb function after stroke

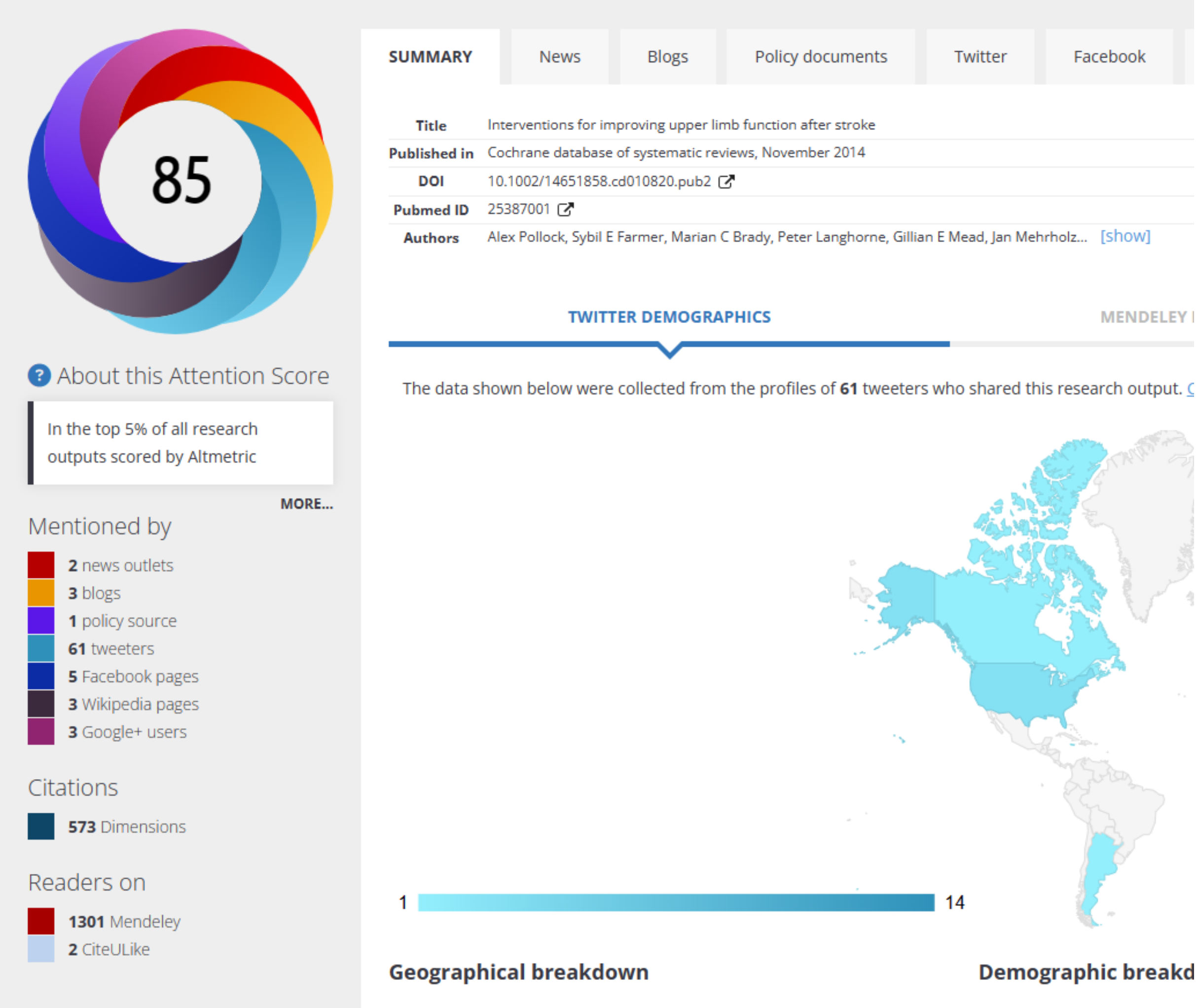

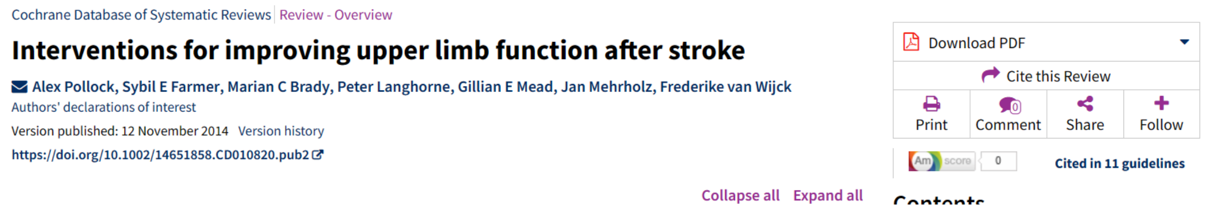

[103] Posadzki, Mastellos, Ryan, Gunn, Felix, Pappas, Gagnon, Julious, Xiang, Oldenburg and et al. (2016) Automated telephone communication systems for preventive healthcare and management of long-term conditions

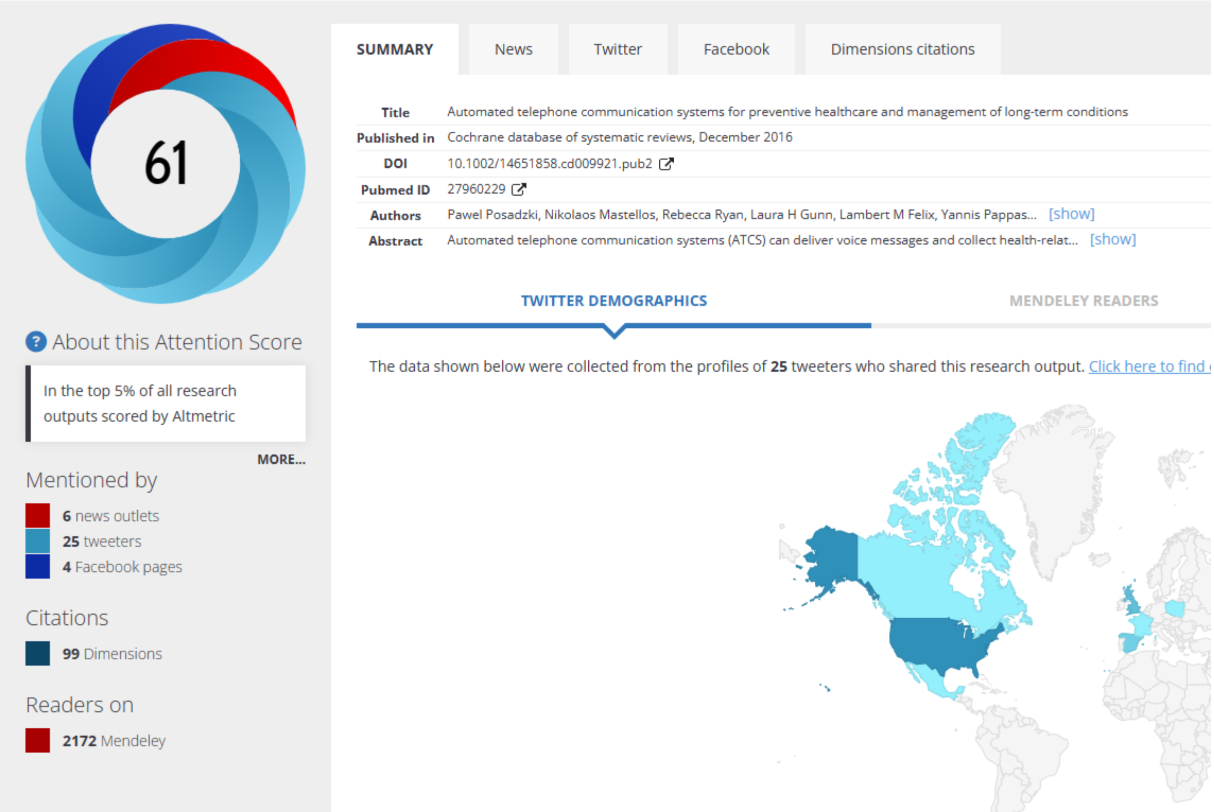

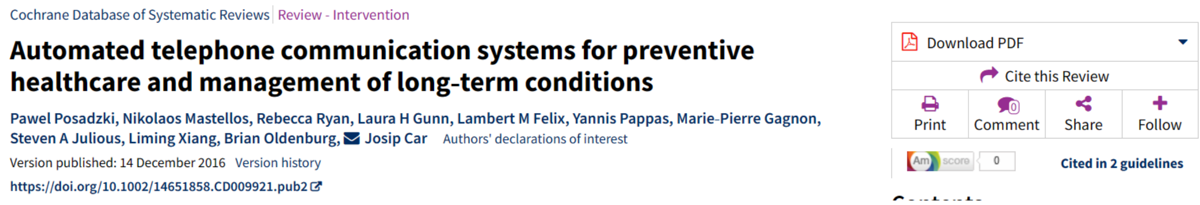

[104] Raman, Shepherd, Dowswell, Middleton and Crowther (2017) Different methods and settings for glucose monitoring for gestational diabetes during pregnancy

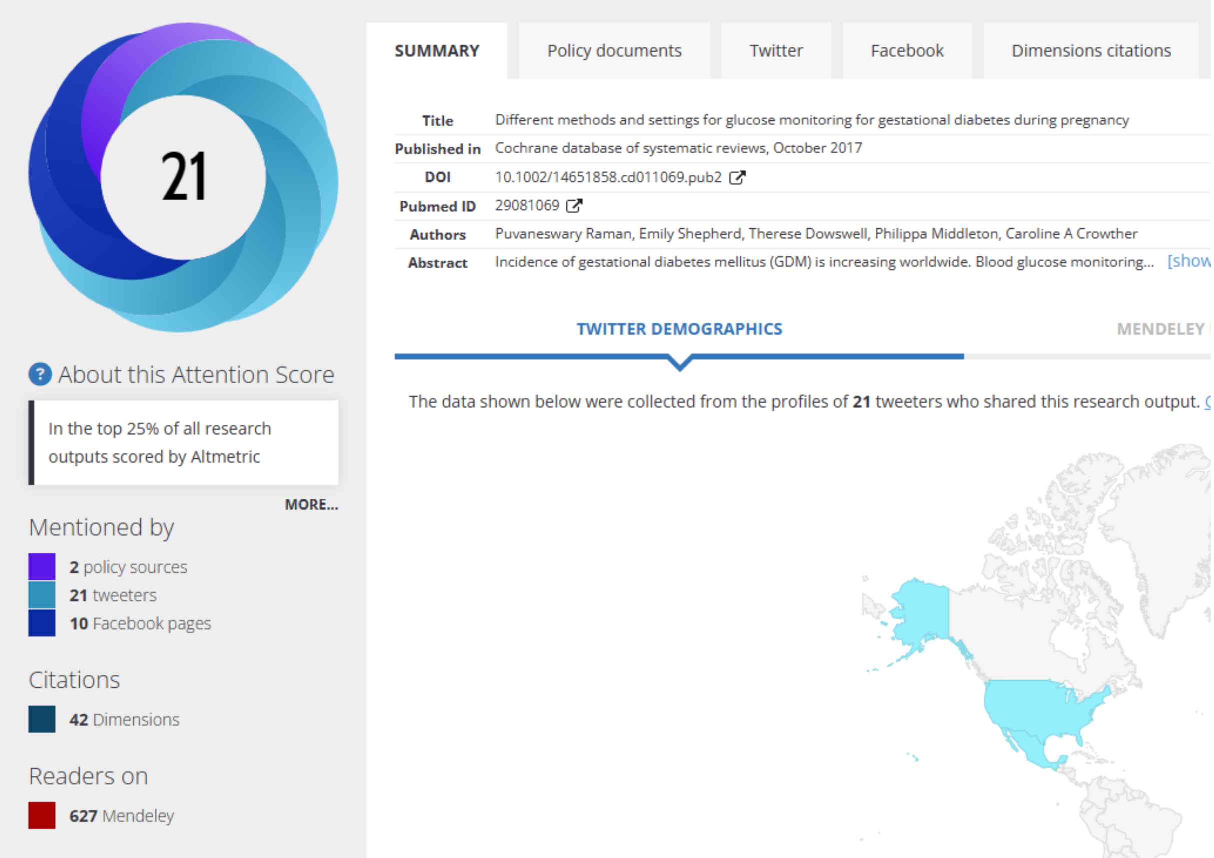

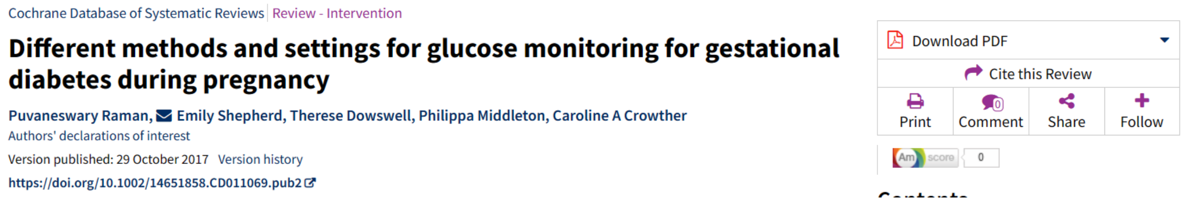

[105] Ream, Hughes, Cox, Skarparis, Richardson, Pedersen, Wiseman, Forbes and Bryant (2020)
Telephone interventions for symptom management in adults with cancer

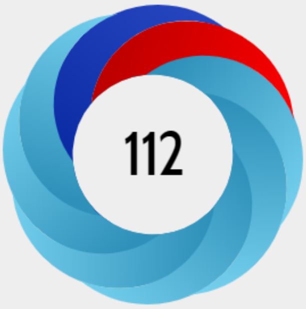

About this Attention Score

In the top 5% of all research outputs scored by Altmetric

MORE...

Mentioned by

6 news outlets
107 tweeters
4 Facebook pages

Citations

51 Dimensions

Readers on

392 Mendeley

SUMMARY | News | Twitter | Facebook | Dimensions citations

**Title**	Telephone interventions for symptom management in adults with cancer
**Published in**	Cochrane database of systematic reviews, June 2020
**DOI**	10.1002/14651858.cd007568.pub2
**Pubmed ID**	32483832
**Authors**	Emma Ream, Amanda Euesden Hughes, Anna Cox, Katy Skarparis, Alison Richardson, Vibe H Pedersen... [show]

TWITTER DEMOGRAPHICS MENDELEY READ

The data shown below were collected from the profiles of **107** tweeters who shared this research output. Click

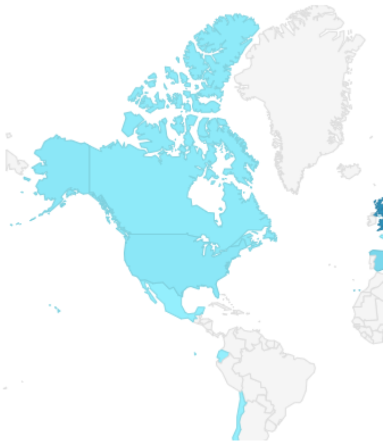

Cochrane Database of Systematic Reviews | Review - Intervention

Telephone interventions for symptom management in adults with cancer

Emma Ream, Amanda Euesden Hughes, Anna Cox, Katy Skarparis, Alison Richardson, Vibe H Pedersen, Theresa Wiseman, Angus Forbes, Andrew Bryant Authors' declarations of interest

Version published: 02 June 2020 Version history

https://doi.org/10.1002/14651858.CD007568.pub2

Download PDF
Cite this Review
Print | Comment | Share | Follow
score 0

Contents

[106] Reeves, Pelone, Harrison, Goldman and Zwarenstein (2017) Interprofessional collaboration to improve professional practice and healthcare outcomes

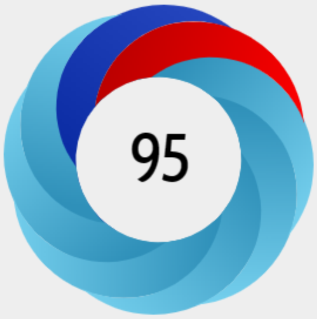

About this Attention Score

In the top 5% of all research outputs scored by Altmetric

MORE...

Mentioned by

2 news outlets
129 tweeters
2 Facebook pages

Citations

596 Dimensions

Readers on

1414 Mendeley
1 CiteULike

SUMMARY | News | Twitter | Facebook | Dimensions citations

**Title**	Interprofessional collaboration to improve professional practice and healthcare outcomes
**Published in**	Cochrane database of systematic reviews, June 2017
**DOI**	10.1002/14651858.cd000072.pub3
**Pubmed ID**	28639262
**Authors**	Scott Reeves, Ferruccio Pelone, Reema Harrison, Joanne Goldman, Merrick Zwarenstein
**Abstract**	Poor interprofessional collaboration (IPC) can adversely affect the delivery of health services... [show]

TWITTER DEMOGRAPHICS   MENDELEY

The data shown below were collected from the profiles of **129** tweeters who shared this research output.

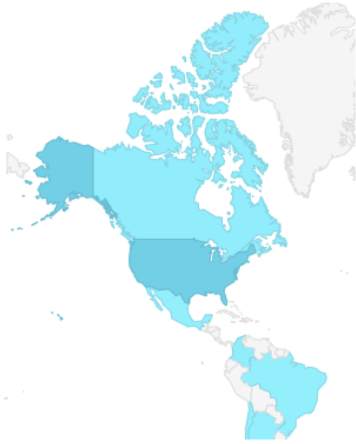

Cochrane Database of Systematic Reviews | Review - Intervention

Interprofessional collaboration to improve professional practice and healthcare outcomes

Scott Reeves[a], Ferruccio Pelone, Reema Harrison, Joanne Goldman, Merrick Zwarenstein   Authors' declarations of interest

Version published: 22 June 2017   Version history

https://doi.org/10.1002/14651858.CD000072.pub3

Download PDF
Cite this Review
Print | Comment | Share | Follow

0   Cited in 3 guidelines

[107] Richards, Thorogood, Hillsdon and Foster (2013) Face-to-face versus remote and web 2.0 interventions for promoting physical activity

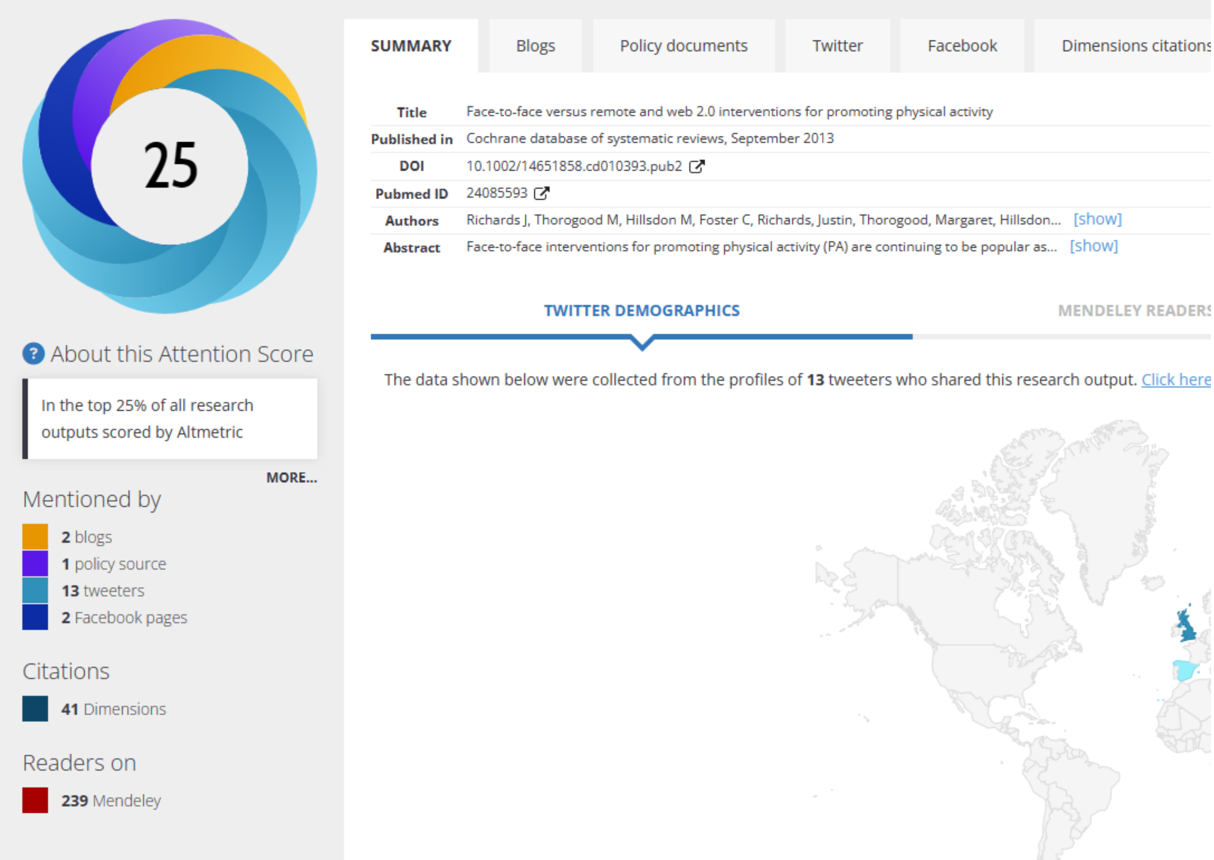

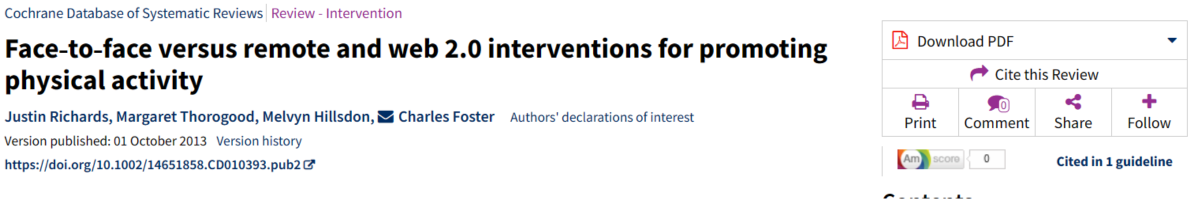

[108] Roberts, Lloyd, Välimäki, Ho, Freemantle and Békefi (2021) Video games for people with schizophrenia

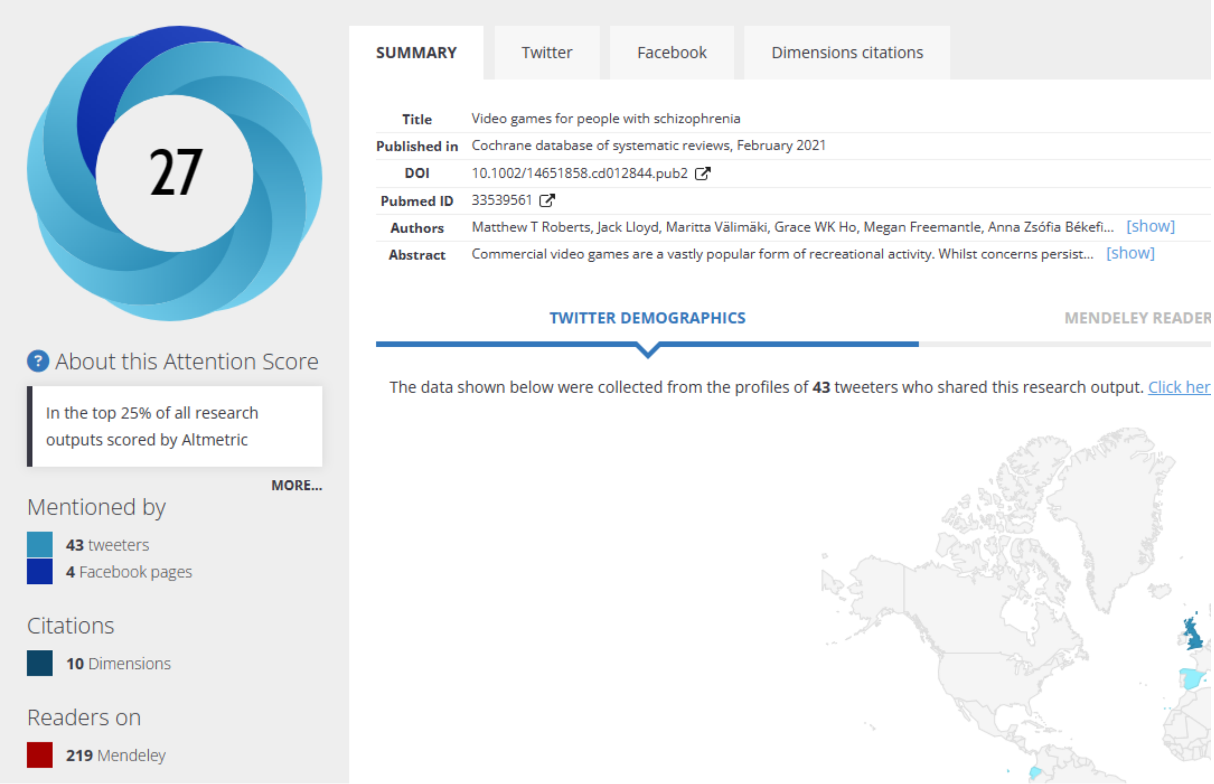

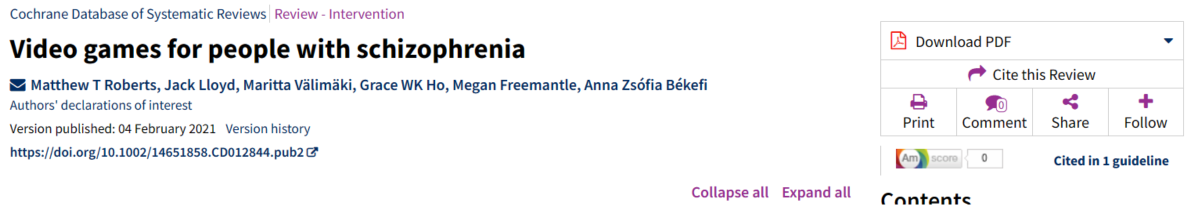

[109] Sawmynaden, Atherton, Majeed and Car (2012) Email for the provision of information on disease prevention and health promotion

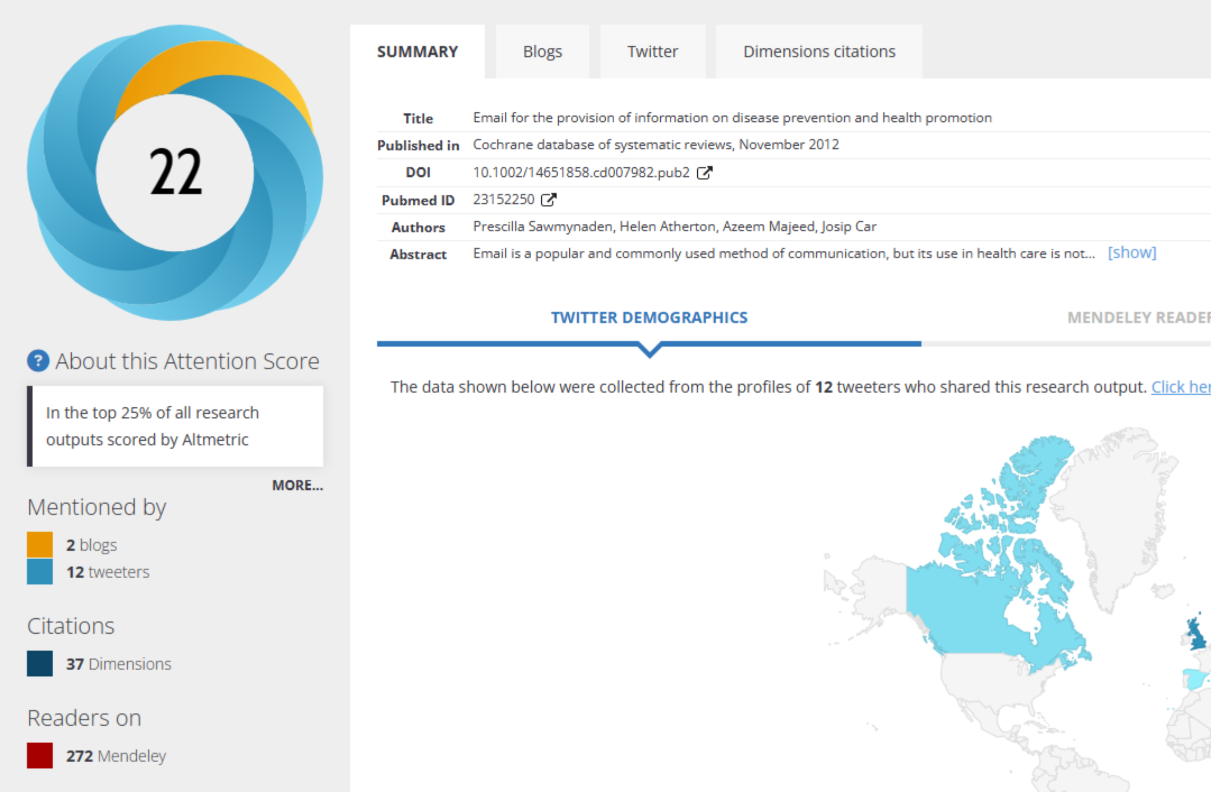

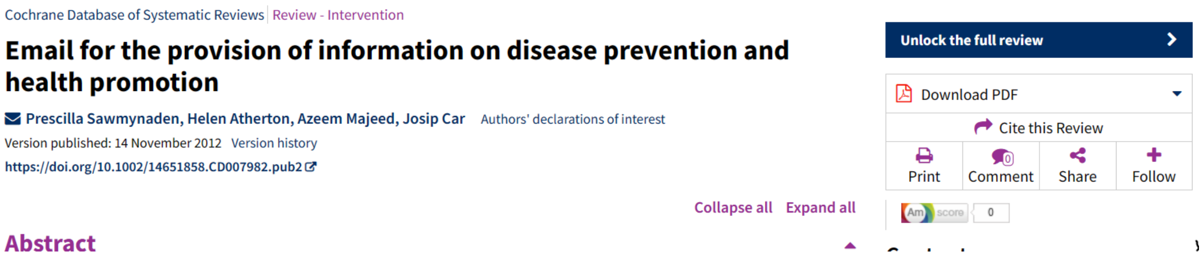

[110] Shojania, Jennings, Ramsay, Grimshaw, Kwan and Lo (2009) The effects of on-screen, point of care computer reminders on processes and outcomes of care

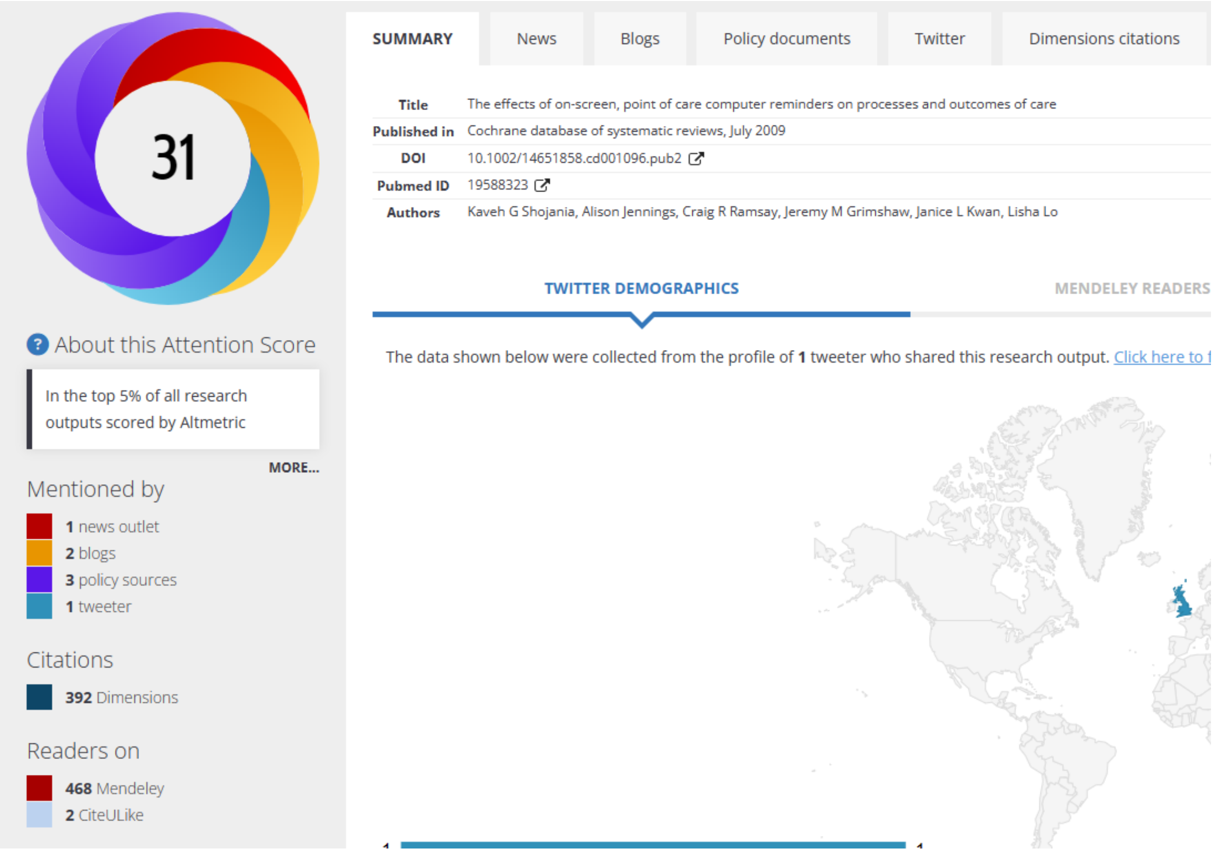

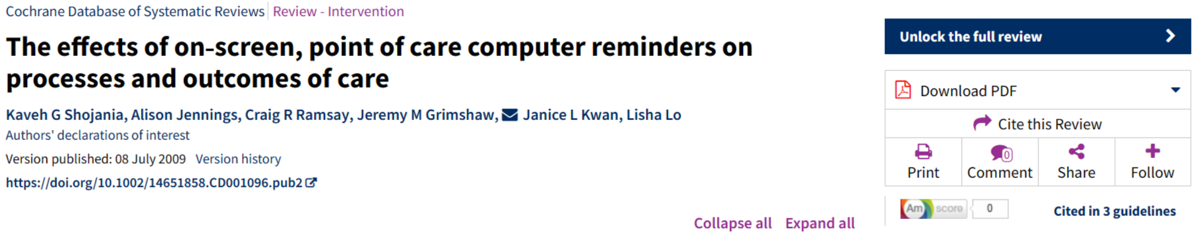

[111] Simon, Robertson, Lewis, Roberts, Bethell, Dawson and Bisson (2021) Internet-based cognitive and behavioural therapies for post-traumatic stress disorder (PTSD) in adults

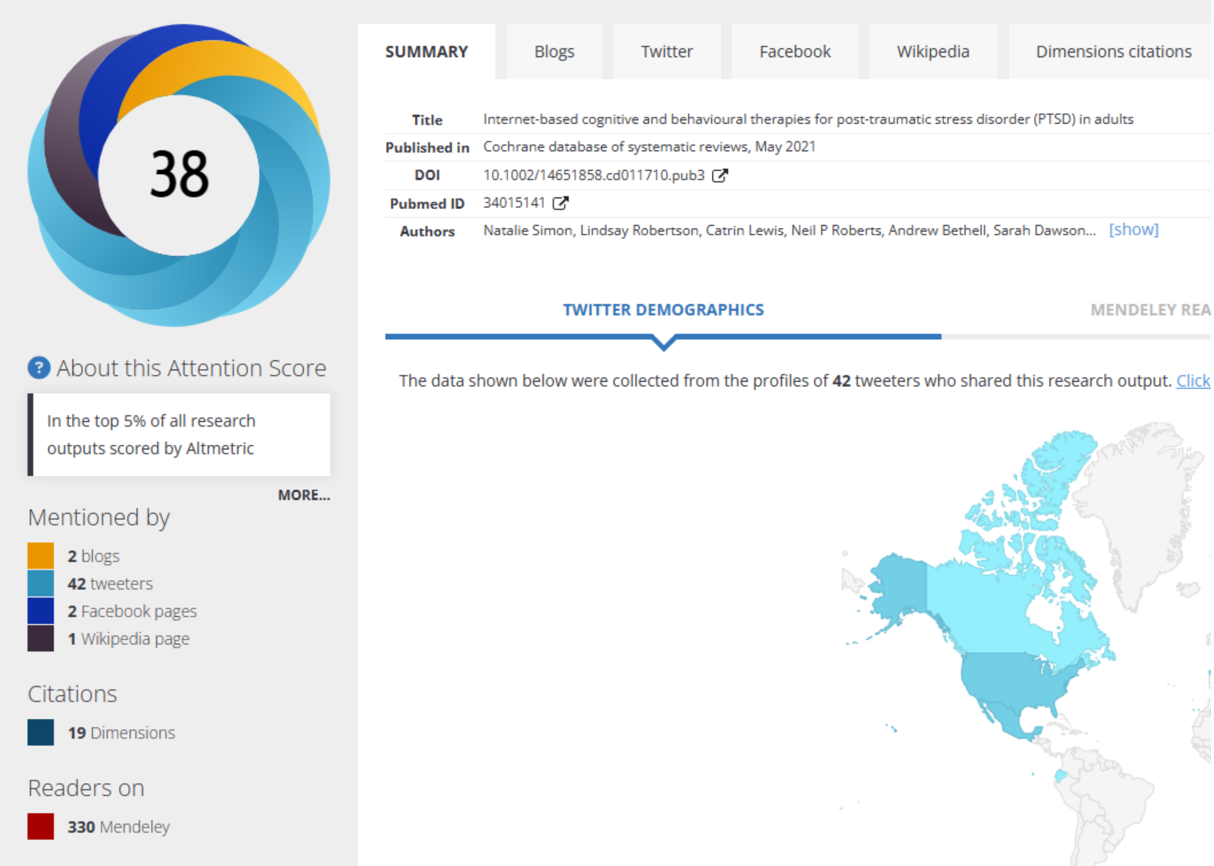

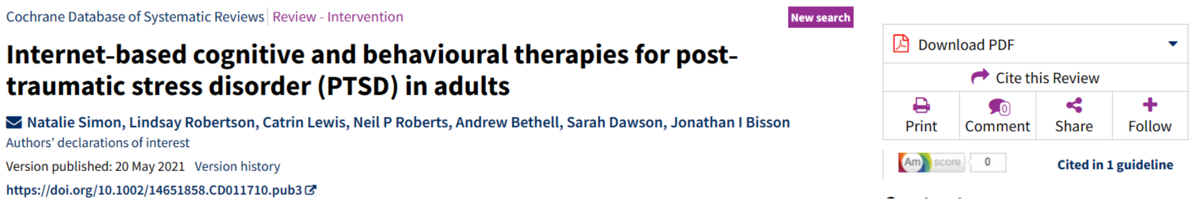

[112] Sjøstrand, Kefalianos, Hofslundsengen, Guttormsen, Kirmess, Lervåg, Hulme and Bottegaard Næss (2021) Non-pharmacological interventions for stuttering in children six years and younger

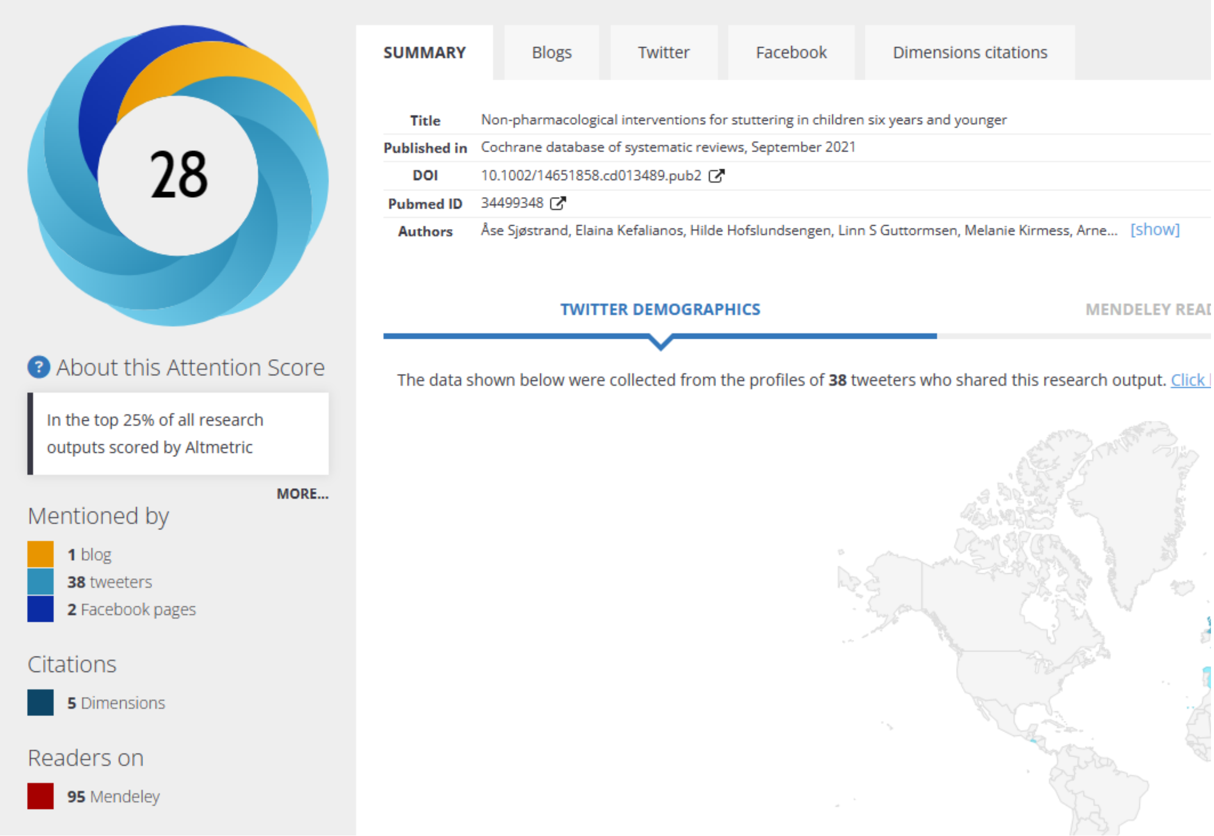

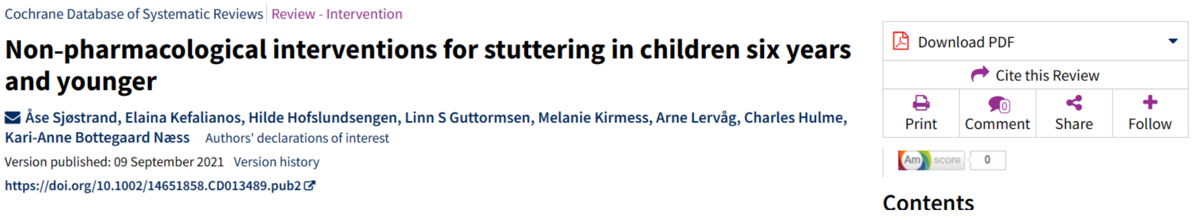

[113] Smith, Gold, Ngo, Sumpter and Free (2015) Mobile phone-based interventions for improving contraception use

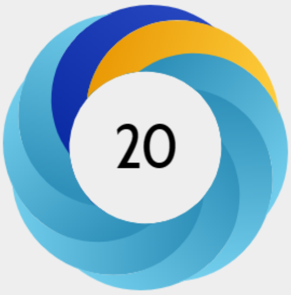

About this Attention Score

In the top 25% of all research outputs scored by Altmetric

MORE...

Mentioned by

2 blogs
12 tweeters
1 Facebook page

Citations

107 Dimensions

Readers on

478 Mendeley

SUMMARY | Blogs | Twitter | Facebook | Dimensions citations

**Title**	Mobile phone-based interventions for improving contraception use
**Published in**	Cochrane database of systematic reviews, June 2015
**DOI**	10.1002/14651858.cd011159.pub2
**Pubmed ID**	26115146
**Authors**	Chris Smith, Judy Gold, Thoai D Ngo, Colin Sumpter, Caroline Free
**Abstract**	Contraception provides significant benefits for women's and children's health, yet an estimated... [show]

TWITTER DEMOGRAPHICS | MENDELEY READERS

The data shown below were collected from the profiles of **12** tweeters who shared this research output. Click here to fi

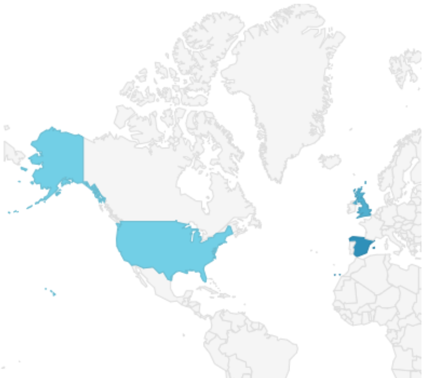

Cochrane Database of Systematic Reviews | Review - Intervention

Mobile phone-based interventions for improving contraception use

Chris Smith, Judy Gold, Thoai D Ngo, Colin Sumpter, Caroline Free   Authors' declarations of interest

Version published: 26 June 2015   Version history
https://doi.org/10.1002/14651858.CD011159.pub2

Download PDF
Cite this Review
Print | Comment | Share | Follow

Collapse all   Expand all

0   Cited in 7 guidelines

[114] Smith, Calthorpe, Herbert and Smyth (2023) Digital technology for monitoring adherence to inhaled therapies in people with cystic fibrosis

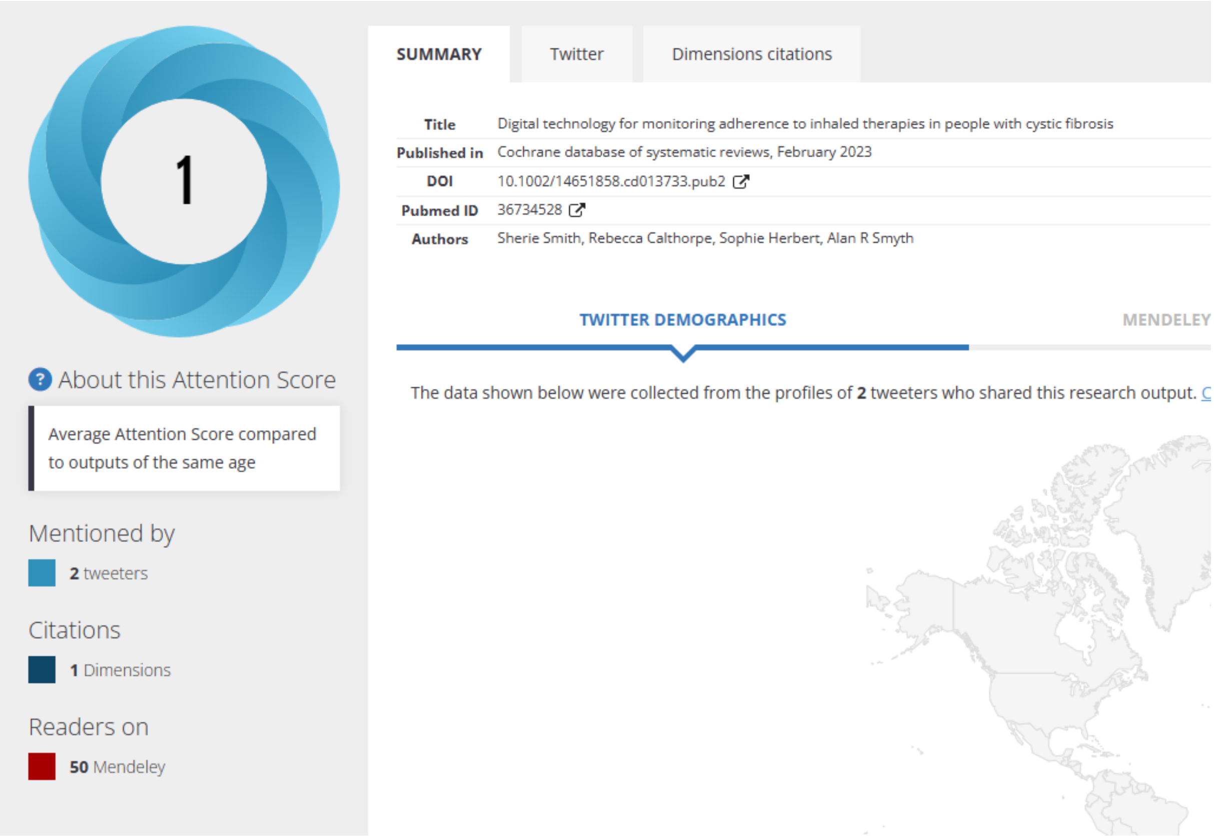

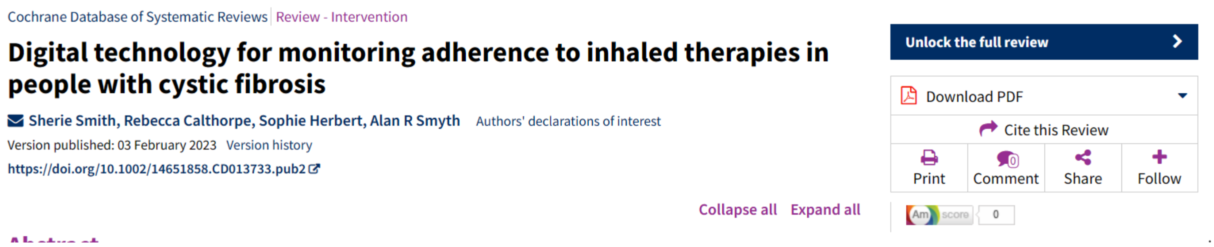

[115] Stevenson, Campbell, Webster, Chow, Tong, Craig, Campbell and Lee (2019) eHealth interventions for people with chronic kidney disease

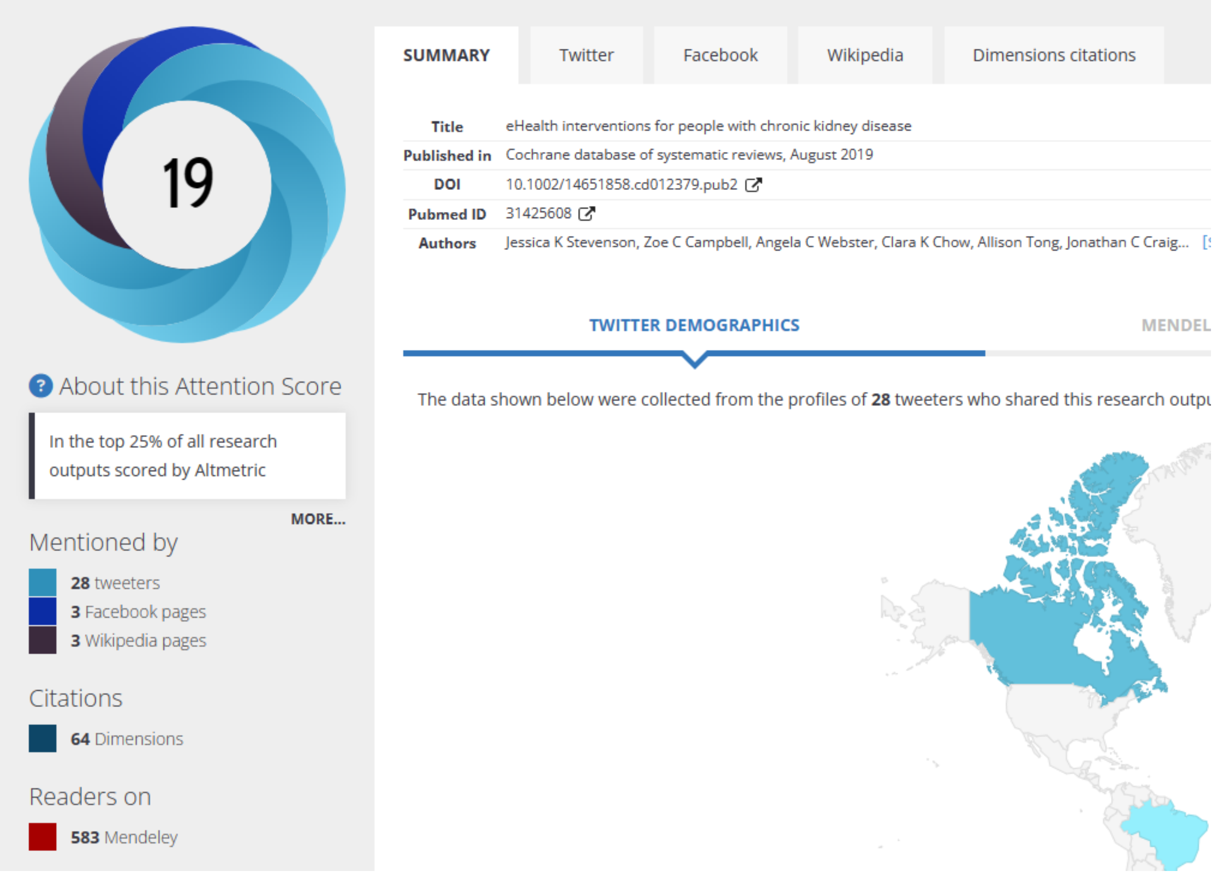

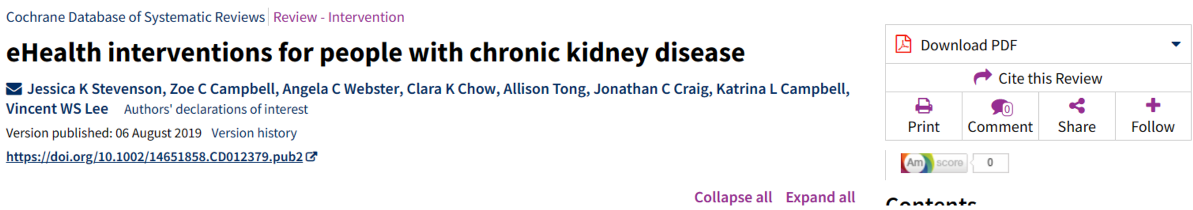

[116] Tailor, Ludden, Bossi, Bunce, Greenwood and Dahlmann-Noor (2022) Binocular versus standard occlusion or blurring treatment for unilateral amblyopia in children aged three to eight years

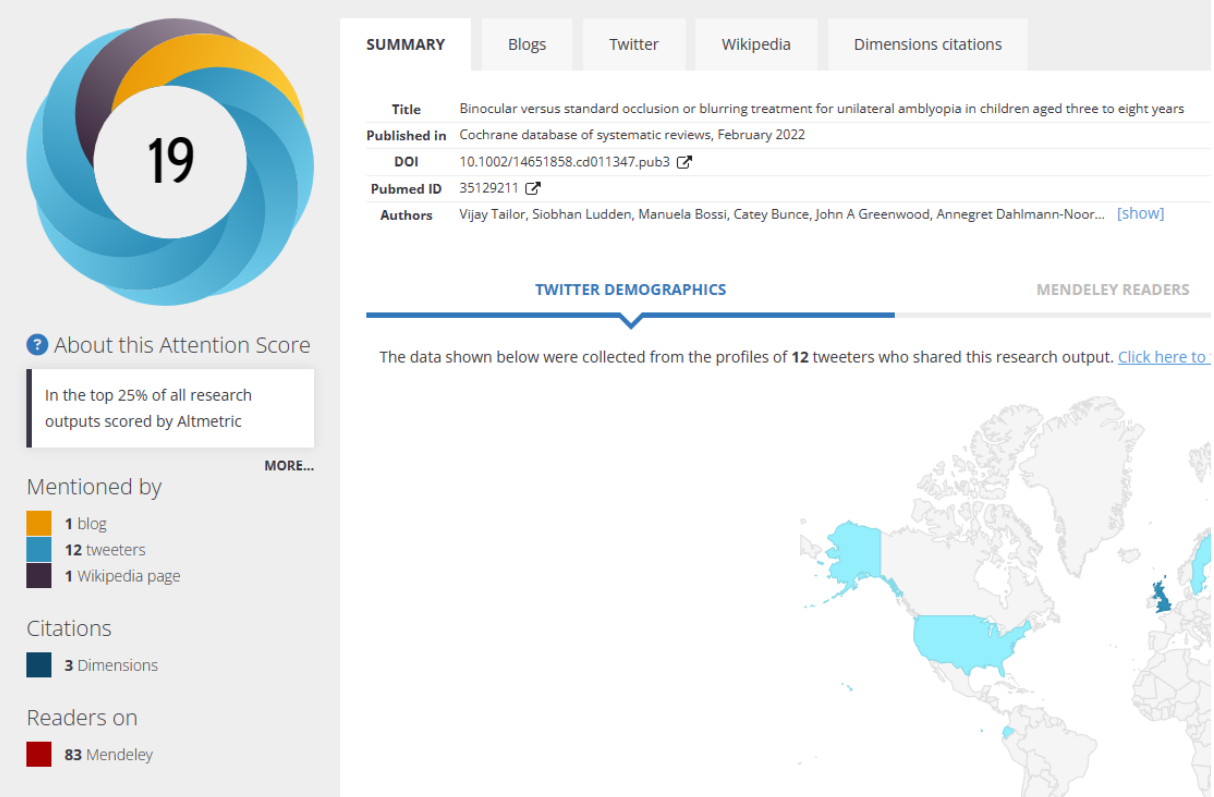

Cochrane Database of Systematic Reviews | Review - Intervention

Binocular versus standard occlusion or blurring treatment for unilateral amblyopia in children aged three to eight years

Vijay Tailor, Siobhan Ludden, Manuela Bossi, Catey Bunce, John A Greenwood, Annegret Dahlmann-Noor
Authors' declarations of interest

Version published: 07 February 2022 Version history
https://doi.org/10.1002/14651858.CD011347.pub3

Download PDF
Cite this Review
Print Comment Share Follow
0

[117] Tan, Dear and Newell (2005) Clinical decision support systems for neonatal care

Clinical decision support systems for neonatal care

Overview of attention for article published in Cochrane database of systematic reviews, April 2005

SUMMARY | Dimensions citations

**Title**	Clinical decision support systems for neonatal care
**Published in**	Cochrane database of systematic reviews, April 2005
**DOI**	10.1002/14651858.cd004211.pub2
**Pubmed ID**	15846701
**Authors**	Kenneth Tan, Peter RF Dear, Simon J Newell

?

Citations

33 Dimensions

Readers on

211 Mendeley

The data shown below were compiled from readership statistics for **211** Men

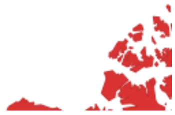

Cochrane Database of Systematic Reviews | Review - Intervention

Clinical decision support systems for neonatal care

Kenneth Tan, Peter RF Dear, Simon J Newell   Authors' declarations of interest

Version published: 20 April 2005   Version history
https://doi.org/10.1002/14651858.CD004211.pub2

Unlock the full review

Download PDF

Cite this Review

Print   Comment   Share   Follow

score 0

Collapse all   Expand all

Abstract

Available in English | Español

[118] Tan and Lai (2012) Telemedicine for the support of parents of high-risk newborn infants

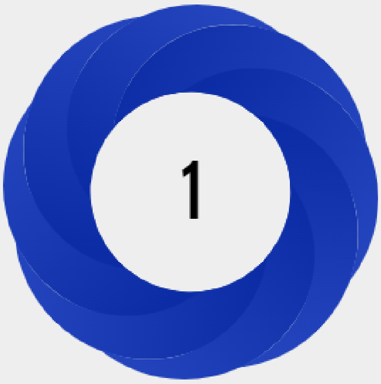

1

Mentioned by

1 Facebook page

Citations

28 Dimensions

Readers on

300 Mendeley

SUMMARY | Facebook | Dimensions citations

**Title**	Telemedicine for the support of parents of high-risk newborn infants
**Published in**	Cochrane database of systematic reviews, June 2012
**DOI**	10.1002/14651858.cd006818.pub2
**Pubmed ID**	22696360
**Authors**	Kenneth Tan, Nai Ming Lai

MENDELEY READERS

The data shown below were compiled from readership statistics for **300**

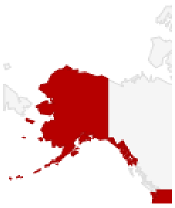

Cochrane Database of Systematic Reviews | Review - Intervention

Telemedicine for the support of parents of high-risk newborn infants

Kenneth Tan, Nai Ming Lai   Authors' declarations of interest

Version published: 13 June 2012   Version history

https://doi.org/10.1002/14651858.CD006818.pub2

Collapse all   Expand all

Abstract

Available in **English** | Español | فارسی | Français | 日本語 | ภาษาไทย

Unlock the full review

Download PDF

Cite this Review

Print | Comment | Share | Follow

Contents

[119] Taylor, Dalili, Semwal, Civljak, Sheikh and Car (2017) Internet-based interventions for smoking cessation

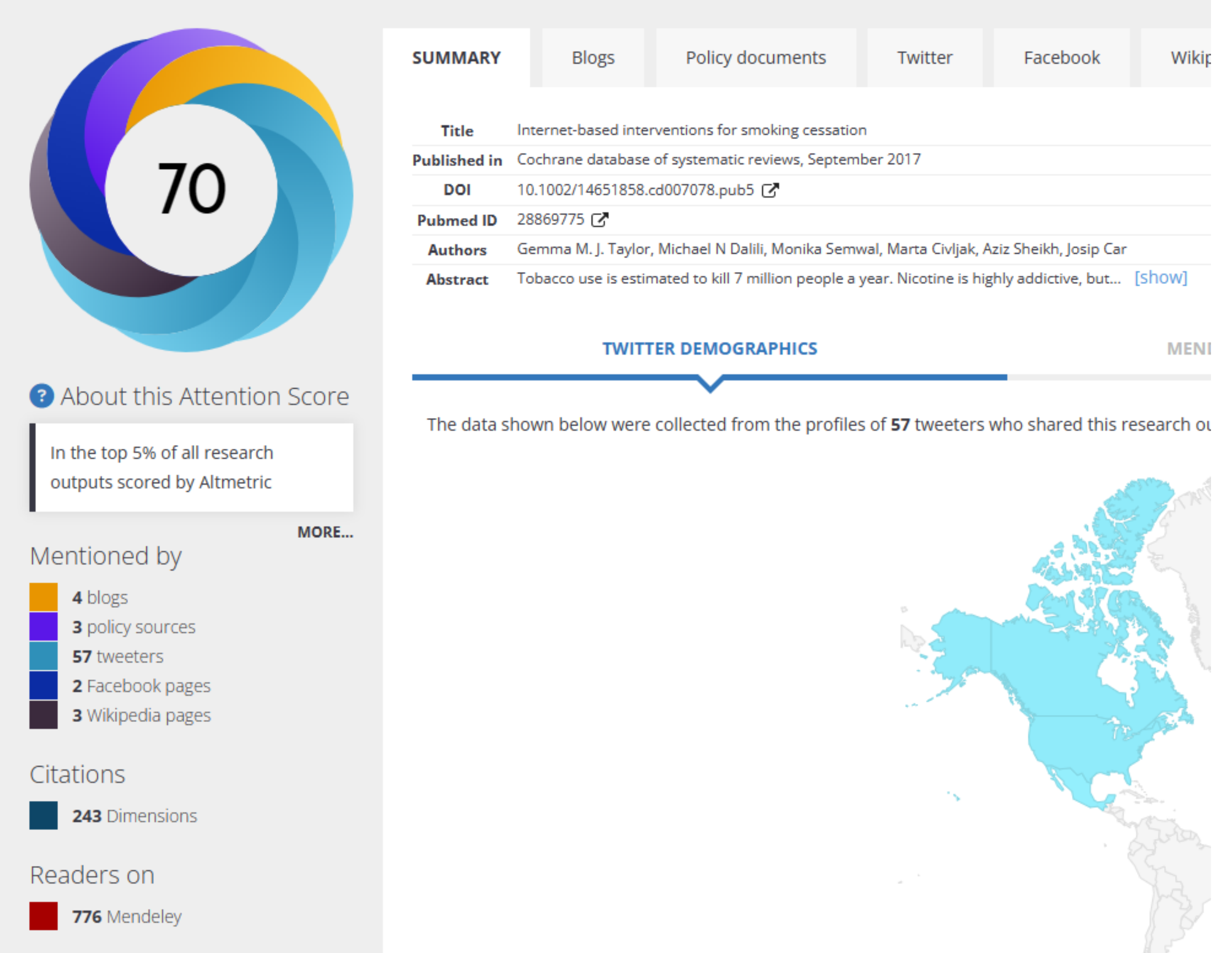

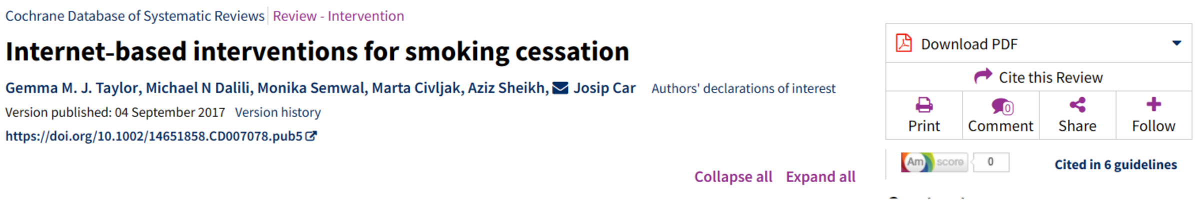

[120] Thabrew, Stasiak, Hetrick, Wong, Huss and Merry (2018) E-Health interventions for anxiety and depression in children and adolescents with long-term physical conditions

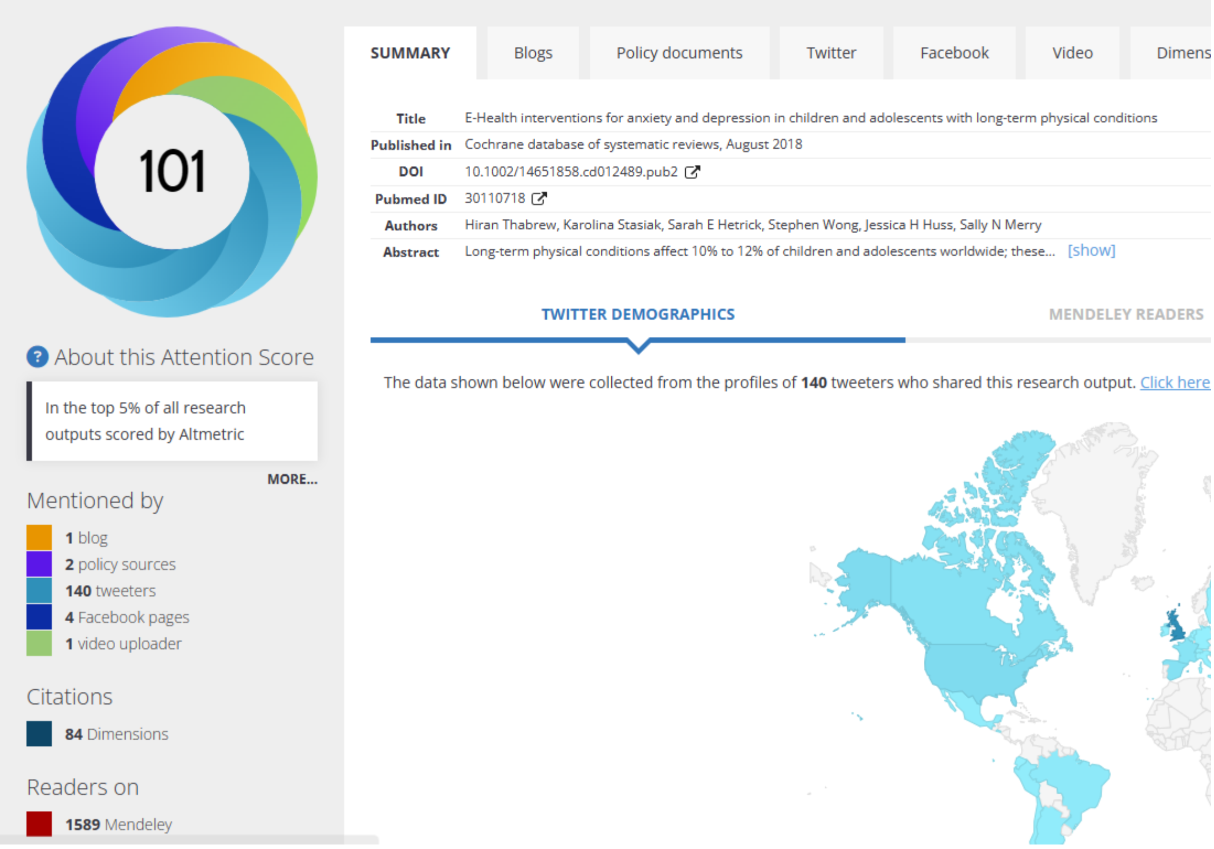

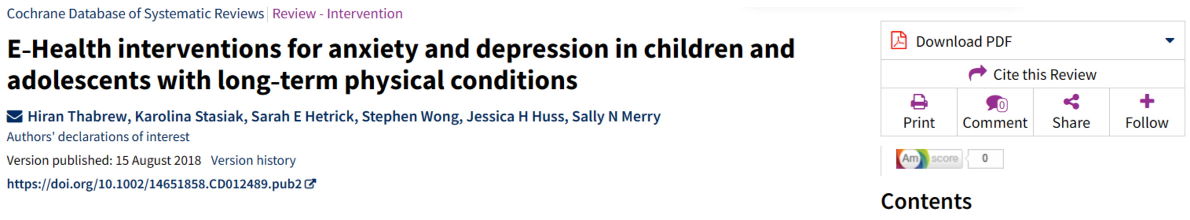

[121] Treanor, McMenamin, O'Neill, Cardwell, Clarke, Cantwell and Donnelly (2016) Non-pharmacological interventions for cognitive impairment due to systemic cancer treatment

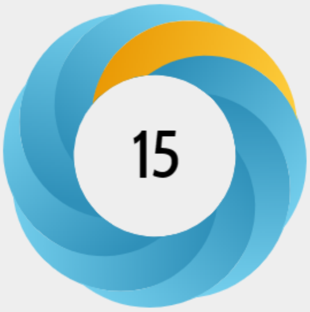

About this Attention Score

In the top 25% of all research outputs scored by Altmetric

MORE...

Mentioned by

1 blog
14 tweeters

Citations

75 Dimensions

Readers on

612 Mendeley

SUMMARY | Blogs | Twitter | Dimensions citations

**Title**	Non-pharmacological interventions for cognitive impairment due to systemic cancer treatment
**Published in**	Cochrane database of systematic reviews, August 2016
**DOI**	10.1002/14651858.cd011325.pub2
**Pubmed ID**	27529826
**Authors**	Charlene J Treanor, Una C McMenamin, Roisin F O'Neill, Chris R Cardwell, Mike J Clarke, Marie M... [show]
**Abstract**	It is estimated that up to 75% of cancer survivors may experience cognitive impairment as a result... [show]

TWITTER DEMOGRAPHICS | MENDELEY REA

The data shown below were collected from the profiles of **14** tweeters who shared this research output. Click

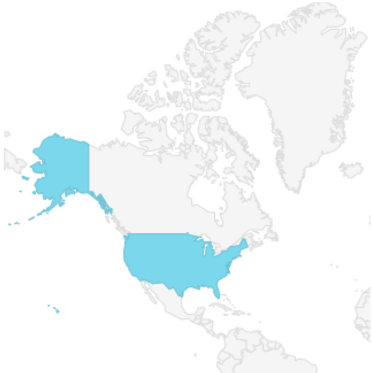

Cochrane Database of Systematic Reviews | Review - Intervention

Non-pharmacological interventions for cognitive impairment due to systemic cancer treatment

Charlene J Treanor, Una C McMenamin, Roisin F O'Neill, Chris R Cardwell, Mike J Clarke, Marie M Cantwell, Michael Donnelly Authors' declarations of interest

Version published: 16 August 2016 Version history

https://doi.org/10.1002/14651858.CD011325.pub2

Download PDF

Cite this Review

Print | Comment | Share | Follow

Am score 0

Cited in 8 guidelines

Contents

[122] Treweek, Pitkethly, Cook, Fraser, Mitchell, Sullivan, Jackson, Taskila and Gardner (2018) Strategies to improve recruitment to randomised trials

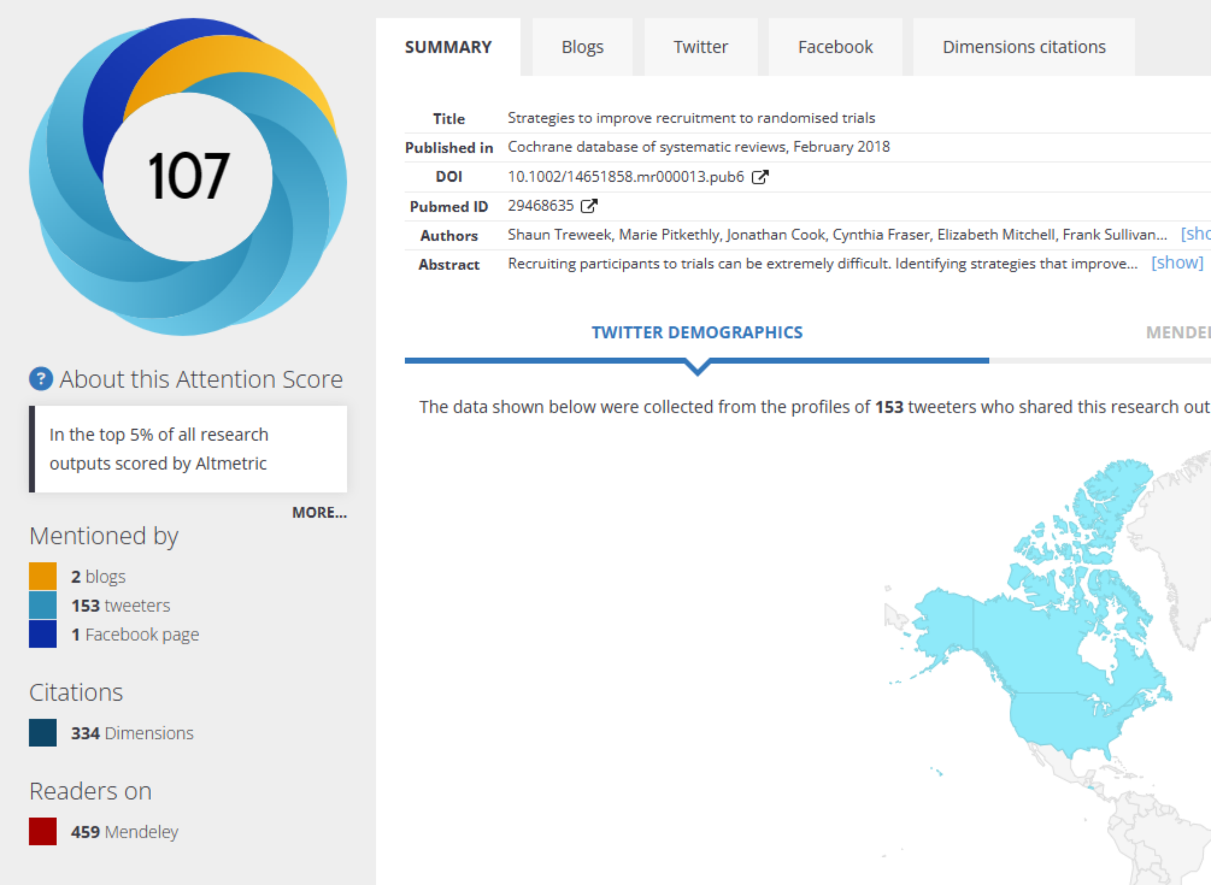

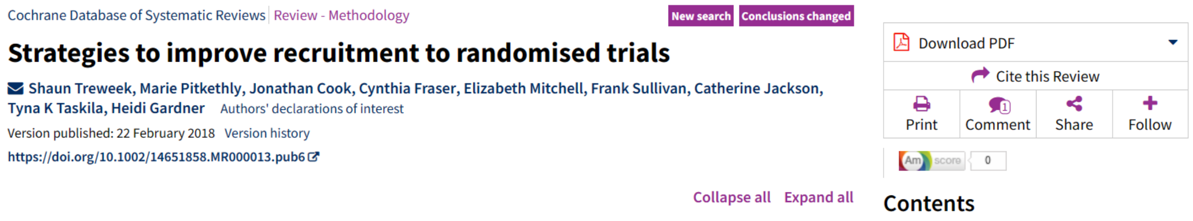

[123] Tudor Car, Gentry, van-Velthoven and Car (2013) Telephone communication of HIV testing results for improving knowledge of HIV infection status

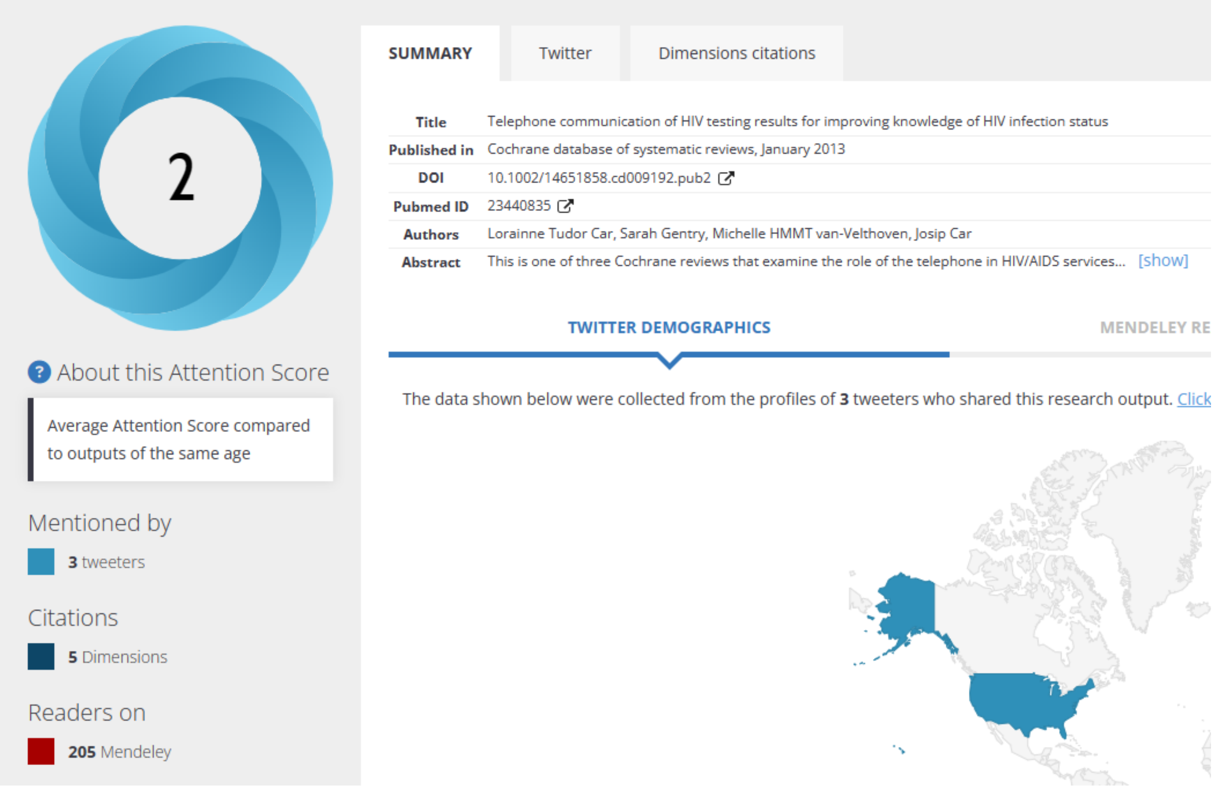

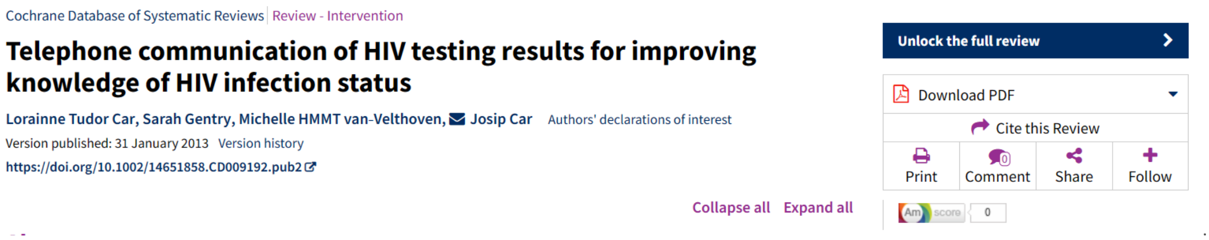

[124] Välimäki, Hätönen, Lahti, Kurki, Hottinen, Metsäranta, Riihimäki and Adams (2014) Virtual reality for treatment compliance for people with serious mental illness

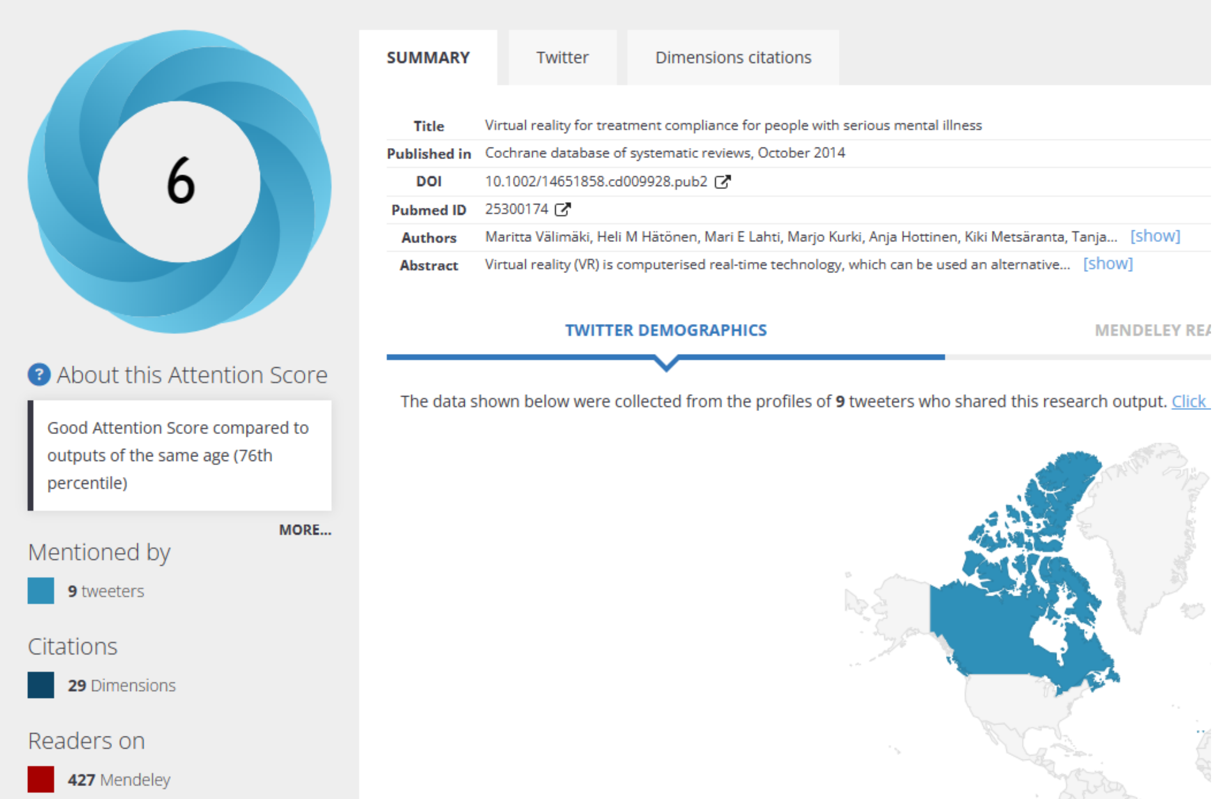

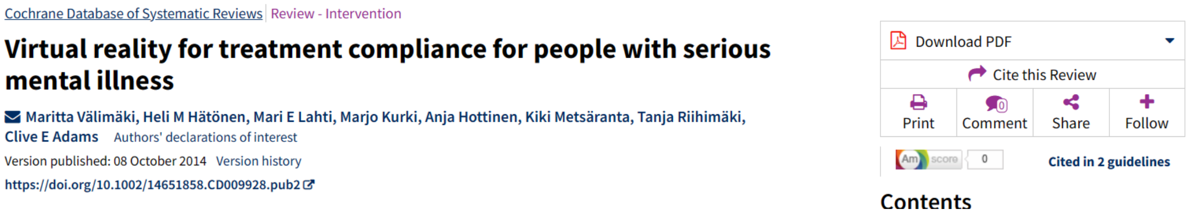

[125] van-Velthoven, Tudor Car, Gentry and Car (2013) Telephone delivered interventions for preventing HIV infection in HIV-negative persons

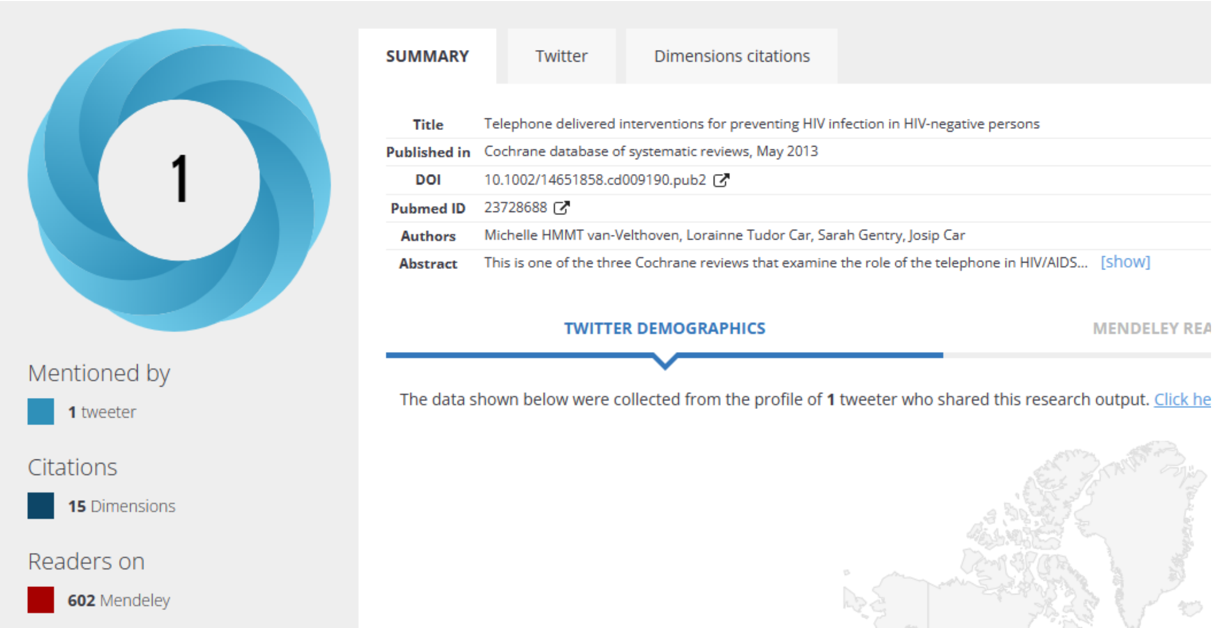

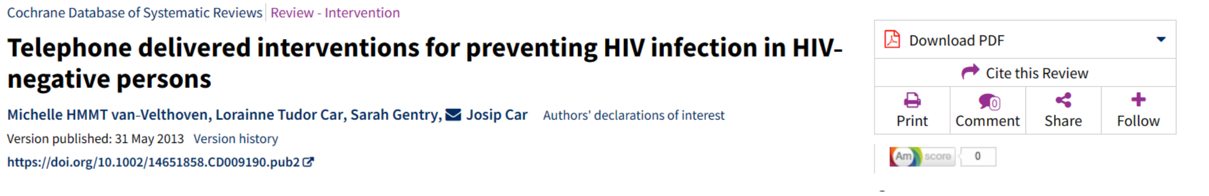

[126] Vaona, Banzi, Kwag, Rigon, Cereda, Pecoraro, Tramacere and Moja (2018) E-learning for health professionals

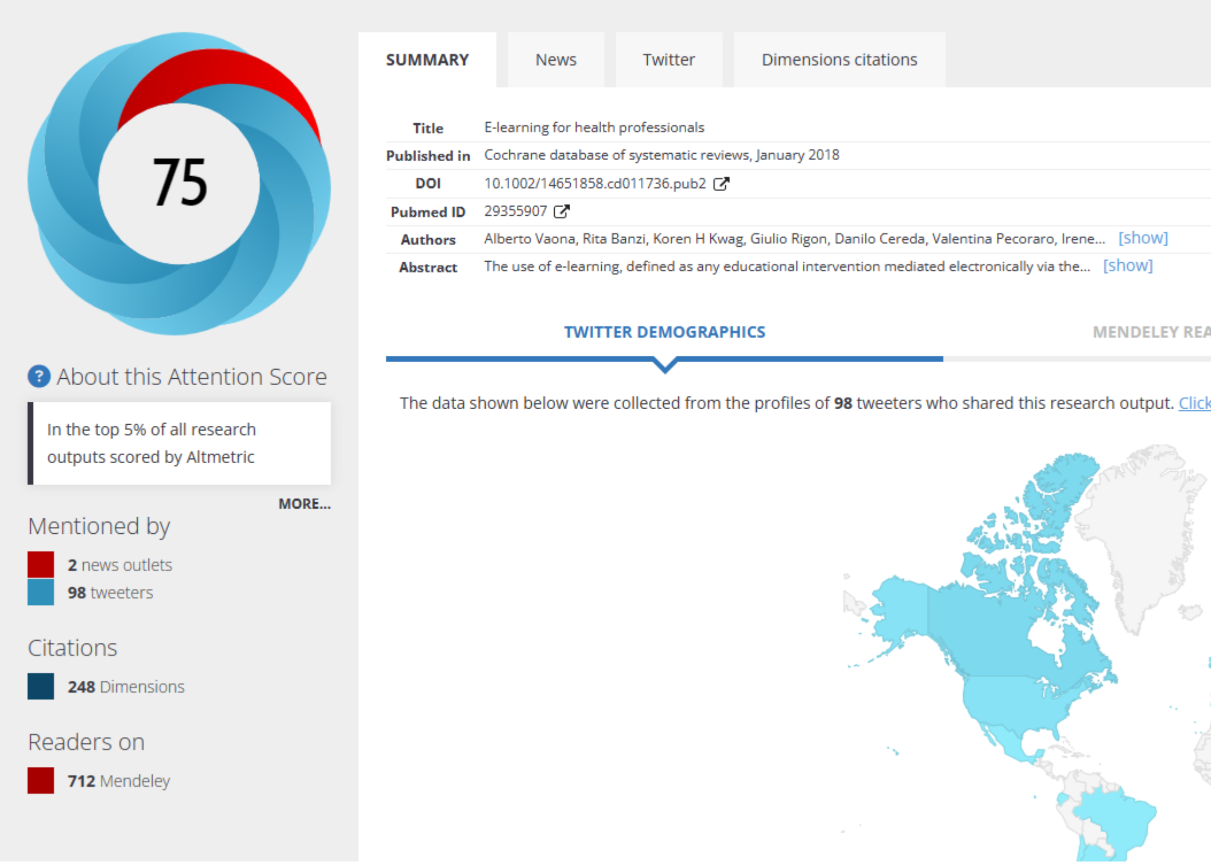

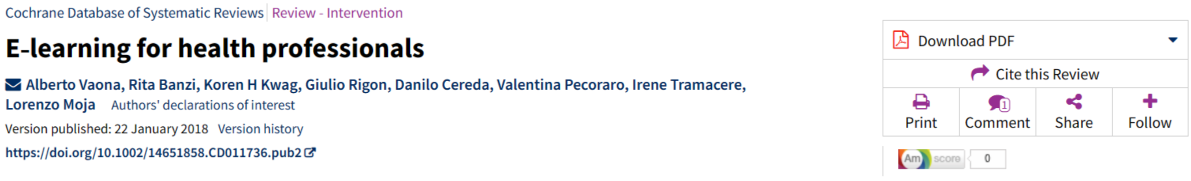

[127] Vasudevan, Glenton, Henschke, Maayan, Eyers, Fønhus, Tamrat, Mehl and Lewin (2021) Birth and death notification via mobile devices: a mixed methods systematic review

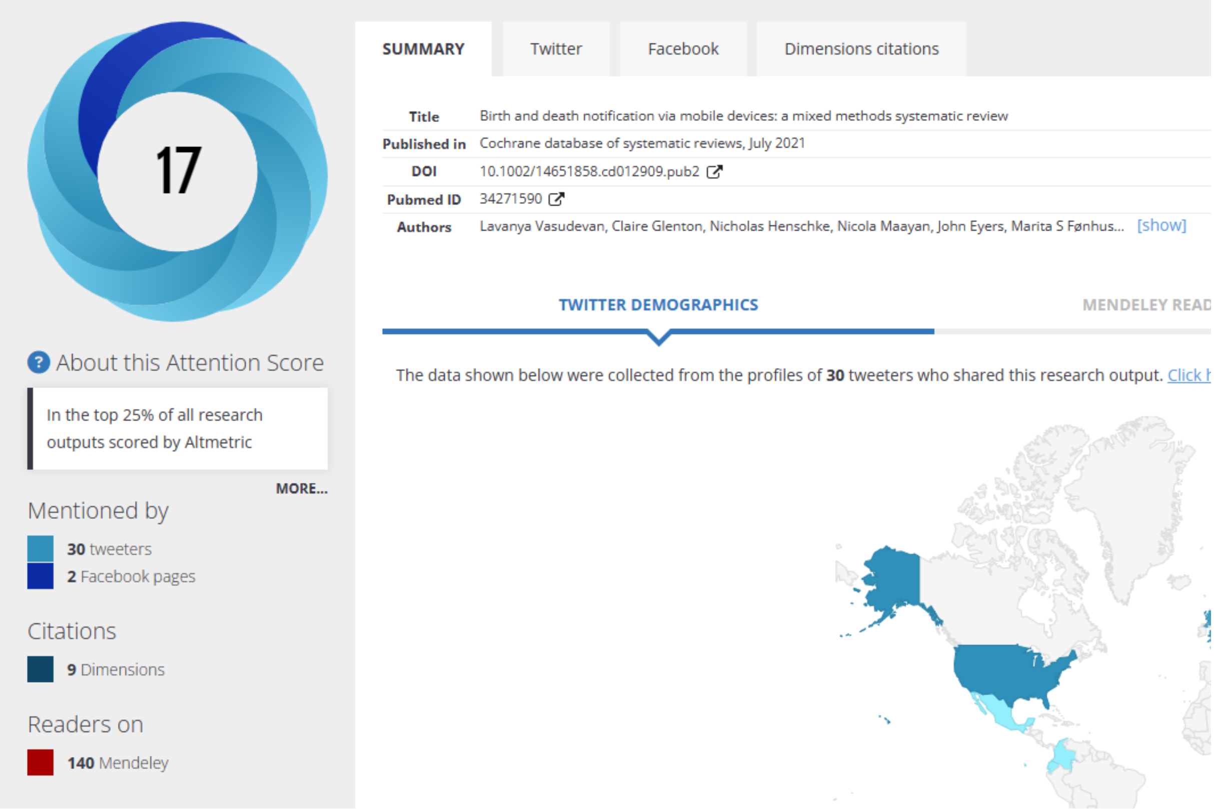

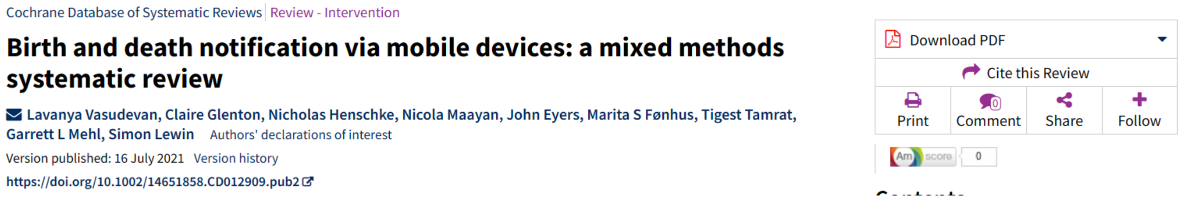

[128] Verbeek, Rajamaki, Ijaz, Sauni, Toomey, Blackwood, Tikka, Ruotsalainen and Kilinc Balci (2020) Personal protective equipment for preventing highly infectious diseases due to exposure to contaminated body fluids in healthcare staff

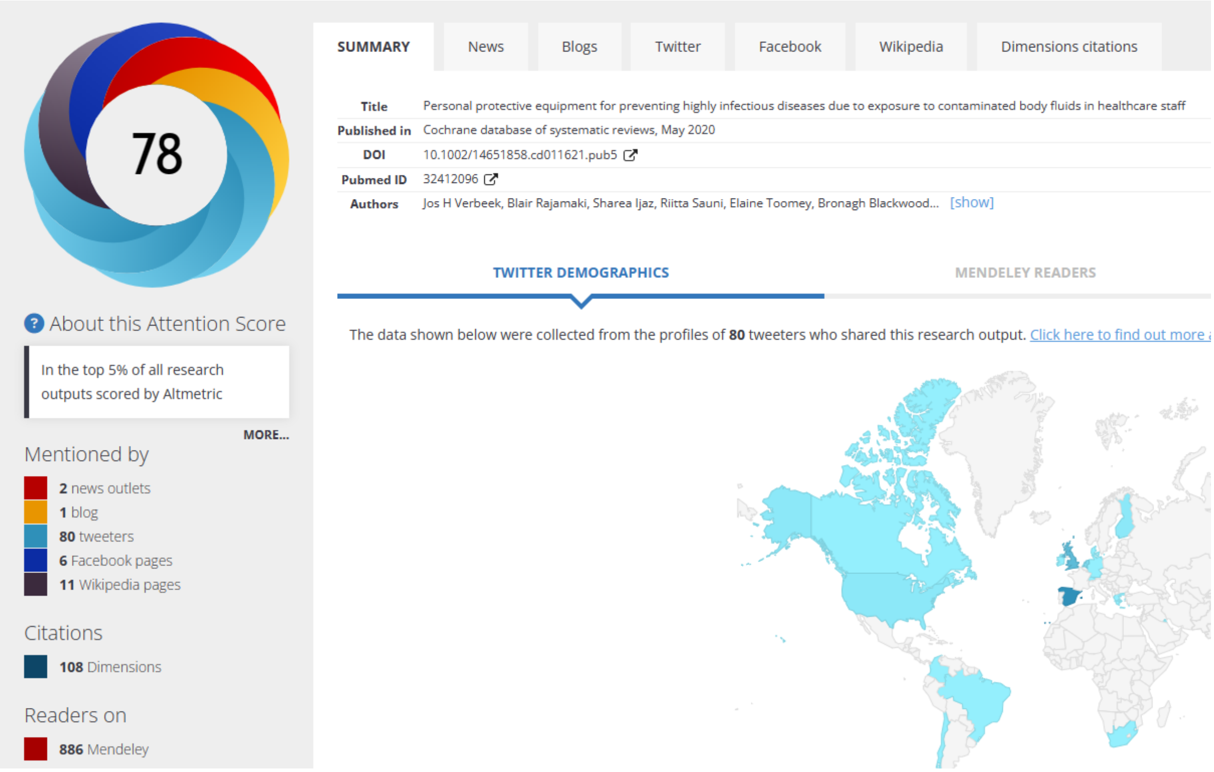

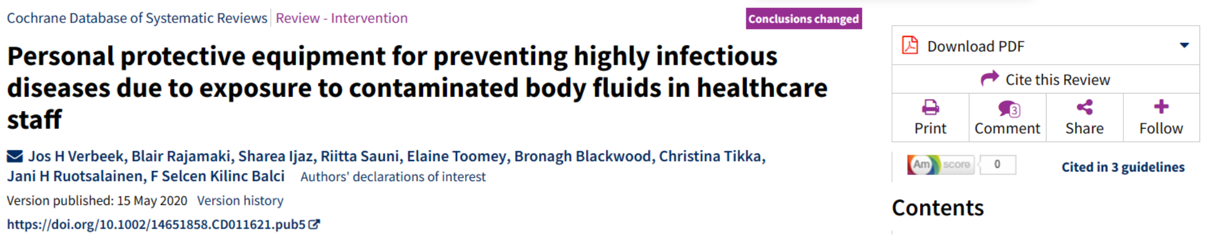

[129] Vijayaraghavan, Elser, Frazer, Lindson and Apollonio (2020) Interventions to reduce tobacco use in people experiencing homelessness

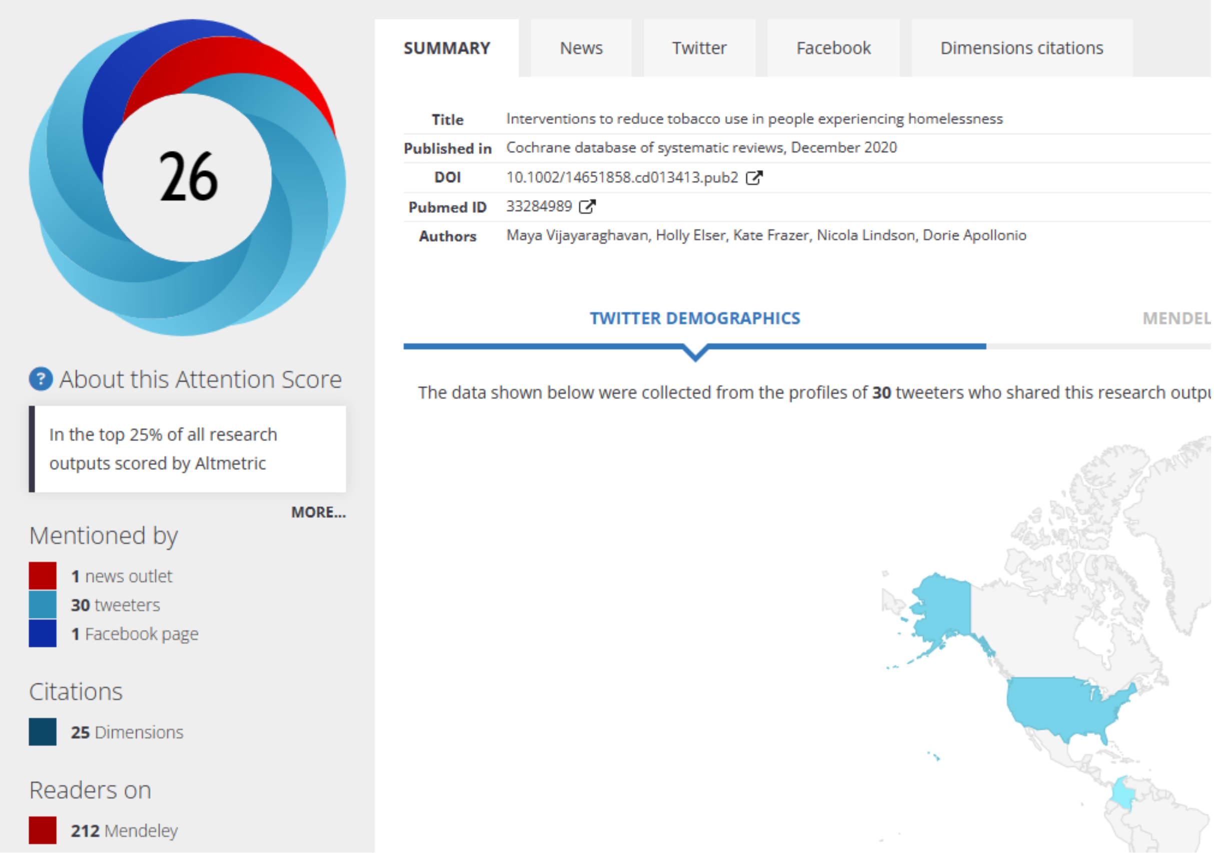

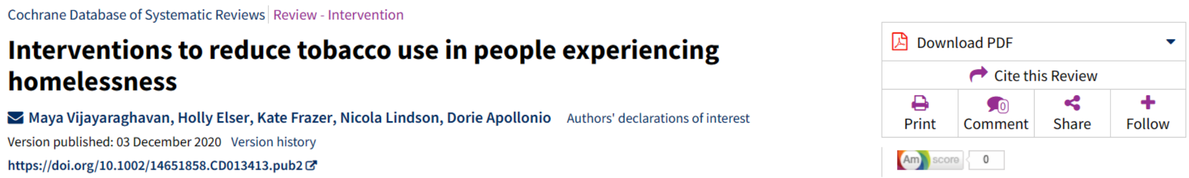

[130] Vodopivec-Jamsek, de Jongh, Gurol-Urganci, Atun and Car (2012) Mobile phone messaging for preventive health care

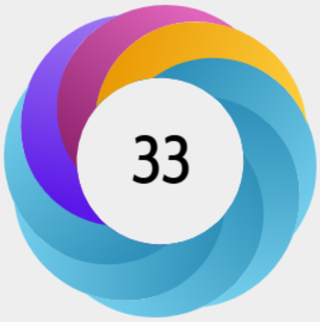

33

About this Attention Score

In the top 5% of all research outputs scored by Altmetric

MORE...

Mentioned by

1 blog
4 policy sources
22 tweeters
1 Google+ user

Citations

263 Dimensions

Readers on

1412 Mendeley
2 CiteULike

SUMMARY | Blogs | Policy documents | Twitter | Google+ | Dimensions

Title	Mobile phone messaging for preventive health care
Published in	Cochrane database of systematic reviews, December 2012
DOI	10.1002/14651858.cd007457.pub2
Pubmed ID	23235643
Authors	Vlasta Vodopivec-Jamsek, Thyra de Jongh, Ipek Gurol-Urganci, Rifat Atun, Josip Car
Abstract	Preventive health care promotes health and prevents disease or injuries by addressing factors that... [sho

TWITTER DEMOGRAPHICS   MENDELEY

The data shown below were collected from the profiles of **22** tweeters who shared this research output.

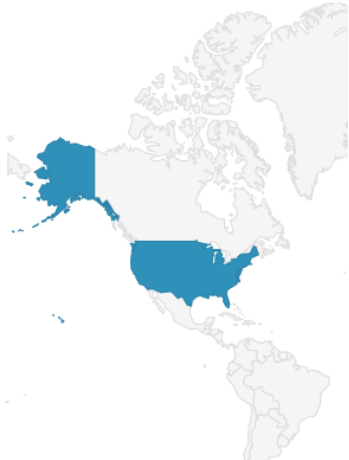

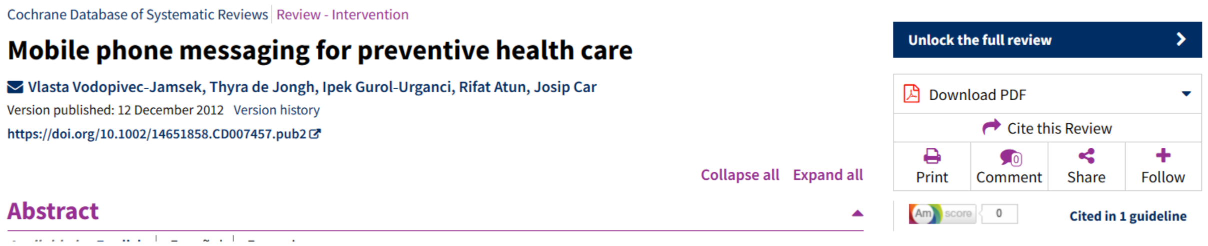

Cochrane Database of Systematic Reviews | Review - Intervention

Mobile phone messaging for preventive health care

Vlasta Vodopivec-Jamsek, Thyra de Jongh, Ipek Gurol-Urganci, Rifat Atun, Josip Car

Version published: 12 December 2012 Version history

https://doi.org/10.1002/14651858.CD007457.pub2

Collapse all Expand all

Abstract

Unlock the full review

Download PDF

Cite this Review

Print | Comment | Share | Follow

Cited in 1 guideline

[131] Wei, Pappas, Car, Sheikh and Majeed (2011) Computer-assisted versus oral-and-written dietary history taking for diabetes mellitus

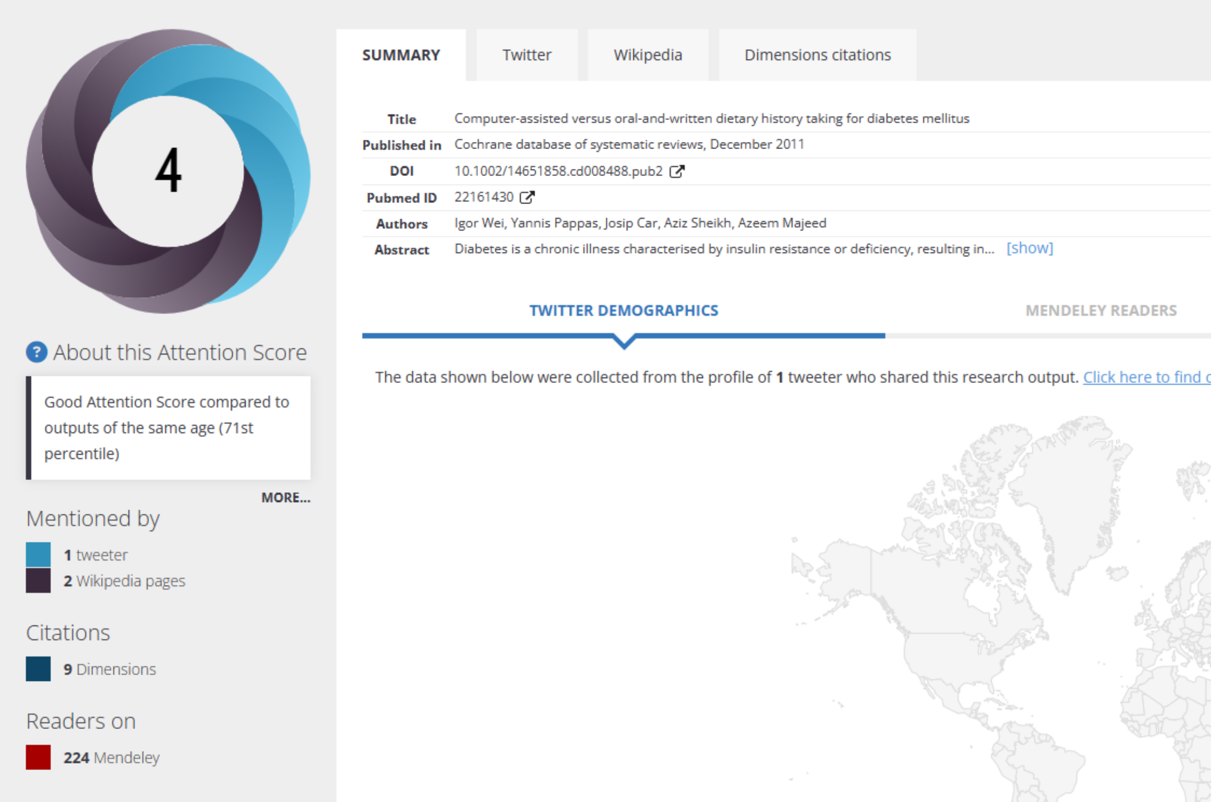

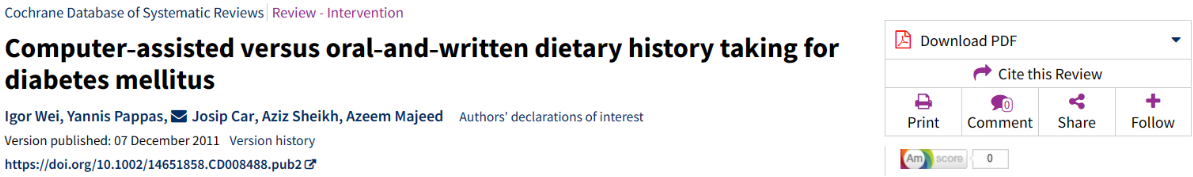

[132] Whitford, Wallis, Dowswell, West and Renfrew (2017) Breastfeeding education and support for women with twins or higher order multiples

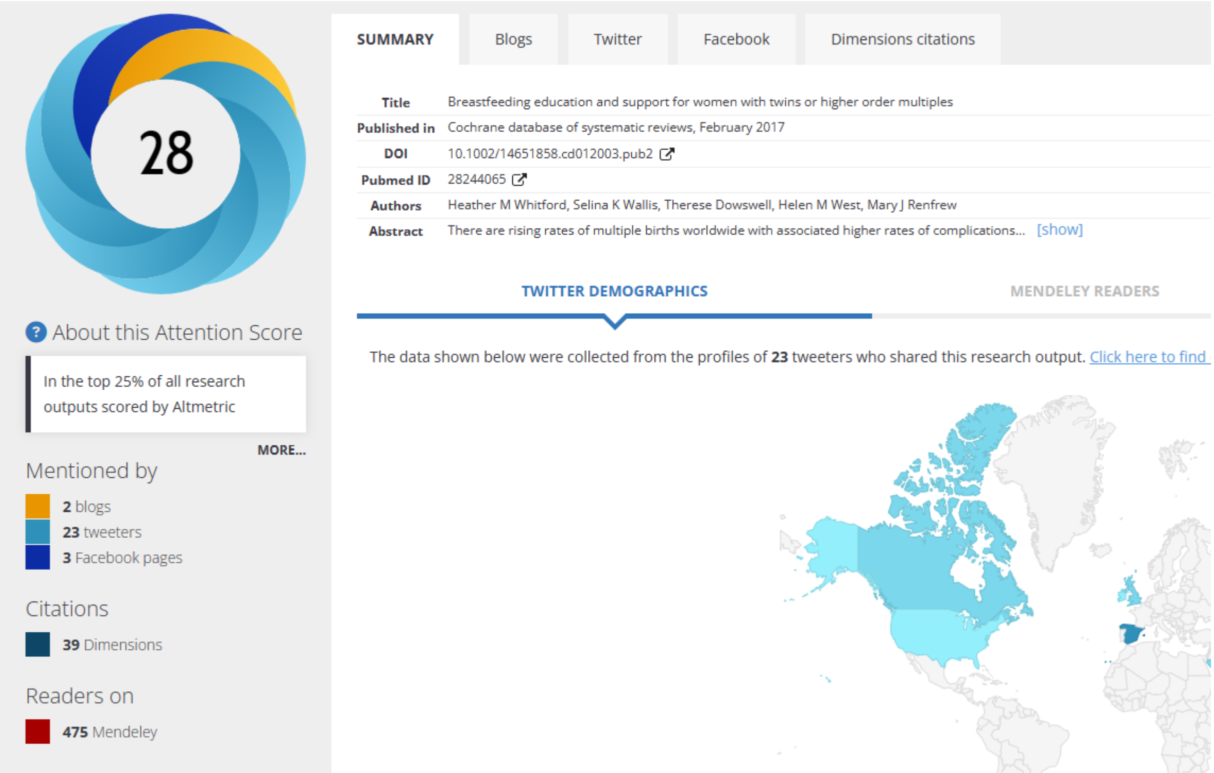

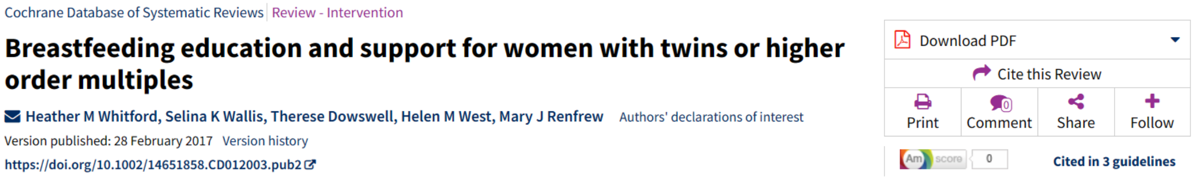

[133] Whittaker, McRobbie, Bullen, Rodgers, Gu and Dobson (2019) Mobile phone text messaging and app-based interventions for smoking cessation

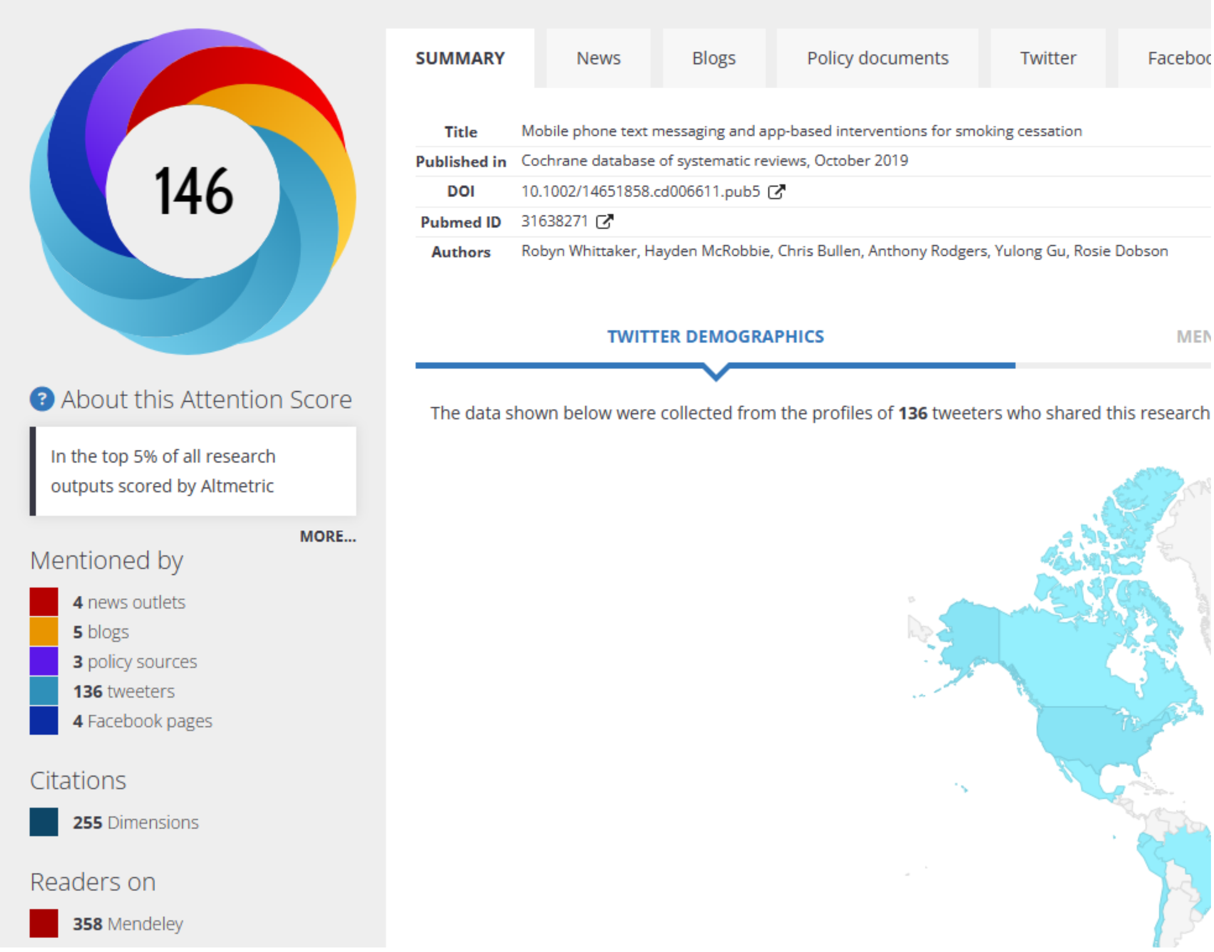

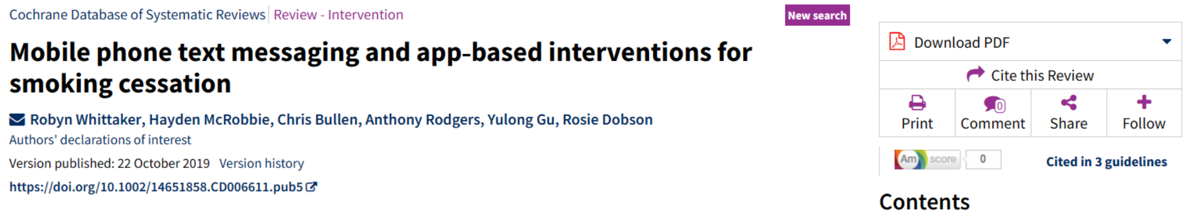

[134] Wieland, Falzon, Sciamanna, Trudeau, Brodney Folse, Schwartz and Davidson (2012) Interactive computer-based interventions for weight loss or weight maintenance in overweight or obese people

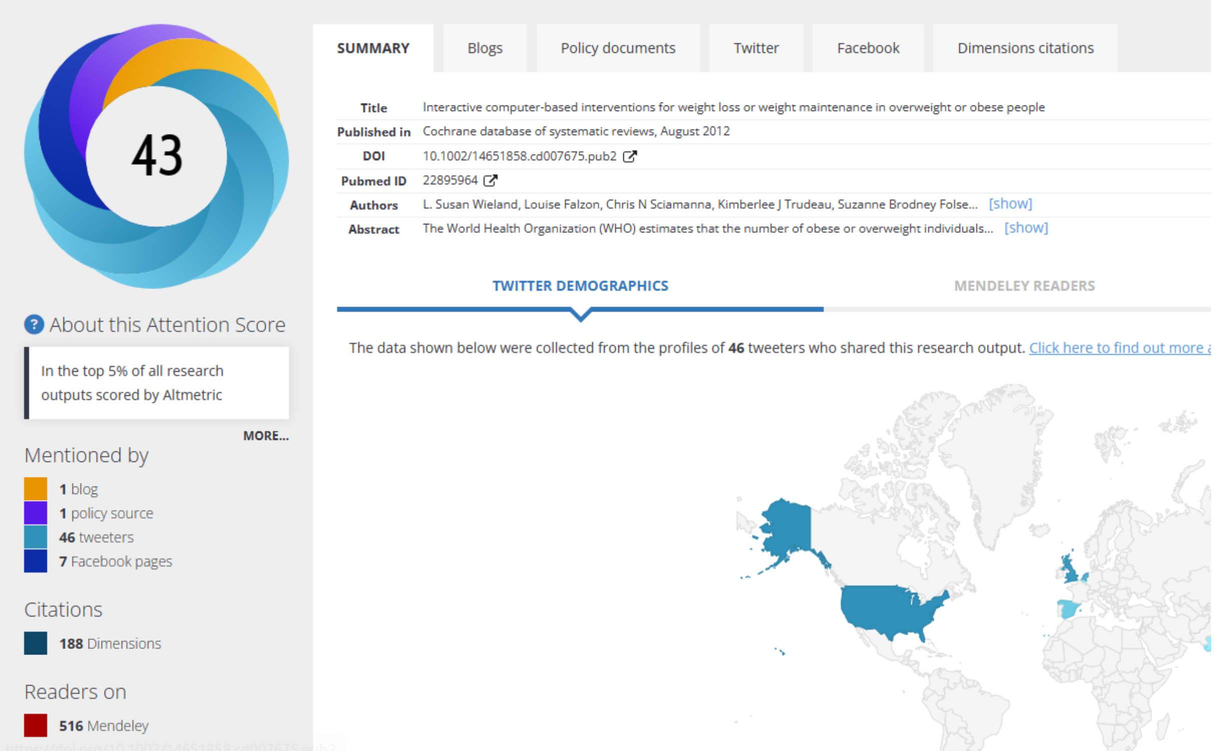

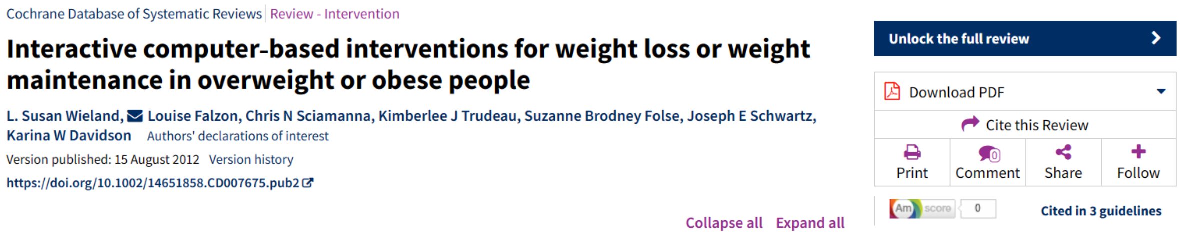

[135] Wilson, Willis, Hendrikz, Le Brocque and Bellamy (2010) Speed cameras for the prevention of road traffic injuries and deaths

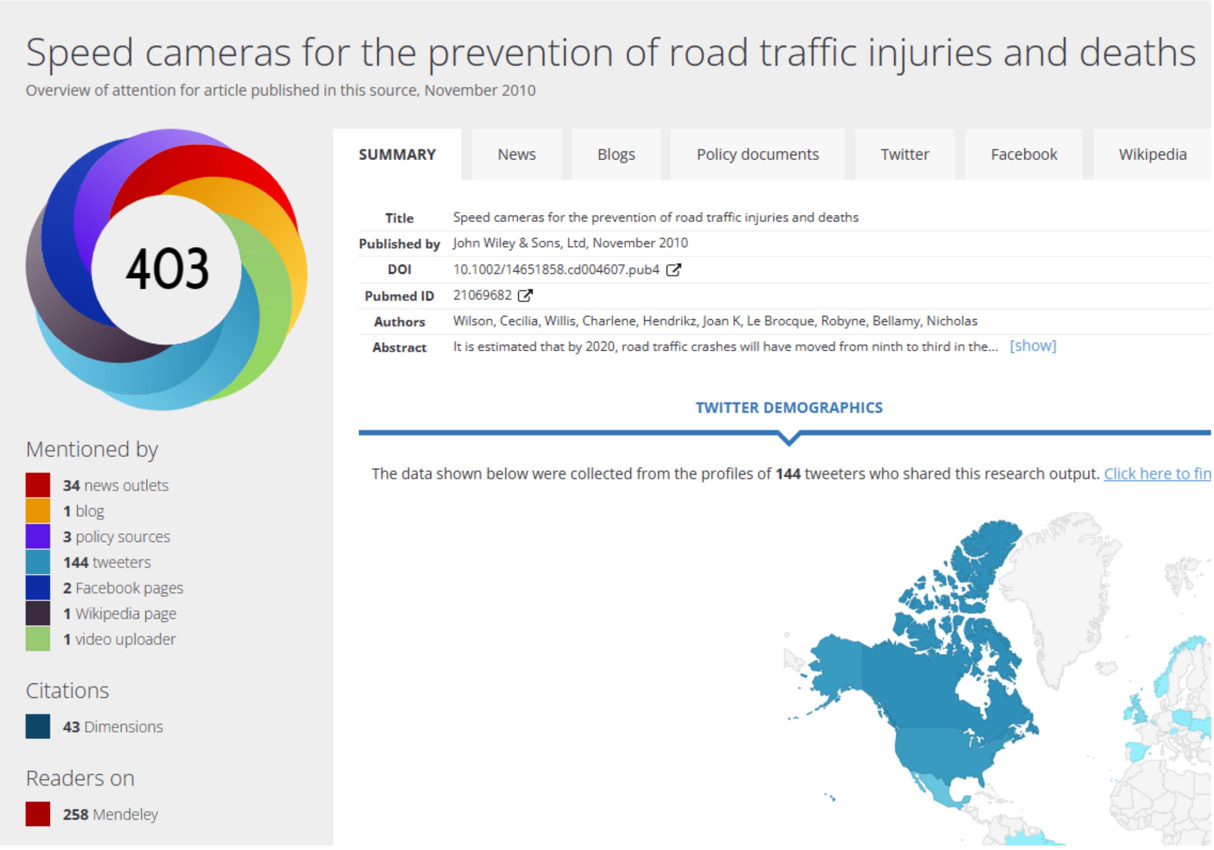

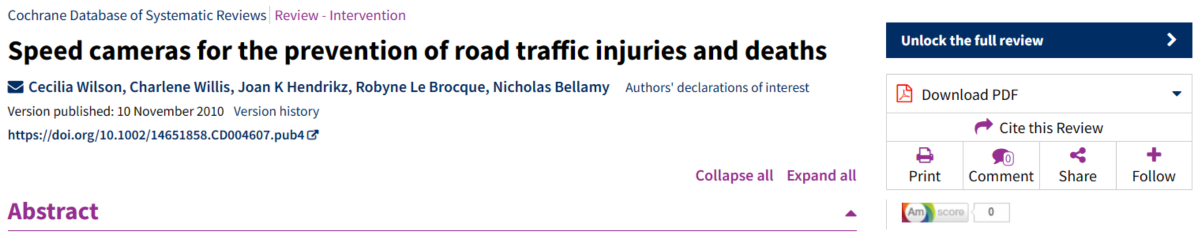

[136] Wong, Smith and Kansra (2023) Digital technology for early identification of exacerbations in people with cystic fibrosis

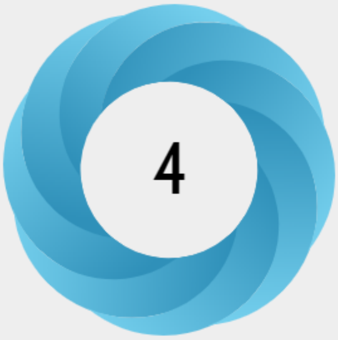

SUMMARY | Twitter

**Title**	Digital technology for early identification of exacerbations in people with cystic fibrosis
**Published in**	Cochrane database of systematic reviews, April 2023
**DOI**	10.1002/14651858.cd014606.pub2
**Pubmed ID**	37057835
**Authors**	Chu-Hai Wong, Sherie Smith, Sonal Kansra

About this Attention Score

Good Attention Score compared to outputs of the same age (68th percentile)

Mentioned by

5 tweeters

TWITTER DEMOGRAPHICS

The data shown below were collected from the profiles of **5** tweeters who shared this research

Cochrane Database of Systematic Reviews | Review - Intervention

Digital technology for early identification of exacerbations in people with cystic fibrosis

Chu-Hai Wong, Sherie Smith, Sonal Kansra Authors' declarations of interest

Version published: 14 April 2023 Version history

https://doi.org/10.1002/14651858.CD014606.pub2

Unlock the full review

Download PDF

Cite this Review

Print | Comment | Share | Follow

Collapse all Expand all

[illegible]

[137] Xyrichis, Iliopoulou, Mackintosh, Bench, Terblanche, Philippou and Sandall (2021) Healthcare stakeholders' perceptions and experiences of factors affecting the implementation of critical care telemedicine (CCT): qualitative evidence synthesis

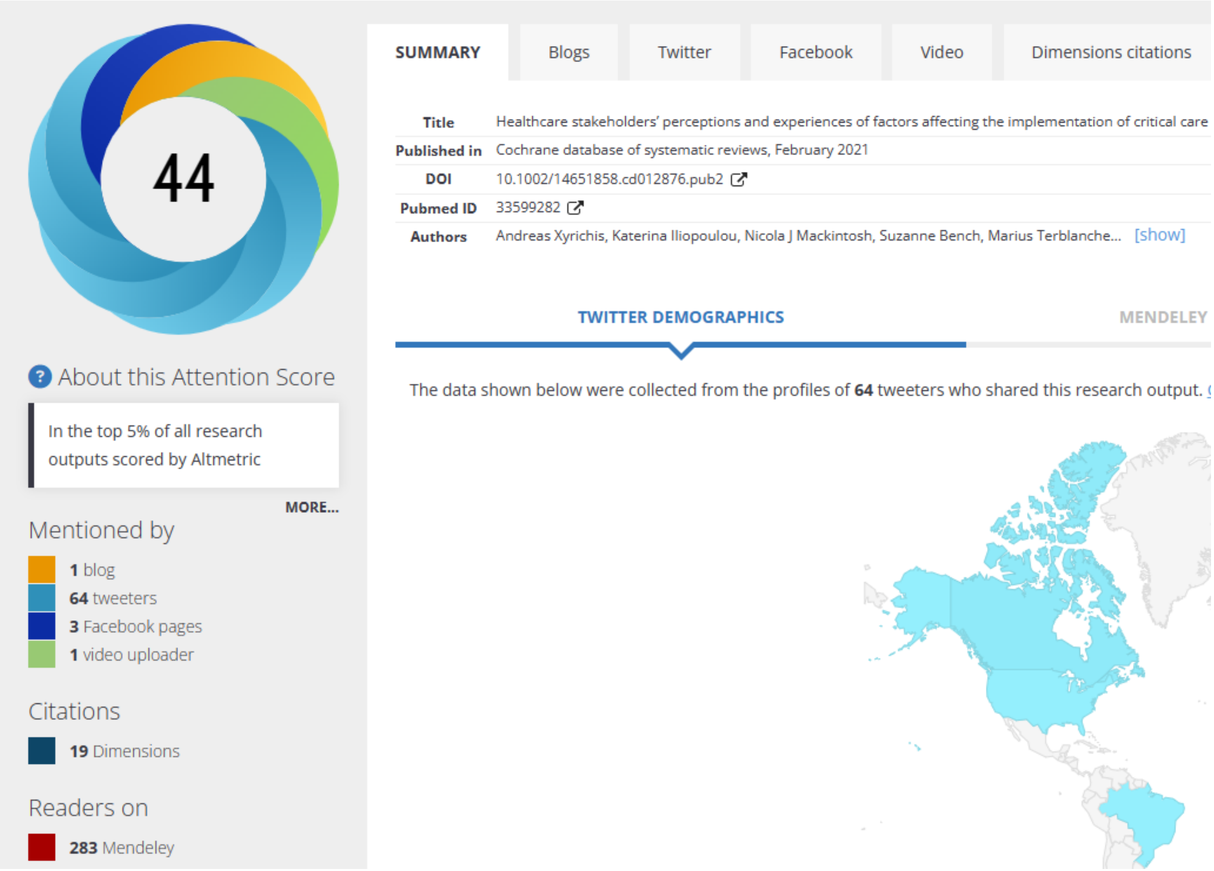

Cochrane Database of Systematic Reviews | Review - Qualitative

Healthcare stakeholders' perceptions and experiences of factors affecting the implementation of critical care telemedicine (CCT): qualitative evidence synthesis

Andreas Xyrichis, Katerina Iliopoulou, Nicola J Mackintosh, Suzanne Bench, Marius Terblanche, Julia Philippou, Jane Sandall Authors' declarations of interest

Version published: 18 February 2021 Version history
https://doi.org/10.1002/14651858.CD012876.pub2

Download PDF
Cite this Review
Print Comment Share Follow
0

Contents

[138] Young and Hopewell (2011) Methods for obtaining unpublished data

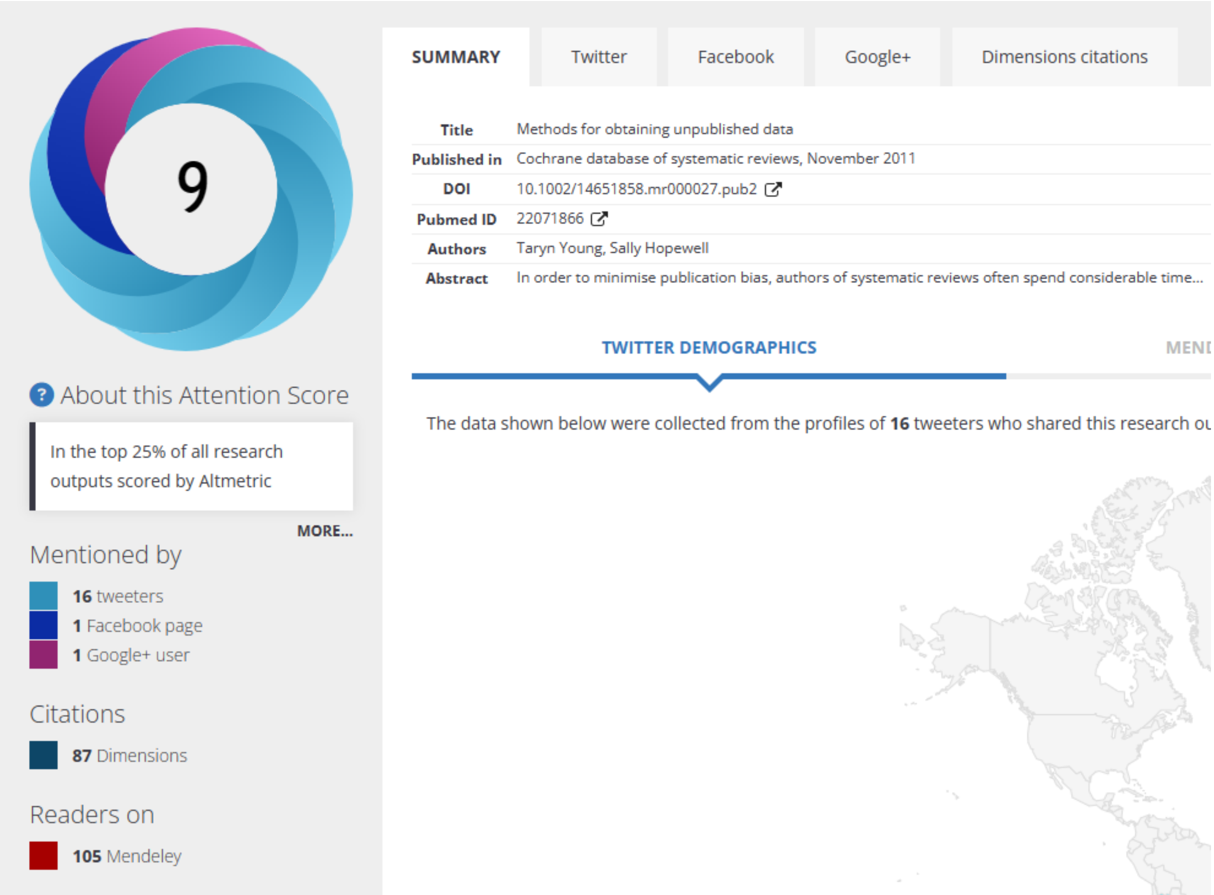

9

About this Attention Score

In the top 25% of all research outputs scored by Altmetric

MORE...

Mentioned by

16 tweeters
1 Facebook page
1 Google+ user

Citations

87 Dimensions

Readers on

105 Mendeley

SUMMARY | Twitter | Facebook | Google+ | Dimensions citations

**Title**	Methods for obtaining unpublished data
**Published in**	Cochrane database of systematic reviews, November 2011
**DOI**	10.1002/14651858.mr000027.pub2
**Pubmed ID**	22071866
**Authors**	Taryn Young, Sally Hopewell
**Abstract**	In order to minimise publication bias, authors of systematic reviews often spend considerable time...

TWITTER DEMOGRAPHICS   MENI

The data shown below were collected from the profiles of **16** tweeters who shared this research ou

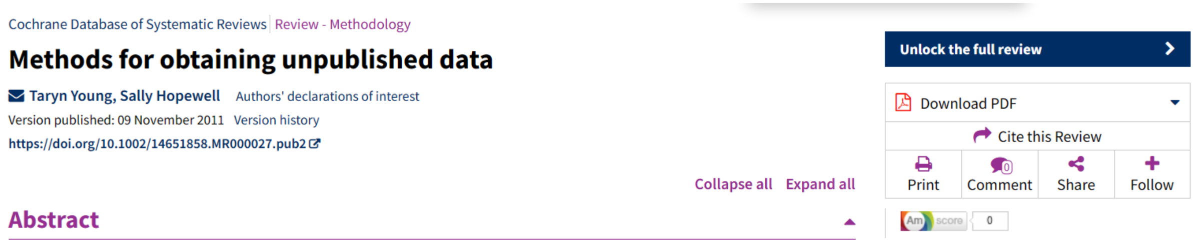

Cochrane Database of Systematic Reviews | Review - Methodology

Methods for obtaining unpublished data

Taryn Young, Sally Hopewell   Authors' declarations of interest

Version published: 09 November 2011   Version history

https://doi.org/10.1002/14651858.MR000027.pub2

Collapse all   Expand all

Unlock the full review

Download PDF

Cite this Review

Print | Comment | Share | Follow

Am score 0

Abstract

1. Aali, G., T. Kariotis, and F. Shokraneh, *Avatar Therapy for people with schizophrenia or related disorders.* Cochrane Database of Systematic Reviews, 2020(5) DOI: 10.1002/14651858.CD011898.pub2.
2. Adler, A.J., et al., *Mobile phone text messaging to improve medication adherence in secondary prevention of cardiovascular disease.* Cochrane Database of Systematic Reviews, 2017(4) DOI: 10.1002/14651858.CD011851.pub2.
3. Agarwal, S., et al., *Tracking health commodity inventory and notifying stock levels via mobile devices: a mixed methods systematic review.* Cochrane Database of Systematic Reviews, 2020(10) DOI: 10.1002/14651858.CD012907.pub2.
4. Agarwal, S., et al., *Decision-support tools via mobile devices to improve quality of care in primary healthcare settings.* Cochrane Database of Systematic Reviews, 2021(7) DOI: 10.1002/14651858.CD012944.pub2.
5. Allida, S., et al., *mHealth education interventions in heart failure.* Cochrane Database of Systematic Reviews, 2020(7) DOI: 10.1002/14651858.CD011845.pub2.
6. Ames, H.M.R., et al., *Clients' perceptions and experiences of targeted digital communication accessible via mobile devices for reproductive, maternal, newborn, child, and adolescent health: a qualitative evidence synthesis.* Cochrane Database of Systematic Reviews, 2019(10) DOI: 10.1002/14651858.CD013447.
7. Ammenwerth, E., et al., *Adult patient access to electronic health records.* Cochrane Database of Systematic Reviews, 2021(2) DOI: 10.1002/14651858.CD012707.pub2.
8. Anglemyer, A., et al., *Digital contact tracing technologies in epidemics: a rapid review.* Cochrane Database of Systematic Reviews, 2020(8) DOI: 10.1002/14651858.CD013699.
9. Archambault, P.M., et al., *Collaborative writing applications in healthcare: effects on professional practice and healthcare outcomes.* Cochrane Database of Systematic Reviews, 2017(5) DOI: 10.1002/14651858.CD011388.pub2.
10. Arditi, C., M. Rège-Walther, P. Durieux, and B. Burnand, *Computer-generated reminders delivered on paper to healthcare professionals: effects on professional practice and healthcare outcomes.* Cochrane Database of Systematic Reviews, 2017(7) DOI: 10.1002/14651858.CD001175.pub4.
11. Atherton, H., P. Sawmynaden, B. Meyer, and J. Car, *Email for the coordination of healthcare appointments and attendance reminders.* Cochrane Database of Systematic Reviews, 2012(8) DOI: 10.1002/14651858.CD007981.pub2.
12. Atherton, H., et al., *Email for clinical communication between patients/caregivers and healthcare professionals.* Cochrane Database of Systematic Reviews, 2012(11) DOI: 10.1002/14651858.CD007978.pub2.
13. Badawy, S.M., K. Morrone, A. Thompson, and T.M. Palermo, *Computer and mobile technology interventions to promote medication adherence and disease management in people with thalassemia.* Cochrane Database of Systematic Reviews, 2019(6) DOI: 10.1002/14651858.CD012900.pub2.
14. Bahar-Fuchs, A., et al., *Cognitive training for people with mild to moderate dementia.* Cochrane Database of Systematic Reviews, 2019(3) DOI: 10.1002/14651858.CD013069.pub2.
15. Bailey, J.V., et al., *Interactive computer-based interventions for sexual health promotion.* Cochrane Database of Systematic Reviews, 2010(9) DOI: 10.1002/14651858.CD006483.pub2.
16. Barth, J., T. Jacob, I. Daha, and J.A. Critchley, *Psychosocial interventions for smoking cessation in patients with coronary heart disease.* Cochrane Database of Systematic Reviews, 2015(7) DOI: 10.1002/14651858.CD006886.pub2.
17. Beishon, L.C., et al., *Diagnostic test accuracy of remote, multidomain cognitive assessment (telephone and video call) for dementia.* Cochrane Database of Systematic Reviews, 2022(4) DOI: 10.1002/14651858.CD013724.pub2.
18. Bittner, A.K., P.D. Yoshinaga, T. Rittiphairoj, and T. Li, *Telerehabilitation for people with low vision.* Cochrane Database of Systematic Reviews, 2023(1) DOI: 10.1002/14651858.CD011019.pub4.

19. Bunn, F., G. Byrne, and S. Kendall, *Telephone consultation and triage: effects on health care use and patient satisfaction.* Cochrane Database of Systematic Reviews, 2004(3) DOI: 10.1002/14651858.CD004180.pub2.
20. Boyle, R., L. Solberg, and M. Fiore, *Use of electronic health records to support smoking cessation.* Cochrane Database of Systematic Reviews, 2014(12) DOI: 10.1002/14651858.CD008743.pub3.
21. Burge, A.T., N.S. Cox, M.J. Abramson, and A.E. Holland, *Interventions for promoting physical activity in people with chronic obstructive pulmonary disease (COPD).* Cochrane Database of Systematic Reviews, 2020(4) DOI: 10.1002/14651858.CD012626.pub2.
22. Car, J., et al., *Interventions for enhancing consumers' online health literacy.* Cochrane Database of Systematic Reviews, 2011(6) DOI: 10.1002/14651858.CD007092.pub2.
23. Chan, A., et al., *Digital interventions to improve adherence to maintenance medication in asthma.* Cochrane Database of Systematic Reviews, 2022(6) DOI: 10.1002/14651858.CD013030.pub2.
24. Chuchu, N., et al., *Teledermatology for diagnosing skin cancer in adults.* Cochrane Database of Systematic Reviews, 2018(12) DOI: 10.1002/14651858.CD013193.
25. Chuchu, N., et al., *Smartphone applications for triaging adults with skin lesions that are suspicious for melanoma.* Cochrane Database of Systematic Reviews, 2018(12) DOI: 10.1002/14651858.CD013192.
26. Corry, M., et al., *Telephone interventions, delivered by healthcare professionals, for providing education and psychosocial support for informal caregivers of adults with diagnosed illnesses.* Cochrane Database of Systematic Reviews, 2019(5) DOI: 10.1002/14651858.CD012533.pub2.
27. Cox, N.S., et al., *Telerehabilitation for chronic respiratory disease.* Cochrane Database of Systematic Reviews, 2021(1) DOI: 10.1002/14651858.CD013040.pub2.
28. Dale, J., I.O. Caramlau, A. Lindenmeyer, and S.M. Williams, *Peer support telephone calls for improving health.* Cochrane Database of Systematic Reviews, 2008(4) DOI: 10.1002/14651858.CD006903.pub2.
29. Datta, S.S., R. Daruvala, and A. Kumar, *Psychological interventions for psychosis in adolescents.* Cochrane Database of Systematic Reviews, 2020(7) DOI: 10.1002/14651858.CD009533.pub2.
30. de Jongh, T., et al., *Mobile phone messaging for facilitating self-management of long-term illnesses.* Cochrane Database of Systematic Reviews, 2012(12) DOI: 10.1002/14651858.CD007459.pub2.
31. Dennett, E.J., et al., *Tailored or adapted interventions for adults with chronic obstructive pulmonary disease and at least one other long-term condition: a mixed methods review.* Cochrane Database of Systematic Reviews, 2021(7) DOI: 10.1002/14651858.CD013384.pub2.
32. Devi, R., et al., *Internet-based interventions for the secondary prevention of coronary heart disease.* Cochrane Database of Systematic Reviews, 2015(12) DOI: 10.1002/14651858.CD009386.pub2.
33. Eccleston, C., et al., *Psychological therapies (Internet-delivered) for the management of chronic pain in adults.* Cochrane Database of Systematic Reviews, 2014(2) DOI: 10.1002/14651858.CD010152.pub2.
34. Eccleston, C., et al., *Interventions for the reduction of prescribed opioid use in chronic non-cancer pain.* Cochrane Database of Systematic Reviews, 2017(11) DOI: 10.1002/14651858.CD010323.pub3.
35. Fiander, M., et al., *Interventions to increase the use of electronic health information by healthcare practitioners to improve clinical practice and patient outcomes.* Cochrane Database of Systematic Reviews, 2015(3) DOI: 10.1002/14651858.CD004749.pub3.
36. Fisher, E., et al., *Psychological therapies (remotely delivered) for the management of chronic and recurrent pain in children and adolescents.* Cochrane Database of Systematic Reviews, 2019(4) DOI: 10.1002/14651858.CD011118.pub3.

37. Fleming, P.S., et al., *Non-pharmacological interventions for alleviating pain during orthodontic treatment.* Cochrane Database of Systematic Reviews, 2016(12) DOI: 10.1002/14651858.CD010263.pub2.
38. Flodgren, G., et al., *Interactive telemedicine: effects on professional practice and health care outcomes.* Cochrane Database of Systematic Reviews, 2015(9) DOI: 10.1002/14651858.CD002098.pub2.
39. Foster, C., J. Richards, M. Thorogood, and M. Hillsdon, *Remote and web 2.0 interventions for promoting physical activity.* Cochrane Database of Systematic Reviews, 2013(9) DOI: 10.1002/14651858.CD010395.pub2.
40. Galaal, K., et al., *Interventions for reducing anxiety in women undergoing colposcopy.* Cochrane Database of Systematic Reviews, 2011(12) DOI: 10.1002/14651858.CD006013.pub3.
41. Gates, N.J., et al., *Computerised cognitive training for maintaining cognitive function in cognitively healthy people in midlife.* Cochrane Database of Systematic Reviews, 2019(3) DOI: 10.1002/14651858.CD012278.pub2.
42. Gates, N.J., et al., *Computerised cognitive training for 12 or more weeks for maintaining cognitive function in cognitively healthy people in late life.* Cochrane Database of Systematic Reviews, 2020(2) DOI: 10.1002/14651858.CD012277.pub3.
43. Gates, N.J., et al., *Computerised cognitive training for preventing dementia in people with mild cognitive impairment.* Cochrane Database of Systematic Reviews, 2019(3) DOI: 10.1002/14651858.CD012279.pub2.
44. Gavine, A., et al., *Support for healthy breastfeeding mothers with healthy term babies.* Cochrane Database of Systematic Reviews, 2022(10) DOI: 10.1002/14651858.CD001141.pub6.
45. Gentry, S., M. van-Velthoven, L. Tudor Car, and J. Car, *Telephone delivered interventions for reducing morbidity and mortality in people with HIV infection.* Cochrane Database of Systematic Reviews, 2013(5) DOI: 10.1002/14651858.CD009189.pub2.
46. Gillaizeau, F., et al., *Computerized advice on drug dosage to improve prescribing practice.* Cochrane Database of Systematic Reviews, 2013(11) DOI: 10.1002/14651858.CD002894.pub3.
47. Gonçalves-Bradley, D.C., et al., *Mobile technologies to support healthcare provider to healthcare provider communication and management of care.* Cochrane Database of Systematic Reviews, 2020(8) DOI: 10.1002/14651858.CD012927.pub2.
48. González-Fraile, E., et al., *Remotely delivered information, training and support for informal caregivers of people with dementia.* Cochrane Database of Systematic Reviews, 2021(1) DOI: 10.1002/14651858.CD006440.pub3.
49. Gordon, M., et al., *Remote care through telehealth for people with inflammatory bowel disease.* Cochrane Database of Systematic Reviews, 2023(5) DOI: 10.1002/14651858.CD014821.pub2.
50. Goyder, C., et al., *Email for clinical communication between healthcare professionals.* Cochrane Database of Systematic Reviews, 2015(2) DOI: 10.1002/14651858.CD007979.pub3.
51. Gurol-Urganci, I., et al., *Mobile phone messaging reminders for attendance at healthcare appointments.* Cochrane Database of Systematic Reviews, 2013(12) DOI: 10.1002/14651858.CD007458.pub3.
52. Gurol-Urganci, I., et al., *Mobile phone messaging for communicating results of medical investigations.* Cochrane Database of Systematic Reviews, 2012(6) DOI: 10.1002/14651858.CD007456.pub2.
53. Gurusamy, K.S., J. Vaughan, and B.R. Davidson, *Formal education of patients about to undergo laparoscopic cholecystectomy.* Cochrane Database of Systematic Reviews, 2014(2) DOI: 10.1002/14651858.CD009933.pub2.
54. Handford, C., A.M. Tynan, J.M. Rackal, and R. Glazier, *Setting and organization of care for persons living with HIV/AIDS.* Cochrane Database of Systematic Reviews, 2006(3) DOI: 10.1002/14651858.CD004348.pub2.

55. Horvath, T., H. Azman, G.E. Kennedy, and G.W. Rutherford, *Mobile phone text messaging for promoting adherence to antiretroviral therapy in patients with HIV infection.* Cochrane Database of Systematic Reviews, 2012(3) DOI: 10.1002/14651858.CD009756.
56. Inglis, S.C., et al., *Structured telephone support or non-invasive telemonitoring for patients with heart failure.* Cochrane Database of Systematic Reviews, 2015(10) DOI: 10.1002/14651858.CD007228.pub3.
57. Jacobson Vann, J.C., et al., *Patient reminder and recall interventions to improve immunization rates.* Cochrane Database of Systematic Reviews, 2018(1) DOI: 10.1002/14651858.CD003941.pub3.
58. Janjua, S., et al., *Digital interventions for the management of chronic obstructive pulmonary disease.* Cochrane Database of Systematic Reviews, 2021(4) DOI: 10.1002/14651858.CD013246.pub2.
59. Janjua, S., et al., *Telehealth interventions: remote monitoring and consultations for people with chronic obstructive pulmonary disease (COPD).* Cochrane Database of Systematic Reviews, 2021(7) DOI: 10.1002/14651858.CD013196.pub2.
60. Jawad, A., I. Jawad, and N.A. Alwan, *Interventions using social networking sites to promote contraception in women of reproductive age.* Cochrane Database of Systematic Reviews, 2019(3) DOI: 10.1002/14651858.CD012521.pub2.
61. Kaner, E.F.S., et al., *Personalised digital interventions for reducing hazardous and harmful alcohol consumption in community-dwelling populations.* Cochrane Database of Systematic Reviews, 2017(9) DOI: 10.1002/14651858.CD011479.pub2.
62. Kauppi, K., et al., *Information and communication technology based prompting for treatment compliance for people with serious mental illness.* Cochrane Database of Systematic Reviews, 2014(6) DOI: 10.1002/14651858.CD009960.pub2.
63. Kew, K.M. and C.J. Cates, *Home telemonitoring and remote feedback between clinic visits for asthma.* Cochrane Database of Systematic Reviews, 2016(8) DOI: 10.1002/14651858.CD011714.pub2.
64. Kew, K.M. and C.J. Cates, *Remote versus face-to-face check-ups for asthma.* Cochrane Database of Systematic Reviews, 2016(4) DOI: 10.1002/14651858.CD011715.pub2.
65. Khan, F., B. Amatya, J. Kesselring, and M. Galea, *Telerehabilitation for persons with multiple sclerosis.* Cochrane Database of Systematic Reviews, 2015(4) DOI: 10.1002/14651858.CD010508.pub2.
66. Kobayashi, S., et al., *Assessment and support during early labour for improving birth outcomes.* Cochrane Database of Systematic Reviews, 2017(4) DOI: 10.1002/14651858.CD011516.pub2.
67. Kuster, A.T., et al., *Computer-based versus in-person interventions for preventing and reducing stress in workers.* Cochrane Database of Systematic Reviews, 2017(8) DOI: 10.1002/14651858.CD011899.pub2.
68. Lavender, T., et al., *Telephone support for women during pregnancy and the first six weeks postpartum.* Cochrane Database of Systematic Reviews, 2013(7) DOI: 10.1002/14651858.CD009338.pub2.
69. Laver, K.E., et al., *Telerehabilitation services for stroke.* Cochrane Database of Systematic Reviews, 2020(1) DOI: 10.1002/14651858.CD010255.pub3.
70. Laver, K.E., et al., *Virtual reality for stroke rehabilitation.* Cochrane Database of Systematic Reviews, 2017(11) DOI: 10.1002/14651858.CD008349.pub4.
71. Leon, N., et al., *Routine Health Information System (RHIS) improvements for strengthened health system management.* Cochrane Database of Systematic Reviews, 2020(8) DOI: 10.1002/14651858.CD012012.pub2.
72. Linden, M., et al., *Technological aids for the rehabilitation of memory and executive functioning in children and adolescents with acquired brain injury.* Cochrane Database of Systematic Reviews, 2016(7) DOI: 10.1002/14651858.CD011020.pub2.

73. Lins, S., et al., *Efficacy and experiences of telephone counselling for informal carers of people with dementia.* Cochrane Database of Systematic Reviews, 2014(9) DOI: 10.1002/14651858.CD009126.pub2.
74. Lopez, L.M., et al., *Behavioral interventions for improving dual-method contraceptive use.* Cochrane Database of Systematic Reviews, 2014(3) DOI: 10.1002/14651858.CD010915.pub2.
75. Lynch, E.A., et al., *Activity monitors for increasing physical activity in adult stroke survivors.* Cochrane Database of Systematic Reviews, 2018(7) DOI: 10.1002/14651858.CD012543.pub2.
76. Malaguti, C., S. Dal Corso, S. Janjua, and A.E. Holland, *Supervised maintenance programmes following pulmonary rehabilitation compared to usual care for chronic obstructive pulmonary disease.* Cochrane Database of Systematic Reviews, 2021(8) DOI: 10.1002/14651858.CD013569.pub2.
77. Manyande, A., et al., *Non-pharmacological interventions for assisting the induction of anaesthesia in children.* Cochrane Database of Systematic Reviews, 2015(7) DOI: 10.1002/14651858.CD006447.pub3.
78. Marcano Belisario, J.S., et al., *Smartphone and tablet self management apps for asthma.* Cochrane Database of Systematic Reviews, 2013(11) DOI: 10.1002/14651858.CD010013.pub2.
79. Marcano Belisario, J.S., et al., *Comparison of self-administered survey questionnaire responses collected using mobile apps versus other methods.* Cochrane Database of Systematic Reviews, 2015(7) DOI: 10.1002/14651858.MR000042.pub2.
80. Martin, S., et al., *Smart home technologies for health and social care support.* Cochrane Database of Systematic Reviews, 2008(4) DOI: 10.1002/14651858.CD006412.pub2.
81. Matkin, W., J.M. Ordóñez-Mena, and J. Hartmann-Boyce, *Telephone counselling for smoking cessation.* Cochrane Database of Systematic Reviews, 2019(5) DOI: 10.1002/14651858.CD002850.pub4.
82. Mayo-Wilson, E. and P. Montgomery, *Media-delivered cognitive behavioural therapy and behavioural therapy (self-help) for anxiety disorders in adults.* Cochrane Database of Systematic Reviews, 2013(9) DOI: 10.1002/14651858.CD005330.pub4.
83. McCabe, C., M. McCann, and A.M. Brady, *Computer and mobile technology interventions for self-management in chronic obstructive pulmonary disease.* Cochrane Database of Systematic Reviews, 2017(5) DOI: 10.1002/14651858.CD011425.pub2.
84. McCleery, J., J. Laverty, and T.J. Quinn, *Diagnostic test accuracy of telehealth assessment for dementia and mild cognitive impairment.* Cochrane Database of Systematic Reviews, 2021(7) DOI: 10.1002/14651858.CD013786.pub2.
85. McLean, S., et al., *Telehealthcare for asthma.* Cochrane Database of Systematic Reviews, 2010(10) DOI: 10.1002/14651858.CD007717.pub2.
86. McLean, S., et al., *Telehealthcare for chronic obstructive pulmonary disease.* Cochrane Database of Systematic Reviews, 2011(7) DOI: 10.1002/14651858.CD007718.pub2.
87. Meyer, B., H. Atherton, P. Sawmynaden, and J. Car, *Email for communicating results of diagnostic medical investigations to patients.* Cochrane Database of Systematic Reviews, 2012(8) DOI: 10.1002/14651858.CD007980.pub2.
88. Mistiaen, P. and E. Poot, *Telephone follow-up, initiated by a hospital-based health professional, for postdischarge problems in patients discharged from hospital to home.* Cochrane Database of Systematic Reviews, 2006(4) DOI: 10.1002/14651858.CD004510.pub3.
89. Murray, E., et al., *Interactive Health Communication Applications for people with chronic disease.* Cochrane Database of Systematic Reviews, 2005(4) DOI: 10.1002/14651858.CD004274.pub4.
90. Murtagh, E.M., et al., *Interventions outside the workplace for reducing sedentary behaviour in adults under 60 years of age.* Cochrane Database of Systematic Reviews, 2020(7) DOI: 10.1002/14651858.CD012554.pub2.
91. Nagendran, M., et al., *Virtual reality training for surgical trainees in laparoscopic surgery.* Cochrane Database of Systematic Reviews, 2013(8) DOI: 10.1002/14651858.CD006575.pub3.

92. Noone, C., et al., *Video calls for reducing social isolation and loneliness in older people: a rapid review.* Cochrane Database of Systematic Reviews, 2020(5) DOI: 10.1002/14651858.CD013632.
93. Odendaal, W.A., et al., *Health workers' perceptions and experiences of using mHealth technologies to deliver primary healthcare services: a qualitative evidence synthesis.* Cochrane Database of Systematic Reviews, 2020(3) DOI: 10.1002/14651858.CD011942.pub2.
94. Olthuis, J.V., et al., *Therapist-supported Internet cognitive behavioural therapy for anxiety disorders in adults.* Cochrane Database of Systematic Reviews, 2016(3) DOI: 10.1002/14651858.CD011565.pub2.
95. Pal, K., et al., *Computer-based diabetes self-management interventions for adults with type 2 diabetes mellitus.* Cochrane Database of Systematic Reviews, 2013(3) DOI: 10.1002/14651858.CD008776.pub2.
96. Palmer, M.J., et al., *Targeted client communication via mobile devices for improving maternal, neonatal, and child health.* Cochrane Database of Systematic Reviews, 2020(8) DOI: 10.1002/14651858.CD013679.
97. Palmer, M.J., et al., *Targeted client communication via mobile devices for improving sexual and reproductive health.* Cochrane Database of Systematic Reviews, 2020(8) DOI: 10.1002/14651858.CD013680.
98. Palmer, M.J., et al., *Mobile phone-based interventions for improving adherence to medication prescribed for the primary prevention of cardiovascular disease in adults.* Cochrane Database of Systematic Reviews, 2021(3) DOI: 10.1002/14651858.CD012675.pub3.
99. Pappas, Y., et al., *Computer-assisted versus oral-and-written family history taking for identifying people with elevated risk of type 2 diabetes mellitus.* Cochrane Database of Systematic Reviews, 2011(12) DOI: 10.1002/14651858.CD008489.pub2.
100. Petkovic, J., et al., *Behavioural interventions delivered through interactive social media for health behaviour change, health outcomes, and health equity in the adult population.* Cochrane Database of Systematic Reviews, 2021(5) DOI: 10.1002/14651858.CD012932.pub2.
101. Piromchai, P., et al., *Virtual reality training for improving the skills needed for performing surgery of the ear, nose or throat.* Cochrane Database of Systematic Reviews, 2015(9) DOI: 10.1002/14651858.CD010198.pub2.
102. Pollock, A., et al., *Interventions for improving upper limb function after stroke.* Cochrane Database of Systematic Reviews, 2014(11) DOI: 10.1002/14651858.CD010820.pub2.
103. Posadzki, P., et al., *Automated telephone communication systems for preventive healthcare and management of long-term conditions.* Cochrane Database of Systematic Reviews, 2016(12) DOI: 10.1002/14651858.CD009921.pub2.
104. Raman, P., et al., *Different methods and settings for glucose monitoring for gestational diabetes during pregnancy.* Cochrane Database of Systematic Reviews, 2017(10) DOI: 10.1002/14651858.CD011069.pub2.
105. Ream, E., et al., *Telephone interventions for symptom management in adults with cancer.* Cochrane Database of Systematic Reviews, 2020(6) DOI: 10.1002/14651858.CD007568.pub2.
106. Reeves, S., et al., *Interprofessional collaboration to improve professional practice and healthcare outcomes.* Cochrane Database of Systematic Reviews, 2017(6) DOI: 10.1002/14651858.CD000072.pub3.
107. Richards, J., M. Thorogood, M. Hillsdon, and C. Foster, *Face-to-face versus remote and web 2.0 interventions for promoting physical activity.* Cochrane Database of Systematic Reviews, 2013(9) DOI: 10.1002/14651858.CD010393.pub2.
108. Roberts, M.T., et al., *Video games for people with schizophrenia.* Cochrane Database of Systematic Reviews, 2021(2) DOI: 10.1002/14651858.CD012844.pub2.
109. Sawmynaden, P., H. Atherton, A. Majeed, and J. Car, *Email for the provision of information on disease prevention and health promotion.* Cochrane Database of Systematic Reviews, 2012(11) DOI: 10.1002/14651858.CD007982.pub2.

110. Shojania, K.G., et al., *The effects of on-screen, point of care computer reminders on processes and outcomes of care.* Cochrane Database of Systematic Reviews, 2009(3) DOI: 10.1002/14651858.CD001096.pub2.
111. Simon, N., et al., *Internet-based cognitive and behavioural therapies for post-traumatic stress disorder (PTSD) in adults.* Cochrane Database of Systematic Reviews, 2021(5) DOI: 10.1002/14651858.CD011710.pub3.
112. Sjøstrand, Å., et al., *Non-pharmacological interventions for stuttering in children six years and younger.* Cochrane Database of Systematic Reviews, 2021(9) DOI: 10.1002/14651858.CD013489.pub2.
113. Smith, C., et al., *Mobile phone-based interventions for improving contraception use.* Cochrane Database of Systematic Reviews, 2015(6) DOI: 10.1002/14651858.CD011159.pub2.
114. Smith, S., R. Calthorpe, S. Herbert, and A.R. Smyth, *Digital technology for monitoring adherence to inhaled therapies in people with cystic fibrosis.* Cochrane Database of Systematic Reviews, 2023(2) DOI: 10.1002/14651858.CD013733.pub2.
115. Stevenson, J.K., et al., *eHealth interventions for people with chronic kidney disease.* Cochrane Database of Systematic Reviews, 2019(8) DOI: 10.1002/14651858.CD012379.pub2.
116. Tailor, V., et al., *Binocular versus standard occlusion or blurring treatment for unilateral amblyopia in children aged three to eight years.* Cochrane Database of Systematic Reviews, 2022(2) DOI: 10.1002/14651858.CD011347.pub3.
117. Tan, K., P.R.F. Dear, and S.J. Newell, *Clinical decision support systems for neonatal care.* Cochrane Database of Systematic Reviews, 2005(2) DOI: 10.1002/14651858.CD004211.pub2.
118. Tan, K. and N.M. Lai, *Telemedicine for the support of parents of high-risk newborn infants.* Cochrane Database of Systematic Reviews, 2012(6) DOI: 10.1002/14651858.CD006818.pub2.
119. Taylor, G.M.J., et al., *Internet-based interventions for smoking cessation.* Cochrane Database of Systematic Reviews, 2017(9) DOI: 10.1002/14651858.CD007078.pub5.
120. Thabrew, H., et al., *E-Health interventions for anxiety and depression in children and adolescents with long-term physical conditions.* Cochrane Database of Systematic Reviews, 2018(8) DOI: 10.1002/14651858.CD012489.pub2.
121. Treanor, C.J., et al., *Non-pharmacological interventions for cognitive impairment due to systemic cancer treatment.* Cochrane Database of Systematic Reviews, 2016(8) DOI: 10.1002/14651858.CD011325.pub2.
122. Treweek, S., et al., *Strategies to improve recruitment to randomised trials.* Cochrane Database of Systematic Reviews, 2018(2) DOI: 10.1002/14651858.MR000013.pub6.
123. Tudor Car, L., S. Gentry, M. van-Velthoven, and J. Car, *Telephone communication of HIV testing results for improving knowledge of HIV infection status.* Cochrane Database of Systematic Reviews, 2013(1) DOI: 10.1002/14651858.CD009192.pub2.
124. Välimäki, M., et al., *Virtual reality for treatment compliance for people with serious mental illness.* Cochrane Database of Systematic Reviews, 2014(10) DOI: 10.1002/14651858.CD009928.pub2.
125. van-Velthoven, M., L. Tudor Car, S. Gentry, and J. Car, *Telephone delivered interventions for preventing HIV infection in HIV-negative persons.* Cochrane Database of Systematic Reviews, 2013(5) DOI: 10.1002/14651858.CD009190.pub2.
126. Vaona, A., et al., *E-learning for health professionals.* Cochrane Database of Systematic Reviews, 2018(1) DOI: 10.1002/14651858.CD011736.pub2.
127. Vasudevan, L., et al., *Birth and death notification via mobile devices: a mixed methods systematic review.* Cochrane Database of Systematic Reviews, 2021(7) DOI: 10.1002/14651858.CD012909.pub2.
128. Verbeek, J.H., et al., *Personal protective equipment for preventing highly infectious diseases due to exposure to contaminated body fluids in healthcare staff.* Cochrane Database of Systematic Reviews, 2020(5) DOI: 10.1002/14651858.CD011621.pub5.
129. Vijayaraghavan, M., et al., *Interventions to reduce tobacco use in people experiencing homelessness.* Cochrane Database of Systematic Reviews, 2020(12) DOI: 10.1002/14651858.CD013413.pub2.

130. Vodopivec-Jamsek, V., et al., *Mobile phone messaging for preventive health care.* Cochrane Database of Systematic Reviews, 2012(12) DOI: 10.1002/14651858.CD007457.pub2.
131. Wei, I., et al., *Computer-assisted versus oral-and-written dietary history taking for diabetes mellitus.* Cochrane Database of Systematic Reviews, 2011(12) DOI: 10.1002/14651858.CD008488.pub2.
132. Whitford, H.M., et al., *Breastfeeding education and support for women with twins or higher order multiples.* Cochrane Database of Systematic Reviews, 2017(2) DOI: 10.1002/14651858.CD012003.pub2.
133. Whittaker, R., et al., *Mobile phone text messaging and app-based interventions for smoking cessation.* Cochrane Database of Systematic Reviews, 2019(10) DOI: 10.1002/14651858.CD006611.pub5.
134. Wieland, L.S., et al., *Interactive computer-based interventions for weight loss or weight maintenance in overweight or obese people.* Cochrane Database of Systematic Reviews, 2012(8) DOI: 10.1002/14651858.CD007675.pub2.
135. Wilson, C., et al., *Speed cameras for the prevention of road traffic injuries and deaths.* Cochrane Database of Systematic Reviews, 2010(11) DOI: 10.1002/14651858.CD004607.pub4.
136. Wong, C.H., S. Smith, and S. Kansra, *Digital technology for early identification of exacerbations in people with cystic fibrosis.* Cochrane Database of Systematic Reviews, 2023(4) DOI: 10.1002/14651858.CD014606.pub2.
137. Xyrichis, A., et al., *Healthcare stakeholders' perceptions and experiences of factors affecting the implementation of critical care telemedicine (CCT): qualitative evidence synthesis.* Cochrane Database of Systematic Reviews, 2021(2) DOI: 10.1002/14651858.CD012876.pub2.
138. Young, T. and S. Hopewell, *Methods for obtaining unpublished data.* Cochrane Database of Systematic Reviews, 2011(11) DOI: 10.1002/14651858.MR000027.pub2.
