# Supplementary material for: Online dissemination of Cochrane reviews on digital health technologies: a cross-sectional study
Source: Syst Rev. 2024 May 15;13:133. doi: 10.1186/s13643-024-02557-6 (PMC11095012; doi:10.1186/s13643-024-02557-6)

**Additional file 6**

**Data synthesis**

Table of Contents

[Bibliographic characteristics 2](#_Toc152761794)

[PICO 3](#_Toc152761795)

[Population 3](#_Toc152761796)

[Intervention 4](#_Toc152761797)

[Comparison 5](#_Toc152761798)

[Outcome 5](#_Toc152761799)

[Outcome strength of evidence 7](#_Toc152761800)

[Dissemination data from Cochrane website 9](#_Toc152761801)

[Dissemination: clinical guidelines 11](#_Toc152761802)

[Dissemination Altmetric data 12](#_Toc152761803)

[Weighing of Altmetric data 12](#_Toc152761804)

[Dissemination online sources 13](#_Toc152761805)

[Dissemination all mentions 15](#_Toc152761806)

[Dissemination Altmetric Score 18](#_Toc152761807)

[Regression analysis 23](#_Toc152761808)

[Variable distribution 23](#_Toc152761809)

[Variables in regression n=87 28](#_Toc152761810)

[Binary logistic regression analysis n=87 30](#_Toc152761811)

[Sensitivity analysis: Binary logistic regression analysis n=84 32](#_Toc152761812)

## Bibliographic characteristics

| **Statistics** | | | | | | |
| --- | --- | --- | --- | --- | --- | --- |
|  | | publication year | 3b Region of corresponding author | 6 Review type | meta analysis performed yes vs. no | primary studies in review |
| N | Valid | 100 | 100 | 100 | 100 | 100 |
|  | Missing | 0 | 0 | 0 | 0 | 0 |
| Minimum | | 2005 |  |  |  | 1 |
| Maximum | | 2023 |  |  |  | 132 |

| **3b Region of corresponding author** | | | | | |
| --- | --- | --- | --- | --- | --- |
|  | | Frequency | Percent | Valid Percent | Cumulative Percent |
| Valid | Africa | 2 | 2.0 | 2.0 | 2.0 |
|  | Asia | 3 | 3.0 | 3.0 | 5.0 |
|  | Australia | 21 | 21.0 | 21.0 | 26.0 |
|  | Europe | 61 | 61.0 | 61.0 | 87.0 |
|  | North America | 13 | 13.0 | 13.0 | 100.0 |
|  | Total | 100 | 100.0 | 100.0 |  |

| **6 Review type** | | | | | |
| --- | --- | --- | --- | --- | --- |
|  | | Frequency | Percent | Valid Percent | Cumulative Percent |
| Valid | overview of reviews | 1 | 1.0 | 1.0 | 1.0 |
|  | rapid review | 2 | 2.0 | 2.0 | 3.0 |
|  | systematic review | 97 | 97.0 | 97.0 | 100.0 |
|  | Total | 100 | 100.0 | 100.0 |  |

| **meta analysis performed yes vs. no** | | | | | |
| --- | --- | --- | --- | --- | --- |
|  | | Frequency | Percent | Valid Percent | Cumulative Percent |
| Valid | no | 31 | 31.0 | 31.0 | 31.0 |
|  | yes | 69 | 69.0 | 69.0 | 100.0 |
|  | Total | 100 | 100.0 | 100.0 |  |

## PICO

### Population

| **Population by type** | **n studies** |
| --- | --- |
| consumers (clients, patients or carers) | 86 |
| healthcare professionals | 16 |

| **Population by age if consumers** | **n studies** |
| --- | --- |
| any adults or children | 44 |
| only adults | 36 |
| only children | 6 |
| **sum** | **86** |

| **Population by health if consumers (clients, patients or carers)** | **n studies** |
| --- | --- |
| any clinical or healthy | 16 |
| only healthy | 2 |
| only clinical | 68 |
| **sum** | **86** |

| **Disease group or clinical focus if healthy** | **n studies** |
| --- | --- |
| brain | 34 |
| any disease | 16 |
| respiratory | 13 |
| other in <10 reviews | 23 |
| genitourinary | 9 |
| cardiovascular | 4 |
| endocrine | 3 |
| gastrointestinal | 3 |
| genetic | 2 |
| cancer | 1 |
| infectious | 1 |
| NA (only healthcare professionals) | 14 |
| **sum** | **100** |

### Intervention

| **0b Intervention** | | | | | |
| --- | --- | --- | --- | --- | --- |
|  | | Frequency | Percent | Valid Percent | Cumulative Percent |
| Valid | multiple | 75 | 75.0 | 75.0 | 75.0 |
|  | single | 25 | 25.0 | 25.0 | 100.0 |
|  | Total | 100 | 100.0 | 100.0 |  |

| **0c Digital Intervention** | | | | | |
| --- | --- | --- | --- | --- | --- |
|  | | Frequency | Percent | Valid Percent | Cumulative Percent |
| Valid | All digital | 62 | 62.0 | 62.0 | 62.0 |
|  | mixed | 38 | 38.0 | 38.0 | 100.0 |
|  | Total | 100 | 100.0 | 100.0 |  |

| **15 Intervention type (based on Item 13)** | | | | | |
| --- | --- | --- | --- | --- | --- |
|  | | Frequency | Percent | Valid Percent | Cumulative Percent |
| Valid | any | 70 | 70.0 | 70.0 | 70.0 |
|  | mobile | 21 | 21.0 | 21.0 | 91.0 |
|  | not mobile | 9 | 9.0 | 9.0 | 100.0 |
|  | Total | 100 | 100.0 | 100.0 |  |

| **16b Digital device type (based on Item 16a)** | | | | | |
| --- | --- | --- | --- | --- | --- |
|  | | Frequency | Percent | Valid Percent | Cumulative Percent |
| Valid | any | 73 | 73.0 | 73.0 | 73.0 |
|  | computer | 9 | 9.0 | 9.0 | 82.0 |
|  | mobile phone | 12 | 12.0 | 12.0 | 94.0 |
|  | mobile phone, computer | 1 | 1.0 | 1.0 | 95.0 |
|  | mobile phone, tablet | 2 | 2.0 | 2.0 | 97.0 |
|  | mobile phone, tablet, personal digital assistant | 1 | 1.0 | 1.0 | 98.0 |
|  | tablet | 1 | 1.0 | 1.0 | 99.0 |
|  | wearable | 1 | 1.0 | 1.0 | 100.0 |
|  | Total | 100 | 100.0 | 100.0 |  |

### Comparison

| **18 Comparison**  **type (based on Item 17, at least one comparison= digital)** | | | | | |
| --- | --- | --- | --- | --- | --- |
|  | | Frequency | Percent | Valid Percent | Cumulative Percent |
| Valid | digital | 40 | 40.0 | 40.0 | 40.0 |
|  | no comparison | 3 | 3.0 | 3.0 | 43.0 |
|  | not digital | 57 | 57.0 | 57.0 | 100.0 |
|  | Total | 100 | 100.0 | 100.0 |  |

### Outcome

| **Health focus** | **n studies** |
| --- | --- |
| treatment | 56 |
| health promotion and disease prevention | 27 |
| management | 17 |
| **sum** | **100** |

| **Outcome focus** | | **n studies** |
| --- | --- | --- |
| treatment | therapy | 17 |
|  | disease management or monitoring | 23 |
|  | adherence | 9 |
|  | rehabilitation | 7 |
|  | **sum** | **56** |
| health promotion and disease prevention | health education | 17 |
|  | lifestyle monitoring | 10 |
|  | **sum** | **27** |
| management | support systems | 11 |
|  | medical training | 3 |
|  | patient management via electronic health records | 3 |
|  | **sum** | **17** |

| **21b Health focus (based on Item 21a)** | | | | | |
| --- | --- | --- | --- | --- | --- |
|  | | Frequency | Percent | Valid Percent | Cumulative Percent |
| Valid | health promotion and disease prevention | 27 | 100.0 | 100.0 | 100.0 |

| **health focus health promotion and disease prevention** | | | | | |
| --- | --- | --- | --- | --- | --- |
|  | | Frequency | Percent | Valid Percent | Cumulative Percent |
| Valid | any | 5 | 18.5 | 18.5 | 18.5 |
|  | cardiovascular | 1 | 3.7 | 3.7 | 22.2 |
|  | cholecystectomy | 1 | 3.7 | 3.7 | 25.9 |
|  | chronic disease | 1 | 3.7 | 3.7 | 29.6 |
|  | chronic kidney disease | 1 | 3.7 | 3.7 | 33.3 |
|  | dementia | 1 | 3.7 | 3.7 | 37.0 |
|  | immunization | 1 | 3.7 | 3.7 | 40.7 |
|  | obesity | 1 | 3.7 | 3.7 | 44.4 |
|  | pain | 1 | 3.7 | 3.7 | 48.1 |
|  | reproductive | 7 | 25.9 | 25.9 | 74.1 |
|  | social isolation | 1 | 3.7 | 3.7 | 77.8 |
|  | stress | 2 | 7.4 | 7.4 | 85.2 |
|  | substance misuse | 4 | 14.8 | 14.8 | 100.0 |
|  | Total | 27 | 100.0 | 100.0 |  |

## Outcome strength of evidence

| **Statistics** | | | | | | |
| --- | --- | --- | --- | --- | --- | --- |
|  | | outcomes rated | outcomes strong | outcome moderate | outcome low | outcome very low |
| N | Valid | 100 | 87 | 87 | 87 | 87 |
|  | Missing | 0 | 13 | 13 | 13 | 13 |

| **outcomes rated** | | | | | |
| --- | --- | --- | --- | --- | --- |
|  | | Frequency | Percent | Valid Percent | Cumulative Percent |
| Valid | no | 13 | 13.0 | 13.0 | 13.0 |
|  | yes | 87 | 87.0 | 87.0 | 100.0 |
|  | Total | 100 | 100.0 | 100.0 |  |

| **outcomes strong** | | | | | |
| --- | --- | --- | --- | --- | --- |
|  | | Frequency | Percent | Valid Percent | Cumulative Percent |
| Valid | no | 77 | 77.0 | 88.5 | 88.5 |
|  | yes | 10 | 10.0 | 11.5 | 100.0 |
|  | Total | 87 | 87.0 | 100.0 |  |
| Missing | -99 | 13 | 13.0 |  |  |
| Total | | 100 | 100.0 |  |  |

| **outcome moderate** | | | | | |
| --- | --- | --- | --- | --- | --- |
|  | | Frequency | Percent | Valid Percent | Cumulative Percent |
| Valid | no | 42 | 42.0 | 48.3 | 48.3 |
|  | yes | 45 | 45.0 | 51.7 | 100.0 |
|  | Total | 87 | 87.0 | 100.0 |  |
| Missing | -99 | 13 | 13.0 |  |  |
| Total | | 100 | 100.0 |  |  |

| **outcome low** | | | | | |
| --- | --- | --- | --- | --- | --- |
|  | | Frequency | Percent | Valid Percent | Cumulative Percent |
| Valid | no | 16 | 16.0 | 18.4 | 18.4 |
|  | yes | 71 | 71.0 | 81.6 | 100.0 |
|  | Total | 87 | 87.0 | 100.0 |  |
| Missing | -99 | 13 | 13.0 |  |  |
| Total | | 100 | 100.0 |  |  |

| **outcome very low** | | | | | |
| --- | --- | --- | --- | --- | --- |
|  | | Frequency | Percent | Valid Percent | Cumulative Percent |
| Valid | no | 29 | 29.0 | 33.3 | 33.3 |
|  | yes | 58 | 58.0 | 66.7 | 100.0 |
|  | Total | 87 | 87.0 | 100.0 |  |
| Missing | -99 | 13 | 13.0 |  |  |
| Total | | 100 | 100.0 |  |  |

| **Statistics** | | | | | | |
| --- | --- | --- | --- | --- | --- | --- |
|  | | 22 Number of quantitative rated outcomes (based on Summary of findings) | 22a Number of outcomes with strong/high evidence (based on Item 22) | 22b Number of outcomes with moderate evidence (based on Item 22) | 22c Number of outcomes with low evidence (based on Item 22) | 22d Number of outcomes with very low evidence (based on Item 22) |
| N | Valid | 100 | 87 | 87 | 87 | 87 |
|  | Missing | 0 | 13 | 13 | 13 | 13 |
| Mean | | 7.67 | .25 | 1.95 | 3.43 | 3.18 |
| Median | | 6.00 | .00 | 1.00 | 3.00 | 2.00 |
| Mode | | 0 | 0 | 0 | 3 | 0 |
| Std. Deviation | | 7.107 | .892 | 3.114 | 3.633 | 4.334 |
| Minimum | | 0 | 0 | 0 | 0 | 0 |
| Maximum | | 48 | 6 | 20 | 23 | 19 |
| Sum | | 767 | 22 | 170 | 298 | 277 |

## Dissemination data from Cochrane website

| **23 PLS included (from Cochrane Website)** | | | | | |
| --- | --- | --- | --- | --- | --- |
|  | | Frequency | Percent | Valid Percent | Cumulative Percent |
| Valid | yes | 100 | 100.0 | 100.0 | 100.0 |

| **24 PLS OA (from Cochrane Website)** | | | | | |
| --- | --- | --- | --- | --- | --- |
|  | | Frequency | Percent | Valid Percent | Cumulative Percent |
| Valid | yes | 100 | 100.0 | 100.0 | 100.0 |

| **25 PLS type (from Cochrane Website)** | | | | | |
| --- | --- | --- | --- | --- | --- |
|  | | Frequency | Percent | Valid Percent | Cumulative Percent |
| Valid | text-based | 100 | 100.0 | 100.0 | 100.0 |

| **26 PLS translation (from Cochrane Website)** | | | | | |
| --- | --- | --- | --- | --- | --- |
|  | | Frequency | Percent | Valid Percent | Cumulative Percent |
| Valid | yes | 100 | 100.0 | 100.0 | 100.0 |

| **28 PLS language number** | | | | | |
| --- | --- | --- | --- | --- | --- |
|  | | Frequency | Percent | Valid Percent | Cumulative Percent |
| Valid | 3 | 10 | 10.0 | 10.0 | 10.0 |
|  | 4 | 10 | 10.0 | 10.0 | 20.0 |
|  | 5 | 9 | 9.0 | 9.0 | 29.0 |
|  | 6 | 7 | 7.0 | 7.0 | 36.0 |
|  | 7 | 4 | 4.0 | 4.0 | 40.0 |
|  | 8 | 7 | 7.0 | 7.0 | 47.0 |
|  | 9 | 18 | 18.0 | 18.0 | 65.0 |
|  | 10 | 14 | 14.0 | 14.0 | 79.0 |
|  | 11 | 7 | 7.0 | 7.0 | 86.0 |
|  | 12 | 5 | 5.0 | 5.0 | 91.0 |
|  | 13 | 5 | 5.0 | 5.0 | 96.0 |
|  | 14 | 4 | 4.0 | 4.0 | 100.0 |
|  | Total | 100 | 100.0 | 100.0 |  |

| **Languages PLS** | **n studies** |
| --- | --- |
| NA | 0 |
| **English** | **100** |
| German | 54 |
| **Spanish** | **100** |
| **Arabic** | **72** |
| **French** | **88** |
| Croatian | 33 |
| Japanese | 51 |
| South Korean | 24 |
| Malay | 69 |
| Thai | 25 |
| Tamil | 8 |
| Polish | 27 |
| Portuguese | 13 |
| Russian | 52 |
| simple Chinese | 45 |
| traditional Chinese | 40 |
| **Total languages** | **801** |

### Dissemination: clinical guidelines

| **Statistics** | | |
| --- | --- | --- |
| 30q guidelines (based on Item 30) | | |
| N | Valid | 100 |
|  | Missing | 0 |
| Mean | | 2.11 |
| Median | | 1.00 |
| Mode | | 0 |
| Std. Deviation | | 3.168 |
| Minimum | | 0 |
| Maximum | | 18 |
| Sum | | 211 |

| **30q**  **Reviews cited in clinical guidelines**  **(based on Item 30)** | | | | | |
| --- | --- | --- | --- | --- | --- |
|  | | Frequency | Percent | Valid Percent | Cumulative Percent |
| Valid | 0 | 40 | 40.0 | 40.0 | 40.0 |
|  | 1 | 19 | 19.0 | 19.0 | 59.0 |
|  | 2 | 15 | 15.0 | 15.0 | 74.0 |
|  | 3 | 8 | 8.0 | 8.0 | 82.0 |
|  | 4 | 3 | 3.0 | 3.0 | 85.0 |
|  | 5 | 2 | 2.0 | 2.0 | 87.0 |
|  | 6 | 3 | 3.0 | 3.0 | 90.0 |
|  | 7 | 4 | 4.0 | 4.0 | 94.0 |
|  | 8 | 2 | 2.0 | 2.0 | 96.0 |
|  | 11 | 1 | 1.0 | 1.0 | 97.0 |
|  | 12 | 1 | 1.0 | 1.0 | 98.0 |
|  | 13 | 1 | 1.0 | 1.0 | 99.0 |
|  | 18 | 1 | 1.0 | 1.0 | 100.0 |
|  | Total | 100 | 100.0 | 100.0 |  |

## Dissemination Altmetric data

### Weighing of Altmetric data

<https://help.altmetric.com/support/solutions/articles/6000232839-how-are-outputs-scored>.


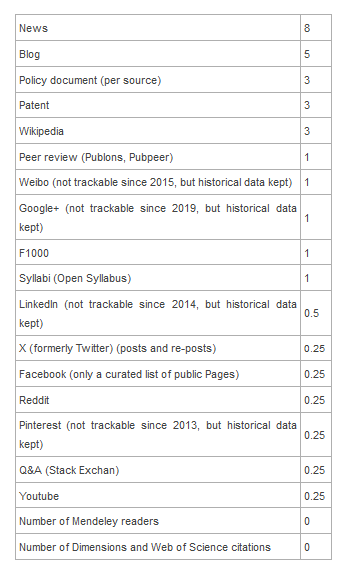


### Dissemination online sources

| **Number** | **Type of channel** | **n studies** |
| --- | --- | --- |
| 1 | Twitter | 99 |
| 2 | Facebook | 69 |
| 3 | Blogs | 48 |
| 4 | Policy sources | 40 |
| 5 | News | 33 |
| 6 | Wikipedia | 30 |
| 7 | Google+ | 6 |
| 8 | Youtube | 3 |
| 9 | Patent | 1 |
| 10 | Peer-reviews | 0 |
| 11 | Weibo | 0 |
| 12 | Syllabi | 0 |
| 13 | F1000 | 0 |
| 14 | LinkedIn | 0 |
| 15 | Reddit | 0 |
| 16 | Q&A | 0 |
| 17 | Pinterest | 0 |
|  | **Total** | **329** |

| **Twitter** | | | | | |
| --- | --- | --- | --- | --- | --- |
|  | | Frequency | Percent | Valid Percent | Cumulative Percent |
| Valid | 0 | 1 | 1.0 | 1.0 | 1.0 |
|  | 1 | 99 | 99.0 | 99.0 | 100.0 |
|  | Total | 100 | 100.0 | 100.0 |  |

| **Facebook** | | | | | |
| --- | --- | --- | --- | --- | --- |
|  | | Frequency | Percent | Valid Percent | Cumulative Percent |
| Valid | 0 | 31 | 31.0 | 31.0 | 31.0 |
|  | 1 | 69 | 69.0 | 69.0 | 100.0 |
|  | Total | 100 | 100.0 | 100.0 |  |

| **blogs** | | | | | |
| --- | --- | --- | --- | --- | --- |
|  | | Frequency | Percent | Valid Percent | Cumulative Percent |
| Valid | 0 | 52 | 52.0 | 52.0 | 52.0 |
|  | 1 | 48 | 48.0 | 48.0 | 100.0 |
|  | Total | 100 | 100.0 | 100.0 |  |

| **policy** | | | | | |
| --- | --- | --- | --- | --- | --- |
|  | | Frequency | Percent | Valid Percent | Cumulative Percent |
| Valid | 0 | 60 | 60.0 | 60.0 | 60.0 |
|  | 1 | 40 | 40.0 | 40.0 | 100.0 |
|  | Total | 100 | 100.0 | 100.0 |  |

| **news** | | | | | |
| --- | --- | --- | --- | --- | --- |
|  | | Frequency | Percent | Valid Percent | Cumulative Percent |
| Valid | 0 | 67 | 67.0 | 67.0 | 67.0 |
|  | 1 | 33 | 33.0 | 33.0 | 100.0 |
|  | Total | 100 | 100.0 | 100.0 |  |

| **Wikipedia** | | | | | |
| --- | --- | --- | --- | --- | --- |
|  | | Frequency | Percent | Valid Percent | Cumulative Percent |
| Valid | 0 | 70 | 70.0 | 70.0 | 70.0 |
|  | 1 | 30 | 30.0 | 30.0 | 100.0 |
|  | Total | 100 | 100.0 | 100.0 |  |

| **Google_plus** | | | | | |
| --- | --- | --- | --- | --- | --- |
|  | | Frequency | Percent | Valid Percent | Cumulative Percent |
| Valid | 0 | 94 | 94.0 | 94.0 | 94.0 |
|  | 1 | 6 | 6.0 | 6.0 | 100.0 |
|  | Total | 100 | 100.0 | 100.0 |  |

| **Youtube** | | | | | |
| --- | --- | --- | --- | --- | --- |
|  | | Frequency | Percent | Valid Percent | Cumulative Percent |
| Valid | 0 | 97 | 97.0 | 97.0 | 97.0 |
|  | 1 | 3 | 3.0 | 3.0 | 100.0 |
|  | Total | 100 | 100.0 | 100.0 |  |

| **patents** | | | | | |
| --- | --- | --- | --- | --- | --- |
|  | | Frequency | Percent | Valid Percent | Cumulative Percent |
| Valid | 0 | 99 | 99.0 | 99.0 | 99.0 |
|  | 1 | 1 | 1.0 | 1.0 | 100.0 |
|  | Total | 100 | 100.0 | 100.0 |  |

### Dissemination all mentions

| **Statistics** | | |
| --- | --- | --- |
| Altmetric_all_posts | | |
| N | Valid | 100 |
|  | Missing | 0 |
| Mean | | 46.61 |
| Median | | 34.00 |
| Mode | | 15 |
| Std. Deviation | | 48.489 |
| Minimum | | 1 |
| Maximum | | 271 |
| Sum | | 4661 |

| **Altmetric_all_posts** | | | | | |
| --- | --- | --- | --- | --- | --- |
|  | | Frequency | Percent | Valid Percent | Cumulative Percent |
| Valid | 1 | 1 | 1.0 | 1.0 | 1.0 |
|  | 2 | 3 | 3.0 | 3.0 | 4.0 |
|  | 3 | 1 | 1.0 | 1.0 | 5.0 |
|  | 4 | 2 | 2.0 | 2.0 | 7.0 |
|  | 5 | 1 | 1.0 | 1.0 | 8.0 |
|  | 6 | 4 | 4.0 | 4.0 | 12.0 |
|  | 7 | 2 | 2.0 | 2.0 | 14.0 |
|  | 9 | 1 | 1.0 | 1.0 | 15.0 |
|  | 10 | 2 | 2.0 | 2.0 | 17.0 |
|  | 11 | 1 | 1.0 | 1.0 | 18.0 |
|  | 12 | 3 | 3.0 | 3.0 | 21.0 |
|  | 13 | 1 | 1.0 | 1.0 | 22.0 |
|  | 14 | 3 | 3.0 | 3.0 | 25.0 |
|  | 15 | 6 | 6.0 | 6.0 | 31.0 |
|  | 17 | 1 | 1.0 | 1.0 | 32.0 |
|  | 19 | 1 | 1.0 | 1.0 | 33.0 |
|  | 21 | 1 | 1.0 | 1.0 | 34.0 |
|  | 22 | 3 | 3.0 | 3.0 | 37.0 |
|  | 23 | 2 | 2.0 | 2.0 | 39.0 |
|  | 26 | 1 | 1.0 | 1.0 | 40.0 |
|  | 27 | 1 | 1.0 | 1.0 | 41.0 |
|  | 28 | 2 | 2.0 | 2.0 | 43.0 |
|  | 29 | 1 | 1.0 | 1.0 | 44.0 |
|  | 30 | 1 | 1.0 | 1.0 | 45.0 |
|  | 31 | 1 | 1.0 | 1.0 | 46.0 |
|  | 32 | 1 | 1.0 | 1.0 | 47.0 |
|  | 33 | 1 | 1.0 | 1.0 | 48.0 |
|  | 34 | 5 | 5.0 | 5.0 | 53.0 |
|  | 35 | 1 | 1.0 | 1.0 | 54.0 |
|  | 36 | 1 | 1.0 | 1.0 | 55.0 |
|  | 39 | 2 | 2.0 | 2.0 | 57.0 |
|  | 41 | 2 | 2.0 | 2.0 | 59.0 |
|  | 42 | 2 | 2.0 | 2.0 | 61.0 |
|  | 44 | 1 | 1.0 | 1.0 | 62.0 |
|  | 46 | 2 | 2.0 | 2.0 | 64.0 |
|  | 47 | 3 | 3.0 | 3.0 | 67.0 |
|  | 48 | 1 | 1.0 | 1.0 | 68.0 |
|  | 50 | 1 | 1.0 | 1.0 | 69.0 |
|  | 51 | 1 | 1.0 | 1.0 | 70.0 |
|  | 53 | 2 | 2.0 | 2.0 | 72.0 |
|  | 54 | 1 | 1.0 | 1.0 | 73.0 |
|  | 55 | 1 | 1.0 | 1.0 | 74.0 |
|  | 56 | 1 | 1.0 | 1.0 | 75.0 |
|  | 58 | 1 | 1.0 | 1.0 | 76.0 |
|  | 59 | 1 | 1.0 | 1.0 | 77.0 |
|  | 60 | 1 | 1.0 | 1.0 | 78.0 |
|  | 64 | 1 | 1.0 | 1.0 | 79.0 |
|  | 67 | 1 | 1.0 | 1.0 | 80.0 |
|  | 69 | 1 | 1.0 | 1.0 | 81.0 |
|  | 70 | 1 | 1.0 | 1.0 | 82.0 |
|  | 72 | 1 | 1.0 | 1.0 | 83.0 |
|  | 78 | 1 | 1.0 | 1.0 | 84.0 |
|  | 80 | 1 | 1.0 | 1.0 | 85.0 |
|  | 82 | 1 | 1.0 | 1.0 | 86.0 |
|  | 87 | 1 | 1.0 | 1.0 | 87.0 |
|  | 93 | 1 | 1.0 | 1.0 | 88.0 |
|  | 100 | 1 | 1.0 | 1.0 | 89.0 |
|  | 110 | 2 | 2.0 | 2.0 | 91.0 |
|  | 111 | 1 | 1.0 | 1.0 | 92.0 |
|  | 129 | 1 | 1.0 | 1.0 | 93.0 |
|  | 133 | 1 | 1.0 | 1.0 | 94.0 |
|  | 134 | 1 | 1.0 | 1.0 | 95.0 |
|  | 148 | 1 | 1.0 | 1.0 | 96.0 |
|  | 152 | 1 | 1.0 | 1.0 | 97.0 |
|  | 166 | 1 | 1.0 | 1.0 | 98.0 |
|  | 261 | 1 | 1.0 | 1.0 | 99.0 |
|  | 271 | 1 | 1.0 | 1.0 | 100.0 |
|  | Total | 100 | 100.0 | 100.0 |  |


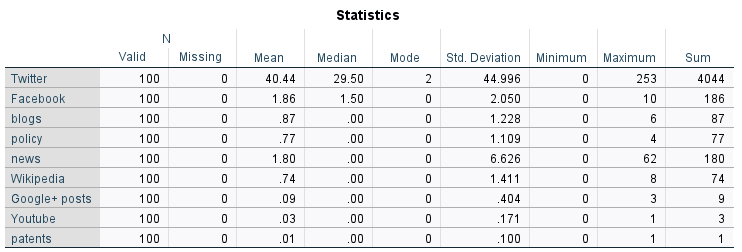


### Dissemination Altmetric Score

| **Statistics** | | | | | | | | | | |  |  |  |  |
| --- | --- | --- | --- | --- | --- | --- | --- | --- | --- | --- | --- | --- | --- | --- |
|  | | | | 29 Altmetric score (from Cochrane Website) | | | | Altmetric score in the top of all research outputs % | | |  |  |  |  |
| N | Valid | | | 100 | | | | 90 | | |  |  |  |  |
|  | Missing | | | 0 | | | | 10 | | |  |  |  |  |
| Mean | | | | 48.38 | | | | 14.11 | | |  |  |  |  |
| Median | | | | 28.50 | | | | 5.00 | | |  |  |  |  |
| Mode | | | | 4^a^ | | | | 5 | | |  |  |  |  |
| Std. Deviation | | | | 67.344 | | | | 10.016 | | |  |  |  |  |
| Minimum | | | | 1 | | | | 5 | | |  |  |  |  |
| Maximum | | | | 553 | | | | 25 | | |  |  |  |  |
| a. Multiple modes exist. The smallest value is shown | | | | | | | | | | |  |  |  |  |
| **29 Altmetric score (from Cochrane Website)** | | | | | | | | | | | | | |  |
|  | | | Frequency | | | Percent | | | Valid Percent | | | Cumulative Percent | |  |
| Valid | 1 | | 3 | | | 3.0 | | | 3.0 | | | 3.0 | |  |
|  | 3 | | 2 | | | 2.0 | | | 2.0 | | | 5.0 | |  |
|  | 4 | | 4 | | | 4.0 | | | 4.0 | | | 9.0 | |  |
|  | 6 | | 1 | | | 1.0 | | | 1.0 | | | 10.0 | |  |
|  | 7 | | 1 | | | 1.0 | | | 1.0 | | | 11.0 | |  |
|  | 8 | | 2 | | | 2.0 | | | 2.0 | | | 13.0 | |  |
|  | 9 | | 1 | | | 1.0 | | | 1.0 | | | 14.0 | |  |
|  | 10 | | 4 | | | 4.0 | | | 4.0 | | | 18.0 | |  |
|  | 11 | | 2 | | | 2.0 | | | 2.0 | | | 20.0 | |  |
|  | 12 | | 1 | | | 1.0 | | | 1.0 | | | 21.0 | |  |
|  | 13 | | 3 | | | 3.0 | | | 3.0 | | | 24.0 | |  |
|  | 14 | | 1 | | | 1.0 | | | 1.0 | | | 25.0 | |  |
|  | 15 | | 1 | | | 1.0 | | | 1.0 | | | 26.0 | |  |
|  | 17 | | 2 | | | 2.0 | | | 2.0 | | | 28.0 | |  |
|  | 19 | | 3 | | | 3.0 | | | 3.0 | | | 31.0 | |  |
|  | 20 | | 2 | | | 2.0 | | | 2.0 | | | 33.0 | |  |
|  | 21 | | 2 | | | 2.0 | | | 2.0 | | | 35.0 | |  |
|  | 22 | | 3 | | | 3.0 | | | 3.0 | | | 38.0 | |  |
|  | 23 | | 3 | | | 3.0 | | | 3.0 | | | 41.0 | |  |
|  | 24 | | 1 | | | 1.0 | | | 1.0 | | | 42.0 | |  |
|  | 26 | | 3 | | | 3.0 | | | 3.0 | | | 45.0 | |  |
|  | 27 | | 4 | | | 4.0 | | | 4.0 | | | 49.0 | |  |
|  | 28 | | 1 | | | 1.0 | | | 1.0 | | | 50.0 | |  |
|  | 29 | | 1 | | | 1.0 | | | 1.0 | | | 51.0 | |  |
|  | 30 | | 1 | | | 1.0 | | | 1.0 | | | 52.0 | |  |
|  | 31 | | 1 | | | 1.0 | | | 1.0 | | | 53.0 | |  |
|  | 33 | | 2 | | | 2.0 | | | 2.0 | | | 55.0 | |  |
|  | 34 | | 2 | | | 2.0 | | | 2.0 | | | 57.0 | |  |
|  | 35 | | 1 | | | 1.0 | | | 1.0 | | | 58.0 | |  |
|  | 36 | | 3 | | | 3.0 | | | 3.0 | | | 61.0 | |  |
|  | 37 | | 2 | | | 2.0 | | | 2.0 | | | 63.0 | |  |
|  | 38 | | 2 | | | 2.0 | | | 2.0 | | | 65.0 | |  |
|  | 41 | | 3 | | | 3.0 | | | 3.0 | | | 68.0 | |  |
|  | 43 | | 1 | | | 1.0 | | | 1.0 | | | 69.0 | |  |
|  | 45 | | 2 | | | 2.0 | | | 2.0 | | | 71.0 | |  |
|  | 46 | | 1 | | | 1.0 | | | 1.0 | | | 72.0 | |  |
|  | 47 | | 1 | | | 1.0 | | | 1.0 | | | 73.0 | |  |
|  | 50 | | 1 | | | 1.0 | | | 1.0 | | | 74.0 | |  |
|  | 56 | | 2 | | | 2.0 | | | 2.0 | | | 76.0 | |  |
|  | 61 | | 1 | | | 1.0 | | | 1.0 | | | 77.0 | |  |
|  | 64 | | 1 | | | 1.0 | | | 1.0 | | | 78.0 | |  |
|  | 66 | | 1 | | | 1.0 | | | 1.0 | | | 79.0 | |  |
|  | 70 | | 1 | | | 1.0 | | | 1.0 | | | 80.0 | |  |
|  | 72 | | 1 | | | 1.0 | | | 1.0 | | | 81.0 | |  |
|  | 73 | | 1 | | | 1.0 | | | 1.0 | | | 82.0 | |  |
|  | 75 | | 1 | | | 1.0 | | | 1.0 | | | 83.0 | |  |
|  | 77 | | 2 | | | 2.0 | | | 2.0 | | | 85.0 | |  |
|  | 85 | | 1 | | | 1.0 | | | 1.0 | | | 86.0 | |  |
|  | 90 | | 1 | | | 1.0 | | | 1.0 | | | 87.0 | |  |
|  | 95 | | 1 | | | 1.0 | | | 1.0 | | | 88.0 | |  |
|  | 96 | | 1 | | | 1.0 | | | 1.0 | | | 89.0 | |  |
|  | 101 | | 1 | | | 1.0 | | | 1.0 | | | 90.0 | |  |
|  | 111 | | 1 | | | 1.0 | | | 1.0 | | | 91.0 | |  |
|  | 134 | | 1 | | | 1.0 | | | 1.0 | | | 92.0 | |  |
|  | 137 | | 1 | | | 1.0 | | | 1.0 | | | 93.0 | |  |
|  | 139 | | 1 | | | 1.0 | | | 1.0 | | | 94.0 | |  |
|  | 142 | | 1 | | | 1.0 | | | 1.0 | | | 95.0 | |  |
|  | 147 | | 1 | | | 1.0 | | | 1.0 | | | 96.0 | |  |
|  | 150 | | 1 | | | 1.0 | | | 1.0 | | | 97.0 | |  |
|  | 207 | | 1 | | | 1.0 | | | 1.0 | | | 98.0 | |  |
|  | 237 | | 1 | | | 1.0 | | | 1.0 | | | 99.0 | |  |
|  | 553 | | 1 | | | 1.0 | | | 1.0 | | | 100.0 | |  |
|  | Total | | 100 | | | 100.0 | | | 100.0 | | |  | |  |
| **Altmetric score in the top of all research outputs %** | | | | | | | | | | | | | | |
|  | | | | | Frequency | | Percent | | | Valid Percent | | | Cumulative Percent | |
| Valid | | 5 | | | 49 | | 49.0 | | | 54.4 | | | 54.4 | |
|  |  | 25 | | | 41 | | 41.0 | | | 45.6 | | | 100.0 | |
|  |  | Total | | | 90 | | 90.0 | | | 100.0 | | |  | |
| Missing | | System | | | 10 | | 10.0 | | |  | | |  | |
| Total | | | | | 100 | | 100.0 | | |  | | |  | |

| **Case Summaries^a^** | | | |
| --- | --- | --- | --- |
|  | | 29 Altmetric score (from Cochrane Website) | Altmetric score in the top of all research outputs % |
| 1 | | 1 | . |
| 2 | | 1 | . |
| 3 | | 1 | . |
| 4 | | 3 | . |
| 5 | | 3 | . |
| 6 | | 4 | . |
| 7 | | 4 | . |
| 8 | | 4 | . |
| 9 | | 4 | . |
| 10 | | 6 | . |
| 11 | | 7 | 25 |
| 12 | | 8 | 25 |
| 13 | | 8 | 25 |
| 14 | | 9 | 25 |
| 15 | | 10 | 25 |
| 16 | | 10 | 25 |
| 17 | | 10 | 25 |
| 18 | | 10 | 25 |
| 19 | | 11 | 25 |
| 20 | | 11 | 25 |
| 21 | | 12 | 25 |
| 22 | | 13 | 25 |
| 23 | | 13 | 25 |
| 24 | | 13 | 25 |
| 25 | | 14 | 25 |
| 26 | | 15 | 25 |
| 27 | | 17 | 25 |
| 28 | | 17 | 25 |
| 29 | | 19 | 25 |
| 30 | | 19 | 25 |
| 31 | | 19 | 25 |
| 32 | | 20 | 25 |
| 33 | | 20 | 25 |
| 34 | | 21 | 25 |
| 35 | | 21 | 25 |
| 36 | | 22 | 25 |
| 37 | | 22 | 25 |
| 38 | | 22 | 25 |
| 39 | | 23 | 25 |
| 40 | | 23 | 25 |
| 41 | | 23 | 25 |
| 42 | | 24 | 25 |
| 43 | | 26 | 25 |
| 44 | | 26 | 25 |
| 45 | | 26 | 25 |
| 46 | | 27 | 25 |
| 47 | | 27 | 25 |
| 48 | | 27 | 25 |
| 49 | | 27 | 25 |
| 50 | | 28 | 25 |
| 51 | | 29 | 25 |
| 52 | | 30 | 5 |
| 53 | | 31 | 5 |
| 54 | | 33 | 5 |
| 55 | | 33 | 5 |
| 56 | | 34 | 5 |
| 57 | | 34 | 5 |
| 58 | | 35 | 5 |
| 59 | | 36 | 5 |
| 60 | | 36 | 5 |
| 61 | | 36 | 5 |
| 62 | | 37 | 5 |
| 63 | | 37 | 5 |
| 64 | | 38 | 5 |
| 65 | | 38 | 5 |
| 66 | | 41 | 5 |
| 67 | | 41 | 5 |
| 68 | | 41 | 5 |
| 69 | | 43 | 5 |
| 70 | | 45 | 5 |
| 71 | | 45 | 5 |
| 72 | | 46 | 5 |
| 73 | | 47 | 5 |
| 74 | | 50 | 5 |
| 75 | | 56 | 5 |
| 76 | | 56 | 5 |
| 77 | | 61 | 5 |
| 78 | | 64 | 5 |
| 79 | | 66 | 5 |
| 80 | | 70 | 5 |
| 81 | | 72 | 5 |
| 82 | | 73 | 5 |
| 83 | | 75 | 5 |
| 84 | | 77 | 5 |
| 85 | | 77 | 5 |
| 86 | | 85 | 5 |
| 87 | | 90 | 5 |
| 88 | | 95 | 5 |
| 89 | | 96 | 5 |
| 90 | | 101 | 5 |
| 91 | | 111 | 5 |
| 92 | | 134 | 5 |
| 93 | | 137 | 5 |
| 94 | | 139 | 5 |
| 95 | | 142 | 5 |
| 96 | | 147 | 5 |
| 97 | | 150 | 5 |
| 98 | | 207 | 5 |
| 99 | | 237 | 5 |
| 100 | | 553 | 5 |
| Total | N | 100 | 90 |
| a. Limited to first 100 cases. | | | |

## Regression analysis

### Variable distribution


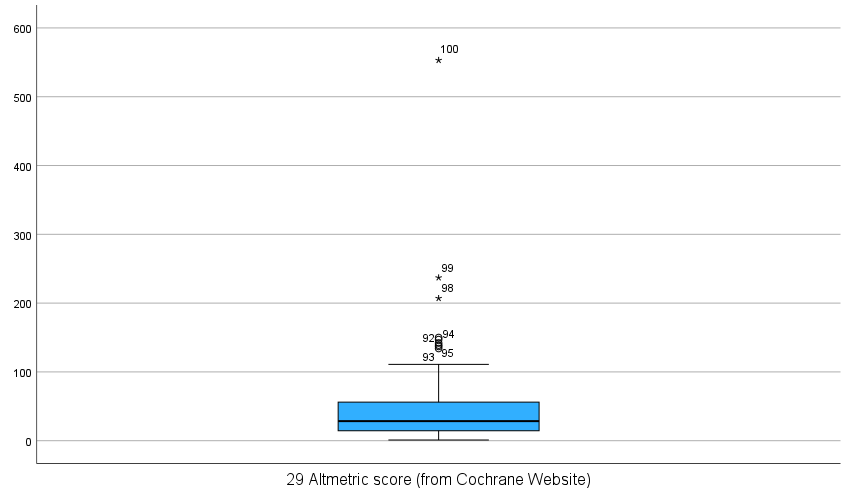


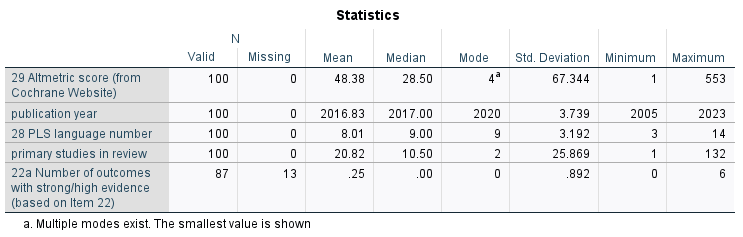


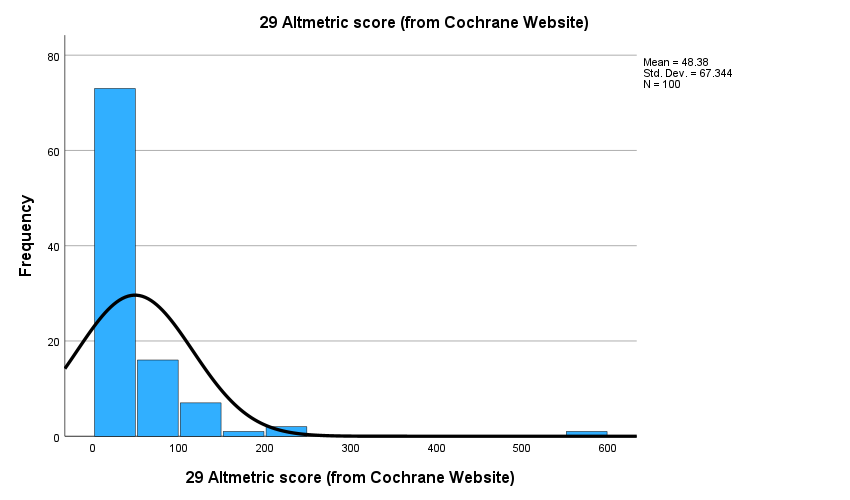


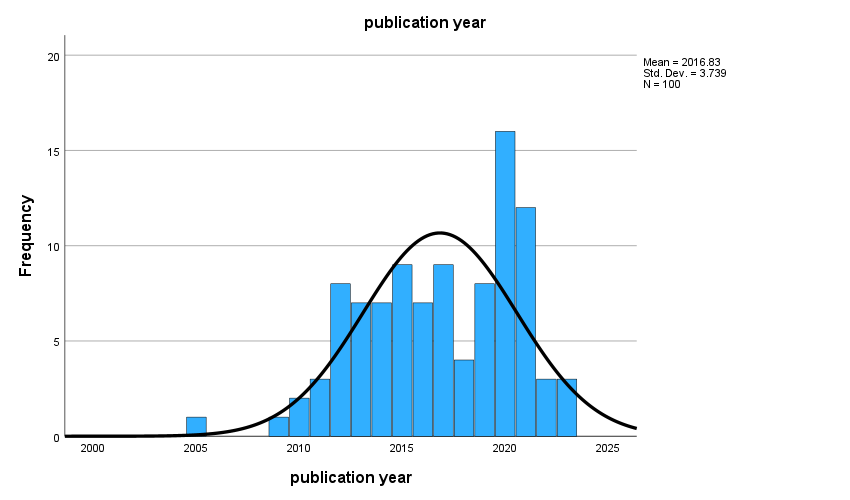


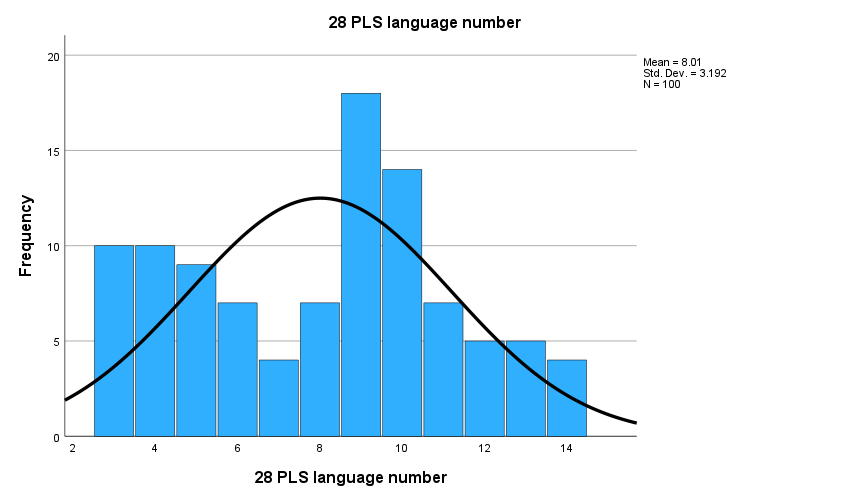


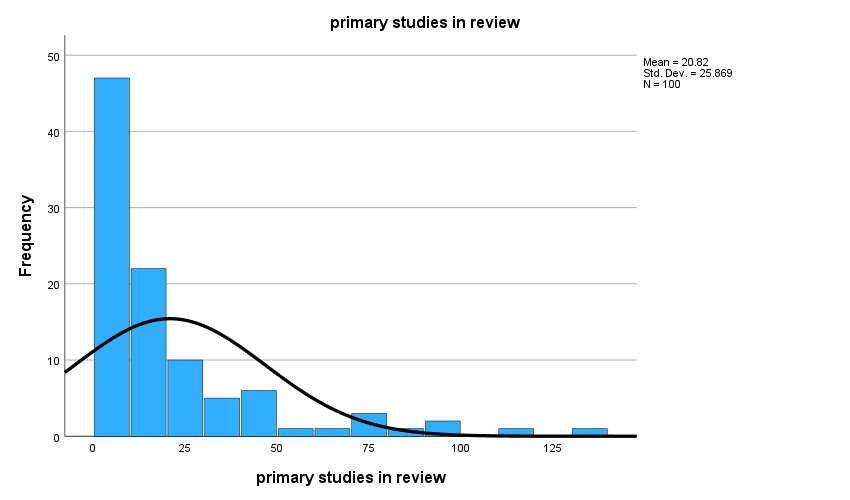


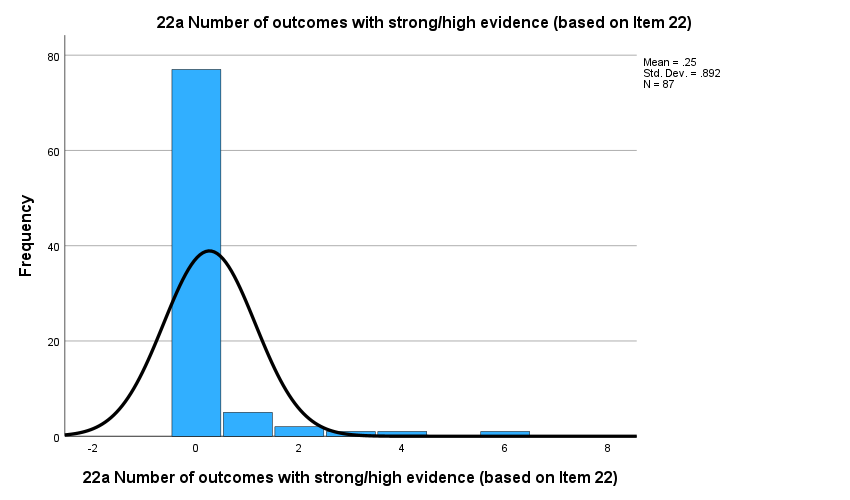


| **Altmetric Score high vs. low (R)** | | | | | |
| --- | --- | --- | --- | --- | --- |
|  | | Frequency | Percent | Valid Percent | Cumulative Percent |
| Valid | low < top 25% | 51 | 51.0 | 51.0 | 51.0 |
|  | high top 5% | 49 | 49.0 | 49.0 | 100.0 |
|  | Total | 100 | 100.0 | 100.0 |  |

| **primary studies many vs. few (R)** | | | | | |
| --- | --- | --- | --- | --- | --- |
|  | | Frequency | Percent | Valid Percent | Cumulative Percent |
| Valid | few (1-10) | 50 | 50.0 | 50.0 | 50.0 |
|  | many (11-132) | 50 | 50.0 | 50.0 | 100.0 |
|  | Total | 100 | 100.0 | 100.0 |  |

| **meta analysis performed yes vs. no (R)** | | | | | |
| --- | --- | --- | --- | --- | --- |
|  | | Frequency | Percent | Valid Percent | Cumulative Percent |
| Valid | no | 31 | 31.0 | 31.0 | 31.0 |
|  | yes | 69 | 69.0 | 69.0 | 100.0 |
|  | Total | 100 | 100.0 | 100.0 |  |

| **outcomes high-moderate vs. low-very low (R)** | | | | | |
| --- | --- | --- | --- | --- | --- |
|  | | Frequency | Percent | Valid Percent | Cumulative Percent |
| Valid | no | 41 | 41.0 | 47.1 | 47.1 |
|  | yes | 46 | 46.0 | 52.9 | 100.0 |
|  | Total | 87 | 87.0 | 100.0 |  |
| Missing | -99 | 13 | 13.0 |  |  |
| Total | | 100 | 100.0 |  |  |

### Variables in regression n=87

**13 reviews without strength of evidence rating excluded**


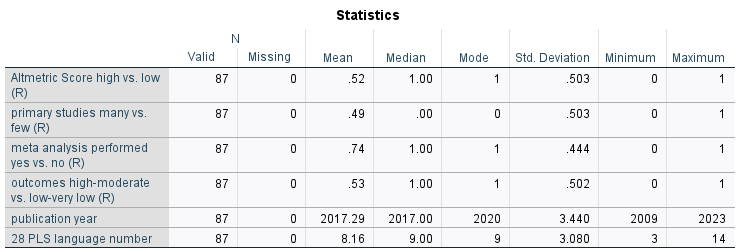


| **Altmetric Score high vs. low (R)** | | | | | |
| --- | --- | --- | --- | --- | --- |
|  | | Frequency | Percent | Valid Percent | Cumulative Percent |
| Valid | low < top 25% | 42 | 48.3 | 48.3 | 48.3 |
|  | high top 5% | 45 | 51.7 | 51.7 | 100.0 |
|  | Total | 87 | 100.0 | 100.0 |  |

| **primary studies many vs. few (R)** | | | | | |
| --- | --- | --- | --- | --- | --- |
|  | | Frequency | Percent | Valid Percent | Cumulative Percent |
| Valid | few (1-10) | 44 | 50.6 | 50.6 | 50.6 |
|  | many (11-132) | 43 | 49.4 | 49.4 | 100.0 |
|  | Total | 87 | 100.0 | 100.0 |  |

| **meta analysis performed yes vs. no (R)** | | | | | |
| --- | --- | --- | --- | --- | --- |
|  | | Frequency | Percent | Valid Percent | Cumulative Percent |
| Valid | no | 23 | 26.4 | 26.4 | 26.4 |
|  | yes | 64 | 73.6 | 73.6 | 100.0 |
|  | Total | 87 | 100.0 | 100.0 |  |

| **outcomes high-moderate vs. low-very low (R)** | | | | | |
| --- | --- | --- | --- | --- | --- |
|  | | Frequency | Percent | Valid Percent | Cumulative Percent |
| Valid | no | 41 | 47.1 | 47.1 | 47.1 |
|  | yes | 46 | 52.9 | 52.9 | 100.0 |
|  | Total | 87 | 100.0 | 100.0 |  |


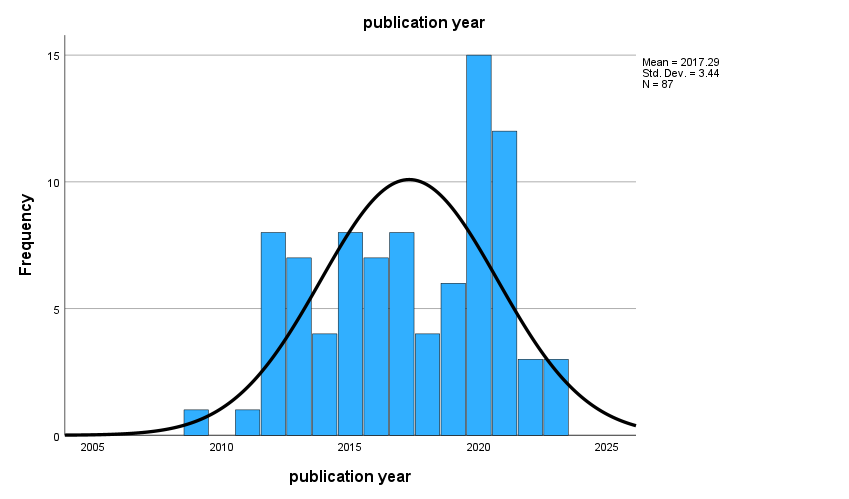


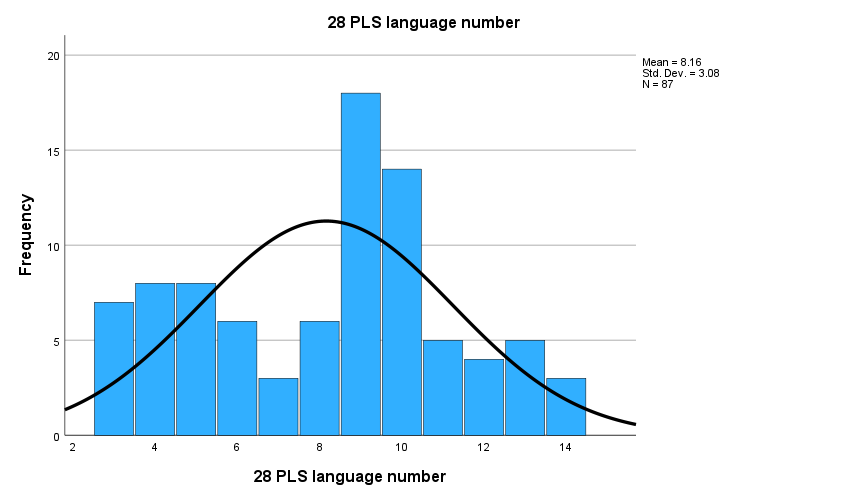


### Binary logistic regression analysis n=87

| **Case Processing Summary** | | | |
| --- | --- | --- | --- |
| Unweighted Cases^a^ | | N | Percent |
| Selected Cases | Included in Analysis | 87 | 87.0 |
|  | Missing Cases | 13 | 13.0 |
|  | Total | 100 | 100.0 |
| Unselected Cases | | 0 | .0 |
| Total | | 100 | 100.0 |
| a. If weight is in effect, see classification table for the total number of cases. | | | |

| **Dependent Variable Encoding** | |
| --- | --- |
| Original Value | Internal Value |
| low < top 25% | 0 |
| high top 5% | 1 |

| **Omnibus Tests of Model Coefficients** | | | | |
| --- | --- | --- | --- | --- |
|  | | Chi-square | df | Sig. |
| Step 1 | Step | 12.837 | 5 | .025 |
|  | Block | 12.837 | 5 | .025 |
|  | Model | 12.837 | 5 | .025 |

| **Model Summary** | | | |
| --- | --- | --- | --- |
| Step | -2 Log likelihood | Cox & Snell R Square | Nagelkerke R Square |
| 1 | 107.667^a^ | .137 | .183 |
| a. Estimation terminated at iteration number 5 because parameter estimates changed by less than .001. | | | |

| **Hosmer and Lemeshow Test** | | | |
| --- | --- | --- | --- |
| Step | Chi-square | df | Sig. |
| 1 | 6.306 | 8 | .613 |


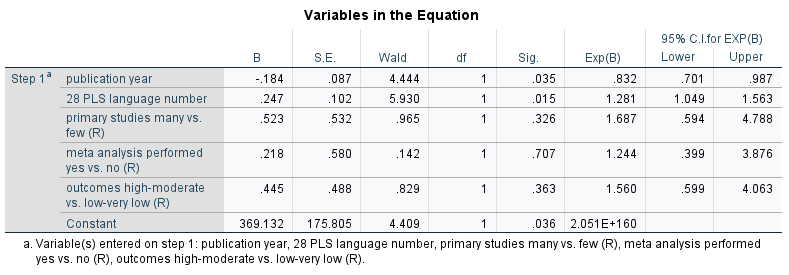


### Sensitivity analysis: Binary logistic regression analysis n=84

Three reviews with the highest Altmetric Scores of >150 removed from the analysis.

| **Case Processing Summary** | | | |
| --- | --- | --- | --- |
| Unweighted Cases^a^ | | N | Percent |
| Selected Cases | Included in Analysis | 84 | 100.0 |
|  | Missing Cases | 0 | .0 |
|  | Total | 84 | 100.0 |
| Unselected Cases | | 0 | .0 |
| Total | | 84 | 100.0 |
| a. If weight is in effect, see classification table for the total number of cases. | | | |

| **Omnibus Tests of Model Coefficients** | | | | |
| --- | --- | --- | --- | --- |
|  | | Chi-square | df | Sig. |
| Step 1 | Step | 11.354 | 5 | .045 |
|  | Block | 11.354 | 5 | .045 |
|  | Model | 11.354 | 5 | .045 |

| **Model Summary** | | | |
| --- | --- | --- | --- |
| Step | -2 Log likelihood | Cox & Snell R Square | Nagelkerke R Square |
| 1 | 105.095^a^ | .126 | .169 |
| a. Estimation terminated at iteration number 5 because parameter estimates changed by less than .001. | | | |

| **Hosmer and Lemeshow Test** | | | |
| --- | --- | --- | --- |
| Step | Chi-square | df | Sig. |
| 1 | 6.266 | 8 | .618 |


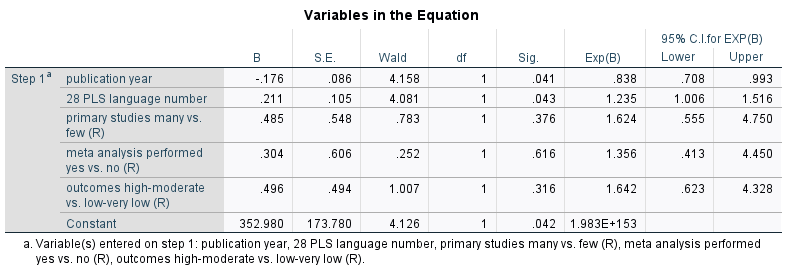

Supplement: Supplementary file 6 — Additional file 6. Data synthesis. [file 13643_2024_2557_MOESM6_ESM.docx]
