# Supplementary material for: Online dissemination of Cochrane reviews on digital health technologies: a cross-sectional study
Source: Syst Rev. 2024 May 15;13:133. doi: 10.1186/s13643-024-02557-6 (PMC11095012; doi:10.1186/s13643-024-02557-6)
Supplement: Supplementary file 7 — Additional file 7. Plain Language Summary. [file 13643_2024_2557_MOESM7_ESM.docx]

**Additional file 7**

# Plain language summary

| **Language** | **Text** |
| --- | --- |
| English | In this study we looked at online mentions of 100 scientific papers about digital health technologies in 2023. These papers were often mentioned online, especially in X/Twitter or Facebook. Older papers and papers with simple summaries in more languages were more often mentioned online. Papers on COVID-19 topics were most often mentioned online. We would like that papers are mentioned online more often if their results have high quality. |
| German | In dieser Studie untersuchten wir, in welchen Online-Ressourcen 100 wissenschaftliche Arbeiten über digitale Gesundheitstechnologien im Jahr 2023 erwähnt wurden. Diese Arbeiten wurden häufig online erwähnt, insbesondere in X/Twitter oder Facebook. Ältere Arbeiten und Arbeiten mit einer leicht verständlich geschriebenen Zusammenfassung in mehreren Sprachen wurden häufiger online erwähnt. Arbeiten zu COVID-19-Themen wurden online am häufigsten genannt. Wir würden uns wünschen, dass Arbeiten häufiger online erwähnt werden, wenn ihre Ergebnisse von hoher Qualität sind. |
